# Concise and Stereoselective Total Syntheses of Annotinolides C, D, and E

**DOI:** 10.1021/jacs.1c05942

**Published:** 2021-08-02

**Authors:** Pei Qu, Scott A. Snyder

**Affiliations:** Department of Chemistry, University of Chicago, 5735 South Ellis Avenue, Chicago, Illinois 60637, United States

## Abstract

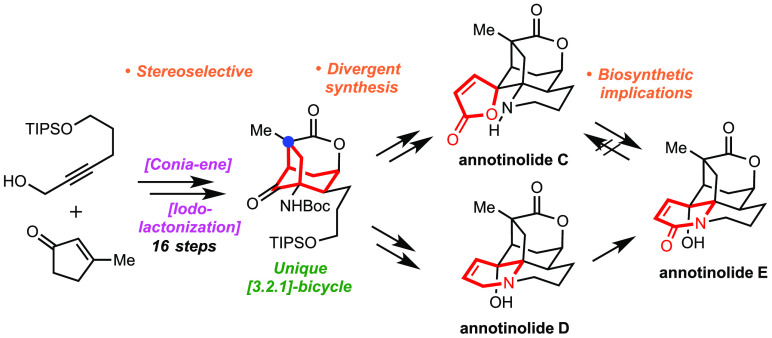

The annotinolides are one of the most recent additions to the *Lycopodium* family of alkaloids, with its members possessing challenging, caged structures that include a [3.2.1]-bicyclic core bearing six contiguous stereocenters, including four that are fully substituted. Herein, we document a concise and stereoselective route that achieves the first total syntheses of three of its members: annotinolides C, D, and E. Key operations include a gold(I)-catalyzed Conia-ene reaction that fashions much of the main core in a single operation, as well as a number of other challenging and chemoselective transformations to generate the remaining elements. Moreover, efforts utilizing the natural products themselves, seeking adjustments in their oxidation states and the rearrangement of individual ring systems, shed light on their potential biogenesis with some outcomes counter to those originally proposed. Finally, formal enantioenriched syntheses of the target molecules are also presented.

In 2016, Zhang, Hu, and co-workers reported the isolation and characterization of a unique collection of alkaloid-based natural products from *Lycopodium annotinium*, including **1**–**3** (Scheme 1).^1^ While structurally related to the famous lycopodine family, of which **4** is the flagship member given its historic isolation in 1881,^2^ these compounds possess a truncated and more highly oxidized [3.2.1]-bicyclic core in contrast to the standard [3.3.1]-framework of the parent class. This domain within the annotinolides, while generally rare, has been found in other alkaloid classes as exemplified by the structures of gelsemine (**5**)^3^ and isopalhinine A (**6**),^4^ and can be quite challenging to fashion as revealed by published syntheses of these particular targets over the past decade. For **1**–**3** specifically, this [3.2.1]-bicycle leads to the presence of 6 contiguous chiral centers within the main core, of which 3 are fully substituted, with 1 being a quaternary center (with the C-12 and C-15 centers exhibiting increased oxidation states versus **4**). The remaining structural variations among the drawn annotinolides reflect further changes in oxidation state and/or peripheral ring patterning. To date, no member of this collection has succumbed to laboratory synthesis, with only a recent model study by Tu illustrating a potential approach to fashion their common core.^5^ Herein, we report the first route capable of achieving their total syntheses through a cohesive strategy featuring several carefully orchestrated transformations to both fashion and manipulate their structural elements despite their intricate and sterically encumbered environments. Additionally, a series of chemical operations performed on the synthesized natural products offer insights into their biosynthetic relationships, in one case suggesting a connection counter to the original biogenetic proposals posited by the isolation team. Finally, a formal asymmetric synthesis of a key building block is presented.

**Scheme 1:**
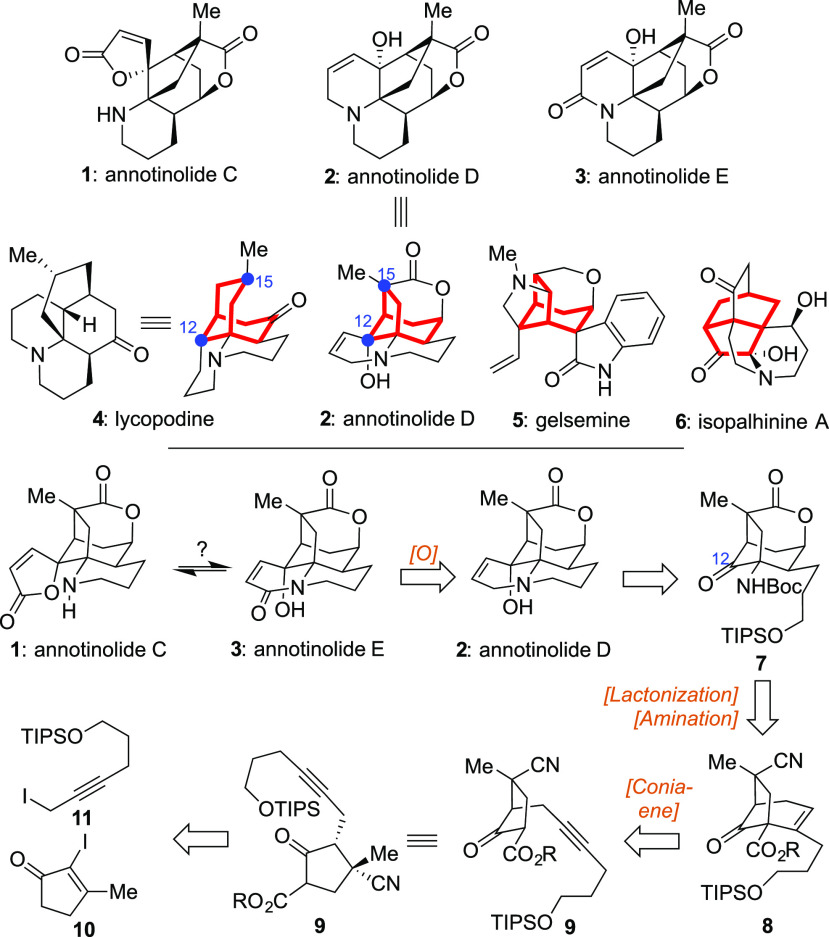
Structures of Selected Annotinolides Possessing a Challenging [3.2.1]-Bicyclic Core Shared with Other Alkaloids and a Strategy To Achieve Their Laboratory Synthesis

Our general strategy, shown in the lower half of Scheme 1, sought a unified approach wherein we could potentially interconvert a given family member into another. Thus, we anticipated annotinolide E (**3**) may be derived from annotinolide D (**2**) through an allylic oxidation, and that annotinolides C (**1**) and E (**3**) might be able to interconvert under appropriate conditions between their respective lactam and lactone forms. Whether one variant would be preferred over the other and/or whether they might exist in equilibrium was unknown, noting that the isolation team proposed that annotinolide E (**3**) may arise from annotinolide D (**2**).^1a^ However, no experimental or computational support for that assertion was provided. Next, if the remaining elements of their ring systems outside of the core [3.2.1]-bicycle were excised, projecting their late-stage incorporation, we then arrived at goal structure **7**. Here, we hoped that the core elements could arise readily from a far simpler cyclopentanone derivative (**9**) as a result of three main operations: (1) a lactonization onto a precursor alkene (using a carboxylic acid derived from a nitrile), (2) conversion of the ester within **8** into an amine through a Curtius or related rearrangement,^6^ and (3) a Conia-ene reaction^7^ to sew up the main bicyclic core. In these events, the formation of each new chiral center in the core, including those that are fully substituted, would be governed by the nitrile-containing all-carbon quaternary center found within **9**.^8^ That intermediate might in turn arise from a diastereoselective vicinal difunctionalization of cyclopentenone **10**.

Our efforts commenced with the preparation of the substrate needed for the key Conia-ene step, starting with the merger of iodide **11** (prepared in 3 steps;^9^ see Supporting Information) and known cyclopentenone **10** (Scheme 2).^10^ That operation, facilitated by the use of *i*-PrMgCl and CuCN·2LiCl using Knochel’s procedure,^11^ proceeded smoothly in 81% yield to afford **12**. Then, similar to Huet’s protocol,^12^ we achieved a synthesis of the 1,4-cyano addition product **13** in 68% yield using *in situ* generated Nagata’s reagent; in this event, the initially obtained silyl enol ether was cleaved under an HCl-promoted workup to afford the desired (and likely thermodynamically favored) *trans*-relationship between the original methyl group and propargyl side chain of **13**. Next, treatment with LDA followed by Mander’s reagent (NCCO_2_Me, **14**) afforded 1,3-dicarbonyl **15** in 64% yield as an inconsequential mixture of diastereomers about the newly formed chiral center.

**Scheme 2:**
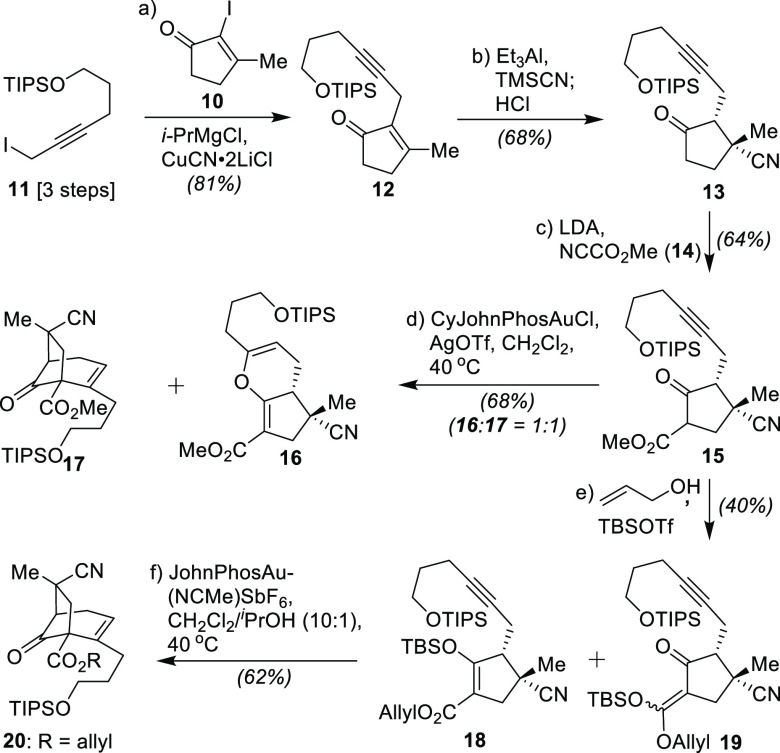
Successful Execution of the Key Conia-Ena Reaction To Generate the Core Framework of the Annotinolides Reagents and conditions: (a) **10** (1.5 equiv), *i*-PrMgCl (1.5 equiv), CuCN·2LiCl (1.5 equiv), THF (0.12 M), −78 to 23 °C, 1.5 h, 81%; (b) Et_3_Al (1.2 equiv), TMSCN (2.2 equiv), hexanes (0.1 M), 60 °C, 1 h, then THF/3 N HCl = 4:1 (0.3 M), 23 °C, 10 min, 68%; (c) LDA (2.1 equiv), NCCO_2_Me (**14**, 1.5 equiv), THF (0.1 M), −78 °C, 1.5 h, 64%; (d) CyJohnPhosAuCl (0.2 equiv), AgOTf (0.2 equiv), CH_2_Cl_2_, 40 °C, 24 h, 68%; (e) toluene/allyl alcohol = 4:1 (0.1 M), 110 °C, 3 h, then Ḧunig’s base (5.0 equiv), TBSOTf (2.0 equiv), CH_2_Cl_2_ (0.1 M), 0.5 h, 40%; (f) JohnPhosAu(NCMe)SbF_6_ (0.3 equiv), CH_2_Cl_2_/*i*-PrOH = 10:1 (0.1 M), 40 °C, 48 h, 62%.

With this critical material in hand, efforts to complete the [3.2.1]-core through a Conia-ene reaction commenced. In practice, this event proved quite challenging to achieve cleanly. Although initial explorations revealed that bicycle **17** could be fashioned from **15** using appropriate promoters,^13^ the best result obtained after extensive screening was a 1:1 mixture of both the desired **17** and the *O*-cyclization product **16** in a combined yield of 68% (Scheme 2). That outcome was achieved using 20 mol % CyJohnPhosAuCl in the presence of 20 mol % AgOTf in refluxing CH_2_Cl_2_ over the course of 24 h.^7c^ Unfortunately, despite this partial success, subsequent efforts to selectively hydrolyze the methyl ester within **17** to set the stage for an eventual Curtius rearrangement proved fruitless, affording only decomposition instead. Given these collated issues, we wondered whether execution of the Conia-ene reaction using a silyl enol ether precursor with a different ester analog might prove superior. Pleasingly, such a substrate could be fashioned in a single step by stirring **15** with allyl alcohol in refluxing toluene^14^ followed by exposure to Hünig’s base and TBSOTf. This operation afforded an inseparable mixture of **18** and **19**, both of which could then be smoothly converted into [3.2.1]-bicycle **20**, the allyl ester variant of **17**, in 25% overall yield. For this Conia-ene transformation, the optimal conditions proved to be treatment with JohnPhosAu(NCMe)SbF_6_ in the absence of an added Ag(I) promoter, with a mixed solvent system^7c^ (CH_2_Cl_2_/*i*-PrOH = 10:1) being essential.

With the main framework secured, efforts shifted next to the structural alterations needed to finalize both the full core and its remaining appended ring systems; executing these operations and effecting certain transformations with proper diastereocontrol required both chemospecific and substrate-based solutions. First, although the ketone moiety within **20** was formally needed to realize the targets (*vide infra*), its presence inflicted undesirable instability within all related intermediates, thus requiring the C-12 ketone to be stereospecifically reduced and protected over two steps to afford **21** (Scheme 3). Next, the allyl ester was selectively cleaved through the action of Pd(PPh_3_)_4_ and pyrrolidine,^15^ allowing for the highly hindered bridgehead nitrogen-containing center to then be forged through a Curtius rearrangement conducted under Fukuyama’s conditions,^16^ delivering the Boc-protected amine **22**. With an eye toward forming the bridged lactone of the annotinolides, the nitrile group was then converted into a carboxylic acid via a standard reduction/Pinnick oxidation sequence to afford **23** in 81% overall yield. Despite the adamantine-like rigidity of this structure, subsequent efforts to directly promote lactonization onto the neighboring alkene using Ag(I) failed.^17^ Pleasingly, when this event was promoted instead with NIS in CH_2_Cl_2_ at 23 °C, polycycle **24** could be formed smoothly (74% yield).

**Scheme 3:**
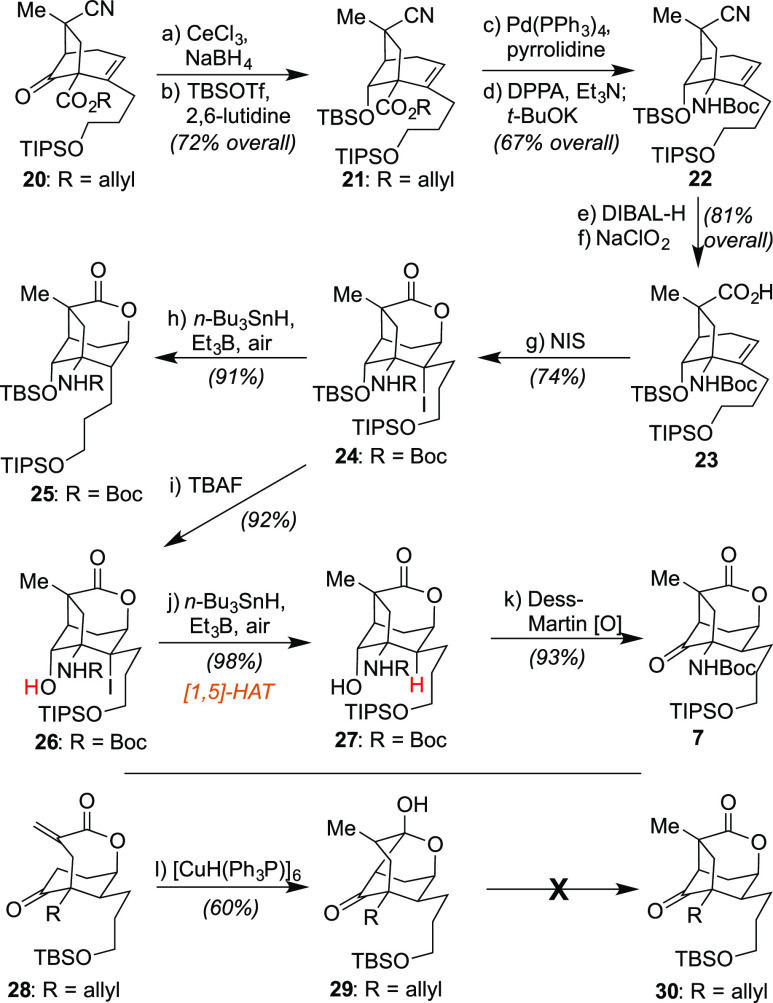
Completion of the [3.2.1]-Core of the Annotinolides Reagents and conditions: (a) CeCl_3_·7H_2_O (1.2 equiv), NaBH_4_ (1.5 equiv), MeOH (0.1 M), 0 to 23 °C, 0.5 h, 84%; (b) 2,6-lutidine (5.0 equiv), TBSOTf (1.5 equiv), CH_2_Cl_2_ (0.1 M), 23 °C, 4 h, 86%; (c) Pd(PPh_3_)_4_ (0.4 equiv), pyrrolidine (1.2 equiv), MeCN (0.1 equiv), 0 to 23 °C, 1 h, 82%; (d) DPPA (1.0 equiv), Et_3_N (2.0 equiv), toluene (0.1 M), 23 °C, 0.5 h; then 110 °C, 1 h; then *t-*BuOK (2.0 equiv), 23 °C, 1 h, 82%; (e) DIBAL-H (4.0 equiv), toluene, 0 °C, 15 min, 85%; (f) NaH_2_PO_4_·2H_2_O (20 equiv), NaClO_2_ (10 equiv), *t-*BuOH/H_2_O/2-methyl-2-butene = 3:3:1 (0.05 M), 23 °C, 40 min, 95%; (g) NIS (10 equiv), CH_2_Cl_2_, 23 °C, 6 h, 74%; (h) *n*-Bu_3_SnH (1.5 equiv), Et_3_B (1.0 equiv), air, toluene (0.05 M), 0 °C, 15 min, 91%; (i) TBAF (1.0 equiv), THF (0.1 M), 0 °C, 15 min, 92%; (j) *n-*Bu_3_SnH (1.5 equiv), Et_3_B (1.0 equiv), air, toluene (0.05 M), 0 °C, 15 min, 98%; (k) Dess−Martin periodinane (2.0 equiv), NaHCO_3_ (10.0 equiv), CH_2_Cl_2_ (0.05 M), 23 °C, 30 min, 93%; (l) [CuH(Ph_3_P)]_6_ (0.5 equiv), toluene (0.5 M), 23 °C, 1 h, 60%.

The price for that facility, however, was the need to remove the target superfluous iodine atom within **24** and replace it, stereospecifically, with a hydrogen atom to retain the desired orientation of the alkyl side chain at the same site. Unfortunately, while that deiodination could be readily achieved using *n*-Bu_3_SnH as promoted by Et_3_B in air, the substrate’s inherent preference for H· recapture was entirely from the undesired face, affording **25** exclusively. Here, though, we hoped that if we removed the neighboring silicon protecting group to afford **26**, the resultant alcohol might be able to afford the desired adduct from the same type of radical intermediate through an intramolecular 1,5-hydrogen atom transfer. That supposition proved true, with compound **27** formed in near quantitative yield under commensurate deiodination conditions. Subsequent Dess–Martin oxidation then completed the assembly of core bicycle **7** in 93% yield. As one reflection of the power of the overall Conia-ene/lactonization strategy to reach **7**, we note that alternative modes of core closure, such as one attempting to utilize an oxidative coupling approach (shown here in the lower part of Scheme 3 with the conversion of **28** into **29**) afforded only [3.3.1]-bicyclic adducts which could not, in our hands, be converted into the desired [3.2.1]-alternatives (i.e., **30**); conditions attempted for the latter were LDA/FeCl_3_ and LDA/CuCl_2_ in THF/DMF at −78 °C.

From key intermediate **7**, two members of the annotinolides were then completed through two final ring-forming processes as shown in Scheme 4. First, to access annotinolide D (**2**), an initial ring closure was effected from **7** via silyl deprotection followed by a one-pot mesylate formation, Boc-removal, and intramolecular S_N_2 cyclization. The second ring closure proceeded through *N*-alkylation and an intramolecular, diastereoselective 1,2-addition initiated by *t*-BuLi. These operations completed a 20-step synthesis of this target from commercial materials, all of whose spectral data matched those originally reported.^1a^ To access annotinolide C (**1**) from **7**, this order of ring closures was reversed, with initial diastereoselective addition of lithium propiolate **34**(18) onto **7** followed by reduction with Lindlar’s catalyst and addition of silica gel affording the lactone ring of **35**. A similar desilylation/S_N_2 ring-closure approach then completed this second target in 20 steps as well.

**Scheme 4:**
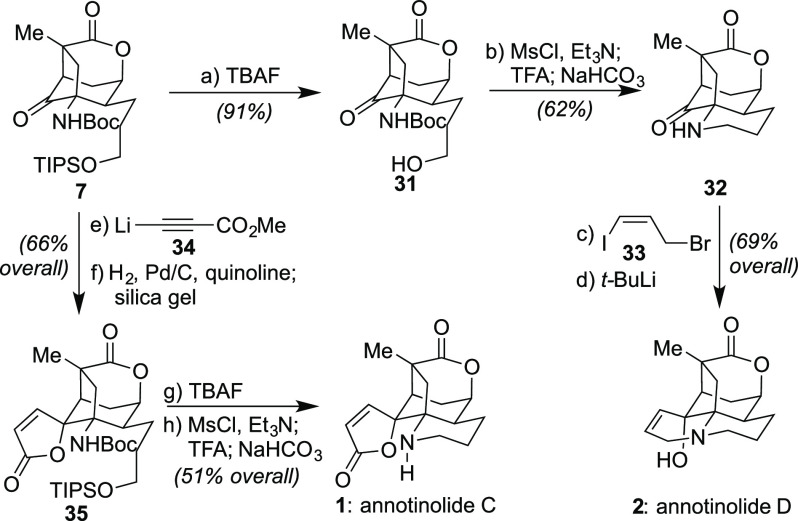
Completion of the Total Syntheses of Annotinolides C and D (**1** and **2**) Reagents and conditions: (a) TBAF (1.5 equiv), THF (0.05 M), 0 to 23 °C, 1 h, 91%; (b) MsCl (3.0 equiv), Et_3_N (10 equiv), CH_2_Cl_2_ (0.05 M), 0 °C, 0.5 h; then CH_2_Cl_2_/TFA = 4:1 (0.05 M), 0 to 23 °C, 1 h; then NaHCO_3_ (excess), 23 °C, 0.5 h, 62%; (c) **33** (5.0 equiv), K_2_CO_3_ (10 equiv), MeCN (0.1 M), 23 °C, 24 h, 87%; (d) *t*-BuLi (2.1 equiv), THF (0.05 M), −78 °C, 0.5 h, 79%; (e) **34** (2.5 equiv), THF (0.05 M), −78 °C, 0.5 h, 84%; (f) Pd/C (0.1 equiv), quinoline (3.0 equiv), H_2_ (balloon pressure), MeOH (0.05 M), 23 °C, 1 h; then silica gel (1.0 g/mmol sub), CH_2_Cl_2_ (0.1 M), 23 °C, 0.5 h, 78%; (g) TBAF (1.5 equiv), THF (0.05 M), 23 °C, 91%; (h) MsCl (3.0 equiv), Et_3_N (10 equiv), CH_2_Cl_2_ (0.05 M), 0 °C, 0.5 h; then CH_2_Cl_2_/TFA = 4:1 (0.05 M), 0 to 23 °C, 1 h; then NaHCO_3_ (excess), 23 °C, 0.5 h, 56%.

Critically, with these two targets in hand, we then explored our ability to interconvert them and access additional members. As expected, annotinolide D (**2**) could be oxidized into annotinolide E (**3**) with KMnO_4_^19^ in a 4:1 mixture of acetone and H_2_O proving optimal (Scheme 5). We then attempted to convert this new material into annotinolide C (**1**) based on the isolation team’s hypothesis that its lactam could rearrange into a lactone.^1a^ However, under the conditions attempted (NaOH/MeOH and 4-DMAP, toluene, 110 °C), we observed no such transformation, only recovered starting material. By contrast, when we exposed annotinolide C (**1**) to variants of these conditions, such as NaOMe in MeOH, we could effect its partial conversion to annotinolide E (**3**, ∼67% conversion, **1**:**3** = 1:1 along with another unknown side product) after 1.5 h of stirring at 23 °C. Other bases, like triazobicyclodecene,^20^ could also achieve this conversion, but with inferior throughput (leading in this case to an ∼4:1 mixture of **1**:**3**). Although we have not yet been able to effect complete conversion under any condition set, these findings to date suggest that **1** and **3** are not in equilibrium under basic conditions, and that **1** is potentially a viable biosynthetic precursor for **3**, but the inverse is unlikely.^21^

**Scheme 5:**
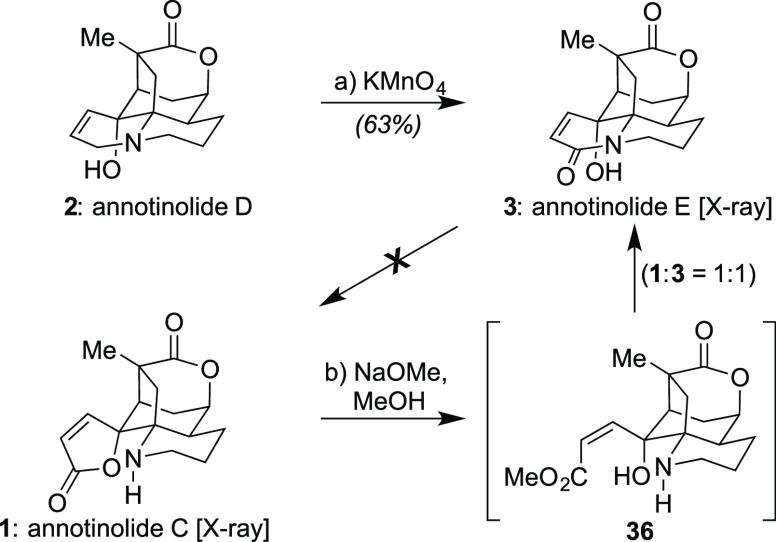
Explorations into Possible Interconversions/Biosynthetic Relationships of the Annotinolides Reagents and conditions: (a) KMnO_4_ (1.5 equiv), acetone/H_2_O = 4:1 (0.01 M), 0 °C, 15 min, 63%; (b) NaOMe (5.0 equiv), MeOH (0.07 M), 23 °C, 1.5 h.

Finally, although our syntheses of the annotinolides were fully diastereoselective, the route presented above afforded racemic material. As a result, we also sought access to enantioenriched supplies of **15** to afford formal asymmetric syntheses. While simply stated, however, our requisite nitrile-containing all carbon-quaternary center was viewed as challenging to fashion as a single enantiomer given literature precedent. Indeed, both the Hesse^22^ and Herzon^23^ groups have shown that under basic conditions these centers in similar rings can both eliminate and/or racemize. Moreover, asymmetric methods to install such nitrile groups directly in a 1,4-fashion are arguably lacking;^24^ for example, as shown in Scheme 6 a recent asymmetric synthesis from the Qin group^25^ relied instead upon a −CH_2_OPiv group as a surrogate for such a cyanide, with substantive functional group changes needed to reach the desired material.

**Scheme 6:**
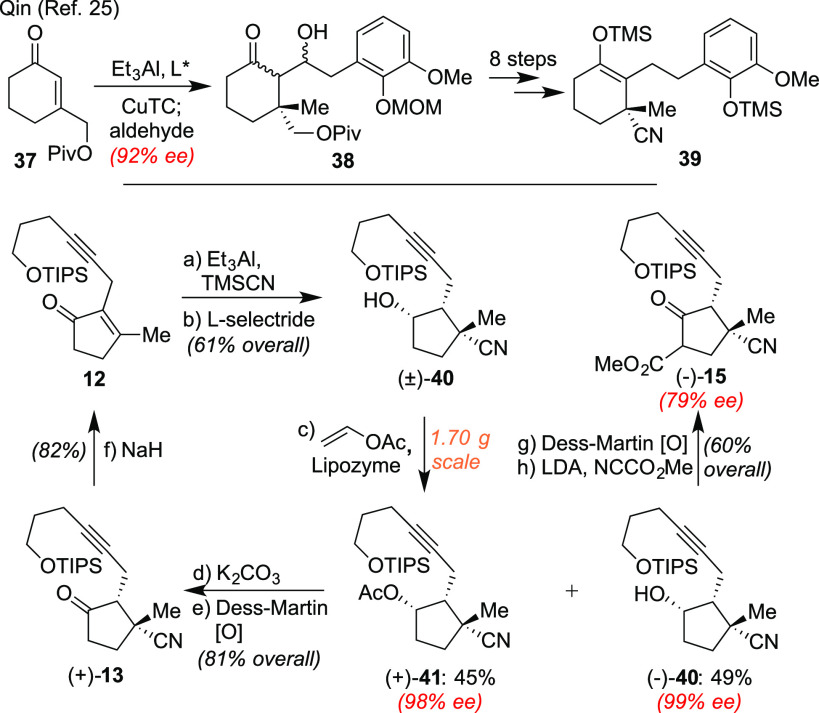
Development of a Formal Asymmetric Route to the Annotinolides and Selected Precedent Highlighting Related Challenges involving Nitrile-Containing All-Carbon Quaternary Centers Reagents and conditions: (a) Et_3_Al (1.2 equiv), TMSCN (2.2 equiv), hexanes (0.1 M), 60 °C, 1 h, then THF/3 N HCl = 4:1 (0.3 M), 23 °C, 10 min, 68%; (b) l-Selectride (1.2 equiv), THF (0.1 M), −78 °C, 30 min, 92%; (c) 4 Å molecular sieves (180 mg/mL solvent), MTBE (0.2 M), vinyl acetate (5.0 equiv), Lipozyme (20 mg/mL solvent), 23 °C, 24 h, 49% (**40**, 99% ee), 45% (**41**, 98% ee); (d) K_2_CO_3_ (10.0 equiv), MeOH (0.02 M), 23 °C, 1 h, 95%; (e) Dess−Martin periodinane (2.0 equiv), NaHCO_3_ (10.0 equiv), CH_2_Cl_2_ (0.1 M), 23 °C, 45 min, 93%; (f) NaH (5.0 equiv), THF (0.1 M), 0 °C, 1 h, 82%; (g) Dess−Martin periodinane (2.0 equiv), CH_2_Cl_2_ (0.1 M), 23 °C, 45 min, 96% (95% ee); (h) LDA (1.9 equiv), THF (0.1 M), −78 °C, 30 min, then NCCO_2_Me (1.5 equiv), −78 °C, 1 h, 62% (79% ee).

Thus, what we perceived as the most efficient approach was an enzymatic resolution. As shown, this idea worked with great facility following the generation of racemic **40** from **12**, using lipozyme^26^ and the indicated acetate source to generate a separable mixture of (+)-**41** and (−)-**40** in near-perfect yield and *ee*. The undesired acetate could be smoothly recycled to **12** through ester hydrolysis, alcohol oxidation, and base-promoted nitrile expulsion. By contrast, (−)-**40** could be advanced into (−)-**15** via oxidation and α-esterification. Unfortunately, these latter processes both proceeded with some degree of racemization, with the oxidation affording an intermediate with a measured *ee* value of 95% and the subsequent lithiation step eroding that further to 79% *ee*. Extensive screening of both operations (see SI for details) revealed that superior results could not be obtained. Pleasingly, though, chiral materials of commensurate *ee* values could be produced reliably and on scale through this general protocol.

In conclusion, we have synthesized three members of the annotinolide family through an approach that leveraged the presence of an initial cyanide-containing quaternary center in a cyclopentanone precursor to stereospecifically fashion the remaining rings and chiral centers of these formidable caged compounds. Critical operations leading to that success include the following: (1) a challenging Conia-ene reaction to forge the entire [3.2.1]-core in a single operation using silyl enol ethers to circumvent alternate modes of cyclization, (2) subsequent use of the ester to generate a hindered aza-quaternary center through a Curtius rearrangement, (3) intramolecular 1,5-hydrogen atom transfer to finalize the core lactone motif, and (4) diastereoselective additions and terminating cyclizations to fashion the periphery. In addition, we established a viable means for the interconversion of two family members and developed a scalable route to access our key cyanide-containing building block in an enantioenriched fashion. Efforts to extend the lessons learned from these endeavors to other members of the family, as well as additional alkaloid classes, are the subject of current study.

## Supporting Information Available

Detailed experimental procedures, copies of all spectral data, cif files, and full characterization (PDF)

## Accession Codes

CCDC 2089512–2089514 contain the supplementary crystallographic data for this paper. These data can be obtained free of charge via www.ccdc.cam.ac.uk/data_request/cif, or by emailing data_request@ccdc.cam.ac.uk, or by contacting The Cambridge Crystallographic Data Centre, 12 Union Road, Cambridge CB2 1EZ, UK; fax: +44 1223 336033.
