# Correction to “Concise and Stereoselective Total Syntheses of Annotinolides C, D, and E”

**DOI:** 10.1021/jacs.1c08386

**Published:** 2021-10-05

**Authors:** Pei Qu, Scott A. Snyder

We wish to clarify that because we have not determined the absolute configuration of our isolated individual enantiomers (of compounds such as **40** and **41**), we have drawn these species with the same relative orientation so as not to imply a defined identity for them.

In addition, following the initial appearance of this work, we realized that the hydride transfer converting **26** into **27**, as drawn in Scheme 3, would actually reflect a [1,4]-hydride transfer, which, while known, is generally rare. An alternate explanation for the observed outcome could be a [1,6]-hydride transfer pathway. For this manifold to be active, Et_3_B would need to react with the free alcohol to form a borinate, followed by intramolecular hydrogen abstraction of a C–H atom on a methylene adjacent to the boron. See, for example, ref (1).

**Scheme 3:**
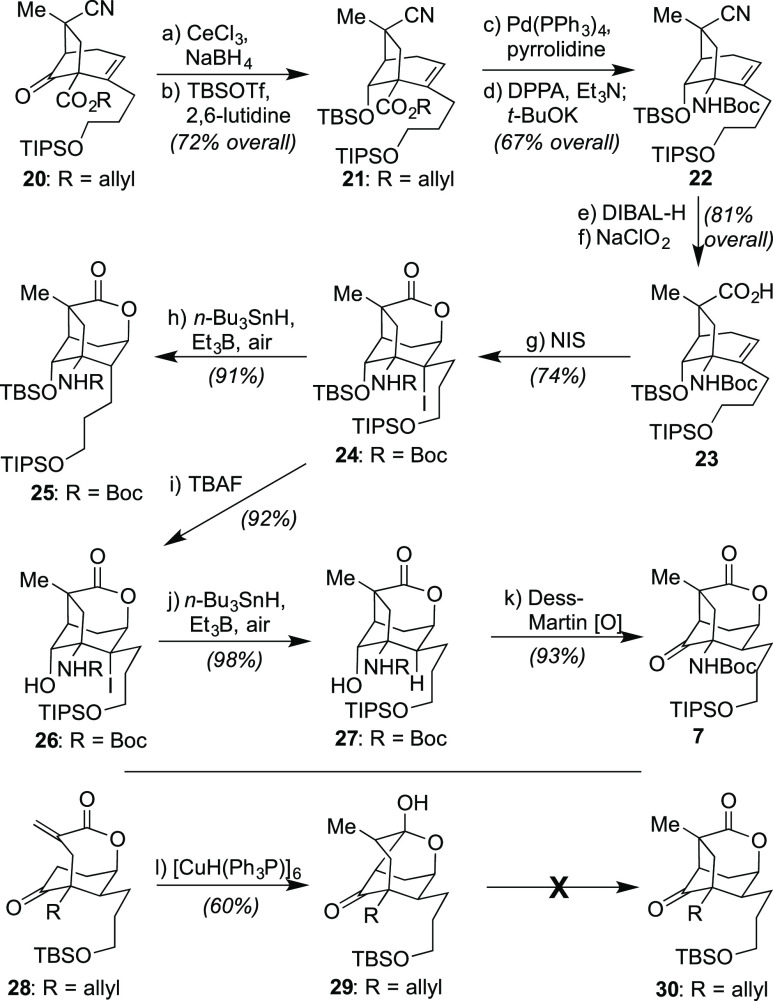
Completion of the [3.2.1]-Core of the Annotinolides Reagents and conditions: (a) CeCl_3_·7H_2_O (1.2 equiv), NaBH_4_ (1.5 equiv), MeOH (0.1 M), 0 to 23 °C, 0.5 h, 84%; (b) 2,6-lutidine (5.0 equiv), TBSOTf (1.5 equiv), CH_2_Cl_2_ (0.1 M), 23 °C, 4 h, 86%; (c) Pd(PPh_3_)_4_ (0.4 equiv), pyrrolidine (1.2 equiv), MeCN (0.1 equiv), 0 to 23 °C, 1 h, 82%; (d) DPPA (1.0 equiv), Et_3_N (2.0 equiv), toluene (0.1 M), 23 °C, 0.5 h; then 110 °C, 1 h; then *t*-BuOK (2.0 equiv), 23 °C, 1 h, 82%; (e) DIBAL-H (4.0 equiv), toluene, 0 °C, 15 min, 85%; (f) NaH_2_PO_4_·2H_2_O (20 equiv), NaClO_2_ (10 equiv), *t*-BuOH/H_2_O/2-methyl-2-butene = 3:3:1 (0.05 M), 23 °C, 40 min, 95%; (g) NIS (10 equiv), CH_2_Cl_2_, 23 °C, 6 h, 74%; (h) *n*-Bu_3_SnH (1.5 equiv), Et_3_B (1.0 equiv), air, toluene (0.05 M), 0 °C, 15 min, 91%; (i) TBAF (1.0 equiv), THF (0.1 M), 0 °C, 15 min, 92%; (j) *n*-Bu_3_SnH (1.5 equiv), Et_3_B (1.0 equiv), air, toluene (0.05 M), 0 °C, 15 min, 98%; (k) Dess–Martin periodinane (2.0 equiv), NaHCO_3_ (10.0 equiv), CH_2_Cl_2_ (0.05 M), 23 °C, 30 min, 93%; (l) [CuH(Ph_3_P)]_6_ (0.5 equiv), toluene (0.5 M), 23 °C, 1 h, 60%.
