# Supplementary material for: Concise and Stereoselective Total Syntheses of Annotinolides C, D, and E
Source: J Am Chem Soc. 2021 Aug 2;143(31):11951–6. doi: 10.1021/jacs.1c05942 (PMC8397315; doi:10.1021/jacs.1c05942)
Supplement: Supplementary file 1 — ja1c05942_si_001.pdf [file ja1c05942_si_001.pdf]

# Concise and Stereoselective Total Syntheses of Annotinolides C, D, and E

Pei Qu and Scott A. Snyder*

[1]Department of Chemistry, The University of Chicago, 5735 S. Ellis Avenue, Chicago, IL 60637

## Supporting Information

Table of Contents

General procedures S2
Abbreviations S2
Synthesis of ketone **12** S3
Exploration of Conia-ene reaction S6
Synthesis of key common intermediate ketone **7** S10
Syntheses of annotinolide D and annotinolide C S17
Transformations between annotinolide C, D and E S25
Asymmetric synthesis of **15** S30
References S41
$^{1}$H and $^{13}$C NMR data for selected intermediates S42
Crystal experiment section S102

## Experimental Data for Compounds

**General Procedures.** All reactions were carried out under an argon atmosphere with dry solvents under anhydrous conditions, unless otherwise noted. Dry tetrahydrofuran (THF), toluene, dimethylformamide (DMF), diethyl ether ($Et_2O$) and dichloromethane ($CH_2Cl_2$) were obtained by passing commercially available pre-dried, oxygen-free formulations through activated alumina columns. Yields refer to chromatographically and spectroscopically ($^1H$ and $^{13}C$ NMR) homogeneous materials, unless otherwise stated. Steps refer to operations conducted in a single reaction flask; filtration, extraction, or other form of purification denotes the end of an individual step. Reagents were purchased at the highest commercial quality and used without further purification, unless otherwise stated. Reactions were magnetically stirred and monitored by thin-layer chromatography (TLC) carried out on 0.25 mm E. Merck silica gel plates (60F-254) using UV light as visualizing agent, and an ethanolic solution of phosphomolybdic acid and cerium sulfate, and heat as developing agents. SiliCycle silica gel (60, academic grade, particle size 0.040–0.063 mm) was used for flash column chromatography. Preparative thin-layer chromatography separations were carried out on 0.50 mm E. Merck silica gel plates (60F-254). NMR spectra were recorded on Bruker 400, 500 and 700 MHz instruments and calibrated using residual undeuterated solvent as an internal reference. The following abbreviations were used to explain the multiplicities: s = singlet, d = doublet, t = triplet, q = quartet, br = broad, m = multiplet, app = apparent. IR spectra were recorded on a Perkin-Elmer 1000 series FT-IR spectrometer. High-resolution mass spectra (HRMS) were recorded on Agilent 6244 Tof-MS using ESI (Electrospray Ionization) at the University of Chicago Mass Spectroscopy Core Facility.

**Abbreviations.** TIPSCl = triisopropylsilyl chloride, MeOH = methanol, THF = tetrahydrofuran, EtOAc = ethyl acetate, *n*-BuLi = *n*-butyl lithium, $Ph_3P$ = triphenyl phosphine, *i*-PrMgCl = isopropyl magnesium chloride, $Et_3Al$ = triethyl aluminum, TMSCN = trimethylsilyl cyanide, LDA = lithium diisopropylamide, *i*-PrNH = diisopropyl amine, TBSOTf = tert-butyel dimethyl trifluoromethanesulfonate, *i*-$Pr_2NEt$ = diisopropyl ethyl amine, $Ph_3PAuNTf_2$ = [bis(trifluoromethanesulfonyl)imidate] (triphenylphosphine)gold(I), AgOTf = silver trifluoromethanesulfonate, *t*-BuOH = tert-butyl alcohol, *i*-PrOH = isopropanol, $Pd(Ph_3P)_4$ = tetrakis(triphenylphosphine)palladium(0), MeCN = acetonitrile, DPPA = diphenylphosphoryl azide, $Et_3N$ = triethyl amine, *t*-BuOK = potassium tert-butoxide, DIBAL-H = diisobutylaluminum hydride, NIS = *N*-iodosuccinimide, $Et_3B$ = triethyl borane, *n*-$Bu_3SnH$ = tributyltin hydride, TBAF = tetrabutylammonium fluoride, DMP = Dess–Martin periodinane, MsCl = methanesulfonyl chloride, TFA = trifluoroacetic acid, *t*-BuLi = tert-butyl lithium, MeONa = sodium methoxide, TBD = triazabicyclodecene, L-selectride = lithium tri-*sec*-butylborohydride, MTBE = Methyl-tert-butyl ether, MS = molecular sieves, Ac = acetyl, BzCl = benzoyl chloride, 4-DMAP = 4-(dimethylamino)pyridine, PCC = pyridinium chlorochromate, DMSO = dimethyl sulfoxide, LiHMDS = lithium hexamethyldisilazide, NaHMDS = sodium hexamethyldisilazide, KHMDS = potassium hexamethyldisilazide, LiTMP = lithium tetramethylpiperidide.

**Synthesis of ketone 12**

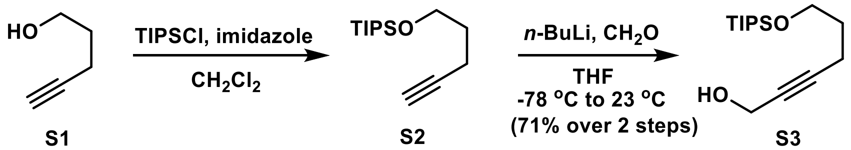


**Propargyl alcohol S3.** To a solution of 4-penty-1-ol (16.8 g, 0.200 mol, 1.0 equiv) in $CH_2Cl_2$ (400 mL) at 23 °C was sequentially added imidazole (20.4 g, 0.300 mol, 1.5 equiv) and TIPSCl (51.4 mL, 0.240 mol, 1.2 equiv), after which a white participate formed. The resultant suspension was then stirred at 23 °C for 4 h. Next, MeOH (1.72 mL, 1.36 g, 0.0400 mol) was added and the reaction contents were stirred for an additional 30 min. Upon completion, the reaction contents were filtered through a pad of Celite (eluting with hexanes) and concentrated directly. Pressing forward without any further purification, the so-obtained TIPS-protected alcohol was dissolved in THF (1.05 L) and the reaction contents were cooled to –78 °C. Next, *n*-BuLi (100 mL, 2.5 M in hexane, 0.250 mol, 1.25 equiv) was then added at –78 °C via cannula over the course of 5 min, during which time the solution turned bright yellow. The reaction contents were then stirred for an additional 30 min at –78 °C. Solid paraformaldehyde (12.7 g, 0.420 mol, 2.1 equiv) was then added to the solution in a single portion, and the resultant suspension was then slowly warmed to 23 °C and stirred for 12 h. Upon completion, the reaction contents were quenched by the addition of saturated aqueous $NH_4Cl$ (600 mL) and poured into a seapratory funnel. After separating the layers, the aqueous phase was extracted with EtOAc (2 × 600 mL). The combined organic layers were then washed with brine (1 L), dried ($Na_2SO_4$), filtered, and concentrated. Purification of the resultant residue by flash chromatography chromatography (silica gel, hexanes/EtOAc = 10:1) provided the desired propargyl alcohol (38.4 g, 71% yield over 2 steps) as a pale-yellow oil. Its spectral data matched that previously reported.[1]

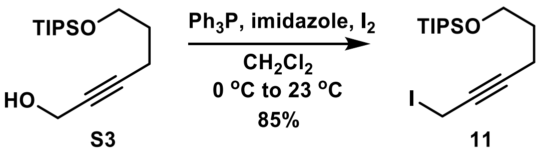


**Propargyl iodide 11.** To a flame-dried flask containing $CH_2Cl_2$ (800 mL) at 23 °C was sequentially added $Ph_3P$ (45.86 g, 0.175 mol, 1.2 equiv) and imidazole (11.9 g, 0.175 mol, 1.2 equiv). The resultant solution was then cooled to 0 °C using an ice-water bath and $I_2$ (44.4 g, 0.175 mol, 1.2 equiv) was added in a single portion, forming an orange-brown suspension. The resultant suspension was then stirred at 0 °C for 30 min before a solution of propargyl alcohol (39.4 g, 0.146 mol, 1.0 equiv) in $CH_2Cl_2$ (200 mL) was added, rinse that flask with a minimal amount of $CH_2Cl_2$ to ensure a complete transfer. Next, the ice-water bath was removed, at which time the suspension turned bright yellow. After stirring the resultant suspension at 23 °C for 1 h, the reaction was filtered directly through Celite (eluting with hexanes) and concentrated. The resultant residue was redissolved in hexanes (600 mL) and filtered a second time through a pad of Celite (eluting with hexanes) again. The resultant filtrate was concentrated and purification of the resultant residue by flash chromatography chromatography (silica gel, hexanes/EtOAc = 50:1), provided the desired propargyl iodid**e** (47.0 g, 85% yield) as a yellow oil. **11**: $R_f$ = 0.87

(silica gel, hexanes/EtOAc, 4:1); IR (film) $\nu_{max}$ 2930, 2892, 2866, 2361, 2339, 1464, 1171, 1109, 680 cm$^{-1}$; $^1$H NMR (500 MHz, $CDCl_3$) δ 3.76 (t, $J$ = 6.0 Hz, 2 H), 3.70 (t, $J$ = 2.5 Hz, 2 H), 2.32 (tt, $J$ = 7.1, 2.5 Hz, 2 H), 1.72 (p, $J$ = 6.6 Hz, 2 H), 1.11–1.06 (m, 21 H); $^{13}$C NMR (125 MHz, $CDCl_3$) δ 86.4, 77.1, 61.7, 31.6, 18.0, 15.6, 12.0, –16.9; HRMS (ESI) calcd for $C_{15}H_{30}IOSi^+$ [M + $H^+$] 380.1032, found 380.1030.

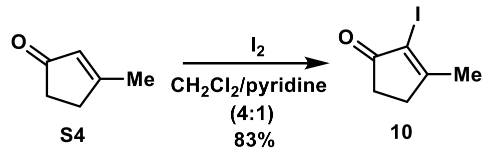


**Iodide 10.** A flame-dried flask at 23 °C was charged sequentially with 3-methyl-2-cyclopenten-1-one (9.81 mL, 9.61 g, 0.100 mol, 1.0 equiv), $CH_2Cl_2$ (800 mL), and pyridine (200 mL). Next, $I_2$ (55.9 g, 0.220 mol, 2.2 equiv) was added, forming a dark-brown solution. The resultant mixture was then stirred at 23 °C for 48 h. Upon completion, the reaction contents were quenched the addition of saturated aqueous $Na_2S_2O_3$ (600 mL) and poured into a separatory funnel. After separating the layers, the organic phase was washed with 3 N HCl (1 L), $H_2O$ (600 mL), and brine (600 mL). The organic layer was then dried ($Na_2SO_4$), filtered, and concentrated. Purification of the resultant residue by flash column chromatography (silica gel, hexanes/EtOAc = 4:1→2:1) afforded the desired iodide (18.5 g, 83% yield) as a pale-yellow solid. Its spectral data matched that previously reported.[2]

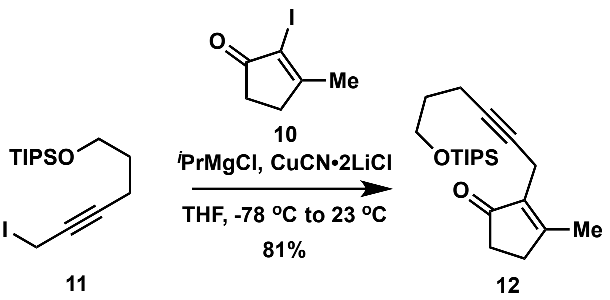


**CuCN•2LiCl solution (1.0 M in THF).** A round-bottom flask equipped with a stir bar at 23 °C was charged with LiCl (8.10 g, 0.191 mol, 3.0 equiv). Next, the flask and its contents were dried with a propane torch under vacuum until the appearance of the LiCl became a sand-like solid without chunks. The flask was then removed from the vacuum line, flushing with argon, and then THF (96 mL) was added followed by CuCN (8.56 g, 95.6 mmol, 1.5 equiv). The resultant cloudy green solution was stirred at 23 °C for 1 h, at which point it was ready for use.

**Enone 12.** Iodide **10** (21.2 g, 95.6 mmol, 1.5 equiv) was dissolved in THF (350 mL) in a flame-dried flask at 23 °C and then was cooled to –78 °C using a dry ice-acetone bath, forming a yellow suspension. Next, *i*-PrMgCl (2.0 M in THF, 47.8 mL, 95.6 mmol, 1.5 equiv) was added dropwise at –78 °C, during which time the yellow suspension turned into a pale brown solution. After stirring the resultant solution for 30 min at –78 °C, freshly prepared CuCN•2LiCl (1.0 M in

THF, 96 mL, 95.6 mmol, 1.5 equiv) was added, and the resultant gray/green suspension was stirred for a further 15 min at –78 °C. Next, a solution of propargyl iodide **11** (24.2 g, 63.7 mmol, 1.0 equiv) in THF (40 mL) was added to the suspension, rinsing the flask with additional THF (10 mL) to ensure a complete transfer. Once the transfer was complete, the cold bath was removed and the suspension was slowly warmed to 23 °C over the course of 30 min with stirring, during which time the suspension turned brown. Upon completion, the reaction contents were quenched by the sequential addition of saturated aqueous $NH_4Cl$ (200 mL) and 3 M NaOH (200 mL) and poured into a separatory funnel. After separating the layers, the aqueous layer was extracted with EtOAc (2 × 400 mL). The combined organic layers were then washed with brine (800 mL), dried ($Na_2SO_4$), filtered, and concentrated. Purification of the resultant residue by flash column chromatography (silica gel, hexanes/EtOAc = 10:1→4:1), providing the desired enone (17.9 g, 81% yield) as a yellow oil. **12**: $R_f$ = 0.29 (silica gel, hexanes/EtOAc, 4:1); IR (film) $\nu_{max}$ 2942, 2865, 2360, 2339, 1700, 1653, 1457, 1107, 668 $cm^{-1}$; $^1$H NMR (500 MHz, $CDCl_3$) δ 3.72 (t, *J* = 6.1 Hz, 2 H), 3.06 (s, 2 H), 2.58–2.47 (m, 2 H), 2.44–2.34 (m, 2 H), 2.23 (tt, *J* = 7.1, 2.5 Hz, 2 H), 2.17 (s, 3 H), 1.68 (p, *J* = 6.7 Hz, 2 H), 1.06–1.03 (m, 21 H); $^{13}$C NMR (125 MHz, $CDCl_3$) δ 207.6, 171.8, 136.1, 79.7, 76.0, 61.8, 33.9, 32.0, 31.6, 17.9, 17.3, 15.1, 12.5, 11.8; HRMS (ESI) calcd for $C_{21}H_{37}O_2Si^+$ [M + $H^+$] 349.2558, found 349.2553.

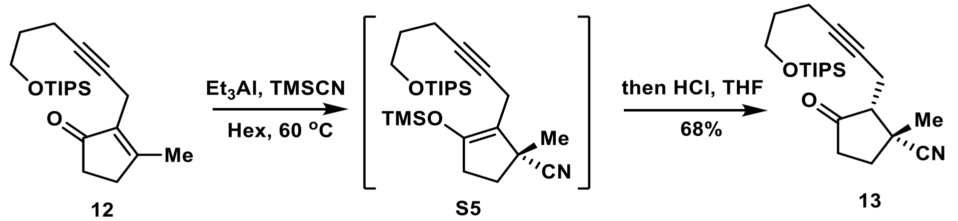


**Nitrile 13.** $Et_3Al$ (1.0 M in heptane, 40.4 mL, 40.4 mmol, 1.2 equiv) and hexanes (19 mL) were added sequentially to a flame-dried flask at 23 °C. Next, TMSCN (9.2 mL, 74.0 mmol, 2.2 equiv) was added and the resultant colorless solution was stirred for 15 min at 23 °C. A solution of enone **12** (11.7 g, 33.6 mmol, 1.0 equiv) in hexanes (200 mL) was then added, using an additional portion of hexanes (60 mL) to complete the transfer. The reaction solution turned a red-brown color and then was warmed to 60 °C using a pre-heated oil bath. After stirring the resultant solution at 60 °C for 1 h. Upon completion, the reaction contents were then cooled to 0 °C using an ice-water bath and quenched by the addition of $H_2O$ until no bubble formation was observed from the solution. The reaction contents were then warmed to 23 °C and stirred for 30 min before being filtered through a pad of $Na_2SO_4$ (eluting with hexanes) and concentrated directly. The resultant crude silyl enol ether was then dissolved in THF (100 mL) and 3 M HCl (25 mL) was added at 23 °C. The resultant solution was stirred at 23 °C until the presence of the silyl enol ether had disappeared based on TLC monitoring (typically 10 min). Upon completion, the reaction contents were quenched by the addition of $H_2O$ (25 mL) and poured into a separatory funnel. After separating the layers, the aqueous layer was extracted with EtOAc (2 × 100 mL). The combined organic layers were then washed with brine (300 mL), dried ($Na_2SO_4$), filtered, and concentrated. Purification of the resultant crude product by flash column chromatography (silica gel, hexanes/EtOAc = 10:1→4:1) providing the desired nitrile (8.67 g, 68% yield) as a pale brown oil. [Note: this compound contains a minor impurity which produces

signals at 2.70 ppm as well as a few other sites; removal of these consistent impurities did not prove possible with several different developing solvents, even preparative TLC with different solvents and collecting different portions of the main band. In all cases, we observed no additional impurity by TLC but these signals are always present based on NMR analysis. We also attempted silyl deprotection/reprotection and reduction/oxidation to reform this compound without that impurity, but no fruitful results were obtained]. **13**: $R_f$ = 0.29 (silica gel, hexanes/EtOAc, 4:1); IR (film) $\nu_{max}$ 2943, 2892, 2866, 2235, 1751, 1490, 1246, 1108 $cm^{-1}$; $^1H$ NMR (500 MHz, $CDCl_3$) δ 3.73 (t, *J* = 6.0 Hz, 2 H), 2.87 (ddq, *J* = 17.4, 4.1, 2.2 Hz, 1 H), 2.53 (ddt, *J* = 12.9, 8.0, 2.0 Hz, 1 H), 2.46–2.40 (m, 2 H), 2.35 (ddt, *J* = 19.6, 12.2, 2.6 Hz, 1 H), 2.25 (td, *J* = 7.2, 3.4 Hz, 2 H), 2.14 (dd, *J* = 10.1, 3.7 Hz, 1 H), 1.86 (ddd, *J* = 12.8, 11.2, 8.9 Hz, 1 H), 1.74 (d, *J* = 1.2 Hz, 3 H), 1.72–1.66 (m, 2 H), 1.09–1.00 (m, 21 H); $^{13}C$ NMR (125 MHz, $CDCl_3$) δ 212.6, 121.3, 82.3, 76.2, 61.8, 57.5, 41.7, 35.2, 34.2, 31.9, 25.3, 17.9, 16.7, 15.1, 11.9; HRMS (ESI) calcd for $C_{22}H_{38}NO_2Si^+$ [M + $H^+$] 376.2667, found 376.2667.

**Exploration of Conia-ene reaction**

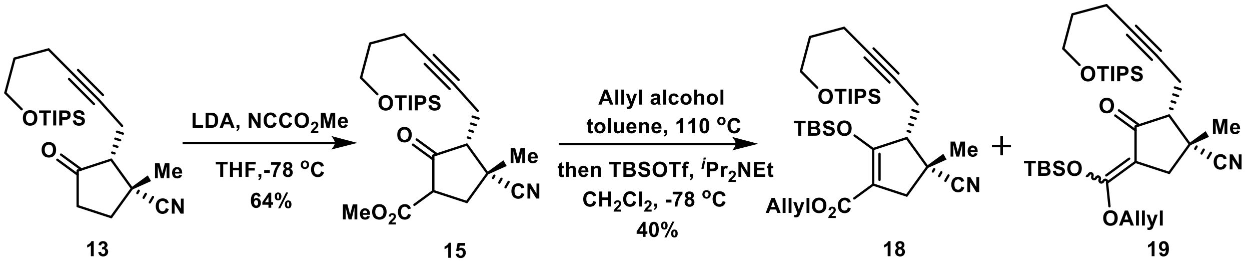


**Silyl enol ether 18 and 19.** To a flame-dried flask at 23 °C was added *i*-$Pr_2NH$ (3.71 mL, 26.5 mmol, 2.1 equiv) and THF (27 mL), and the resultant solution was cooled to 0 °C using an ice-water bath. Next, *n*-BuLi (1.6 M in hexanes, 16.6 mL, 26.5 mmol, 2.1 equiv) was added dropwise, generating a colorless solution. After stirring the reaction contents for 10 min at 0 °C, the ice-water bath was exchanged for a dry ice-acetone bath to cool the solution to –78 °C. A solution of nitrile **13** (4.74 g, 12.6 mmol, 1.0 equiv) in THF (90 mL) was then added quickly, using an additional portion of THF (10 mL) to complete the transfer. The reaction solution turned a red-brown color and was stirred for an additional 30 min at –78 °C before Mander's reagent (**14**, 1.52 mL, 18.9 mmol, 1.5 equiv) was added dropwise. The resultant solution was then stirred at –78 °C for another 1 h. Upon completion, the reaction contents were quenched at –78 °C by the addition of saturated aqueous $NH_4Cl$ (100 mL) and warmed to 23 °C. The contents were then poured into a separatory funnel and the layers were separated. The aqueous layer was further extracted with EtOAc (2 × 100 mL). The combined organic layers were then washed with brine (300 mL), dried ($Na_2SO_4$), filtered, and concentrated. Purification of the resultant residue by flash column chromatography (silica gel, hexanes/EtOAc = 8:1→4:1) provided the desired β-ketoester **15** (3.51 g, 64% yield) as a pale-yellow oil and as an inseparable mixture of diastereomers, also with enol form based on NMR analysis. **15**: $R_f$ = 0.29 (silica gel, hexanes/EtOAc, 4:1); IR (film) $\nu_{max}$ 2943, 2893, 2865, 2230, 1734, 1717, 1705, 1635, 1464, 1386, 1254, 1124, 672 $cm^{-1}$; $^1H$ NMR (500 MHz, $CHCl_3$, list major two diastereomers) δ 3.79–

3.76 (m, 3 H), 3.76–3.72 (m, 2 H), 3.47 (dd, $J$ = 12.2, 8.4 Hz, 0.71 H), 3.26 (dd, $J$ = 11.4, 8.7 Hz, 0.28 H), 3.07 (dd, $J$ = 14.0, 5.4 Hz, 0.28 H), 2.94–2.88 (m, 1 H), 2.88–2.80 (m, 0.28 H), 2.78–2.72 (m, 1 H), 2.72–2.67 (m, 0.28 H), 2.66–2.56 (m, 0.71 H), 2.55–2.49 (m, 0.56 H), 2.39 (ddt, $J$ = 16.9, 10.1, 2.3 Hz, 1 H), 2.35–2.33 (m, 1 H), 2.30–2.23 (m, 3 H), 2.22–2.14 (m, 0.56 H), 1.81 (s, 2 H), 1.77–1.67 (m, 3 H), 1.08–1.01 (m, 21 H); $^{13}$C NMR (125 MHz, $CDCl_3$) δ 205.3, 168.1, 120.8, 82.9, 75.6, 61.8, 57.4, 53.0, 52.3, 51.5, 51.2, 50.8, 39.8, 37.7, 32.1, 32.0, 31.7, 25.0, 19.2, 18.0, 16.8, 15.2, 15.1, 15.0, 12.2, 12.0; HRMS (ESI) calcd for $C_{24}H_{40}NO_4Si^+$ [M + H$^+$] 434.2721, found 434.2719.

Pushing forward, the newly formed β-ketoester **15** was dissolved in a mixture of toluene (40 mL) and allyl alcohol (10 mL), and the reaction contents were then heated directly to 110 °C using a pre-heated oil bath. After stirring at 110 °C for 3 h, the reaction contents were then cooled to 23 °C and concentrated directly. Finally, the resultant crude product was then dissolved in $CH_2Cl_2$ (81 mL) and *i*-$Pr_2NEt$ (7.04 mL, 40.4 mmol, 5.0 equiv) was added at 23 °C. The resultant solution was then cooled to –78 °C using a dry ice-acetone bath and TBSOTf (3.72 mL, 16.2 mmol, 2.0 equiv) was added dropwise. After stirring the resultant solution for 30 min at –78 °C, the reaction was quenched by the addition of saturated aqueous $NaHCO_3$ (60 mL). The reaction contents were then warmed to 23 °C, poured into a separatory funnel, and the layers were separated. The aqueous layer was further extracted with $CH_2Cl_2$ (2 × 60 mL). The combined organic layers were then washed with brine (150 mL), dried ($Na_2SO_4$), filtered, and concentrated. Purification of the resultant residue by flash column chromatography (silica gel, hexanes/EtOAc = 20:1), providing a mixture of silyl enol ethers **18** and **19** (1.86 g, 40% yield) as a pale-yellow oil. **18** and **19**: $R_f$ = 0.59 (silica gel, hexanes/EtOAc, 4:1); IR (film) $\nu_{max}$ 2942, 2893, 2865, 2232, 1720, 1703, 1635, 1463, 1390, 1251, 1231, 1133, 1108, 1067, 1057, 1013, 995, 841, 788 cm$^{-1}$; $^1$H NMR (400 MHz, $C_6D_6$) δ 5.73 (ddtd, $J$ = 18.0, 10.7, 5.6, 2.0 Hz, 1 H), 5.09 (ddt, $J$ = 17.2, 3.6, 1.7 Hz, 1 H), 4.97 (ddt, $J$ = 10.4, 2.7, 1.4 Hz, 1 H), 4.45 (dd, $J$ = 5.6, 1.5 Hz, 2 H), 3.71–3.60 (m, 2 H), 3.24 (dd, $J$ = 14.9, 1.9 Hz, 0.67 H), 2.98–2.87 (m, 0.67 H), 2.68 (ddt, $J$ = 17.1, 4.5, 2.4 Hz, 0.67 H), 2.61–2.54 (m, 0.67 H), 2.51 (dd, $J$ = 15.0, 1.6 Hz, 0.33 H), 2.40–2.31 (m, 1 H), 2.22 (tq, $J$ = 6.9, 2.4 Hz, 2 H), 2.14 (ddd, $J$ = 6.2, 4.0, 1.5 Hz, 0.67 H), 2.10 (dt, $J$ = 9.2, 2.4 Hz, 0.33 H), 1.70–1.61 (m, 2 H), 1.17 (s, 1 H), 1.11–1.06 (m, 23 H), 0.95–0.90 (m, 9 H), 0.31 (d, $J$ = 18.3 Hz, 3 H), 0.15 (d, $J$ = 17.8 Hz, 3 H); $^{13}$C NMR (100 MHz, $C_6D_6$) δ 163.70, 163.67, 163.65, 163.1, 133.64, 133.61, 125.9, 122.9, 118.2, 107.5, 106.5, 83.9, 83.7, 76.67, 76.65, 64.9, 62.8, 57.1, 55.2, 42.5, 42.2, 37.2, 36.5, 33.0, 32.9, 27.7, 26.5, 26.44, 21.1, 20.2, 19.29, 19.25, 18.89, 18.87, 18.7, 17.9, 16.2, 16.1, 12.9, –3.2, –3.3, –3.6, –3.8; HRMS (ESI) calcd for $C_{32}H_{55}NNaO_4Si_2^+$ [M + Na$^+$] 596.3562, found 596.3560.

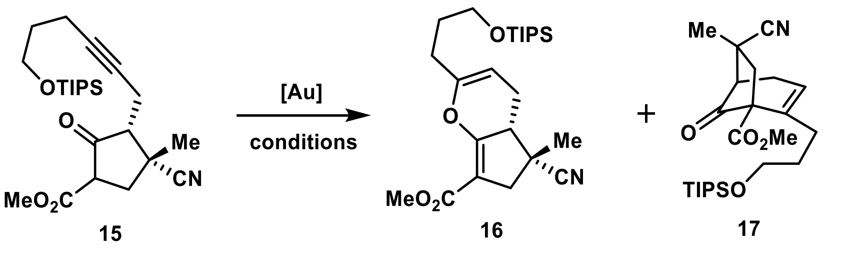

**General procedure for Conia-ene reactions performed as part of condition screening:** To a flame-dried flask at 23 °C was added a solution of substrate **15** (1.0 equiv) in the indicated solvent (at a final concentration of 0.10 M). The indicated gold salt (0.20 equiv) and (if applicable) silver salt (0.20 equiv) were then added subsequently, forming a pale-yellow suspension. If needed, the suspension was then directly placed in pre-heated oil bath, and the resultant solution was either stirred at 23 °C or 40 °C for 24 h. Upon completion, the reaction contents were filtered through a pad of Celite (eluting with $CH_2Cl_2$). The filtrate was then concentrated directly and characterized by NMR analysis. **16**: $R_f$ = 0.33 (silica gel, hexanes/EtOAc, 4:1); IR (film) $\nu_{max}$ 2943, 2891, 2866, 2235, 1752, 1675, 1652, 1472, 1457, 1237, 1104, 882 cm$^{-1}$; $^1$H NMR (500 MHz, $CDCl_3$) δ 4.91 (dd, *J* = 6.4, 2.3 Hz, 1 H), 3.77 (s, 3 H), 3.75 (t, *J* = 6.3 Hz, 2 H), 3.19 (dd, *J* = 15.0, 1.4 Hz, 1 H), 2.82–2.74 (m, 1 H), 2.65 (dd, *J* = 14.9, 2.2 Hz, 1 H), 2.50–2.43 (m, 1 H), 2.42–2.32 (m, 3 H), 1.81 (p, *J* = 6.8 Hz, 2 H), 1.59 (s, 3 H), 1.15–1.03 (m, 21 H); $^{13}$C NMR (126 MHz, $CDCl_3$) δ 164.1, 159.0, 152.4, 122.7, 102.0, 95.9, 62.3, 51.2, 46.9, 41.2, 40.8, 29.2, 24.2, 21.8, 18.0, 12.0; HRMS (ESI) calcd for $C_{24}H_{39}NNaO_4Si^+$ [M + Na$^+$] 456.2541, found 456.2545. **17**: $R_f$ = 0.31 (silica gel, hexanes/EtOAc, 4:1); IR (film) $\nu_{max}$ 2943, 2892, 2866, 2237, 1768, 1737, 1652, 1470, 1455, 1233, 1104, 882 cm$^{-1}$; $^1$H NMR (500 MHz, $CDCl_3$) δ 5.51 (br s, 1 H), 3.82 (s, 3 H), 3.72 (t, *J* = 6.0 Hz, 2 H), 3.05 (d, *J* = 18.2 Hz, 1 H), 2.95 (d, *J* = 18.1 Hz, 1 H), 2.88 (d, *J* = 13.9 Hz, 1 H), 2.61 (d, *J* = 13.8 Hz, 1 H), 2.45 (t, *J* = 3.4 Hz, 1 H), 2.33–2.20 (m, 1 H), 2.15–2.04 (m, 1 H), 1.78–1.63 (m, 2 H), 1.51 (s, 3 H), 1.10–1.02 (m, 21 H); $^{13}$C NMR (126 MHz, $CDCl_3$) δ 205.0, 168.2, 143.4, 122.7, 119.4, 62.5, 62.1, 53.1, 52.6, 46.5, 36.5, 34.0, 31.0, 29.9, 28.4, 18.0, 11.9; HRMS (ESI) calcd for $C_{24}H_{39}NNaO_4Si^+$ [M + Na$^+$] 456.2541, found 456.2542.

***Table S1.*** Condition screening for Conia-ene reaction of **15**

| Entry | Catalyst | Condition | Result |
|---|---|---|---|
| 1 | $Ph_3PAuNTf_2$ | $CH_2Cl_2$, 23 °C | 16:17 = 4:1 |
| 2 | $Ph_3PAuCl/AgOTf$ | $CH_2Cl_2$, 23 °C | Only 16 |
| 3 | CyJohnPhosAuCl/AgOTf | $CH_2Cl_2$, 40 °C | 16:17 = 2:1 |
| 4 | CyJohnPhosAuCl/AgOTf | tol, 40 °C | 16:17 = 1:1 |
| 5 | CyJohnPhosAuCl/AgOTf | tol/$^t$BuOH, 40 °C | 16:17 = 10:1 |
| 6 | CyJohnPhosAuCl/AgOTf | acetone, 40 °C | Only 16 |
| 7 | CyJohnPhosAuCl/AgOTf | $CH_3CN$, 40 °C | Unkown product |
| 8 | CyJohnPhosAuCl/$AgBF_4$ | tol, 40 °C | 16:17 = 8:1 |
| 9 | CyJohnPhosAuCl/$AgSbF_6$ | tol, 40 °C | 16:17 = 10:1 |
| 10 | $LAuNTf_2$ (0.2 eq.) | tol, 40 °C | NR |

CyJohnphos:

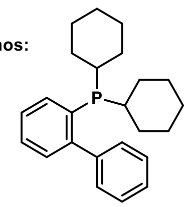


L

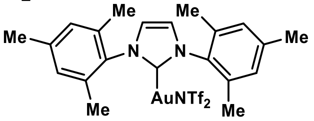

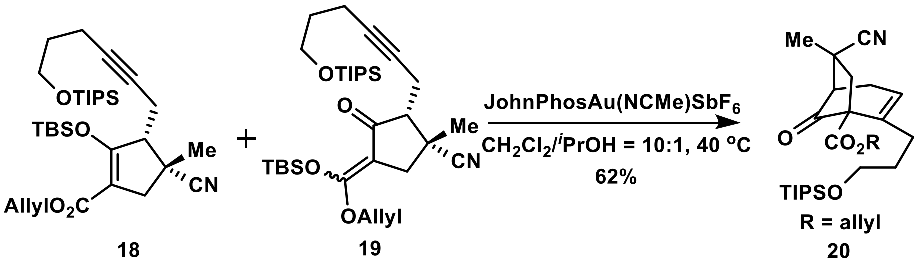

**β-ketoester 20.** A mixture of silyl enol ethers **18** and **19** (2.98 g, 5.20 mmol, 1.0 equiv) were dissolved in $CH_2Cl_2$ (47 mL) and *i*-PrOH (4.7 mL) at 23 °C and then JohnPhosAu(NCMe)$SbF_6$ (1.20 g, 1.56 mmol, 0.30 equiv) was added. The resultant solution was then warmed to 40 °C using a pre-heated oil bath and stirred at that temperature for 48 h, during which time the original yellow solution gradually turned a dark brown and a participate formed. Once the reaction appeared complete, as judged by no obvious turnover based on TLC analysis, the reaction contents were cooled to 23 °C and concentrated directly. Purified the resultant residue by flash column chromatography (silica gel, hexanes/EtOAc = 16:1→4:1), providing the desired β-ketoester (1.48 g, 62% yield) as a pale brown oil. **20**: $R_f$ = 0.43 (silica gel, hexanes/EtOAc, 4:1); IR (film) $\nu_{max}$ 2943, 2892, 2867, 2238, 1768, 1734, 1652, 1463, 1386, 1248, 1106, 995, 882 $cm^{-1}$; $^1H$ NMR (400 MHz, $CDCl_3$) δ 5.93 (ddt, *J* = 17.3, 10.4, 5.9 Hz, 1 H), 5.50 (dd, *J* = 4.5, 2.3 Hz, 1 H), 5.38 (dq, *J* = 17.2, 1.5 Hz, 1 H), 5.27 (dt, *J* = 10.4, 1.3 Hz, 1 H), 4.74 (ddt, *J* = 13.1, 6.0, 1.4 Hz, 1 H), 4.66 (ddt, *J* = 13.1, 6.0, 1.4 Hz, 1 H), 3.69 (t, *J* = 6.0 Hz, 2 H), 3.03 (ddd, *J* = 18.1, 4.4, 2.2 Hz, 1 H), 2.98–2.90 (m, 1 H), 2.87 (d, *J* = 13.8 Hz, 1 H), 2.60 (d, *J* = 13.8 Hz, 1 H), 2.44 (dd, *J* = 4.4, 2.5 Hz, 1 H), 2.30–2.19 (m, 1 H), 2.17–2.06 (m, 1 H), 1.69 (dddd, *J* = 10.1, 7.8, 6.8, 3.7 Hz, 2 H), 1.50 (s, 3 H), 1.07–1.03 (m, 21 H); $^{13}C$ NMR (100 MHz, $CDCl_3$) δ 204.8, 167.4, 143.3, 131.4, 122.7, 119.5, 119.2, 66.4, 62.5, 62.0, 53.0, 46.5, 36.5, 33.9, 31.1, 29.9, 28.4, 25.9, 18.0, 12.0, 11.9; HRMS (ESI) calcd for $C_{26}H_{42}NO_4Si^+$ [M + $H^+$] 460.2878, found 460.2876.

**General procedure for Conia-ene reactions performed as part of condition screening:** To a flame-dried flask at 23 °C was added a solution of substrate mixture **18** and **19** (1.0 equiv) in the indicated solvent (at a final concentration of 0.10 M). The indicated gold salt (0.20 equiv) and (if applicable) silver salt (0.20 equiv) were then added subsequently, forming a pale-yellow suspension. If needed, the suspension was then directly placed in pre-heated oil bath, and the resultant solution was either stirred at 23 °C or 40 °C for 48 h. Upon completion or no further conversion based on TLC analysis, the reaction contents were filtered through a pad of Celite (eluting with $CH_2Cl_2$). The filtrate was then concentrated and purified by flash column chromatography (silica gel, hexanes/EtOAc = 16:1→4:1).

***Table S2.*** Condition screening for Conia-ene reaction of **18** and **19**

| | Catalyst | Condition | Result |
|---|---|---|---|
| 1 | $Ph_3PAuNTf_2$ (0.2 eq.) | $CH_2Cl_2$, 40 °C | 20% (42% brsm) |
| 2 | $Ph_3PAuCl/AgSbF_6$ (0.2 eq.) | $CH_2Cl_2/^iPrOH$, 40 °C | low conversion |
| 3 | JohnPhosAu(NCMe)$SbF_6$ (0.2 eq.) | acetone, 40 °C | 50% (55% brsm) |
| 4 | $Ph_3PAuNTf_2$ (0.2 eq.) | tol/$^t$BuOH, 40 °C | 22% (31% brsm) |
| 5[a] | JohnPhosAu(NCMe)$SbF_6$ (0.2 eq.) | acetone, 40 °C | 30% conversion |
| 6[a] | JohnPhosAu(NCMe)$SbF_6$ (0.2 eq.) | acetone, 23 °C | N. R. |
| 7[a] | $Ph_3PAuNTf_2$ (0.2 eq.) | $CH_2Cl_2/^iPrOH$, 23 °C | N. R. |
| 8 | JohnPhosAu(NCMe)$SbF_6$ (0.3 eq.) | $CH_2Cl_2/^iPrOH$, 40 °C | 62% |

a: with additives 4 Å MS and 2,4,6-tri-tert-butylpyrimidine

## Synthesis of key common intermediate ketone 7

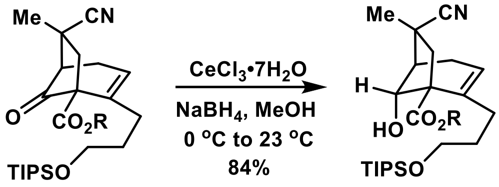

R = allyl
**20**
R = allyl
**S6**

**Alcohol S6.** β-ketoester **20**(1.48 g, 3.22 mmol, 1.0 equiv) was dissolved in MeOH (32 mL) at 23 °C and then $CeCl_3•7H_2O$ (1.44 g, 3.86 mmol, 1.2 equiv) was added. After all the solids had dissolved, the resultant solution was then cooled to 0 °C using an ice-water bath and $NaBH_4$ (0.180 g, 4.83 mmol, 1.5 equiv) was added in a single portion. After the solution stopped bubbling, the reaction contents were stirred at 0 °C for another 5 min and then the ice-water bath was removed. The resultant contents were then stirred at 23 °C for 30 min. Upon completion, the reaction contents were diluted by the addition of $CH_2Cl_2$ (32 mL) and quenched with saturated aqueous $NH_4Cl$ (60 mL). The reaction contents were the poured into a separatory funnel and the resultant layers were separated. The aqueous layer was then further extracted with $CH_2Cl_2$ (3 × 60 mL). The combined organic layers were then washed with brine (120 mL), dried ($Na_2SO_4$), filtered, and concentrated. Purification of the resultant residue by flash column chromatography (silica gel, hexanes/EtOAc = 4:1→2:1) provided the desired alcohol (1.25 g, 84% yield) as a colorless oil. **S6**: $R_f$ = 0.19 (silica gel, hexanes/EtOAc, 4:1); IR (film) $\nu_{max}$ 3527, 2942, 2892, 2866, 2235, 1734, 1717, 1669, 1457, 1437, 1382, 1292, 1248, 1105, 1072, 883 cm$^{-1}$; $^1$H NMR (400 MHz, $CDCl_3$) δ 5.91 (ddt, *J* = 16.5, 10.3, 6.0 Hz, 1 H), 5.62–5.49 (m, 1 H), 5.35 (dq, *J* = 17.2, 1.5 Hz, 1 H), 5.30–5.24 (m, 1 H), 4.65 (ddt, *J* = 5.7, 2.6, 1.3 Hz, 2 H), 4.23 (dd, *J* = 5.5, 1.8 Hz, 1 H), 3.67 (t, *J* = 6.1 Hz, 2 H), 3.17 (d, *J* = 2.8 Hz, 1 H), 2.74–2.66 (m, 1 H), 2.63 (d, *J* = 13.9 Hz, 1 H), 2.41 (ddd, *J* = 18.4, 4.6, 2.2 Hz, 1 H), 2.25 (dt, *J* = 5.2, 2.6 Hz, 1 H), 2.17 (d, *J* = 13.8 Hz, 1 H), 2.02 (h, *J* = 5.4, 4.2 Hz, 2 H), 1.73–1.62 (m, 2 H), 1.50 (s, 3 H), 1.09–0.99 (m, 21 H); $^{13}$C NMR (100 MHz, $CDCl_3$) δ 173.3, 136.1, 131.2, 124.0, 120.6, 119.6, 71.7, 65.9, 62.6,

56.5, 47.6, 45.8, 35.9, 30.9, 29.8, 28.9, 27.7, 18.0, 11.9; HRMS (ESI) calcd for $C_{26}H_{44}NO_4Si^+$ [M + $H^+$] 462.3034, found 462.3032.

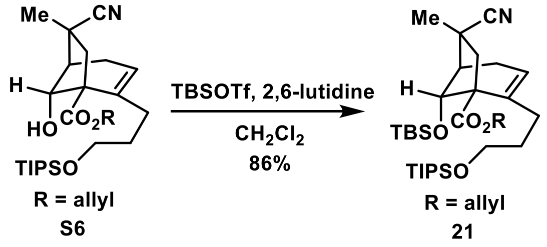


**Silyl ether 21.** Alcohol **S6** (1.25 g, 2.71 mmol, 1.0 equiv) was dissolved in $CH_2Cl_2$ (27 mL) and 2,6-lutidine (1.57 mL, 13.5 mmol, 5.0 equiv) was added at 23 °C. Next, TBSOTf (0.93 mL, 4.06 mmol, 1.5 equiv) was added dropwise. The resultant solution was then stirred for 4 h Stirred the reaction for 4 h at 23 °C. Upon completion, the reaction contents were quenched by the addition of saturated aqueous $NaHCO_3$ (25 mL), poured into a separatory funnel, and the resultant layers were separated. The aqueous layer was then further extracted with (2 × 30 mL). The combined organic layers were then washed with brine (90 mL), dried ($Na_2SO_4$), filtered, and concentrated. Purification of the resultant crude residue by flash column chromatography (silica gel, hexanes/EtOAc = 16:1) provided the desired silyl ether (1.34 g, 86% yield) as a colorless oil. **21**: $R_f$ = 0.69 (silica gel, hexanes/EtOAc, 4:1); IR (film) $\nu_{max}$ 2939, 2892, 2865, 2235, 1733, 1653, 1472, 1457, 1247, 1138, 1102, 873, 838 cm$^{-1}$; $^1$H NMR (500 MHz, $CDCl_3$) δ 5.92 (ddt, *J* = 16.7, 10.3, 6.1 Hz, 1 H), 5.46 (br s, 1 H), 5.38–5.28 (m, 1 H), 5.24 (dq, *J* = 10.4, 1.3 Hz, 1 H), 4.66 (ddt, *J* = 13.1, 6.0, 1.4 Hz, 1 H), 4.50 (ddt, *J* = 13.0, 6.1, 1.4 Hz, 1 H), 4.20 (d, *J* = 5.3 Hz, 1 H), 3.71–3.62 (m, 2 H), 2.64 (d, *J* = 17.9 Hz, 1 H), 2.50 (d, *J* = 13.8 Hz, 1 H), 2.39–2.27 (m, 2 H), 2.23–2.13 (m, 1 H), 2.05 (s, 2 H), 1.66 (ddt, *J* = 24.6, 12.3, 6.1 Hz, 2 H), 1.52 (s, 3 H), 1.08–1.00 (m, 21 H), 0.87 (s, 9 H), 0.03 (d, *J* = 5.2 Hz, 6 H); $^{13}$C NMR (100 MHz, $CDCl_3$) δ 172.4, 137.0, 131.8, 124.3, 119.7, 119.1, 73.5, 65.8, 62.9, 57.4, 48.1, 47.6, 35.7, 31.5, 31.2, 3.1, 29.1, 28.0, 26.0, 25.7, 18.0, 11.9, –5.0, –5.1; HRMS (ESI) calcd for $C_{32}H_{58}NO_4Si_2^+$ [M + $H^+$] 576.3899, found 576.3896.

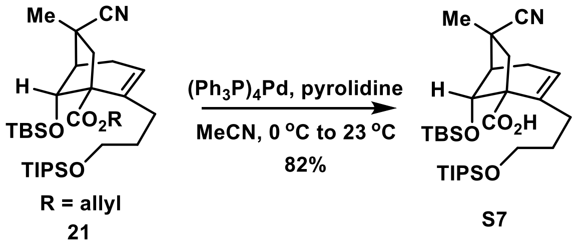


**Carboxylic acid S7.** Silyl ether **21** (1.34 g, 2.33 mmol, 1.0 equiv) was dissolved in MeCN (23 mL) and pyrrolidine (0.229 mL, 2.79 mmol, 1.2 equiv) was added at 23 °C. The resultant solution was then cooled to 0 °C using an ice-water bath, and $Pd(Ph_3P)_4$ (1.07 g, 0.930 mmol, 0.4 equiv) was added. After the reaction contents were stirred at 0 °C for 5 min, the ice-water bath was removed and the reaction contents were stirred at 23 °C for 1 h. Upon completion, the reaction contents were diluted with EtOAc (10 mL) and quenched by the addition of 3 M HCl (20 mL). The reaction contents were then poured into a separatory funnel

and the resultant layers were separated. The aqueous layer was further extracted with EtOAc (3 × 30 mL). The combined organic layers were then washed with brine (90 mL), dried ($Na_2SO_4$), filtered, and concentrated. Purification of the resultant residue by flash column chromatography (silica gel, hexanes/EtOAc = 6:1→4:1) provided the desired carboxylic acid (1.03 g, 82% yield) as a white solid. **S7**: $R_f$ = 0.57 (silica gel, hexanes/EtOAc, 4:1); IR (film) $\nu_{max}$ 2940, 2892, 2865, 2236, 1700, 1653, 1457, 1436, 1254, 1140, 1108, 872, 838 cm$^{-1}$; $^1$H NMR (400 MHz, $CDCl_3$) δ 5.56 (s, 1 H), 4.24 (d, *J* = 5.3 Hz, 1 H), 3.69 (td, *J* = 6.3, 2.5 Hz, 2 H), 2.63–2.53 (m, 2 H), 2.45 (d, *J* = 18.4 Hz, 1 H), 2.21–1.98 (m, 4 H), 1.76–1.65 (m, 2 H), 1.52 (s, 3 H), 1.10–1.02 (m, 21 H), 0.92 (s, 9 H), 0.15 (s, 6 H); $^{13}$C NMR (100 MHz, $CDCl_3$) δ 174.9, 135.8, 123.8, 120.2, 72.8, 62.6, 57.2, 16.9, 46.7, 35.7, 31.17, 29.5, 29.0, 27.9, 25.6, 18, 11.9, –4.6, –5.3; HRMS (ESI) calcd for $C_{29}H_{54}NO_4Si_2^+$ [M + H$^+$] 536.3586, found 536.3587.

***Figure S1.*** X-ray structure of carboxylic acid **S7**

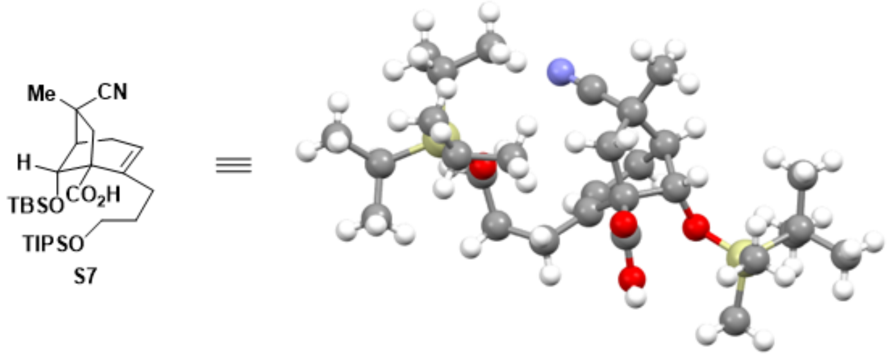


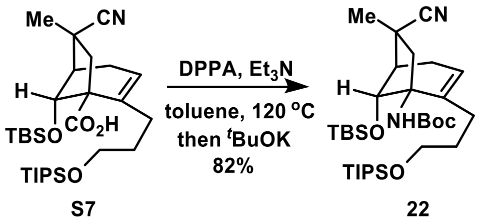


**Boc amide 22.** Carboxylic acid **S7** (1.03 g, 1.92 mmol, 1.0 equiv) was dissolved in toluene (20 mL) at 23 °C and then $Et_3N$ (0.535 mL, 3.84 mmol, 2.0 equiv) and diphenyl phosphoryl azide (0.620 mL, 2.88 mmol, 1.5 equiv) were added sequentially. After stirring the resultant solution for 30 min at 23 °C, the reaction contents were heated at 120 °C using a pre-heated oil bath and stirred for an additional 1 h at that temperature. Upon completion, the reaction contents were cooled to 23 °C and *t*-BuOK (1.0 M in *t*-BuOH, 3.84 mL, 3.84 mmol, 2.0 equiv) was added and the reaction contents were stirred for another 1 h at 23 °C, during which time the color of the reaction solution transformed into a pale yellow suspension. Upon completion, the reaction contents were quenched by the addition of saturated aqueous $NH_4Cl$ (20 mL). The reaction contents were then poured into a separatory funnel and the resultant layers were separated. The aqueous layer was further extracted with EtOAc (2 × 20 mL). The combined organic layers were then washed with brine (50 mL), dried ($Na_2SO_4$), filtered, and

concentrated. Purification of the resultant residue by flash column chromatography (silica gel, hexanes/EtOAc = 10:1) provided the desired Boc amide intermediate (0.971 g, 82% yield) as a colorless oil. **22**: $R_f$ = 0.62 (silica gel, hexanes/EtOAc, 4:1); IR (film) $\nu_{max}$ 3470, 3438, 2940, 2894, 2865, 2235, 1719, 1496, 1472, 1463, 1390, 1366, 1250, 1165, 1135, 1105, 1010, 880, 838 cm$^{-1}$; $^1$H NMR (400 MHz, $CDCl_3$) δ 5.41–5.32 (m, 1 H), 4.68 (s, 1 H), 4.40 (d, *J* = 5.2 Hz, 1 H), 3.70 (t, *J* = 6.2 Hz, 2 H), 2.64 (d, *J* = 13.4 Hz, 1 H), 2.56–2.46 (m, 2 H), 2.32 (ddd, *J* = 18.0, 4.4, 2.2 Hz, 1 H), 2.14–2.06 (m, 2 H), 2.00–1.93 (m, 1 H), 1.67 (ddd, *J* = 14.0, 7.8, 6.2 Hz, 2 H), 1.54 (s, 3 H), 1.42 (s, 9 H), 1.05 (s, 21 H), 0.88 (s, 9 H), 0.08 (d, *J* = 7.5 Hz, 6 H); $^{13}$C NMR (100 MHz, $CDCl_3$) δ 154.8, 139.4, 124.5, 119.8, 79.2, 72.0, 63.0, 62.7, 47.1, 45.9, 35.7, 32.1, 29.2, 28.4, 28.1, 27.5, 25.6, 18.0, 17.9, 11.9, –4.8, –5.0; HRMS (ESI) calcd for $C_{33}H_{62}N_2NaO_4Si_2^+$ [M + Na$^+$] 629.4140, found 629.4137.

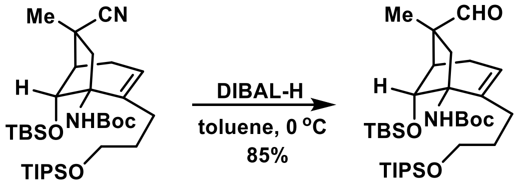

**22** **S8**

**Aldehyde S8.** The Boc amide intermediate **22** (0.960 g, 1.58 mmol, 1.0 equiv) was dissolved in toluene (16 mL) and then the reaction solution was cooled to 0 °C using an ice-water bath. Next, DIBAL-H (20 wt % in toluene, 5.27 mL, 6.32 mmol, 4.0 equiv) was added dropwise, and then the reaction contents were stirred for an additional 15 min at 0 °C. Upon completion, the reaction contents were quenched by the addition of saturated aqueous Rochelle's salt (16 mL). The resultant biphasic reaction contents were then warmed to 23 °C and stirred for 40 min until both layers became clear. The reaction contents were then poured into a separatory funnel and the resultant layers were separated. The aqueous layer was further extracted with EtOAc (2 × 15 mL). The combined organic layers were then washed with brine (20 mL), dried ($Na_2SO_4$), filtered, and concentrated. Purification of the resultant residue by flash column chromatography (silica gel, hexanes/EtOAc = 10:1), providing the desired aldehyde (0.828 g, 85% yield) as a colorless oil. **S8**: $R_f$ = 0.65 (silica gel, hexanes/EtOAc, 4:1); IR (film) $\nu_{max}$ 3437, 2930, 2894, 2865, 2712, 1724, 1720, 1506, 1496, 1472, 1457, 1388, 1366, 1250, 1168, 1136, 1101, 884, 837 cm$^{-1}$; $^1$H NMR (400 MHz, $CDCl_3$) δ 9.60 (s, 1H), 5.17 (dt, *J* = 3.9, 2.0 Hz, 1 H), 4.72 (s, 1 H), 4.42 (s, 1 H), 3.69 (t, *J* = 6.3 Hz, 2 H), 2.78 (d, *J* = 13.5 Hz, 1 H), 2.38 (dt, *J* = 18.0, 3.4 Hz, 1 H), 2.19–2.07 (m, 3 H), 2.04–1.99 (m, 1 H), 1.91 (ddd, *J* = 18.2, 4.6, 2.1 Hz, 1 H), 1.71–1.61 (m, 2 H), 1.43 (s, 9 H), 1.23 (s, 3H), 1.12–0.97 (m, 21 H), 0.88 (s, 9 H), 0.10 (s, 3 H), 0.07 (s, 3 H); $^{13}$C NMR (100 MHz, $CDCl_3$) δ 205.5, 155.0, 140.8, 118.6, 79.0, 74.0, 63.5, 62.9, 49.6, 46.7, 41.8, 32.3, 28.4, 27.6, 25.7, 25.6, 24.9, 18.0, 18.0, 12.0, –4.7, –4.9; HRMS (ESI) calcd for $C_{33}H_{63}NNaO_5Si_2^+$ [M + Na$^+$] 632.4137, found 632.4138.

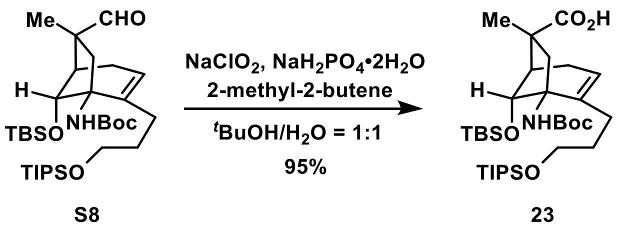


**Carboxylic acid 23.** Aldehyde **S8** (0.825 g, 1.35 mmol, 1.0 equiv) was dissolved in *t*-BuOH (13.5 mL) at 23 °C and then $H_2O$ (13.5 mL), 2-methyl-2-butene (4.5 mL), and $NaH_2PO_4$•$2H_2O$ (4.21 g, 27.0 mmol, 20 equiv) were added sequentially. After all the solids had dissolved, $NaClO_2$ (1.22 g, 13.5 mmol, 10 equiv) was then added and the initially cloudy solution turned yellow in color. The resultant solution was stirred for an additional 40 min at 23 °C for 40 min during which time it became colorless. Upon completion, the reaction contents were diluted with EtOAc (15 mL), poured into a separatory funnel, and the layers were separated. The aqueous layer was further extracted with EtOAc (2 × 15 mL). The combined organic layers were then washed with brine (20 mL), dried ($Na_2SO_4$), filtered, and concentrated. Purification of the resultant residue by flash column chromatography (silica gel, hexanes/EtOAc = 4:1) provided the desired carboxylic acid (0.801 g, 95% yield) as a colorless oil. **23**: $R_f$ = 0.30 (silica gel, hexanes/EtOAc, 4:1); IR (film) $\nu_{max}$ 3470, 2940, 2893, 2865, 1718, 1654, 1496, 1463, 1390, 1251, 1165, 1127, 1104, 880, 837 $cm^{-1}$; $^1H$ NMR (500 MHz, $CDCl_3$) δ 5.14 (d, *J* = 3.8 Hz, 1 H), 4.71 (s, 1 H), 4.59 (s, 1 H), 3.67 (t, *J* = 6.3 Hz, 2 H), 2.89 (d, *J* = 13.4 Hz, 1 H), 2.37 (d, *J* = 17.8 Hz, 1 H), 2.20 (d, *J* = 13.4 Hz, 1 H), 2.09–1.95 (m, 4 H), 1.64 (p, *J* = 7.0 Hz, 2 H), 1.48 (s, 3 H), 1.42 (s, 9 H), 1.11–0.97 (m, 21 H), 0.86 (s, 9 H), 0.08 (s, 3 H), 0.06 (s, 3 H); $^{13}C$ NMR (125 MHz, $CDCl_3$) δ 182.1, 154.8, 139.2, 119.0, 78.8, 73.0, 72.9, 68.0, 63.3, 62.9, 47.6, 46.9, 42.8, 32.1, 29.7, 29.5, 28.4, 27.7, 26.6, 25.7, 18.0, 17.9, 12.0, –4.7, –4.9; HRMS (ESI) calcd for $C_{33}H_{63}NNaO_6Si_2^+$ [M + $Na^+$] 648.4086, found 648.4086.

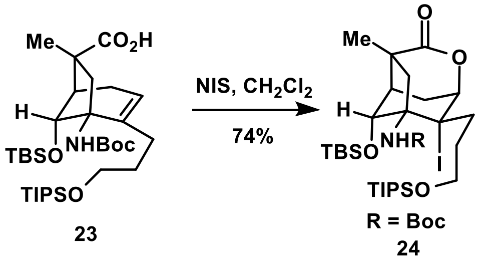


**Iodolactone 24.** Carboxylic acid **23** (0.800 g, 1.28 mmol, 1.0 equiv) was dissolved in $CH_2Cl_2$ (13 mL) at 23 °C and *N*-iodosuccinimide (1.44 g, 6.39 mmol, 5.0 equiv) was then added in a single portion, leading initially to the formation of a white participate and eventually a purple colored solution. The reaction contents were stirred at 23 °C for 6 h. Upon completion, the reaction was quenched by the addition of 3 M $Na_2S_2O_3$ (13 mL). The reaction contents were then poured into a separatory funnel and the resultant layers were separated. The aqueous layer was further extracted with $CH_2Cl_2$ (2 × 15 mL). The combined organic layers were then washed with brine (20 mL), dried ($Na_2SO_4$), filtered, and concentrated. Purification of the resultant residue by flash column chromatography (silica gel, hexanes/EtOAc = 16:1), providing the iodolactone (0.712 g, 74% yield) as a colorless oil. **24**: $R_f$ = 0.58 (silica gel, hexanes/EtOAc,

4:1); IR (film) $\nu_{max}$ 3362, 2941, 2880, 1735, 1700, 1653, 1472, 1455, 1367, 1167, 1110, 1012, 619 cm$^{-1}$; $^{1}$H NMR (500 MHz, $CDCl_3$) δ 4.93 (s, 1 H), 4.86 (d, $J$ = 3.0 Hz, 1 H), 4.46 (s, 1 H), 3.78 (dt, $J$ = 10.9, 5.5 Hz, 1 H), 3.70 (dt, $J$ = 9.9, 6.2 Hz, 1 H), 2.93 (dd, $J$ = 14.2, 2.5 Hz, 1 H), 2.57 (d, $J$ = 14.5 Hz, 1 H), 2.49–2.38 (m, 1 H), 2.04–1.88 (m, 4 H), 1.82–1.70 (m, 2 H), 1.41 (s, 9 H), 1.31 (s, 3 H), 1.03 (d, $J$ = 5.1 Hz, 21 H), 0.95 (s, 9 H), 0.17 (s, 3 H), 0.10 (s, 3 H); $^{13}$C NMR (125 MHz, $CDCl_3$) δ 175.8, 154.1, 80.6, 64.1, 62.6, 60.6, 41.9, 41.5, 35.6, 34.6, 31.5, 29.2, 28.3, 25.9, 25.2, 24.6, 23.7, 22.6, 18.0, 11.9, –4.5, –5.2; HRMS (ESI) calcd for $C_{33}H_{63}INO_6Si_2^+$ [M + H$^+$] 752.3233, found 752.3232.

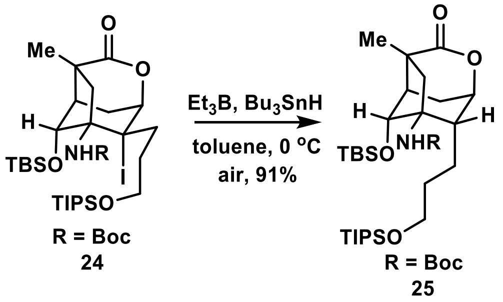


**Lactone 25.** Iodoalcohol **24** (0.270 g, 0.359 mmol, 1.0 equiv) was dissolved in toluene (7.2 mL) at 23 °C and then cooled to 0 °C with an ice-water bath. Next, *n*-$Bu_3SnH$ (0.157 g, 0.538 mmol, 1.5 equiv) and $Et_3B$ (1.0 M in hexane, 0.359 mL, 0.359 mmol, 1.0 equiv) were then added sequentially, and 1.0 mL of air from syringe was subsequently bubbled through the solution to initiate the reaction. The reaction contents were then stirred for 15 min at 0 °C. Upon completion, the reaction contents were quenched by the addition of saturated aqueous $NaHCO_3$ (5 mL). The reaction contents were then poured into a separatory funnel and the resultant layers were separated. The aqueous layer was further extracted with EtOAc (2 × 5 mL). The combined organic layers were then washed with brine (10 mL), dried ($Na_2SO_4$), filtered, and concentrated. Purification of the resultant residue by flash column chromatography (silica gel, hexanes/EtOAc = 8:1) provided the desired lactone (0.201 g, 91% yield) as a colorless oil. **25**: $R_f$ = 0.54 (silica gel, hexanes/EtOAc, 4:1); $^{1}$H NMR (500 MHz, $CDCl_3$) δ 4.79 (s, 1 H), 4.43 (s, 1 H), 4.33–4.22 (m, 1 H), 3.68 (t, $J$ = 6.5 Hz, 2 H), 2.45 (d, $J$ = 14.0 Hz, 1 H), 2.38 (d, $J$ = 13.4 Hz, 1 H), 2.34–2.29 (m, 1 H), 2.07–1.98 (m, 1 H), 1.95 (d, $J$ = 6.2 Hz, 1 H), 1.87–1.77 (m, 1 H), 1.71–1.59 (m, 3 H), 1.42 (s, 9 H), 1.39–1.34 (m, 1 H), 1.31 (s, 3 H), 1.07–1.03 (m, 21 H), 0.92 (s, 9 H), 0.13 (s, 3 H), 0.08 (s, 3 H).

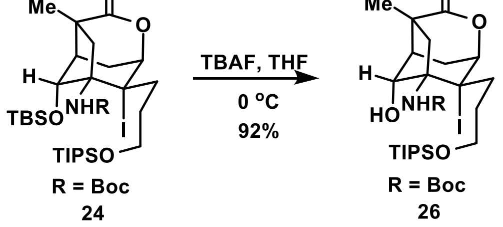


**Iodoalcohol 26.** Iodolactone **24** (0.340 g, 0.452 mmol, 1.0 equiv) was dissolved in THF (4.5 mL) at 23 °C and then was cooled to 0 °C with an ice-water bath. Next, TBAF (1.0 M in

THF, 0.452 mL, 0.452 mmol, 1.0 equiv) was added dropwise. The resultant pale-yellow solution was stirred for an additional 15 min at 0 °C. Upon completion, the reaction contents were quenched by the addition of saturated aqueous $NH_4Cl$ (4 mL). The reaction contents were then poured into a separatory funnel and the resultant layers were separated. The aqueous layer was further extracted with EtOAc (2 × 6 mL). The combined organic layers were then washed with brine (10 mL), dried ($Na_2SO_4$), filtered, and concentrated. Purification of the resultant residue by flash column chromatography (silica gel, hexanes/EtOAc = 8:1) provided the desired iodoalcohol (0.266 g, 92% yield) as a colorless oil. **26**: $R_f$ = 0.34 (silica gel, hexanes/EtOAc, 4:1); IR (film) $\nu_{max}$ 3365, 2942, 2880, 2865, 1734, 1700, 1653, 1472, 1457, 1367, 1166, 1105, 1012, 622 $cm^{-1}$; $^1H$ NMR (500 MHz, $CDCl_3$) δ 5.41 (s, 1 H), 5.20 (s, 1 H), 4.86 (s, 1 H), 4.56 (s, 1 H), 3.82 (dt, *J* = 10.5, 5.1 Hz, 1 H), 3.71 (dt, *J* = 12.5, 6.2 Hz, 1 H), 3.10 (d, *J* = 14.4 Hz, 1 H), 2.47 (d, *J* = 14.0 Hz, 1 H), 2.25 (s, 1 H), 2.07 (q, *J* = 18.3, 16.3 Hz, 2 H), 1.87–1.74 (m, 4 H), 1.43 (s, 9 H), 1.31 (s, 3 H), 1.13–0.97 (m, 21 H); $^{13}C$ NMR (125 MHz, $CDCl_3$) δ 175.7, 154.9, 80.7, 74.6, 65.1, 62.3, 57.8, 45.4, 43.1, 42.0, 35.1, 29.6, 28.8, 28.2, 24.2, 24.1, 18.0, 11.9; HRMS (ESI) calcd for $C_{27}H_{48}INNaO_6Si^+$ [M + $Na^+$] 660.2188, found 660.2184.

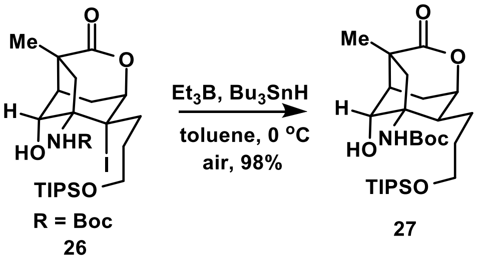


**Lactone 27.** Iodoalcohol **26** (0.260 g, 0.408 mmol, 1.0 equiv) was dissolved in toluene (8.2 mL) at 23 °C and then was cooled to 0 °C with an ice-water bath. Next, *n*-$Bu_3SnH$ (0.178 g, 0.612 mmol, 1.5 equiv) and $Et_3B$ (1.0 M in hexane, 0.41 mL, 0.408 mmol, 1.0 equiv) were then added sequentially and 1.0 mL of air from syringe was subsequently bubbled through the solution to initiate the reaction. The reaction contents were then stirred for 15 min at 0 °C. Upon completion, the reaction contents were quenched by the addition of saturated aqueous $NaHCO_3$ (6 mL). The reaction contents were then poured into a separatory funnel and the resultant layers were separated. The aqueous layer was further extracted with EtOAc (2 × 6 mL). The combined organic layers were then washed with brine (10 mL), dried ($Na_2SO_4$), filtered, and concentrated. Purification of the resultant residue by flash column chromatography (hexanes/EtOAc = 8:1) provided the desired lactone (0.205 g, 98% yield) as a colorless oil. **27**: $R_f$ = 0.32 (silica gel, hexanes/EtOAc, 4:1); IR (film) $\nu_{max}$ 3335, 2942, 2881, 2865, 1734, 1700, 1653, 1473, 1457, 1367, 1168, 1100, 1009 $cm^{-1}$; $^1H$ NMR (500 MHz, $CDCl_3$) δ 6.09 (s, 1 H), 5.10–4.99 (m, 1 H), 4.62 (q, *J* = 2.8 Hz, 1 H), 4.28 (dd, *J* = 6.4, 2.7 Hz, 1 H), 3.71 (qd, *J* = 5.7, 2.5 Hz, 1 H), 3.66–3.55 (m, 1 H), 2.62 (d, *J* = 13.0 Hz, 2 H), 2.17 (q, *J* = 4.5 Hz, 1 H), 2.02 (d, *J* = 13.1 Hz, 1 H), 1.70 (tdd, *J* = 11.6, 6.3, 2.5 Hz, 1 H), 1.65–1.59 (m, 1 H), 1.55–1.46 (m, 3 H), 1.43–1.37 (m, 10 H), 1.27 (s, 3 H), 1.08–0.98 (m, 21 H); $^{13}C$ NMR (125 MHz, $CDCl_3$) δ 177.0, 156.6, 80.9, 76.6, 76.5, 63.7, 63.4, 44.6, 43.03, 42.96, 38.1, 30.3, 28.1, 26.8, 24.3, 21.7, 18.0, 11.8; HRMS (ESI) calcd for $C_{27}H_{49}NNaO_6Si^+$ [M + $Na^+$] 534.3221, found 534.3217.

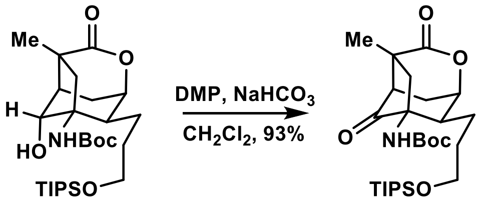


**Ketone 7.** Lactone **27** (0.200 g, 0.391 mmol, 1.0 equiv) was dissolved in $CH_2Cl_2$ (7.8 mL) at 23 °C and then $NaHCO_3$ (0.328 g, 3.91 mmol, 10 equiv) and Dess–Martin periodinane (0.332 g, 0.782 mmol, 2.0 equiv) were added sequentially in single portions. The reaction contents were then stirred for 30 min at 23 °C. Upon completion, the reaction contents were quenched by the addition of saturated aqueous $Na_2S_2O_3$ (6 mL). The reaction contents were then poured into a separatory funnel and the resultant layers were separated. The aqueous layer was further extracted with EtOAc (2 × 6 mL). The combined organic layers were then washed with brine (10 mL), dried ($Na_2SO_4$), filtered, and concentrated. Purification of the resultant residue by flash column chromatography (silica gel, hexanes/EtOAc = 8:1) provided the desired lactone (0.185 g, 93% yield) as a colorless oil. **7**: $R_f$ = 0.32 (silica gel, hexanes/EtOAc, 4:1); IR (film) $\nu_{max}$ 3335, 2942, 2881, 2865, 1734, 1700, 1653, 1473, 1457, 1367, 1168, 1100, 1009 $cm^{-1}$; $^1H$ NMR (500 MHz, $CDCl_3$) δ 5.41 (s, 1 H), 5.20 (s, 1 H), 4.86 (s, 1 H), 4.56 (s, 1 H), 3.82 (dt, $J$ = 10.5, 5.1 Hz, 1 H), 3.71 (dt, $J$ = 12.5, 6.2 Hz, 1 H), 3.10 (d, $J$ = 14.4 Hz, 1 H), 2.47 (d, $J$ = 14.0 Hz, 1 H), 2.25 (s, 1 H), 2.07 (q, $J$ = 18.3, 16.3 Hz, 2 H), 1.87–1.74 (m, 4 H), 1.43 (s, 9 H), 1.31 (s, 3 H), 1.13–0.97 (m, 21 H); $^{13}C$ NMR (125 MHz, $CDCl_3$) δ 175.7, 154.9, 80.7, 74.6, 65.1, 62.3, 57.8, 45.4, 43.1, 42.0, 35.1, 29.6, 28.8, 28.2, 24.2, 24.1, 18.0, 11.9; HRMS (ESI) calcd for $C_{27}H_{47}NNaO_6Si^+$ [M + $Na^+$] 532.3065, found 532.3059.

## Syntheses of Annotinolide D and Annotinolide C

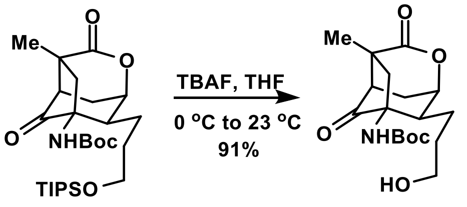


**Alcohol 31.** Ketone **7** (60.1 mg, 0.118 mmol, 1.0 equiv) was dissolved in THF (2.4 mL) at 23 °C and then was cooled to 0 °C with an ice-water bath. Next, TBAF (1.0 M in THF, 0.177 mL, 0.177 mmol, 1.5 equiv) was added dropwise. The resultant pale-yellow solution was stirred for an additional 1 h at 0 °C. Upon completion, the reaction contents were quenched by the addition of saturated aqueous $NH_4Cl$ (2 mL). The reaction contents were then poured into a separatory funnel and the resultant layers were separated. The aqueous layer was further extracted with EtOAc (2 × 3 mL). The combined organic layers were then washed with brine (5 mL), dried ($Na_2SO_4$), filtered, and concentrated. Purification of the resultant residue by flash column chromatography (silica gel, hexanes/acetone = 2:1→1:1) provided the desired alcohol

(37.8 mg, 91% yield) as a colorless oil. **31**: $R_f$ = 0.48 (silica gel, hexanes/acetone, 1:1); IR (film) $\nu_{max}$ 3362, 2973, 2934, 2872, 1734, 1700, 1457, 1387, 1367, 1165, 1131, 1007 cm$^{-1}$; $^1$H NMR (500 MHz, $CDCl_3$) δ 5.27 (s, 1 H), 4.62 (dq, *J* = 4.2, 2.4 Hz, 1 H), 3.68 (dt, *J* = 11.8, 6.0 Hz, 1 H), 3.63 (dt, *J* = 10.9, 6.0 Hz, 1 H), 2.87 (d, *J* = 14.1 Hz, 1 H), 2.51–2.42 (m, 2 H), 2.24 (s, 1 H), 2.15 (td, *J* = 6.3, 3.0 Hz, 1 H), 2.12–1.98 (m, 1 H), 1.92 (d, *J* = 14.0 Hz, 1 H), 1.87–1.65 (m, 2 H), 1.51 (dtd, *J* = 12.3, 6.0, 3.0 Hz, 1 H), 1.41 (s, 9 H), 1.34 (s, 3 H); $^{13}$C NMR (125 MHz, $CDCl_3$) δ 209.2, 174.8, 154.8, 80.1, 73.9, 66.7, 62.3, 52.1, 48.4, 40.8, 39.2, 37.0, 29.9, 28.2, 24.3, 21.5; HRMS (ESI) calcd for $C_{18}H_{27}NNaO_6^+$ [M + Na$^+$] 376.1731, found 376.1724.

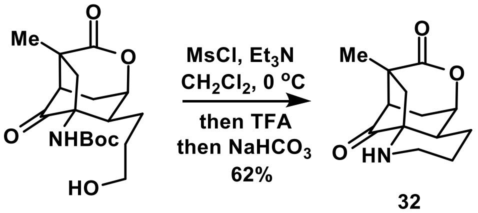


**31** **32**

**Amine 32.** Alcohol **31** (35.2 mg, 0.099 mmol, 1.0 equiv) was dissolved in $CH_2Cl_2$ (2.0 mL) at 23 °C and then the reaction contents were cooled to 0 °C using an ice-water bath. Next, $Et_3N$ (0.138 mL, 0.990 mmol, 10.0 equiv) and MsCl (0.026 mL, 0.297 mmol, 3.0 equiv) were added sequentially, affording a pale-yellow solution. The resultant solution was then stirred for 30 min at 0 °C. TFA (0.5 mL) was then added at 0 °C and the cold bath was removed. The resultant solution was then stirred for 1 h at 23 °C. Upon completion, the reaction contents were quenched by the portion-wise addition of saturated aqueous $NaHCO_3$ until the mixture stopped bubbling. Next, an additional aliquot of saturated aqueous $NaHCO_3$ (3 mL) and $CH_2Cl_2$ (4.0 mL) were then added and the resultant biphasic mixture was stirred vigorously for 30 min at 23 °C. The reaction contents were then poured into a separatory funnel and the resultant layers were separated. The aqueous layer was further extracted with $CH_2Cl_2$ (4 × 5 mL). The combined organic layers were then washed with brine (8 mL), dried ($Na_2SO_4$), filtered, and concentrated. Purification of the resultant residue by flash column chromatography (silica gel, $CH_2Cl_2$/MeOH = 50:1→5:1) provided the desired amine (14.4 mg, 62% yield) as a white amorphous solid. **32**: $R_f$ = 0.13 (silica gel, hexanes/acetone = 1:1); IR (film) $\nu_{max}$ 3333, 2924, 2865, 2844, 1751, 1735, 1457, 1134, 1025 cm$^{-1}$; $^1$H NMR (500 MHz, $CDCl_3$) δ 4.38 (t, *J* = 2.4 Hz, 1 H), 3.04 (d, *J* = 13.2 Hz, 1 H), 2.85 (s, 1 H), 2.63 (t, *J* = 13.0 Hz, 1 H), 2.44–2.38 (m, 2 H), 2.26–2.19 (m, 1 H), 2.06 (dt, *J* = 12.4, 2.7 Hz, 1 H), 1.91 (qd, *J* = 13.0, 3.9 Hz, 1 H), 1.81 (dd, *J* = 13.7, 3.5 Hz, 1 H), 1.74 (d, *J* = 13.6 Hz, 1 H), 1.56 (dd, *J* = 13.4, 1.8 Hz, 1 H), 1.48 (dt, *J* = 13.1, 4.4 Hz, 1 H), 1.34 (d, *J* = 1.3 Hz, 3 H); $^{13}$C NMR (125 MHz, $CDCl_3$) δ 211.7, 175.4, 76.7, 67.1, 50.3, 49.1, 42.1, 40.6, 39.5, 37.9, 26.5, 24.4, 23.0; HRMS (ESI) calcd for $C_{13}H_{17}NNaO_3^+$ [M + Na$^+$] 258.1101, found 258.1100.

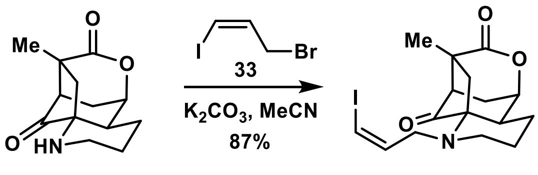


32 S9

**Vinyl iodide S9.** Amine **32** (13.2 mg, 0.0553 mmol, 1.0 equiv) was dissolved in MeCN (0.55 mL) at 23 °C and then $K_2CO_3$ (76.4 mg, 0.553 mmol, 10 equiv) and allylic bromide **33** (68.1 mg, 0.276 mmol, 5.0 equiv)[3] were added sequentially. The resultant white suspension was then stirred for 24 h at 23 °C. Upon completion, the reaction contents were filtered through Celite (eluting with EtOAc). The filtrate was then concentrated and the resultant residue was purified by flash column chromatography (silica gel, hexanes/acetone = 2:1) to provide the desired vinyl iodide (19.4 mg, 87% yield) as a white amorphous solid. **S9**: $R_f$ = 0.70 (silica gel, hexanes/acetone = 1:1); IR (film) $\nu_{max}$ 2932, 2868, 2835, 1748, 1736, 1653, 1457, 1122, 1033, 667 cm$^{-1}$; $^1$H NMR (500 MHz, $CDCl_3$) δ 6.41–6.37 (m, 1 H), 6.36–6.33 (m, 1 H), 4.40 (t, *J* = 2.8 Hz, 1 H), 3.22 (ddq, *J* = 14.2, 4.7, 2.1 Hz, 1 H), 2.92–2.84 (m, 1 H), 2.79 (d, *J* = 12.2 Hz, 1 H), 2.57–2.52 (m, 1 H), 2.40 (ddq, *J* = 14.4, 6.4, 2.2 Hz, 1 H), 2.33 (dt, *J* = 5.7, 2.5 Hz, 1 H), 2.27–2.14 (m, 3 H), 1.88–1.76 (m, 3 H), 1.72 (dt, *J* = 13.5, 2.9 Hz, 1 H), 1.65–1.57 (m, 1 H), 1.38 (s, 3 H); $^{13}$C NMR (125 MHz, $CDCl_3$) δ 213.4, 175.3, 138.9, 83.6, 76.9, 72.7, 55.4, 50.7, 49.0, 48.4, 39.9, 38.8, 31.2, 24.8, 24.7, 23.0; HRMS (ESI) calcd for $C_{16}H_{21}INO_3^+$ [M + $Na^+$] 402.0561, found 402.0570.

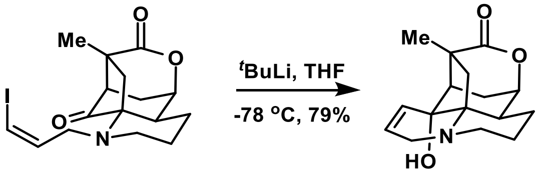


S9 2: annotinolide D

**Annotinolide D (2).** Vinyl iodide **S9** (16.0 mg, 39.9 mmmol, 1.0 equiv) was dissolved in THF (0.79 mL) at 23 °C and then the reaction contents were cooled to –78 °C with a dry ice-acetone bath. Next, *t*-BuLi (1.7 M in pentanes, 0.049 mL, 83.7 mmol, 2.1 equiv) was added dropwise, during which time the solution turned a pale yellow color. The reaction contents were then stirred for 30 min at –78 °C. Upon completion, the reaction contents were quenched by the addition of $H_2O$ (1.5 mL) and warmed to 23 °C with stirring. The reaction contents were then poured into a separatory funnel and the resultant layers were separated. The aqueous layer was further extracted with EtOAc (2 × 3 mL). The combined organic layers were then washed with brine (4 mL), dried ($Na_2SO_4$), filtered, and concentrated. Purification of the resultant residue by flash column chromatography (silica gel, $CH_2Cl_2$/MeOH = 50:1→5:1) provided annotinolide D (**2**, 8.7 mg, 79% yield) as a white amorphous solid. **2**: $R_f$ = 0.24 (silica gel, hexanes/acetone, 1:1); IR (film) $\nu_{max}$ 3245, 2930, 2869, 2837, 1734, 1653, 1473, 1457, 1383, 1288, 1125, 1071, 1017 cm$^{-1}$; $^1$H NMR (500 MHz, $CDCl_3$) δ 5.82 (dt, *J* = 10.1, 1.9 Hz, 1 H), 5.78 (ddd, *J* = 9.9, 3.8, 1.9 Hz, 1 H), 4.36 (p, *J* = 2.7 Hz, 1 H), 3.33 (ddd, *J* = 18.3, 3.5, 1.5 Hz, 1 H), 2.85 (ddd, *J* = 18.3, 2.2, 2.2 Hz, 1 H), 2.71 (br d, *J* = 11.3 Hz, 1 H), 2.67 (ddd, *J* = 14.0, 3.0, 1.3 Hz, 1 H), 2.27 (m, 1 H), 2.24 (dd, *J* = 11.5, 3.1 Hz, 1 H), 2.05 (d, *J* = 13.3 Hz, 1 H), 1.93 (ddd, *J* = 5.0, 1.6, 1.6 Hz, 1 H),

1.75–1.67 (m, 6 H), 1.24 (s, 3 H); $^{13}$C NMR (125 MHz, $CDCl_3$) δ 178.2, 130.6, 127.3, 79.1, 72.7, 65.8, 51.4, 49.8, 46.2, 42.6, 39.6, 31.5, 29.9, 25.0, 24.3, 23.6; HRMS (ESI) calcd for $C_{16}H_{22}NO_3^+$ [M + $H^+$] 276.1594, found 276.1579.

***Table S3.*** $^1$H NMR spectra comparison of natural and synthetic annotinolide D (**2**).

| Natural **2**<br>($^1$H NMR, $CDCl_3$, 400 MHz)<br>δ (*J* in Hz) | Synthetic **2**<br>($^1$H NMR, $CDCl_3$, 500 MHz)<br>δ (*J* in Hz) | Δδ, ppm |
|---|---|---|
| 5.83, ddd (9.8, 2.0, 1.2) | 5.82, dt (10.1, 1.9) | -0.01 |
| 5.79, ddd (9.8, 3.7, 2.0) | 5.78, ddd (9.9, 3.8, 1.9) | -0.01 |
| 4.36, dddd (5.1, 3.1, 2.8, 1.6) | 4.36, p (2.7) | 0.00 |
| 3.34, ddd (18.7, 3.7, 1.2) | 3.33, ddd (18.3, 3.5, 1.5) | -0.01 |
| 2.86, ddd (18.7, 2.0, 2.0) | 2.85, ddd (18.3, 2.2, 2.2) | -0.01 |
| 2.71, br d (11.3) | 2.71, br d (11.3) | 0.00 |
| 2.67, ddd (13.9, 3.1, 1.6) | 2.67, ddd (14.0, 3.0, 1.3) | 0.00 |
| 2.29, br d (13.6) | 2.27, m | -0.02 |
| 2.24, ddd (11.3, 11.3, 1.3) | 2.24, ddd (11.5, 11.5, 3.5) | 0.00 |
| 2.04, d (13.3) | 2.04, d (13.3) | 0.00 |
| 1.92, ddd (5.0, 1.6, 1.6) | 1.91, ddd (5.0, 1.6, 1.6) | -0.01 |
| 1.72 overlapped | 1.75–1.67, m | |
| 1.72 overlapped | | |
| 1.69 overlapped | | |
| 1.68 overlapped | | |
| 1.68 overlapped | | |
| 1.65, dd (13.3, 1.9) | 1.65, dd (13.3, 1.9) | 0.00 |
| 1.25, s | 1.24, s | 0.00 |

***Table S4.*** $^{13}$C NMR spectra comparison of natural and synthetic annotinolide D (**2**).

| Natural **2**<br>($^{13}$C NMR, $CDCl_3$, 100 MHz)<br>δ | Synthetic **2**<br>($^{13}$C NMR, $CDCl_3$, 126 MHz)<br>δ | Δδ, ppm |
|---|---|---|
| 178.2 | 178.2 | 0.0 |
| 130.6 | 130.6 | 0.0 |
| 127.5 | 127.3 | -0.2 |
| 79.1 | 79.0 | -0.1 |

| | | |
|---|---|---|
| 72.7 | 72.7 | 0.0 |
| 65.8 | 65.8 | 0.0 |
| 51.4 | 51.4 | 0.0 |
| 49.9 | 49.8 | -0.1 |
| 46.2 | 46.2 | 0.0 |
| 42.6 | 42.6 | 0.0 |
| 39.7 | 39.7 | 0.0 |
| 31.4 | 31.4 | 0.0 |
| 29.9 | 29.9 | 0.0 |
| 25.1 | 25.0 | -0.1 |
| 24.3 | 24.3 | 0.0 |
| 23.6 | 23.6 | 0.0 |

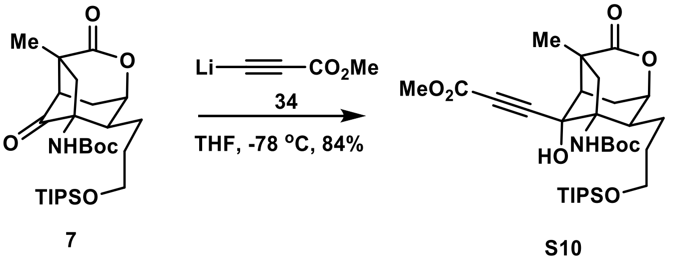


**Alkynyl ester S10.** Methyl propiolate (0.022 mL, 0.250 mmol, 2.5 equiv) was dissolved in THF (0.75 mL) at 23 °C and then the reaction contents were cooled to –78 °C with a dry ice-acetone bath. Next, freshly prepared LDA (1.0 M in THF, 0.250 mL, 0.250 mmol, 2.5 equiv) was added and the resulting orange solution was stirred for an additional 30 min at –78 °C. A solution of ketone **7** (51.1 mg, 0.100 mmol, 1.0 equiv) in THF (0.90 mL) was then added, using another aliquot of THF (0.10 mL) to complete the transfer. The reaction contents were stirred for an additional 15 min at –78 °C. Upon completion, the reaction contents were quenched by the addition of saturated aqueous $NH_4Cl$ (1.5 mL) and warmed to 23 °C. The reaction contents were then poured into a separatory funnel and the resultant layers were separated. The aqueous layer was further extracted with EtOAc (2 × 2 mL). The combined organic layers were then washed with brine (3 mL), dried ($Na_2SO_4$), filtered, and concentrated. Purification of the resultant residue by flash column chromatography (silica gel, hexane/EtOAc= 4:1) to provide the desired alkynyl ester (50.1 mg, 84% yield) as a pale-yellow oil. **S10**: $R_f$ = 0.65 (silica gel, hexanes/EtOAc, 2:1); IR (film) $\nu_{max}$ 3313, 2943, 2880, 2866, 2233, 1750, 1734, 1717, 1473, 1457, 1382, 1254, 1159, 1093 $cm^{-1}$; $^1H$ NMR (500 MHz, $CDCl_3$) δ 7.22 (s, 1 H), 5.02–4.95 (m, 1 H), 4.63 (q, *J* = 2.6 Hz, 1 H), 3.79–3.76 (m, 3 H), 3.74 (t, *J* = 5.3 Hz, 1 H), 3.66–3.61 (m, 1 H), 2.78–2.72 (m, 2 H), 2.45 (dt, *J* = 5.3, 1.7 Hz, 1 H), 2.10 (d, *J* = 13.4 Hz, 1 H), 1.96 (dd, *J* = 13.3, 2.0 Hz, 1 H), 1.77–1.67 (m, 3 H), 1.62–1.51 (m, 2 H), 1.44 (s, 3 H), 1.43 (s, 9 H), 1.06–1.00 (m, 21 H); $^{13}C$ NMR (125 MHz, $CDCl_3$) δ 176.3, 156.9, 153.5, 88.0, 81.9, 77.5, 77.2, 75.9, 67.0,

63.4, 52.8, 50.6, 43.5, 43.1, 38.5, 30.4, 28.3, 28.1, 24.1, 22.1, 18.0, 11.9; HRMS (ESI) calcd for $C_{31}H_{51}NNaO_8Si^+$ [M + $Na^+$] 616.3276, found 616.3273.

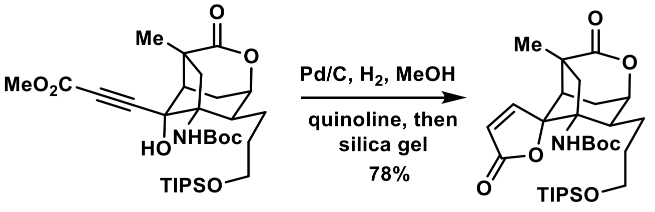


S10 35

**Butenolide 35.** Alkynyl ester **S10** (50.1 mg, 0.842 mmol, 1.0 equiv) and quinoline (0.0030 mL, 0.253 mmol, 3.0 equiv) were sequentially dissolved in MeOH (1.68 mL) at 23 °C. Next, Pd/C (10% wt., 8.9 mg, 0.10 equiv based on Pd) was added, and the resulting black suspension was degassed with a $H_2$ atmosphere. The resultant suspension was then stirred for 1 h at 23 °C in the presence of a $H_2$ atmosphere (from a balloon). Upon completion, the reaction contents were filtered directly through a pad of Celite (eluting with EtOAc). The resultant filtrate was then washed with 3 M HCl (3 mL), poured into a separatory funnel, and the layers were separated. The aqueous layer was then further extracted with EtOAc (3 × 3 mL). The combined organic layers were then washed with brine (4 mL), dried ($Na_2SO_4$), filtered, and concentrated. The resultant crude residue was then dissolved in $CH_2Cl_2$ (0.84 mL) at 23 °C and silica gel (84.2 mg, 1.0 g/mmol substrate) was added. The resultant slurry was stirred for 30 min, before being loaded directly on a silica gel column and purified by flash column chromatography (silica gel, hexane/acetone= 4:1) to provide the desired butenolide (37.2 mg, 78% yield) as a white solid. **35**: $R_f$ = 0.53 (silica gel, hexanes/acetone, 2:1); IR (film) $\nu_{max}$ 3342, 2941, 2878, 2866, 1772, 1748, 1715, 1464, 1457, 1388, 1255, 1245, 1165, 1099 $cm^{-1}$; $^1H$ NMR (500 MHz, $CDCl_3$) δ 7.74 (d, *J* = 5.9 Hz, 1 H), 6.04 (d, *J* = 5.7 Hz, 1 H), 4.65 (s, 1 H), 4.38 (s, 1 H), 3.77 (p, *J* = 5.7, 5.2 Hz, 1 H), 3.67 (td, *J* = 10.4, 8.7, 4.5 Hz, 1 H), 3.12 (d, *J* = 14.7 Hz, 1 H), 2.52 (dd, *J* = 14.4, 3.0 Hz, 1 H), 2.31 (d, *J* = 14.5 Hz, 1 H), 2.16 (t, *J* = 9.2 Hz, 2 H), 1.97 (ddd, *J* = 14.4, 5.2, 2.5 Hz, 1 H), 1.80–1.59 (m, 3 H), 1.56–1.47 (m, 1 H), 1.43 (s, 3 H), 1.33 (s, 9 H), 1.13–0.97 (m, 21 H); $^{13}C$ NMR (125 MHz, $CDCl_3$) δ 175.5, 170.8, 156.2, 154.5, 119.6, 95.0, 80.1, 74.5, 65.9, 62.9, 44.7, 44.2, 42.9, 36.6, 30.4, 30.0, 28.1, 24.5, 21.3, 18.0, 11.9; HRMS (ESI) calcd for $C_{30}H_{49}NNaO_7Si^+$ [M + $Na^+$] 586.3171, found 586.3165.

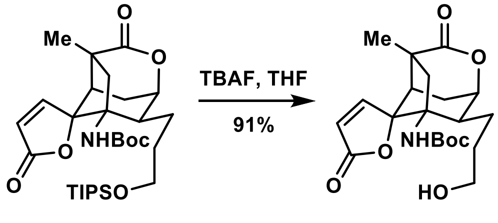


35 S11

**Alcohol S11.** Butenolide **35** (35.5 mg, 0.0621 mmol, 1.0 equiv) was dissolved in THF (1.24 mL) at 23 °C and TBAF (1.0 M in THF, 0.093 mL, 0.0931 mmol, 1.5 equiv) was added dropwise. The resultant light brown solution was stirred for an additional 1 h at 23 °C. Upon completion, the reaction contents were quenched by the addition of saturated aqueous $NH_4Cl$ (2

mL). The reaction contents were then poured into a separatory funnel and the resultant layers were separated. The aqueous layer was further extracted with EtOAc (2 × 3 mL). The combined organic layers were then washed with brine (5 mL), dried ($Na_2SO_4$), filtered, and concentrated. Purification of the resultant residue by flash column chromatography (silica gel, hexanes/acetone = 2:1→1:1), to provide the desired alcohol (23.0 mg, 91% yield) as a white solid. **S11**: $R_f$ = 0.49 (silica gel, hexanes/acetone, 1:1); IR (film) $\nu_{max}$ 3335, 2975, 2936, 2873, 1771, 1744, 1717, 1472, 1457, 1388, 1367, 1270, 1247, 1165, 1096, 1050 $cm^{-1}$; $^1H$ NMR (500 MHz, $CDCl_3$) δ 7.73 (d, *J* = 5.8 Hz, 1 H), 6.06 (d, *J* = 5.7 Hz, 1 H), 4.71 (d, *J* = 7.9 Hz, 1 H), 4.65 (t, *J* = 2.8 Hz, 1 H), 3.73 (dt, *J* = 10.9, 6.9 Hz, 1 H), 3.67 (dt, *J* = 10.8, 6.2 Hz, 1 H), 3.00 (d, *J* = 14.1 Hz, 1 H), 2.53 (dd, *J* = 14.6, 3.0 Hz, 1 H), 2.35 (d, *J* = 14.6 Hz, 1 H), 2.30–2.22 (m, 1 H), 2.18–2.15 (m, 1 H), 1.98 (ddd, *J* = 14.5, 5.1, 2.5 Hz, 1 H), 1.85–1.75 (m, 2 H), 1.72–1.61 (m, 2 H), 1.54 (ddt, *J* = 12.7, 8.8, 6.3 Hz, 1 H), 1.43 (s, 3 H), 1.35 (s, 9 H); $^{13}C$ NMR (125 MHz, $CDCl_3$) δ 175.5, 170.8, 156.2, 154.7, 119.8, 95.1, 80.2, 74.5, 69.5, 65.9, 62.3, 53.7, 43.0, 31.7, 30.5, 29.4, 28.1, 24.5, 21.2; HRMS (ESI) calcd for $C_{21}H_{29}NNaO_7^+$ [M + $Na^+$] 430.1836, found 430.1832.

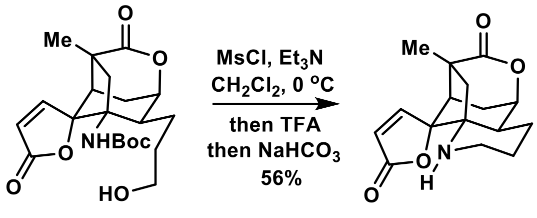

**S11** **1:** annotinolide C

**Annotinolide C (1).** Alcohol **S11** (17.5 mg, 0.0429 mmol, 1.0 equiv) was dissolved in $CH_2Cl_2$ (0.86 mL) at 23 °C and then the reaction contents were cooled to 0 °C using an ice-water bath. Next, $Et_3N$ (0.062 mL, 0.429 mmol, 10 equiv) and MsCl (0.010 mL, 0.129 mmol, 3.0 equiv) were added sequentially, affording a pale-yellow solution. The resultant solution was then stirred for 30 min at 0 °C. TFA (0.22 mL) was then added at 0 °C and the cold bath was removed. The resultant solution was then stirred for 1 h at 23 °C. Upon completion, the reaction contents were quenched by the portion-wise addition of saturated aqueous $NaHCO_3$ until the mixture stopped bubbling. Next, an additional aliquot of saturated aqueous $NaHCO_3$ (3 mL) and $CH_2Cl_2$ (4.0 mL) were then added and the resultant biphasic mixture was stirred vigorously for 30 min at 23 °C. The reaction contents were then poured into a separatory funnel and the resultant layers were separated. The aqueous layer was further extracted with $CH_2Cl_2$ (4 × 5 mL). The combined organic layers were then washed with brine (8 mL), dried ($Na_2SO_4$), filtered, and concentrated. Purification of the resultant residue by flash column chromatography (silica gel, $CH_2Cl_2$/MeOH = 50:1→5:1), provided the desired annotinolide C (**1**, 6.7 mg, 56% yield) as a white amorphous solid. **1**: $R_f$ = 0.22 (silica gel, hexanes/acetone, 1:1); IR (film) $\nu_{max}$ 3306, 2933, 2887, 2860, 1762, 1734, 1653, 1465, 1457, 1248, 1220, 1128, 1097, 678 $cm^{-1}$; $^1H$ NMR (500 MHz, $CDCl_3$) δ 7.60 (d, *J* = 5.8 Hz, 1 H), 6.16 (d, *J* = 5.8 Hz, 1 H), 4.40 (p, *J* = 2.7 Hz, 1 H), 2.90 (ddt, *J* = 13.6, 4.1, 1.8 Hz, 1 H), 2.75 (d, *J* = 14.1 Hz, 1 H), 2.61 (ddd, *J* = 13.8, 3.0, 1.3 Hz, 1 H), 2.55 (br d, *J* = 12.5 Hz, 1 H), 2.25 (br d, *J* = 12.5 Hz, 1 H), 2.20–2.17 (m, 1 H), 1.96 (ddd, *J* = 14.1, 5.1, 2.5 Hz, 1 H), 1.83 (dddd, *J* = 12.9, 12.9, 12.9, 4.2 Hz, 1 H), 1.79–1.75 (m, 1 H), 1.74–1.70 (m, 1 H), 1.68 (dd, *J* = 14.1, 1.8 Hz, 1 H), 1.46–1.40 (m, 1 H), 1.43 (s, 3 H); $^{13}C$ NMR

(125 MHz, $CDCl_3$) δ 176.0, 170.6, 156.0, 121.3, 94.6, 77.9, 65.3, 47.3, 43.2 ,42.9, 42.2, 41.2, 31.5, 27.2, 24.2, 23.6; HRMS (ESI) calcd for $C_{16}H_{20}NO_4^+$ [M + H$^+$] 290.1387, found 290.1389.

***Table S5.*** $^1$H NMR spectra comparison of natural and synthetic annotinolide C (**1**).

| Natural **1** ($^1$H NMR, $CDCl_3$, 400 MHz) δ (*J* in Hz) | Synthetic **1** ($^1$H NMR, $CDCl_3$, 500 MHz) δ (*J* in Hz) | Δδ, ppm |
|---|---|---|
| 7.60, d (5.8) | 7.60, d (5.8) | 0.00 |
| 6.16, d (5.8) | 6.16, d (5.8) | 0.00 |
| 4.40, br ddd (3.0, 2.5, 1.2) | 4.40, p (2.7) | 0.00 |
| 2.90, ddd (13.5, 3.8, 3.8) | 2.90, ddt (13.6, 4.1, 1.8) | 0.00 |
| 2.76, d (14.0) | 2.75, d (14.1) | -0.01 |
| 2.61, ddd (13.0, 3.0, 1.2) | 2.61, ddd (13.8, 3.0, 1.3) | 0.00 |
| 2.56, ddd (13.5, 13.5, 2.8) | 2.56, ddd (13.5, 13.5, 2.9) | 0.00 |
| 2.25, br d (12.7) | 2.25 br d (12.5) | 0.00 |
| 2.19, ddd (5.1, 1.2, 1.2) | 2.19, ddd (5.2, 1.3, 1.3) | 0.00 |
| 1.96, ddd (13.1, 5.1, 2.5) | 1.96, ddd (14.1, 5.1, 2.5) | 0.00 |
| 1.83, dddd (12.7, 12.7, 12.7, 4.0) | 1.83, dddd (12.9, 12.9, 12.9, 4.2) | 0.00 |
| 1.75, m | 1.75, m | 0.00 |
| 1.72, m | 1.73, m | 0.01 |
| 1.68, dd (14.0, 1.9) | 1.68, dd (14.1, 1.8) | 0.00 |
| 1.43, m | 1.43, m | 0.00 |
| 1.43, s | 1.43, s | 0.00 |

***Table S6.*** $^{13}$C NMR spectra comparison of natural and synthetic annotinolide C (**1**).

| Natural **1** ($^{13}$C NMR, $CDCl_3$, 100 MHz) δ | Synthetic **1** ($^{13}$C NMR, $CDCl_3$, 126 MHz) δ | Δδ, ppm |
|---|---|---|
| 176.6 | 176.6 | 0.0 |
| 170.6 | 170.6 | 0.0 |
| 156.0 | 156.0 | 0.0 |
| 121.3 | 121.3 | 0.0 |
| 94.6 | 94.6 | 0.0 |
| 77.9 | 77.9 | 0.0 |
| 65.3 | 65.3 | 0.0 |

| | | |
|---|---|---|
| 47.3 | 47.3 | 0.0 |
| 43.2 | 43.2 | 0.0 |
| 42.9 | 42.9 | 0.0 |
| 42.2 | 42.2 | 0.0 |
| 41.2 | 41.2 | 0.0 |
| 31.5 | 31.5 | 0.0 |
| 27.2 | 27.2 | 0.0 |
| 24.2 | 24.2 | 0.0 |
| 23.6 | 23.6 | 0.0 |

***Figure S2.*** X-ray structure of annotinolide C (**1**).

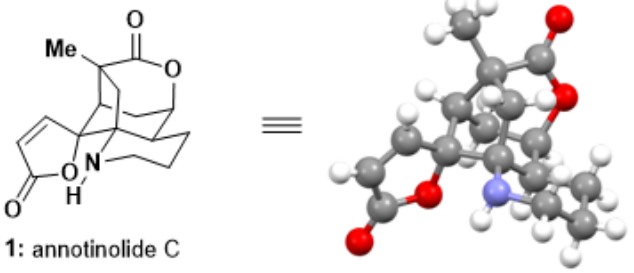


**Transformations between annotinolides C, D and E**

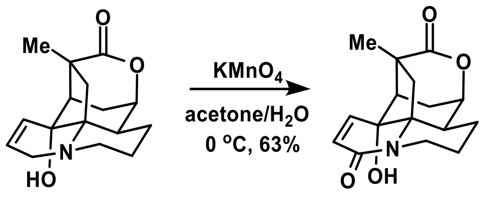


**Annotinolide E (3).** Annotinolide D (**2**, 1.9 mg, 0.0069 mmol, 1.0 equiv) was dissolved in acetone (0.56 mL) and deionized water (0.14 mL) at 23 °C and then the resultant solution was cooled 0 °C using an ice-water bath. $KMnO_4$ (1.6 mg, 0.0104 mmol, 1.5 equiv) was then added in a single portion, leading to a purple-colored solution. The reaction contents were stirred for 15 min at 0 °C. Upon completion, the reaction contents were quenched by the addition of saturated $Na_2S_2O_3$ (0.50 mL) and warmed to 23 °C. The reaction contents were then poured into a separatory funnel and the resultant layers were separated. The aqueous layer was further extracted with EtOAc (3 × 3 mL). The combined organic layers were then washed with brine (4 mL), dried ($Na_2SO_4$), filtered, and concentrated. Purification of the resultant residue by flash column chromatography (silica gel, $CH_2Cl_2$/MeOH = 50:1→5:1) to provide annotinolide E (**3**, 1.2 mg, 63% yield) as a white amorphous solid. **3**: $R_f$ = 0.27 (silica gel, hexanes/acetone, 1:1); IR (film) $\nu_{max}$ 3312, 2931, 2865, 1734, 1653, 1602, 1436, 1383, 1127, 1059, 1021, 988 $cm^{-1}$; $^1H$ NMR (500 MHz, $CDCl_3$) δ 6.66 (d, *J* = 9.7 Hz, 1 H), 6.04 (d, *J* = 9.7 Hz, 1 H), 4.37 (p, *J* = 2.5

Hz, 1 H), 2.76 (ddd, $J$ = 14.2, 3.2, 1.4 Hz, 1 H), 2.67 (ddd, $J$ = 13.5, 13.5, 3.6 Hz, 1 H), 2.56 (br d, $J$ = 12.4 Hz, 1 H), 2.50 (d, $J$ = 13.7 Hz, 1 H), 2.18 (ddd, $J$ = 5.1, 1.6, 1.6 Hz, 1 H), 1.94 (dp, $J$ = 13.3, 3.4 Hz, 1 H), 1.85–1.78 (m, overlapping, 2 H), 1.76 (dddd, $J$ = 13.2, 13.2, 13.2, 3.5 Hz, 1 H), 1.65 (dd, $J$ = 13.7, 1.8 Hz, 1 H), 1.61–1.53 (m, 1 H), 1.25 (s, 3 H); $^{13}C$ NMR (125 MHz, $CDCl_3$) δ 176.9, 163.2, 142.7, 126.4, 78.0, 72.8, 66.1, 48.6, 42.8, 41.5, 39.9, 38.9, 30.3, 23.9, 22.7, 22.6; HRMS (ESI) calcd for $C_{16}H_{20}NO_4^+$ [M + $H^+$] 290.1387, found 290.1393.

***Figure S3.*** X-ray structure of annotinolide E (**3**).

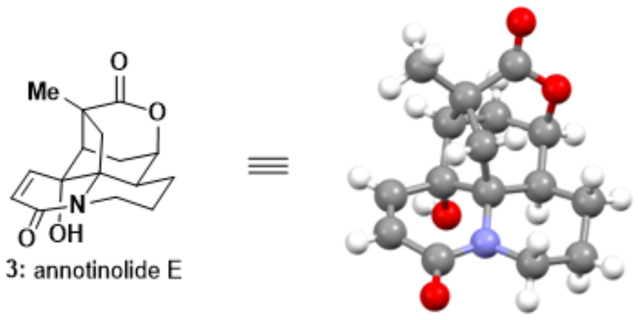


***Table S7.*** $^1$H NMR spectra comparison of natural and synthetic annotinolide E (**3**).

| Natural **3** ($^1$H NMR, $CDCl_3$, 400 MHz) δ ($J$ in Hz) | Synthetic **3** ($^1$H NMR, $CDCl_3$, 500 MHz) δ ($J$ in Hz) | Δδ, ppm |
|---|---|---|
| 6.66, d (9.7) | 6.66, d (9.7) | 0.00 |
| 6.03, d (9.7) | 6.04, d (9.7) | 0.01 |
| 4.47, dddd (5.1, 3.1, 2.8, 1.3) | 4.47, p (2.5) | 0.00 |
| 4.28, br d (13.4) | 4.28, br d (12.7) | 0.00 |
| 2.76, ddd (4.9, 1.3, 1.3) | 2.76, ddd (14.2, 3.2, 1.4) | 0.00 |
| 2.67, ddd (13.4, 13.4, 3.6) | 2.67, ddd (13.5, 13.5, 3.6) | 0.00 |
| 2.55, br d (13.4) | 2.56, br d (12.4) | 0.01 |
| 2.50, d (13.8) | 2.50, d (13.7) | 0.00 |
| 2.18, ddd (4.9, 1.3, 1.3) | 2.18, ddd (5.1, 1.6, 1.6) | 0.00 |
| 1.93, m | 1.94, dp (13.3, 3.4) | 0.01 |
| 1.80, overlapped | 1.81, m, overlapped | 0.01 |
| 1.79, overlapped | 1.79, m, overlapped | 0.00 |
| 1.76, dddd (13.4, 13.4, 13.4, 3.6) | 1.76, dddd (13.2, 13.2, 13.2, 3.5) | 0.00 |
| 1.65, dd (13.8, 1.8) | 1.65, dd (13.7, 1.8) | 0.00 |
| 1.55, m | 1.56, m | 0.01 |
| 1.25, s | 1.25, s | 0.00 |

***Table S8.*** $^{13}$C NMR spectra comparison of natural and synthetic annotinolide E (**3**).

| Natural **3** ($^{13}$C NMR, $CDCl_3$, 100 MHz) δ | Synthetic **3** ($^{13}$C NMR, $CDCl_3$, 126 MHz) δ | Δδ, ppm |
|---|---|---|
| 176.9 | 176.8 | -0.1 |
| 163.2 | 163.2 | 0.0 |
| 142.7 | 142.7 | 0.0 |
| 126.4 | 126.4 | 0.0 |
| 78.0 | 78.0 | 0.0 |
| 72.7 | 72.8 | 0.1 |
| 66.1 | 66.1 | 0.0 |
| 48.6 | 48.6 | 0.0 |
| 42.8 | 12.8 | 0.0 |
| 41.5 | 41.5 | 0.0 |
| 39.9 | 39.9 | 0.0 |
| 38.9 | 38.9 | 0.0 |
| 30.3 | 30.3 | 0.0 |
| 23.9 | 23.9 | 0.0 |
| 22.7 | 22.7 | 0.0 |
| 22.6 | 22.6 | 0.0 |

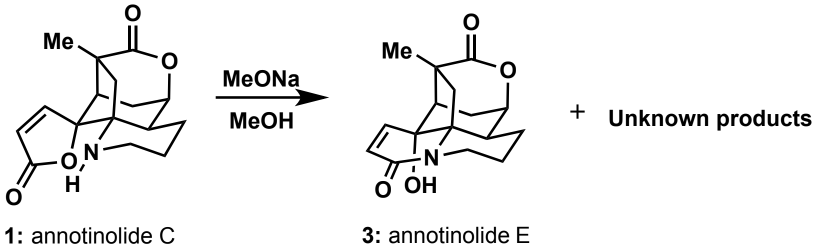


**1:** annotinolide C **3:** annotinolide E

**Annotinolide E (3).** Annotinolide C (**1**, 0.6 mg, 0.0021 mmol, 1.0 equiv) was dissolved in MeOH (0.30 mL) at 23 °C and NaOMe (0.6 mg, 0.01 mmol, 5.0 equiv) was added. The resultant solution was stirred for 1.5 h at 23 °C, at which point TLC analysis indicated no further change. The reaction contents were then diluted with EtOAc (1.0 mL) and quenched by the addition of saturated aqueous $NH_4Cl$ (0.50 mL). The reaction contents were then poured into a separatory funnel and the resultant layers were separated. The aqueous layer was further extracted with EtOAc (3 × 1 mL). The combined organic layers were then washed with brine (2 mL), dried ($Na_2SO_4$), filtered, and concentrated. The resultant residue was then analyzed

directly by $^{1}$H NMR, affording the result shown below indicating a mixture of annotinolide C (**1**), annotinolide E (**3**), and some unknown products in an approximate ratio of 1:1:1.

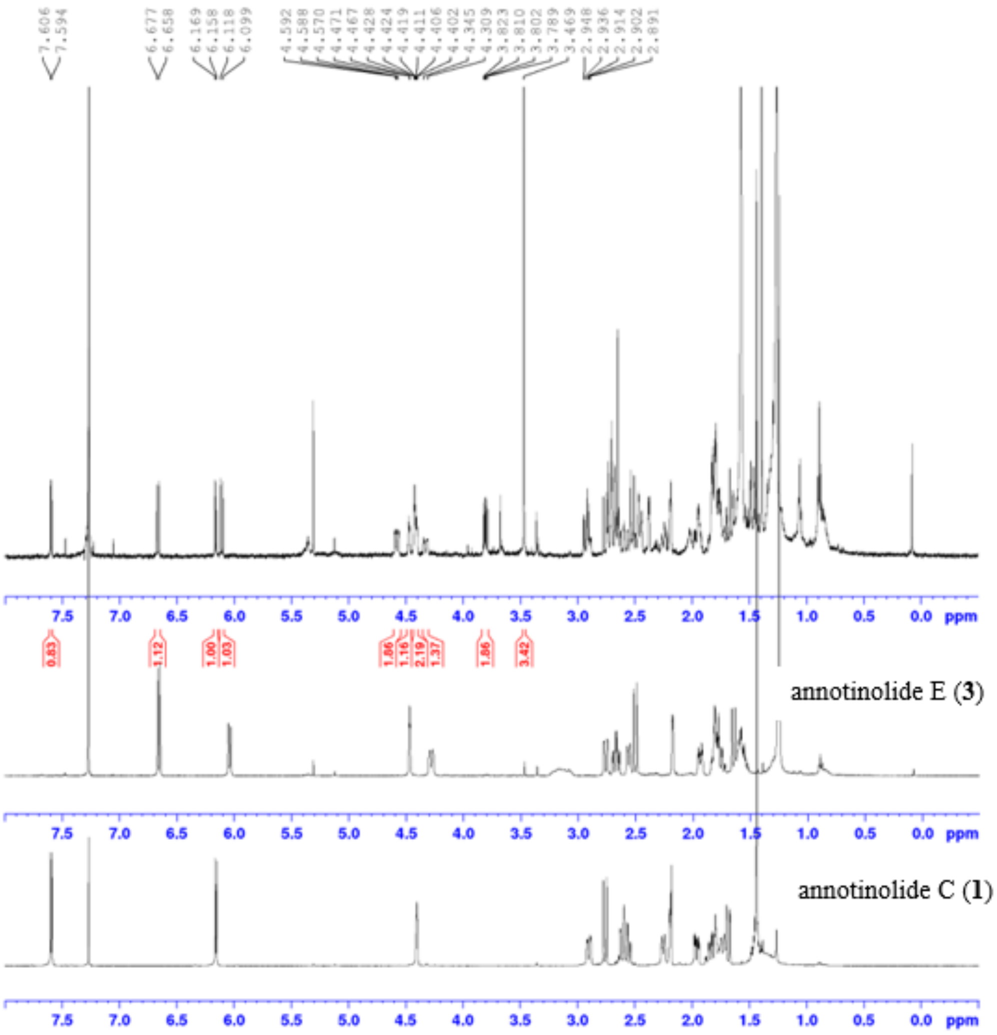


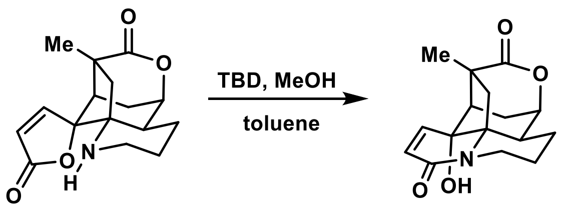


**1:** annotinolide C

**3:** annotinolide E

**Annotinolide E (3).** Annotinolide C (**1**, 0.6 mg, 0.0021 mmol, 1.0 equiv) was dissolved in toluene (0.30 mL) and MeOH (0.030 mL) at 23 °C and triazabicyclo decene (0.3 mg, 0.0021 mmol, 1.0 equiv) was added in one portion. The reaction solution was stirred at 23 °C for 2 h, at which point TLC analysis indicated no further change. The reaction contents were diluted with EtOAc (1.0 mL) and quenched by saturated $NH_4Cl$ solution (0.50 mL). The reaction contents

were then poured into a separatory funnel and the resultant layers were separated. The aqueous layer was further extracted with EtOAc (3 × 1 mL). The combined organic layers were then washed with brine (2 mL), dried ($Na_2SO_4$), filtered, and concentrated. The resultant residue was then analyzed directly by $^1$H NMR, affording the result shown below indicating a mixture of annotinolide C (**1**) and annotinolide E (**3**) in an approximate ratio of 4:1.

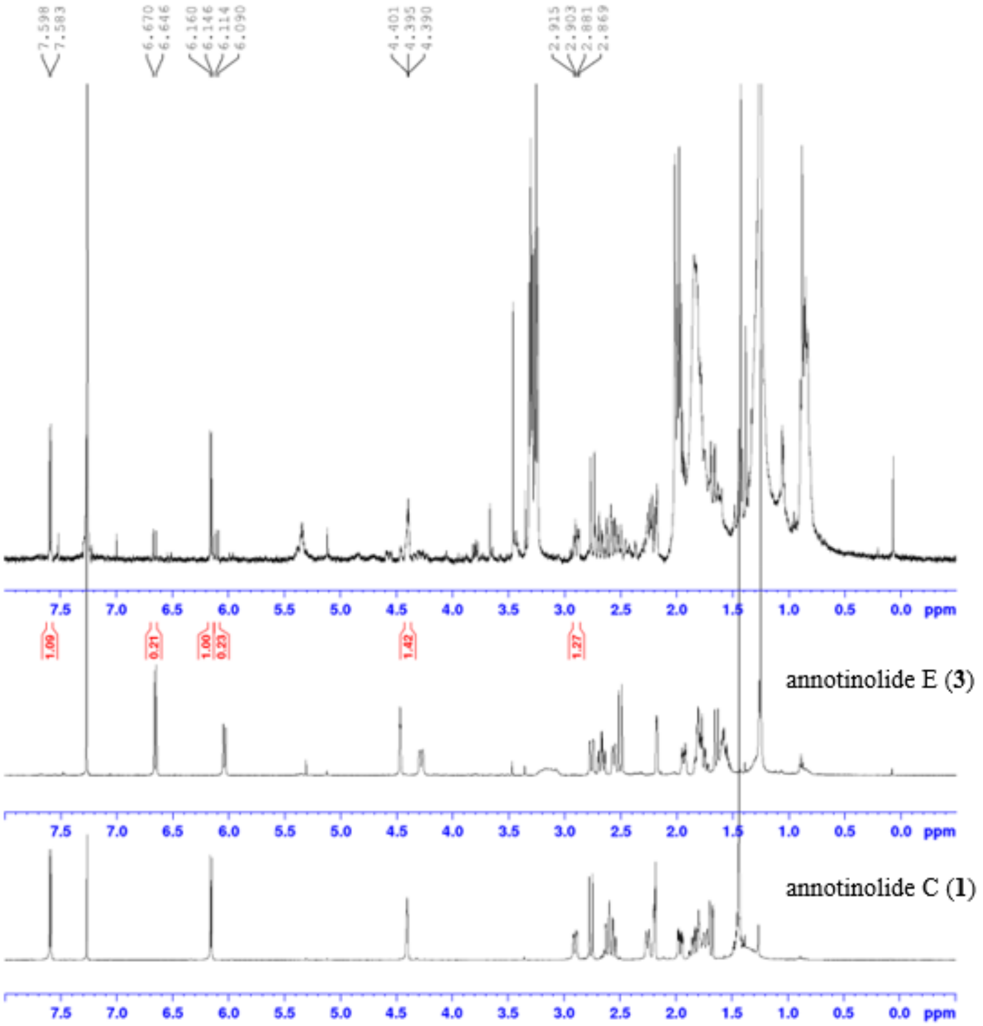

**Asymmetric synthesis of 15:**

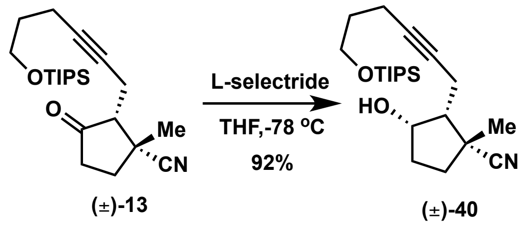


**Alcohol 39.** Ketone **13** (1.90 g, 5.06 mmol, 1.0 equiv) was dissolved in THF (51 mL) and then the reaction solution was cooled to –78 °C with a dry ice-acetone bath. Next, L-selectride (1.0 M in THF, 5.56 mL, 5.56 mmol, 1.1 equiv) was added dropwise, and the resulting pale-yellow solution was stirred at –78 °C for 30 min. Upon completion, the reaction contents were quenched by the addition of saturated aqueous $NH_4Cl$ (40 mL) at –78 °C. After warming the reaction contents to 23 °C, they were poured into a separatory funnel and the resultant layers were separated. The aqueous layer was further extracted with EtOAc (2 × 40 mL). The combined organic layers were then washed with brine (100 mL), dried ($Na_2SO_4$), filtered, and concentrated. Purification of the resultant residue by flash column chromatography (silica gel, hexanes/EtOAc = 6:1→3:1), providing desired alcohol (1.74 g, 92% yield) as a colorless oil. **39**: $R_f$ = 0.26 (silica gel, hexanes/EtOAc = 4:1); IR (film) $\nu_{max}$ 3482, 2943, 2890, 2866, 2234, 1462, 1457, 1248, 1107, 1068 $cm^{-1}$; $^1H$ NMR (500 MHz, $CDCl_3$) δ 4.48–4.40 (m, 1 H), 3.74 (t, $J$ = 6.1 Hz, 2 H), 2.65–2.57 (m, 1 H), 2.51–2.47 (m, 1 H), 2.47–2.40 (m, 1 H), 2.26 (tt, $J$ = 7.1, 2.4 Hz, 2 H), 2.09–2.01 (m, 1 H), 1.91 (d, $J$ = 4.2 Hz, 1 H), 1.86 (dddd, $J$ = 14.5, 8.6, 6.4, 2.3 Hz, 1 H), 1.75 (dt, $J$ = 10.4, 5.3 Hz, 1 H), 1.72–1.64 (m, 3 H), 1.43 (s, 3 H), 1.08–1.01 (m, 21 H); $^{13}C$ NMR (125 MHz, $CDCl_3$) δ 123.8, 84.7, 77.3, 73.9, 61.8, 54.7, 39.6, 38.1 ,32.4, 32.1, 25.45, 18.0, 15.8, 15.1, 11.9; HRMS (ESI) calcd for $C_{22}H_{40}NO_2Si^+$ [M + $H^+$] 378.2823, found 378.2819.

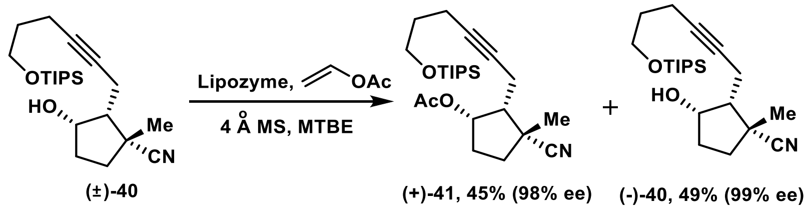


**Acetate 40.** 4 Å molecular sieves (4.05 g, 180 mg/mL solvent) were added to a round bottom flask and subsequently dried by flame heating under vacuum until any chunks in the original sample had disappeared to leave only a residual powder. The flask was then cooled to 23 °C under vacuum and charged with argon. A stir bar and a solution of alcohol **39** (1.65 g, 4.37 mmol, 1.0 equiv) in MTBE (20 mL) were then added at 23 °C. Another portion of MTBE (2.5 mL) was used to rinse the flask to complete the transfer of alcohol **39**. Finally, vinyl acetate (2.08 mL, 22.5 mmol, 5.0 equiv) and Lipozyme (0.450 g, 20 mg/mL solvent) were added and the resulting suspension was stirred at 23 °C for 24 h. Upon completion, the reaction suspension was filtered through a pad of Celite (eluting with EtOAc) and concentrated directly. Purification of the resultant residue by flash column chromatography (silica gel, hexanes/EtOAc = 8:1→4:1) provided desired acetate (0.826 g, 45% yield, 98% ee) as a colorless oil and unreacted alcohol (0.813 g, 49% yield, 99% ee) as a colorless oil. [Note: The ee values of (+)-**41** and (–)-**40** were

measured after they had been transformed separately into benzoate **S12** as delineated below]. **41**: $R_f$ = 0.31 (silica gel, hexanes/EtOAc, 4:1); IR (film) $\nu_{max}$ 2943, 2893, 2866, 2234, 1740, 1464, 1457, 1239, 1107, 1067 $cm^{-1}$; $^1H$ NMR (500 MHz, $CDCl_3$) δ 5.31 (td, *J* = 5.3, 1.9 Hz, 1 H), 3.72 (t, *J* = 6.1 Hz, 2 H), 2.58–2.51 (m, 1 H), 2.47–2.38 (m, 2 H), 2.22 (tt, *J* = 7.2, 2.0 Hz, 2 H), 2.12 (ddd, *J* = 15.2, 9.7, 5.5 Hz, 1H), 2.07 (s, 3 H), 1.94–1.85 (m, 2 H), 1.76–1.70 (m, 1 H), 1.70–1.64 (m, 2 H), 1.52–1.48 (m, 3 H), 1.07–1.01 (m, 21 H); $^{13}C$ NMR (125 MHz, $CDCl_3$) δ 170.3, 123.1, 81.5, 76.7, 76.0, 61.9, 53.1, 40.4, 38.1, 32.1, 30.7, 25.6, 20.9, 18.0, 16.0, 15.1, 11.9; HRMS (ESI) calcd for $C_{24}H_{42}NO_3Si^+$ [M + $H^+$] 420.2928, found 420.2930. $[\alpha]_D^{23}$ = +22° (*c* = 1.0, $CHCl_3$). Alcohol (–)-**40**: $[\alpha]_D^{23}$ = –26° (*c* = 1.0, $CHCl_3$).

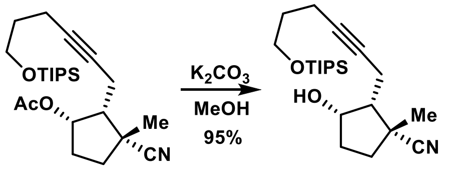


(+)-41 (+)-40

**Alcohol 40.** Acetate **41** (0.770 g, 1.83 mmol, 1.0 equiv) was dissolved in MeOH (92 mL) at 23 °C and then $K_2CO_3$ (2.54 g, 18.3 mmol, 10.0 equiv) was added. The reaction suspension was then vigorously stirred at 23 °C for 1 h. Upon completion, the reaction mixture was filtered through Celite (eluting with EtOAc) and concentrated directly. Purification of the resultant residue by flash column chromatography (silica gel, hexanes/EtOAc = 4:1) providing the desired alcohol (0.658 g, 95% yield) as a colorless oil. (+)-**40**: $[\alpha]_D^{23}$ = +25° (*c* = 1.0, $CHCl_3$)

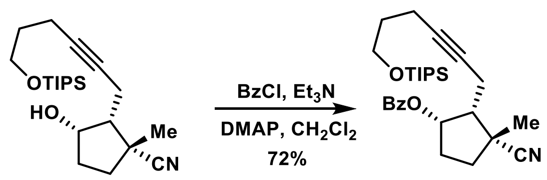


40 S12

**General procedure to prepare benzoate S12 for ee measurement.** Alcohol **40** (10.2 mg, 0.0270 mmol, 1.0 equiv) was dissolved in $CH_2Cl_2$ (0.54 mL) at 23 °C. Then $Et_3N$ (13.7 mg, 0.135 mmol, 5.0 equiv), 4-DMAP (3.3 mg, 0.0270 mmol, 1.0 equiv), and BzCl (5.7 mg, 0.0405 mmol, 1.5 equiv) were added sequentially, forming a yellow solution. The reaction contents were then stirred at 23 °C for 24 h. Upon completion, the reaction solution was quenched by the addition of saturated $NaHCO_3$ solution (0.5 mL) and poured into a separatory funnel. After separating the layers, the aqueous layer was extracted further with $CH_2Cl_2$ (2 × 2 mL). The combined organic layers were then washed with brine (4 mL), dried ($Na_2SO_4$), filtered, and concentrated. Purification of the resultant residue by flash column chromatography (silica gel, hexanes/EtOAc = 10:1) providing the desired benzoate **S12** (9.4 mg, 72% yield) as a colorless oil. IR (film) $\nu_{max}$ 2942, 2894, 2865, 2233, 1722, 1462, 1452, 1272, 1110, 1070, 711 $cm^{-1}$; $^1H$ NMR (500 MHz, $CDCl_3$) δ 8.15–8.11 (m, 2 H), 7.58–7.51 (m, 2 H), 7.48–7.43 (m, 1 H), 5.60 (td, *J* = 5.2, 1.9 Hz, 1 H), 3.68 (t, *J* = 6.0 Hz, 2 H), 2.68 (ddt, *J* = 16.7, 9.1, 2.5 Hz, 1 H), 2.54 (ddt, *J* = 14.8, 6.4, 4.1 Hz, 2 H), 2.28–2.16 (m, 3 H), 2.10–2.00 (m, 2 H), 1.83 (ddd, *J* = 13.7, 10.0, 6.1 Hz, 1 H), 1.64 (dddd, *J* = 13.3, 7.3, 6.1, 2.7 Hz, 2 H), 1.58 (s, 3 H), 1.06–0.99 (m, 21 H); $^{13}C$ NMR

(125 MHz, $CDCl_3$) δ 165.8, 133.1, 129.9, 128.5, 123.5, 81.8, 76.8, 76.7, 61.8, 53.8, 40.5, 38.3, 32.1, 30.8, 25.7, 18.0, 17.9, 16.1, 15.1, 11.9; HRMS (ESI) calcd for $C_{29}H_{43}NNaO_3Si^+$ [M + $Na^+$] 504.2904, found 504.2905. (+)-**S12**: $[\alpha]_D^{23}$ = +45º (*c* = 1.0, $CHCl_3$); (–)-**S12**: $[\alpha]_D^{23}$ = –43º (*c* = 1.0, $CHCl_3$). HPLC condition: OD-H column, 4.6 × 250 mm, hexanes/*i*-PrOH = 99:1, 1 mL/min, UV detector at 240 nm, $R_T$[(+)-**S12**] = 7.14 min, $R_T$[(–)-**S12**] = 8.13 min.

Racemic **S12**:

# Analysis Report

**<Sample Information>**

| | | | |
|---|---|---|---|
| Sample Name | : PQ-1669-7 | | |
| Sample ID | : PQ-1669-7 | | |
| Data Filename | : PQ-1669-7.lcd | | |
| Method Filename | : Default Method.lcm | | |
| Batch Filename | : | | |
| Vial # | : -1 | Sample Type | : Unknown |
| Injection Volume | : 20 uL | | |
| Date Acquired | : 11/16/2020 4:16:53 PM | Acquired by | : Snyder Group |
| Date Processed | : 11/25/2020 6:08:39 PM | Processed by | : Snyder Group |

**<Chromatogram>**

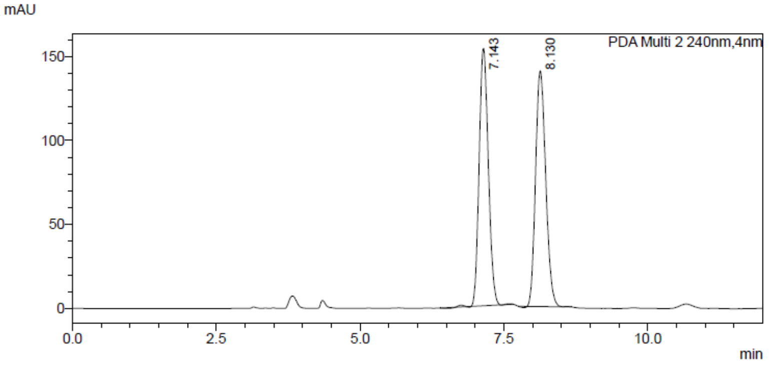


**<Peak Table>**

PDA Ch2 240nm

| Peak# | Ret. Time | Height | Area | Height% | Area% |
|---|---|---|---|---|---|
| 1 | 7.143 | 153147 | 1711250 | 52.174 | 49.808 |
| 2 | 8.130 | 140387 | 1724411 | 47.826 | 50.192 |
| Total | | 293534 | 3435661 | 100.000 | 100.000 |

(+)-**S12** (from resolution product **41**):

# Analysis Report

**<Sample Information>**

Sample Name : PQ-1683
Sample ID : PQ-1683
Data Filename : PQ-1683.lcd
Method Filename : Default Method.lcm
Batch Filename :
Vial # : -1
Injection Volume : 20 uL
Date Acquired : 11/25/2020 5:40:49 PM
Date Processed : 3/17/2021 9:06:02 AM

Sample Type : Unknown
Acquired by : Snyder Group
Processed by : Snyder Group

**<Chromatogram>**

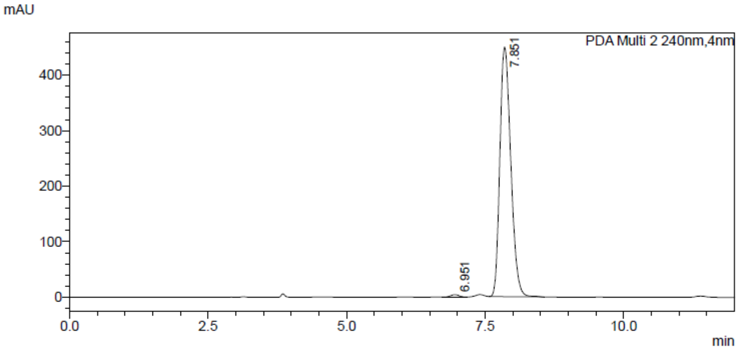


**<Peak Table>**

PDA Ch2 240nm

| Peak# | Ret. Time | Height | Area | Height% | Area% |
|---|---|---|---|---|---|
| 1 | 6.951 | 4635 | 53451 | 1.021 | 0.859 |
| 2 | 7.851 | 449529 | 6168288 | 98.979 | 99.141 |
| Total | | 454164 | 6221739 | 100.000 | 100.000 |

(–)-**S12** (from resolution product **40**):

# Analysis Report

**<Sample Information>**

Sample Name : PQ-1684-2
Sample ID : PQ-1684-2
Data Filename : PQ-1684-2.lcd
Method Filename : Default Method.lcm
Batch Filename :
Vial # : -1
Injection Volume : 20 uL
Date Acquired : 11/26/2020 5:26:05 PM
Date Processed : 3/17/2021 9:03:46 AM

Sample Type : Unknown
Acquired by : Snyder Group
Processed by : Snyder Group

**<Chromatogram>**

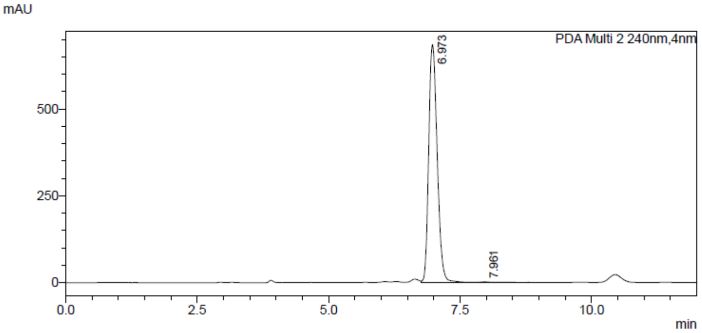


**<Peak Table>**

PDA Ch2 240nm

| Peak# | Ret. Time | Height | Area | Height% | Area% |
|---|---|---|---|---|---|
| 1 | 6.973 | 685158 | 7725821 | 99.807 | 99.804 |
| 2 | 7.961 | 1324 | 15203 | 0.193 | 0.196 |
| Total | | 686482 | 7741024 | 100.000 | 100.000 |

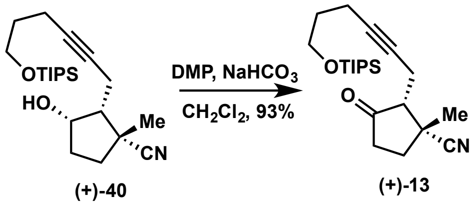


**Ketone 13.** Alcohol **40** (0.650 g, 1.72 mmol, 1.0 equiv) was dissolved in $CH_2Cl_2$ (17 mL) at 23 °C and then $NaHCO_3$ (1.44 g, 17.2 mmol, 10 equiv) and Dess–Martin periodinane (1.45 g, 34.4 mmol, 2.0 equiv) were added sequentially. The resultant suspension was then vigorously stirred at 23 °C for 45 min. Upon completion, the reaction contents were quenched by the addition of 3 M $Na_2S_2O_3$ (15 mL) and poured into a separatory funnel. After separating the layers, the aqueous layer was further extracted with $CH_2Cl_2$ (2 × 15 mL). The combined organic layers were then washed with brine (40 mL), dried ($Na_2SO_4$), filtered, and concentrated. Purification of the resultant residue by flash column chromatography (silica gel, hexanes/EtOAc = 8:1) provided desired ketone (0.604 g, 93% yield) as a colorless oil. (+)-**13**: $[\alpha]_D^{23} = +81°$ ($c$ = 1.0, $CHCl_3$).

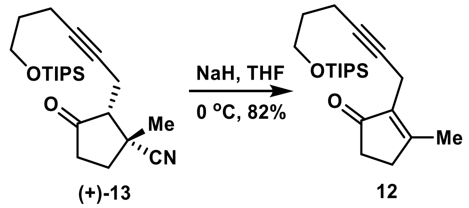


**Enone 12.** Ketone **13** (0.550 g, 1.42 mmol, 1.0 equiv) was dissolved in THF (14 mL) at 23 °C and then the resultant solution was cooled to 0 °C using an ice-water bath. Then NaH (60% dispersion in mineral oil, 0.284 g, 7.10 mmol, 5.0 equiv) was added in a single portion, forming a pale yellow suspension. The reaction mixture was then stirred at 0 °C for 1 h. Upon completion, the reaction contents were quenched by the addition of $H_2O$ (10 mL), warmed to 23 °C, and poured into a separatory funnel. After separating the layers, the aqueous layer was further extracted with EtOAc (2 × 10 mL). The combined organic layers were then washed with brine (30 mL), dried ($Na_2SO_4$), filtered, and concentrated. Purification of the resultant residue by flash column chromatography (silica gel, hexanes/EtOAc = 10:1) provided desired enone (0.405 g, 82% yield) as a pale yellow oil whose spectral data fully matched that of previously characterized material.

*Table S9.* Condition screening for the oxidation of **40**

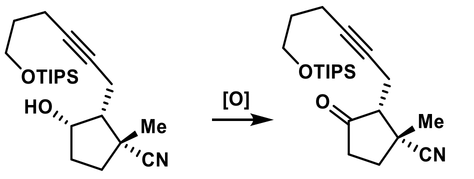


**(-)-40** **(-)-13**

| Entry | Condition | Result[a] |
| --- | --- | --- |
| 1 | PCC (1.5 equiv), $CH_2Cl_2$, 23 °C | 96% ee[b] |
| 2 | DMP (2.0 equiv), $CH_2Cl_2$, 23 °C | 96%, 95% ee[c] |
| 3 | DMP (2.0 equiv), $NaHCO_3$ (10,0 equiv), $CH_2Cl_2$, 23 °C | 88%, 91% ee[d] |
| 4 | $(COCl)_2$ (1.5 equiv), DMSO (5.0 equiv), $Et_3N$ (10.0 equiv) $CH_2Cl_2$, -78 °C to -40 °C | 87% ee[b] |

a: ee value was measured after transforming **13** to benzoate **S12**
b: yield not determined
c: the reaction was perfomed on 0.2 g scale
d: the reaction was perfomed on 0.8 g scale

**Procedure for entry 2.** Alcohol **40** (0.181 g, 0.477 mmol, 1.0 equiv) was dissolved in $CH_2Cl_2$ (4.77 mL) at 23 °C and then Dess–Martin periodinane (0.404 g, 0.953 mmol, 2.0 equiv) was added. The resultant suspension was then vigorously stirred at 23 °C for 30 min. Upon completion, the reaction contents were quenched by the addition of 3 M $Na_2S_2O_3$ (5 mL) and poured into a separatory funnel. After separating the layers, the aqueous layer was further extracted with $CH_2Cl_2$ (2 × 5 mL). The combined organic layers were then washed with brine (10 mL), dried ($Na_2SO_4$), filtered, and concentrated. Purification of the resultant residue by flash column chromatography (silica gel, hexanes/EtOAc = 8:1) provided desired ketone (0.173 g, 96% yield) as a colorless oil. (–)-**13**: $[\alpha]_D^{23} = -84°$ ($c$ = 1.0, $CHCl_3$).

HPLC trace for entry 1:

# Analysis Report

**<Sample Information>**

| | | | |
|---|---|---|---|
| Sample Name | : PQ-1798-2 | | |
| Sample ID | : PQ-1798-2 | | |
| Data Filename | : PQ-1798-2.lcd | | |
| Method Filename | : Default Method.lcm | | |
| Batch Filename | : | | |
| Vial # | : -1 | Sample Type | : Unknown |
| Injection Volume | : 20 uL | | |
| Date Acquired | : 3/18/2021 10:13:40 AM | Acquired by | : Snyder Group |
| Date Processed | : 3/18/2021 10:27:22 AM | Processed by | : Snyder Group |

**<Chromatogram>**

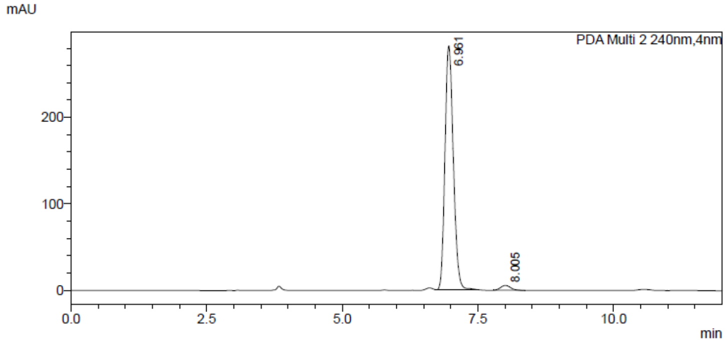


**<Peak Table>**

PDA Ch2 240nm

| Peak# | Ret. Time | Height | Area | Height% | Area% |
|---|---|---|---|---|---|
| 1 | 6.961 | 281849 | 3019970 | 98.052 | 97.716 |
| 2 | 8.005 | 5600 | 70590 | 1.948 | 2.284 |
| Total | | 287449 | 3090559 | 100.000 | 100.000 |

HPLC trace for entry 2:

# Analysis Report

**<Sample Information>**

| | | | |
|---|---|---|---|
| Sample Name | : PQ-1793 | | |
| Sample ID | : PQ-1793 | | |
| Data Filename | : PQ-1793.lcd | | |
| Method Filename | : Default Method.lcm | | |
| Batch Filename | : | | |
| Vial # | : -1 | Sample Type | : Unknown |
| Injection Volume | : 20 uL | | |
| Date Acquired | : 3/6/2021 10:37:55 AM | Acquired by | : Snyder Group |
| Date Processed | : 3/17/2021 9:12:44 AM | Processed by | : Snyder Group |

**<Chromatogram>**

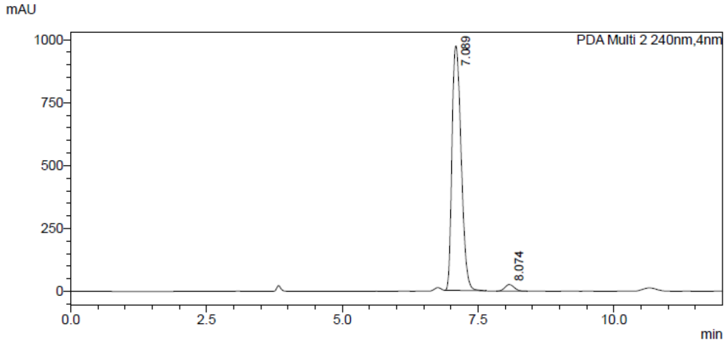


**<Peak Table>**

PDA Ch2 240nm

| Peak# | Ret. Time | Height | Area | Height% | Area% |
|---|---|---|---|---|---|
| 1 | 7.089 | 971303 | 11454802 | 97.390 | 97.369 |
| 2 | 8.074 | 26032 | 309567 | 2.610 | 2.631 |
| Total | | 997335 | 11764369 | 100.000 | 100.000 |

HPLC trace for entry 3:

# Analysis Report

**<Sample Information>**

| | | | |
|---|---|---|---|
| Sample Name | : PQ-1783 | | |
| Sample ID | : PQ-1783 | | |
| Data Filename | : PQ-1783.lcd | | |
| Method Filename | : Default Method.lcm | | |
| Batch Filename | : | | |
| Vial # | : -1 | Sample Type | : Unknown |
| Injection Volume | : 20 uL | | |
| Date Acquired | : 2/28/2021 11:34:56 AM | Acquired by | : Snyder Group |
| Date Processed | : 3/17/2021 9:15:38 AM | Processed by | : Snyder Group |

**<Chromatogram>**

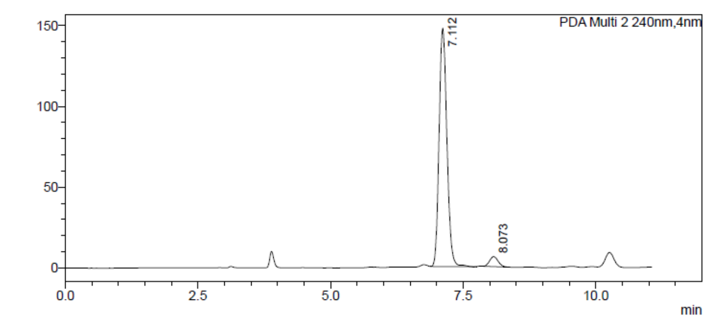


**<Peak Table>**

PDA Ch2 240nm

| Peak# | Ret. Time | Height | Area | Height% | Area% |
|---|---|---|---|---|---|
| 1 | 7.112 | 147484 | 1503542 | 95.988 | 95.603 |
| 2 | 8.073 | 6164 | 69156 | 4.012 | 4.397 |
| Total | | 153647 | 1572698 | 100.000 | 100.000 |

HPLC trace for entry 4:

# Analysis Report

**<Sample Information>**

| | | | |
|---|---|---|---|
| Sample Name | : PQ-1812 | | |
| Sample ID | : PQ-1812 | | |
| Data Filename | : PQ-1812.lcd | | |
| Method Filename | : Default Method.lcm | | |
| Batch Filename | : | | |
| Vial # | : -1 | Sample Type | : Unknown |
| Injection Volume | : 20 uL | | |
| Date Acquired | : 3/17/2021 1:28:59 PM | Acquired by | : Snyder Group |
| Date Processed | : 3/17/2021 1:43:40 PM | Processed by | : Snyder Group |

**<Chromatogram>**

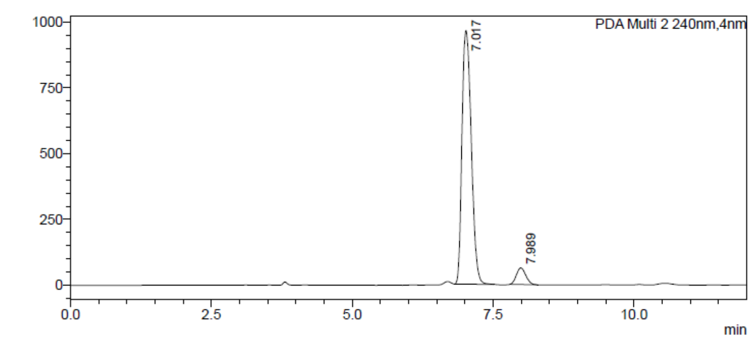


**<Peak Table>**

PDA Ch2 240nm

| Peak# | Ret. Time | Height | Area | Height% | Area% |
|---|---|---|---|---|---|
| 1 | 7.017 | 964041 | 11057125 | 93.857 | 93.795 |
| 2 | 7.989 | 63095 | 731494 | 6.143 | 6.205 |
| Total | | 1027136 | 11788619 | 100.000 | 100.000 |

***Table S10.*** Condition screening acylation of **13**

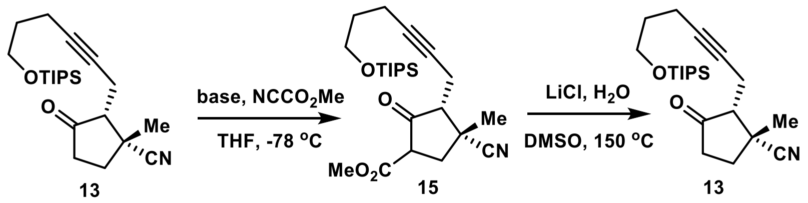


| Entry[a] | Base | Variants | Result[b] |
|---|---|---|---|
| 1 | LDA (1.9 equiv) | none | 72% ee[c] |
| 2 | LDA (1.9 equiv) | pre-mix 13 with $NCCO_2Me$ | unkown product |
| 3 | LDA (1.9 equiv) | $Et_2O$ as solvent | unkown product |
| 4 | LDA (1.9 equiv) | toluene as solvent | unkown product |
| 5 | LiHMDS (1.9 equiv) | none | unkown product |
| 6 | NaHMDS (1.9 equiv) | none | complex mixture |
| 7 | KHMDS (1.9 equiv) | none | 69% ee[c] |
| 8 | LiTMP (1.9 equiv) | none | 76% ee[c] |
| 9[d] | LDA (1.9 equiv) | none | 62%, 79% ee |

a: ketone **13** was 91% ee and the reaction was perfomed on 50 mg scale. The procedures were kept the same as the recamic procdure except the base and variants in this table

b: ee value was measured after transforming **13** to benzoate **S12**

c: yield not determined

d: the reaction was perfomed on 0.2 g scale and keton **13** was 95% ee

**General procedure for preparing ketone 13 from β-ketoester 15.** β-ketoester **15** (16.1 mg, 0.0369 mmol, 1.0 equiv) was dissolved in DMSO (0.19 mL) at 23 °C, and then LiCl (3.1 mg, 0.0738 mmol, 2.0 equiv) and $H_2O$ (3.3 mg, 0.184 mmol, 5.0 equiv) were added subsequently. The resultant reaction solution was then warmed to 150 °C using a pre-heated oil bath and stirred at that temperature for 2 h. Upon completion, the reaction contents were cooled to 23 °C and diluted by the addition of $Et_2O$ (4 mL). The reaction contents were then poured into a separatory funnel, the layers were separated, and the organic layer was washed with 1:1 mixture of brine and water (2 × 2 mL). The organic layer was then dried ($Na_2SO_4$), filtered, and concentrated. Purification of the resultant residue by flash column chromatography (silica gel, hexanes/EtOAc = 8:1) provided desired ketone (6.0 mg, 49% yield) as a colorless oil.

HPLC trace for entry 1:

# Analysis Report

**<Sample Information>**

Sample Name : PQ-1695-2
Sample ID : PQ-1695-2
Data Filename : PQ-1695-3.lcd
Method Filename : Default Method.lcm
Batch Filename :
Vial # : -1
Injection Volume : 20 uL
Date Acquired : 3/17/2021 12:35:35 PM
Date Processed : 3/17/2021 1:03:58 PM

Sample Type : Unknown
Acquired by : Snyder Group
Processed by : Snyder Group

**<Chromatogram>**

mAU

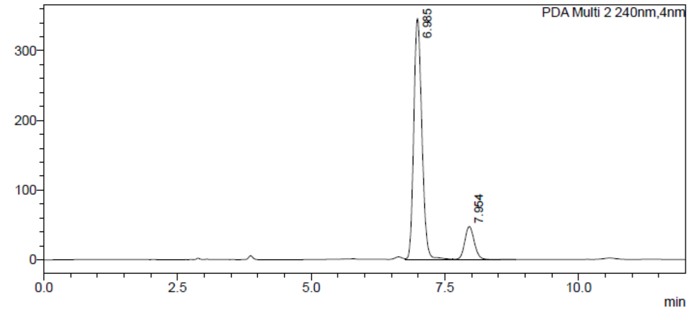


**<Peak Table>**

PDA Ch2 240nm

| Peak# | Ret. Time | Height | Area | Height% | Area% |
|---|---|---|---|---|---|
| 1 | 6.985 | 345925 | 3676198 | 87.924 | 85.961 |
| 2 | 7.954 | 47510 | 600400 | 12.076 | 14.039 |
| Total | | 393435 | 4276597 | 100.000 | 100.000 |

HPLC trace for entry 7:

# Analysis Report

**<Sample Information>**

Sample Name : PQ-1706
Sample ID : PQ-1706
Data Filename : PQ-1706.lcd
Method Filename : Default Method.lcm
Batch Filename :
Vial # : -1
Injection Volume : 20 uL
Date Acquired : 12/10/2020 5:56:06 PM
Date Processed : 3/17/2021 9:00:03 AM

Sample Type : Unknown
Acquired by : Snyder Group
Processed by : Snyder Group

**<Chromatogram>**

mAU

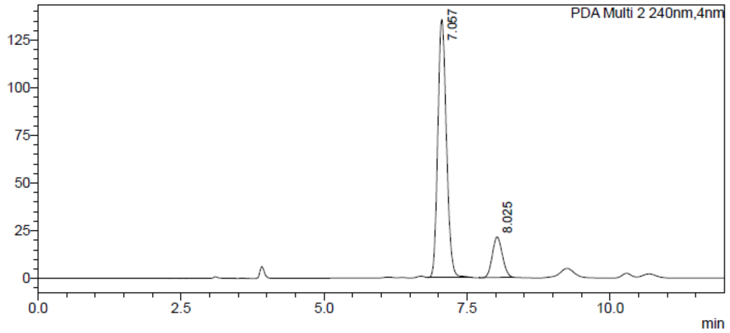


**<Peak Table>**

PDA Ch2 240nm

| Peak# | Ret. Time | Height | Area | Height% | Area% |
|---|---|---|---|---|---|
| 1 | 7.057 | 135380 | 1425525 | 86.440 | 84.523 |
| 2 | 8.025 | 21238 | 261035 | 13.560 | 15.477 |
| Total | | 156618 | 1686560 | 100.000 | 100.000 |

HPLC trace for entry 8:

# Analysis Report

**<Sample Information>**

| | | | |
|---|---|---|---|
| Sample Name | : PQ-1700-2 | | |
| Sample ID | : PQ-1700-2 | | |
| Data Filename | : PQ-1700-2.lcd | | |
| Method Filename | : Default Method.lcm | | |
| Batch Filename | : | | |
| Vial # | : -1 | Sample Type | : Unknown |
| Injection Volume | : 20 uL | | |
| Date Acquired | : 3/18/2021 9:57:45 AM | Acquired by | : Snyder Group |
| Date Processed | : 3/18/2021 10:30:32 AM | Processed by | : Snyder Group |

**<Chromatogram>**

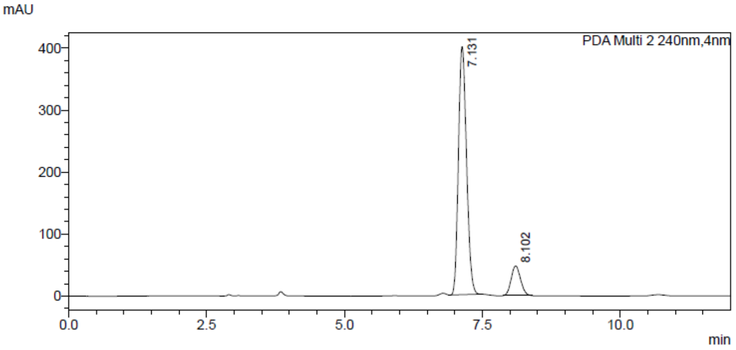


**<Peak Table>**

PDA Ch2 240nm

| Peak# | Ret. Time | Height | Area | Height% | Area% |
|---|---|---|---|---|---|
| 1 | 7.131 | 399781 | 4097706 | 89.327 | 87.850 |
| 2 | 8.102 | 47765 | 566708 | 10.673 | 12.150 |
| Total | | 447546 | 4664414 | 100.000 | 100.000 |

HPLC trace for entry 9:

# Analysis Report

**<Sample Information>**

| | | | |
|---|---|---|---|
| Sample Name | : PQ-1810-1 | | |
| Sample ID | : PQ-1810-1 | | |
| Data Filename | : PQ-1810-1.lcd | | |
| Method Filename | : Default Method.lcm | | |
| Batch Filename | : | | |
| Vial # | : -1 | Sample Type | : Unknown |
| Injection Volume | : 20 uL | | |
| Date Acquired | : 3/17/2021 1:14:31 PM | Acquired by | : Snyder Group |
| Date Processed | : 3/17/2021 1:34:28 PM | Processed by | : Snyder Group |

**<Chromatogram>**

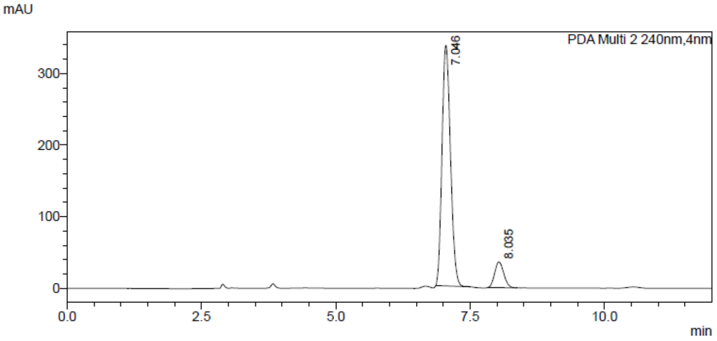


**<Peak Table>**

PDA Ch2 240nm

| Peak# | Ret. Time | Height | Area | Height% | Area% |
|---|---|---|---|---|---|
| 1 | 7.046 | 335603 | 3677124 | 90.316 | 89.666 |
| 2 | 8.035 | 35984 | 423779 | 9.684 | 10.334 |
| Total | | 371586 | 4100903 | 100.000 | 100.000 |

## References


[1] Kippo, T.; Fukuyama, T.; Ryu, I. *Org. Lett.* **2011**, *13*, 3864.

[2] Johnson, C. R.; Adams, J. P.; Braun, M. P.; Senanayake, C. B. W.; Wovkulich, P, M.; Uskoković, M. R. *Tetrahedron Lett.* **1992**, *31*, 917.

[3] Piers, E.; Ranaud, J. *J. Org. Chem.* **1993**, *58*, 11.

# $^{1}$H and $^{13}$C NMR data for selected intermediates

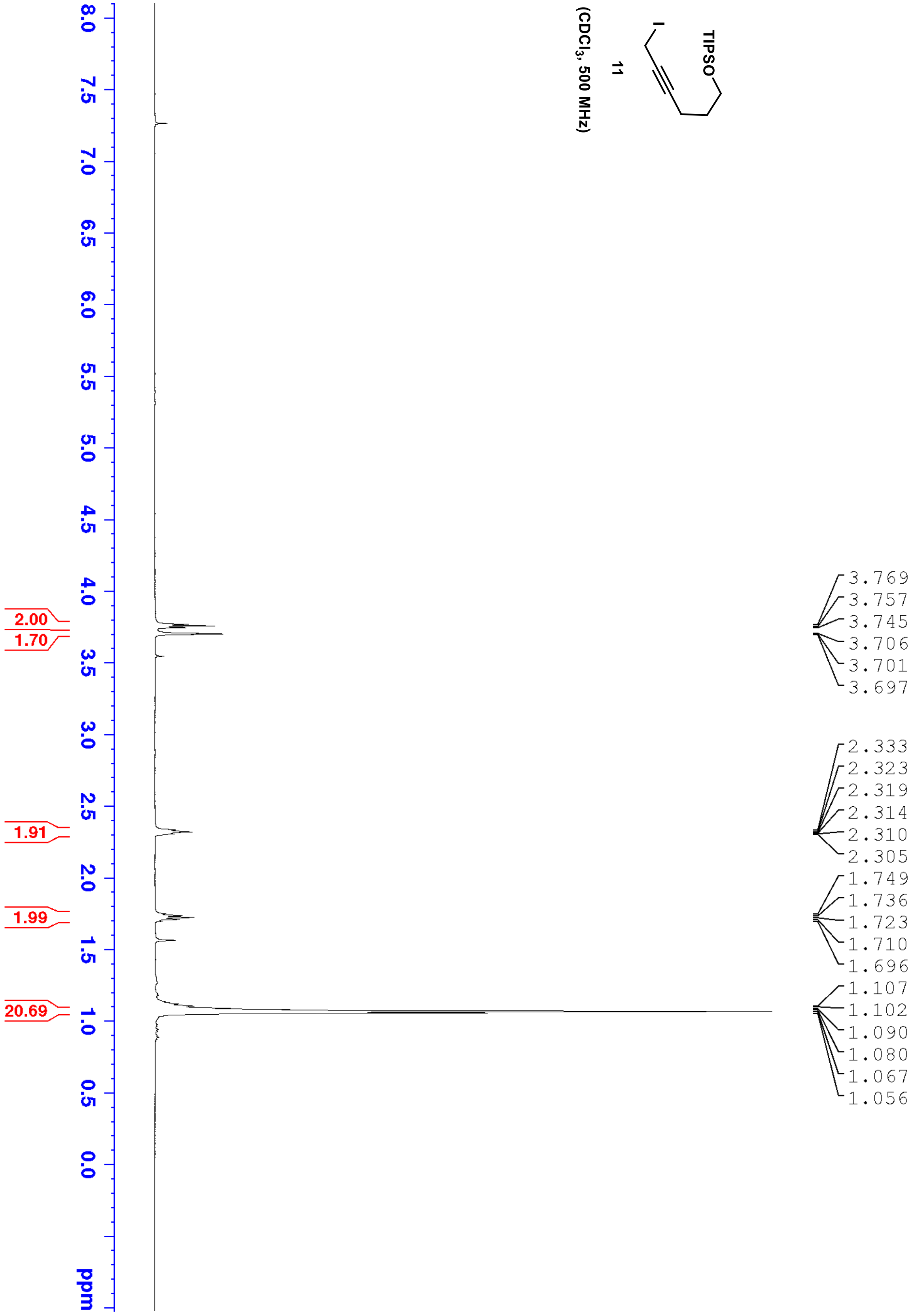

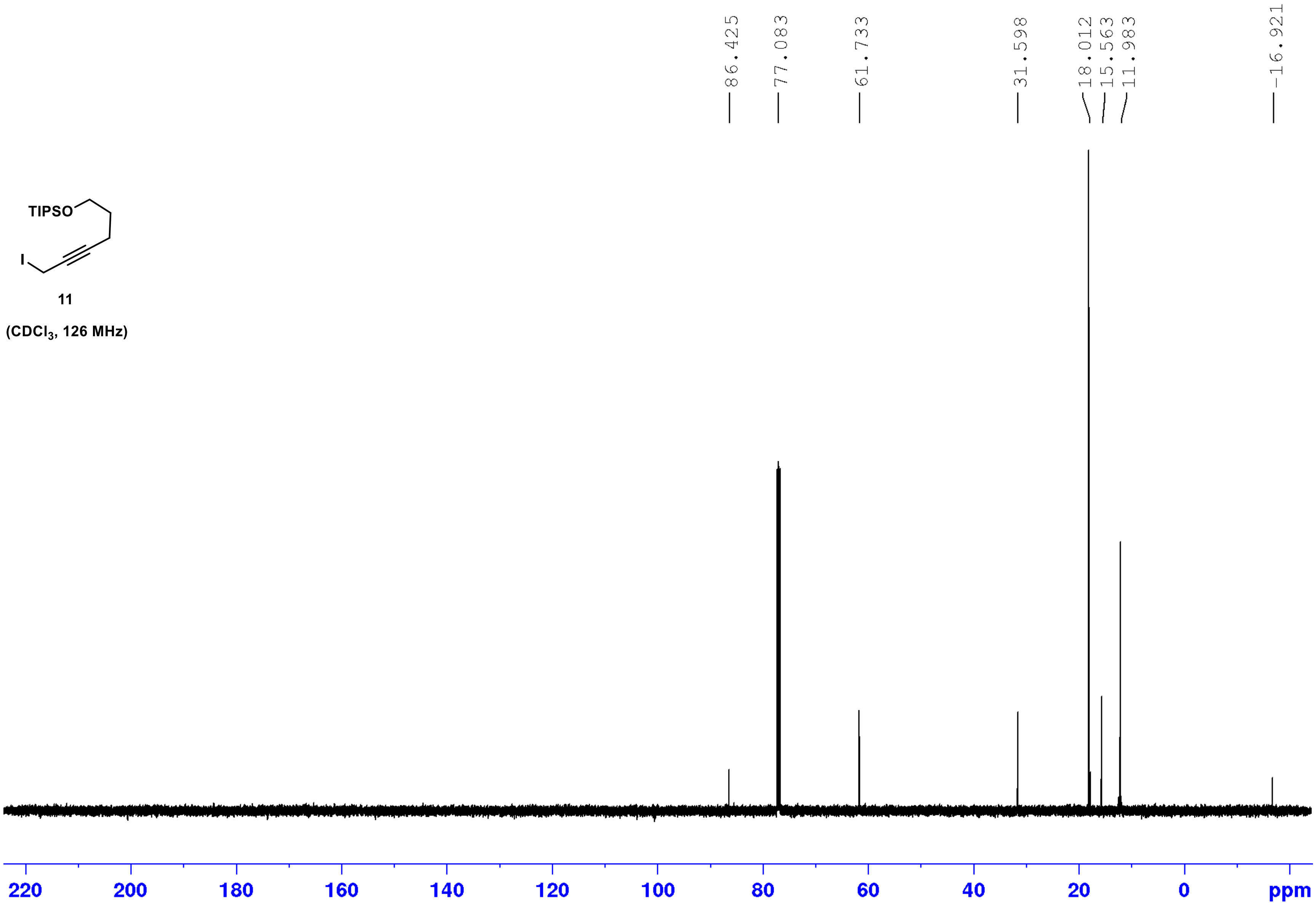
TIPSO
I
11
(CDCl3, 126 MHz)
86.425
77.083
61.733
31.598
18.012
15.563
11.983
-16.921
220
200
180
160
140
120
100
80
60
40
20
0
ppm

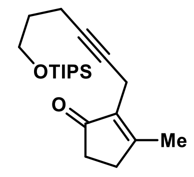


**12**

**($CDCl_3$, 500 MHz)**

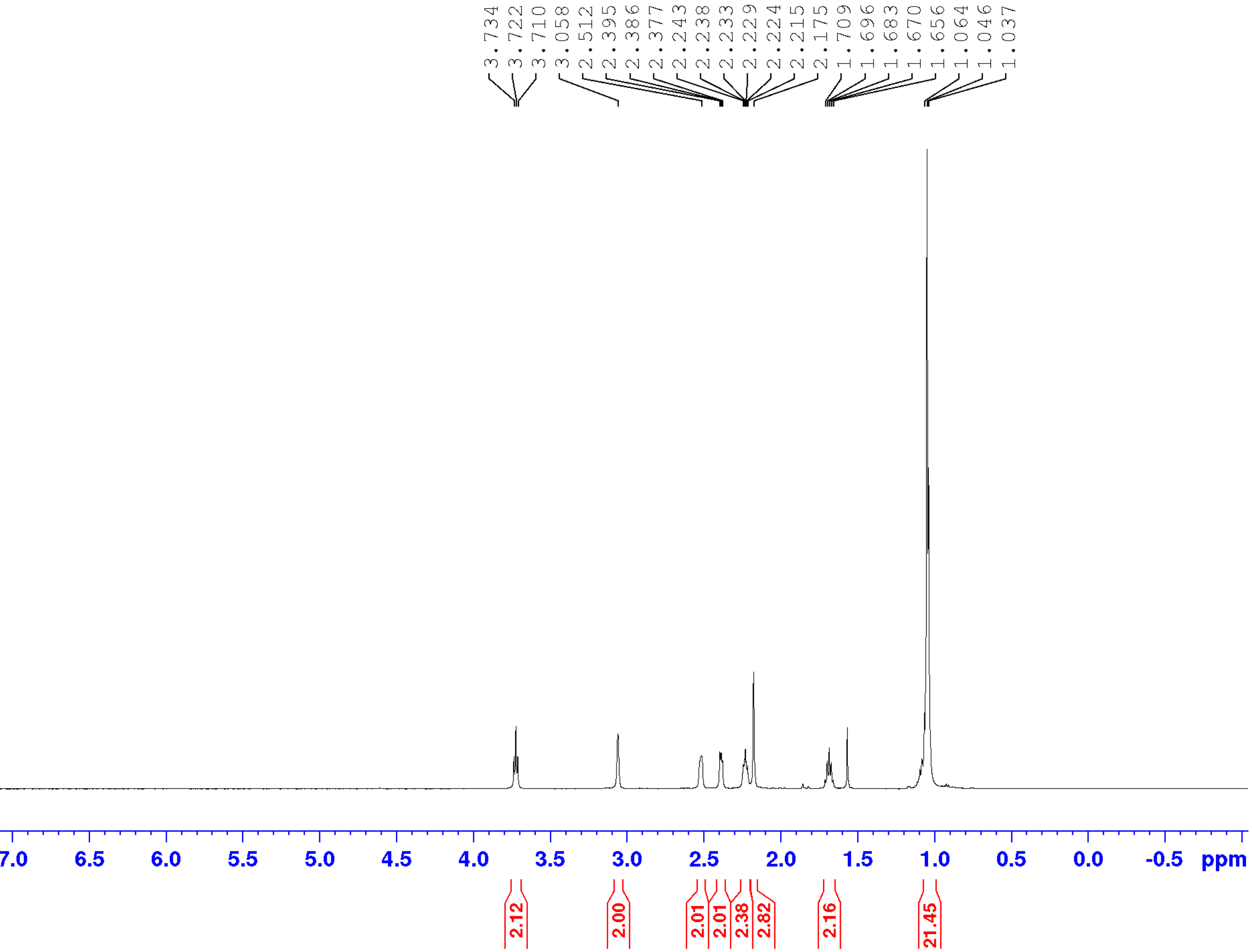

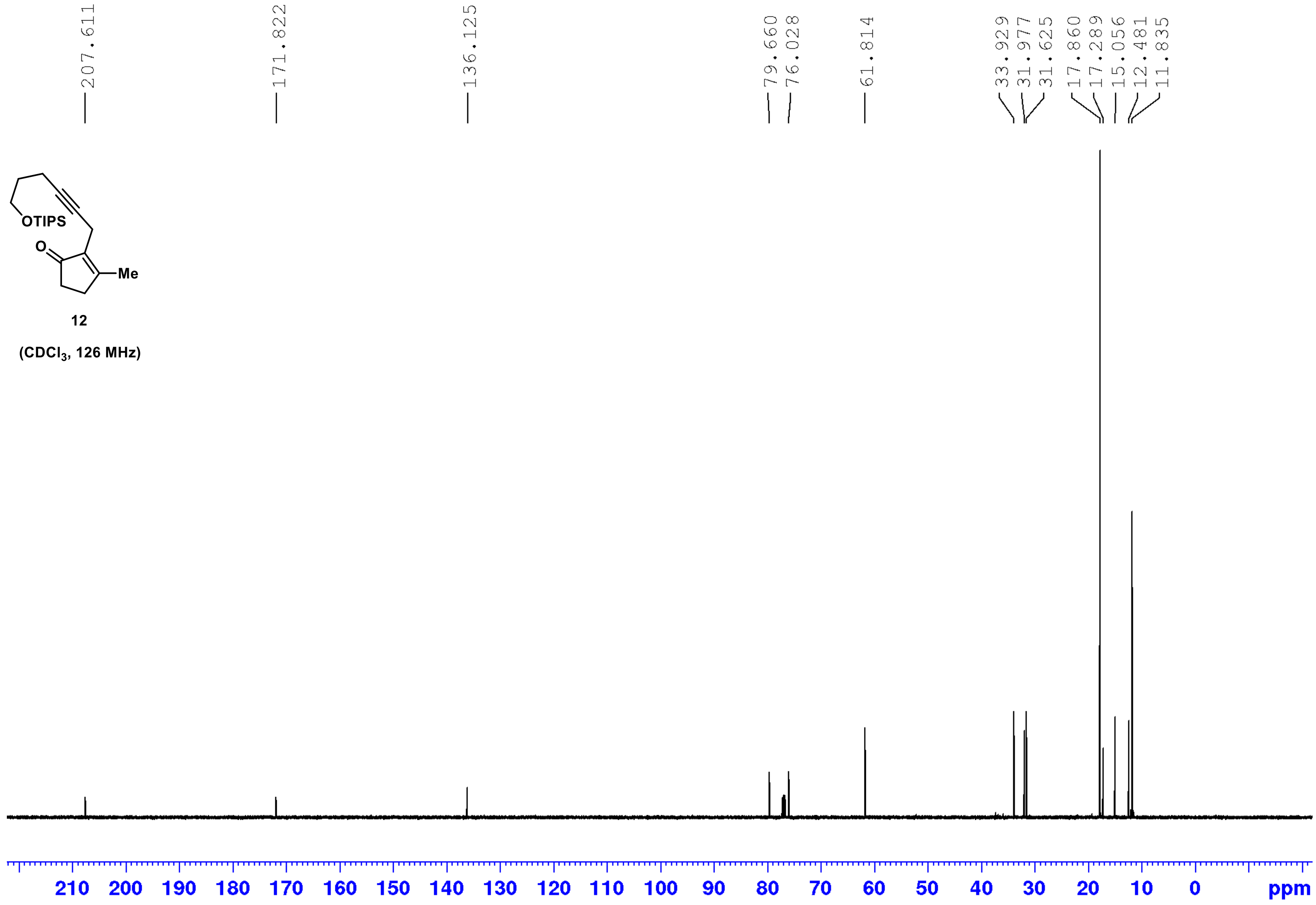
OTIPS
O
Me
12
(CDCl3, 126 MHz)
207.611
171.822
136.125
79.660
76.028
61.814
33.929
31.977
31.625
17.860
17.289
15.056
12.481
11.835
210 200 190 180 170 160 150 140 130 120 110 100 90 80 70 60 50 40 30 20 10 0 ppm

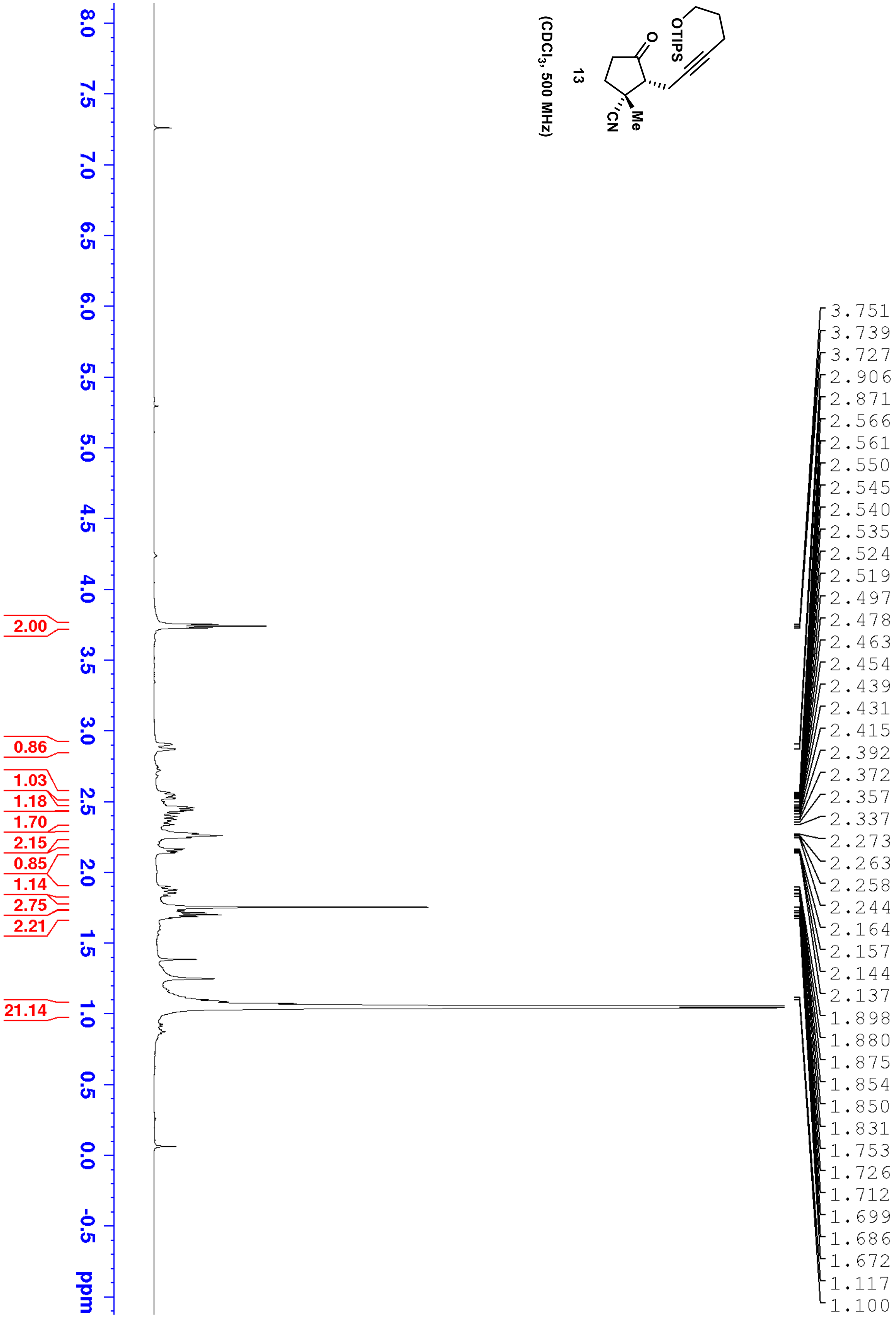

OTIPS
O
Me
CN
13
(CDCl3, 500 MHz)
3.751
3.739
3.727
2.906
2.871
2.566
2.561
2.550
2.545
2.540
2.535
2.524
2.519
2.497
2.478
2.463
2.454
2.439
2.431
2.415
2.392
2.372
2.357
2.337
2.273
2.263
2.258
2.244
2.164
2.157
2.144
2.137
1.898
1.880
1.875
1.854
1.850
1.831
1.753
1.726
1.712
1.699
1.686
1.672
1.117
1.100
2.00
0.86
1.03
1.18
1.70
2.15
0.85
1.14
2.75
2.21
21.14
8.0
7.5
7.0
6.5
6.0
5.5
5.0
4.5
4.0
3.5
3.0
2.5
2.0
1.5
1.0
0.5
0.0
-0.5
ppm

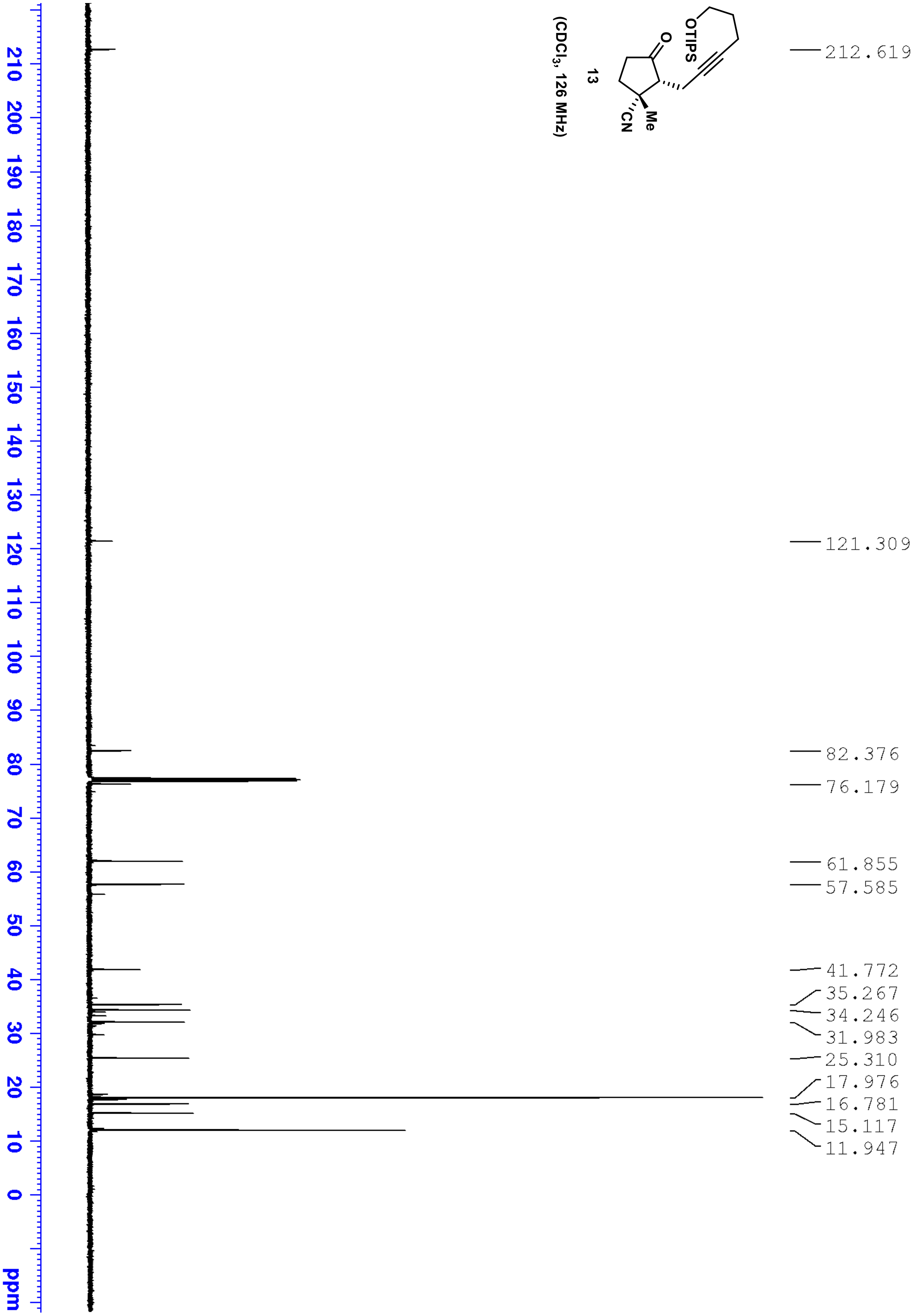

OTIPS
O
Me
CN
13
(CDCl$_3$, 126 MHz)
212.619
121.309
82.376
76.179
61.855
57.585
41.772
35.267
34.246
31.983
25.310
17.976
16.781
15.117
11.947
210 200 190 180 170 160 150 140 130 120 110 100 90 80 70 60 50 40 30 20 10 0
ppm

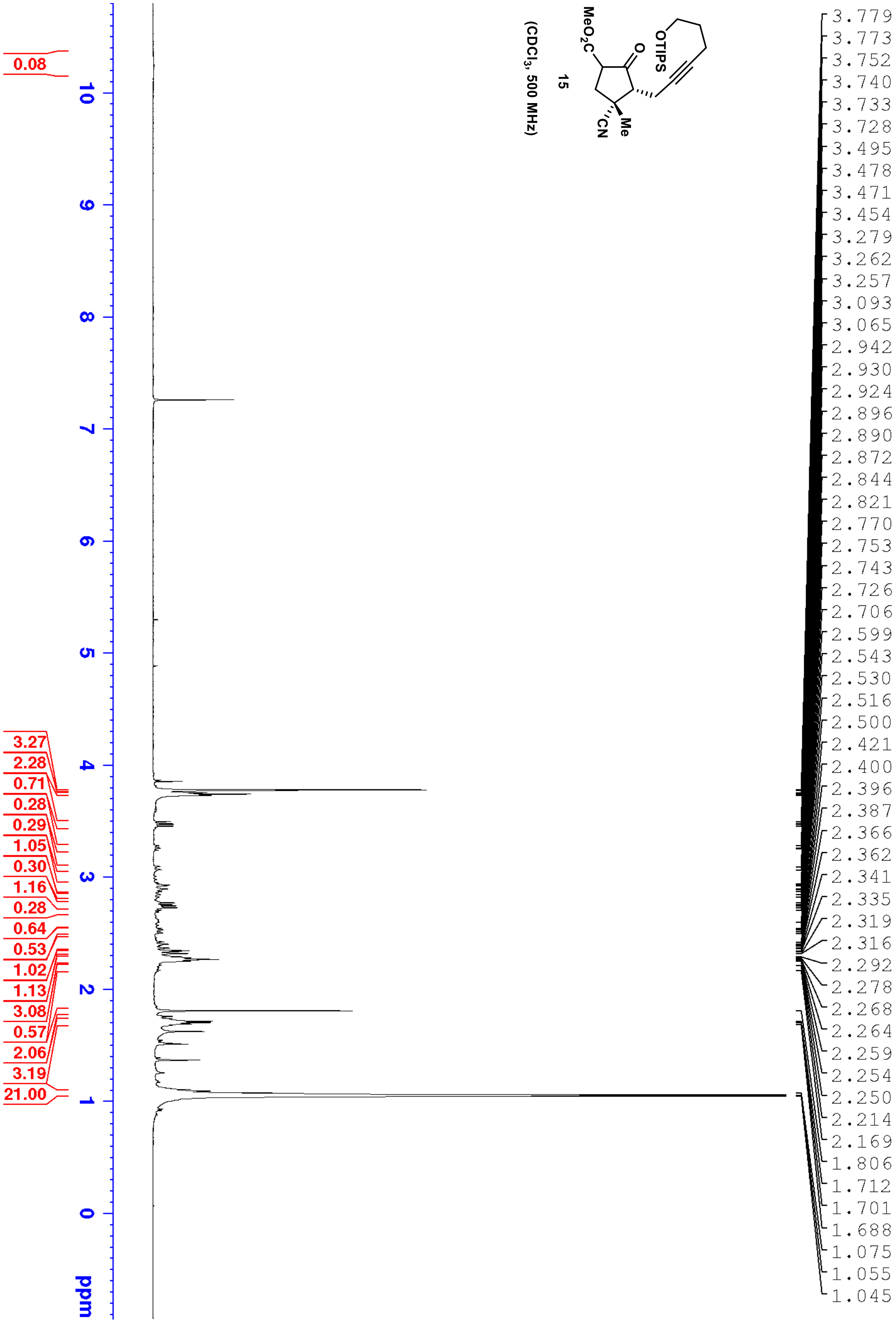
OTIPS
O
MeO2C
Me
CN
15
(CDCl3, 500 MHz)
3.779
3.773
3.752
3.740
3.733
3.728
3.495
3.478
3.471
3.454
3.279
3.262
3.257
3.093
3.065
2.942
2.930
2.924
2.896
2.890
2.872
2.844
2.821
2.770
2.753
2.743
2.726
2.706
2.599
2.543
2.530
2.516
2.500
2.421
2.400
2.396
2.387
2.366
2.362
2.341
2.335
2.319
2.316
2.292
2.278
2.268
2.264
2.259
2.254
2.250
2.214
2.169
1.806
1.712
1.701
1.688
1.075
1.055
1.045
0.08
3.27
2.28
0.71
0.28
0.29
1.05
0.30
1.16
0.28
0.64
0.53
1.02
1.13
3.08
0.57
2.06
3.19
21.00
10
9
8
7
6
5
4
3
2
1
0
ppm

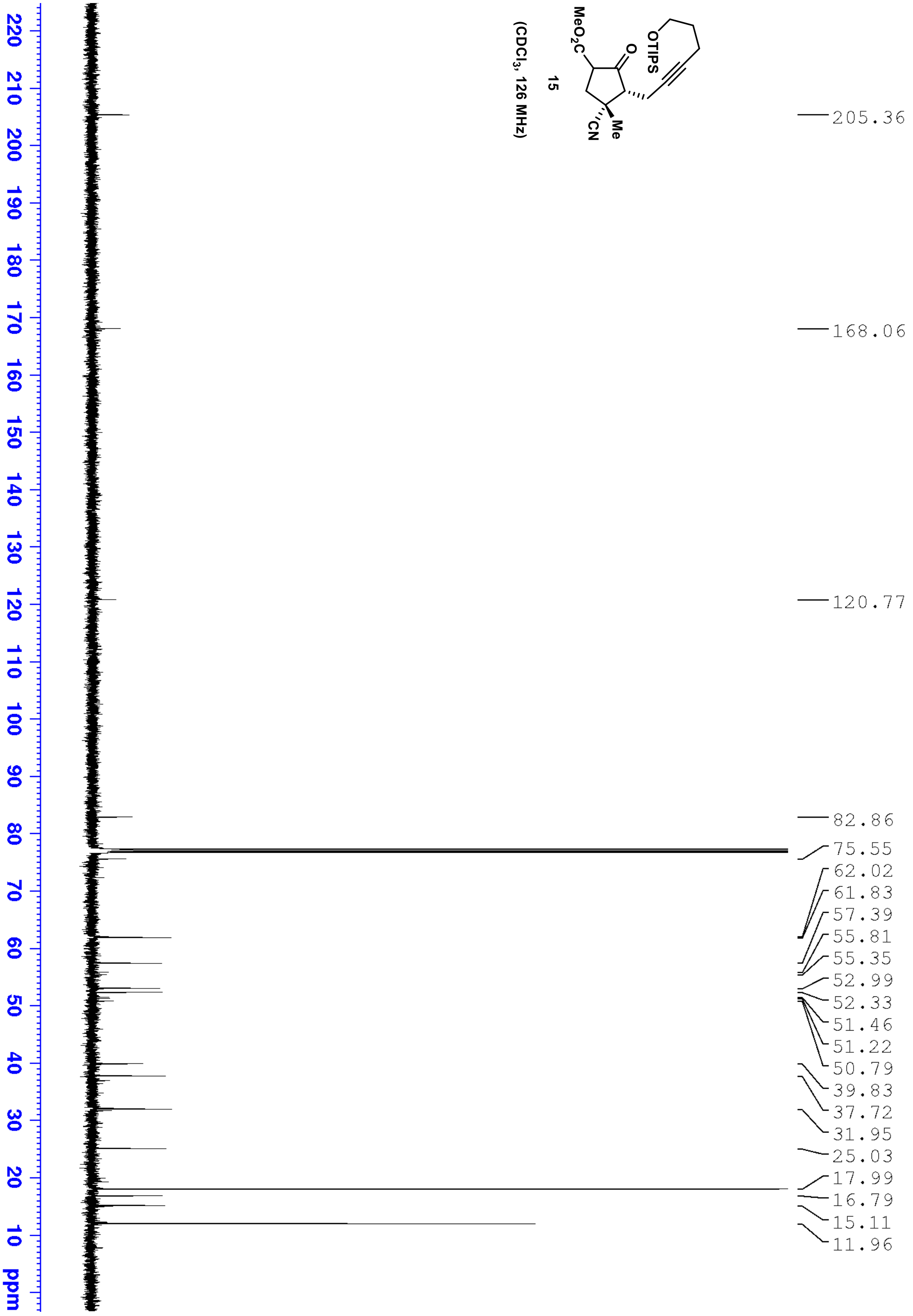

OTIPS
O
MeO2C
Me
CN
15
(CDCl3, 126 MHz)
205.36
168.06
120.77
82.86
75.55
62.02
61.83
57.39
55.81
55.35
52.99
52.33
51.46
51.22
50.79
39.83
37.72
31.95
25.03
17.99
16.79
15.11
11.96
220 210 200 190 180 170 160 150 140 130 120 110 100 90 80 70 60 50 40 30 20 10 ppm

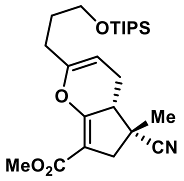


16

($CDCl_3$, 500 MHz)

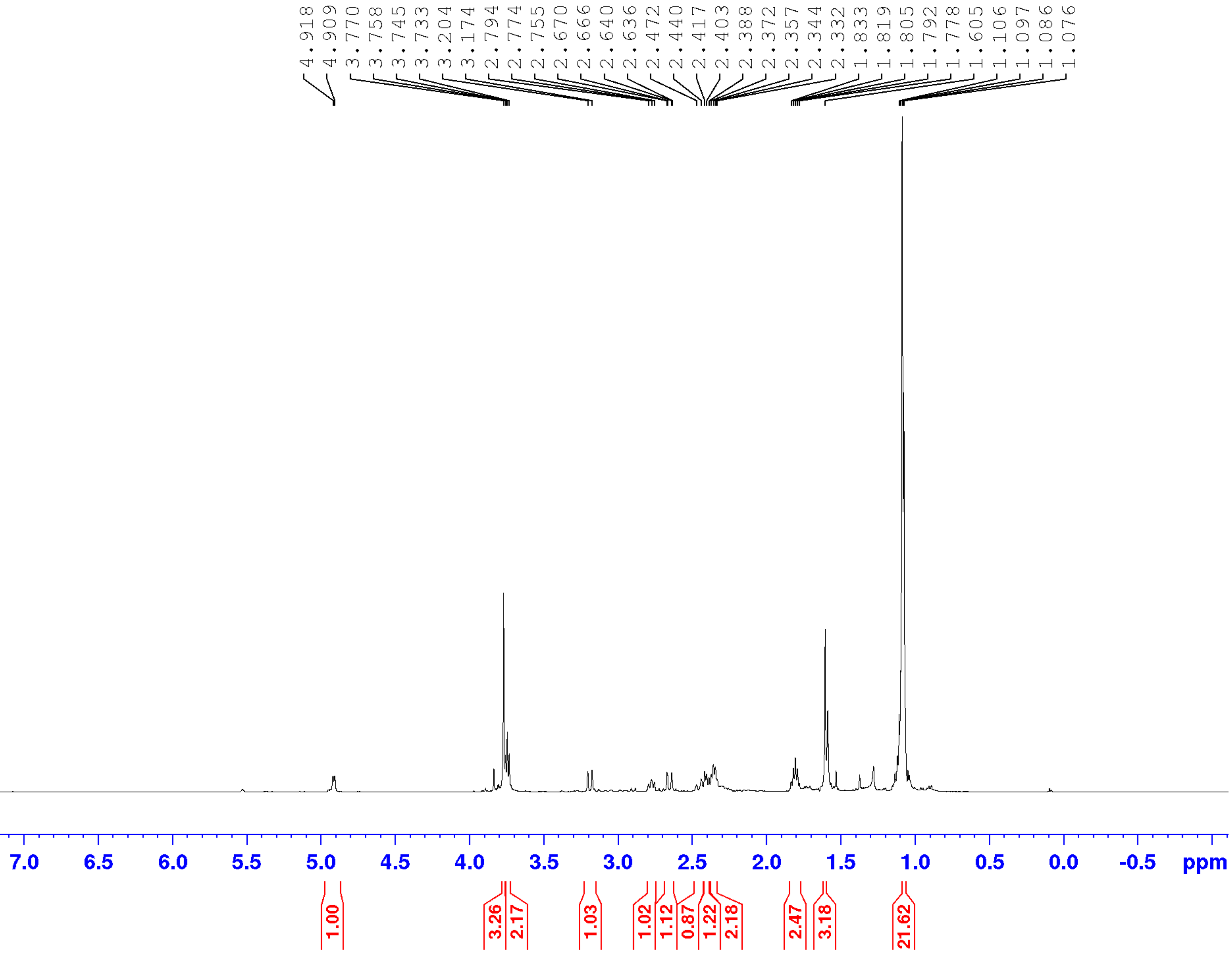

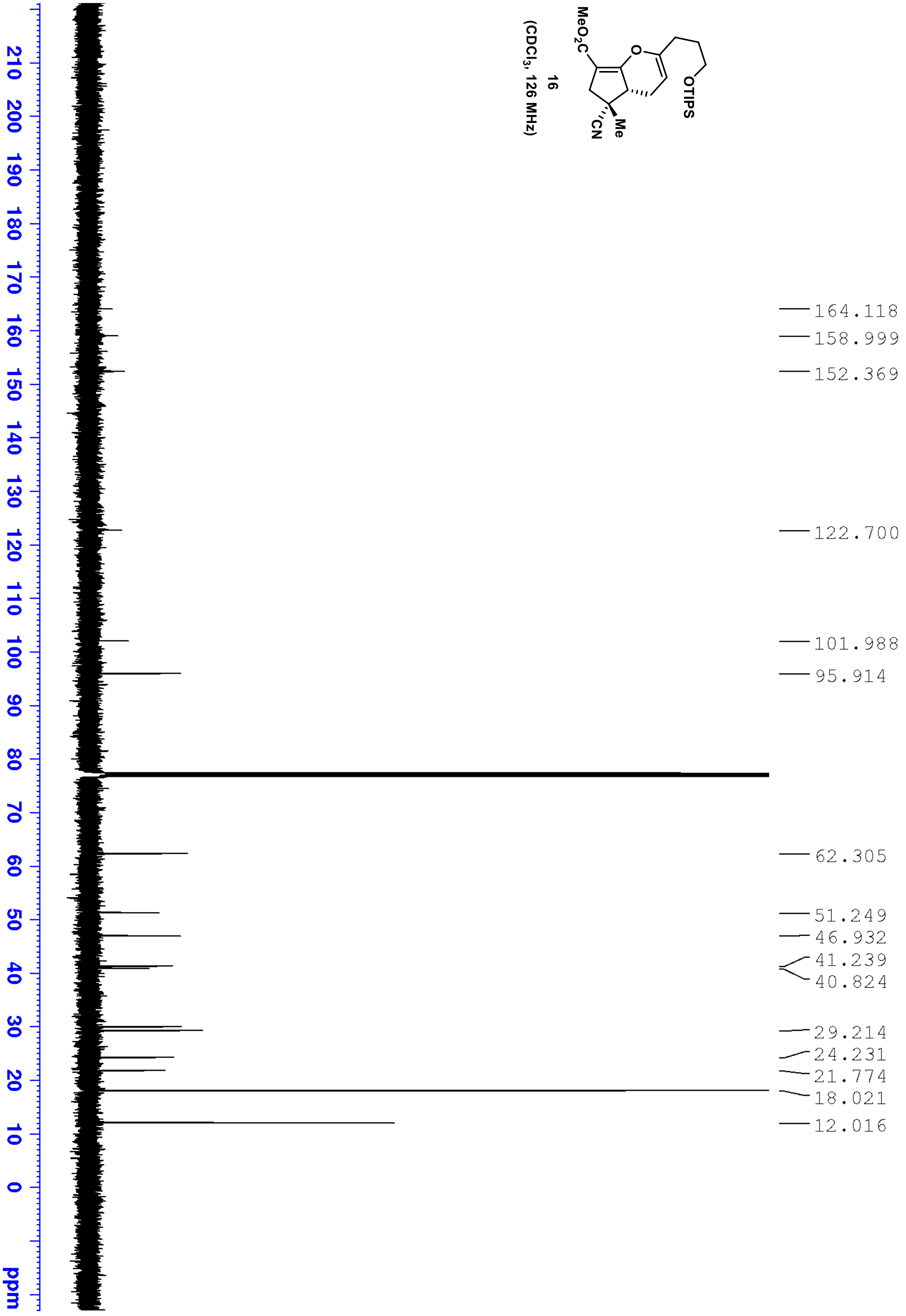

OTIPS
Me
CN
MeO2C
16
(CDCl3, 126 MHz)
164.118
158.999
152.369
122.700
101.988
95.914
62.305
51.249
46.932
41.239
40.824
29.214
24.231
21.774
18.021
12.016
210 200 190 180 170 160 150 140 130 120 110 100 90 80 70 60 50 40 30 20 10 0
ppm

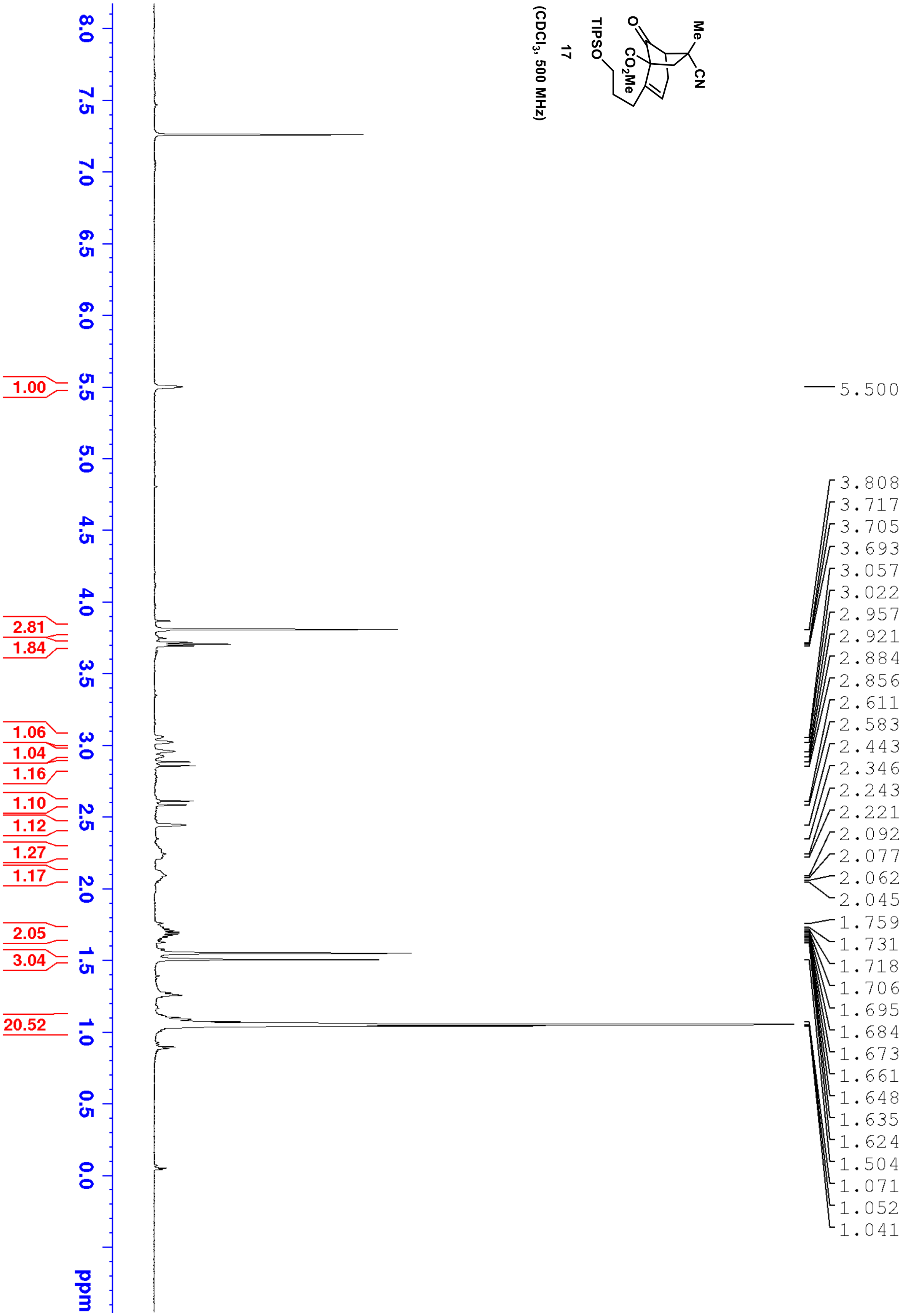
Me
CN
O
CO2Me
TIPSO
17
(CDCl3, 500 MHz)
5.500
3.808
3.717
3.705
3.693
3.057
3.022
2.957
2.921
2.884
2.856
2.611
2.583
2.443
2.346
2.243
2.221
2.092
2.077
2.062
2.045
1.759
1.731
1.718
1.706
1.695
1.684
1.673
1.661
1.648
1.635
1.624
1.504
1.071
1.052
1.041
1.00
2.81
1.84
1.06
1.04
1.16
1.10
1.12
1.27
1.17
2.05
3.04
20.52
8.0
7.5
7.0
6.5
6.0
5.5
5.0
4.5
4.0
3.5
3.0
2.5
2.0
1.5
1.0
0.5
0.0
ppm

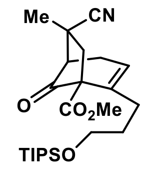


17

($CDCl_3$, 126 MHz)

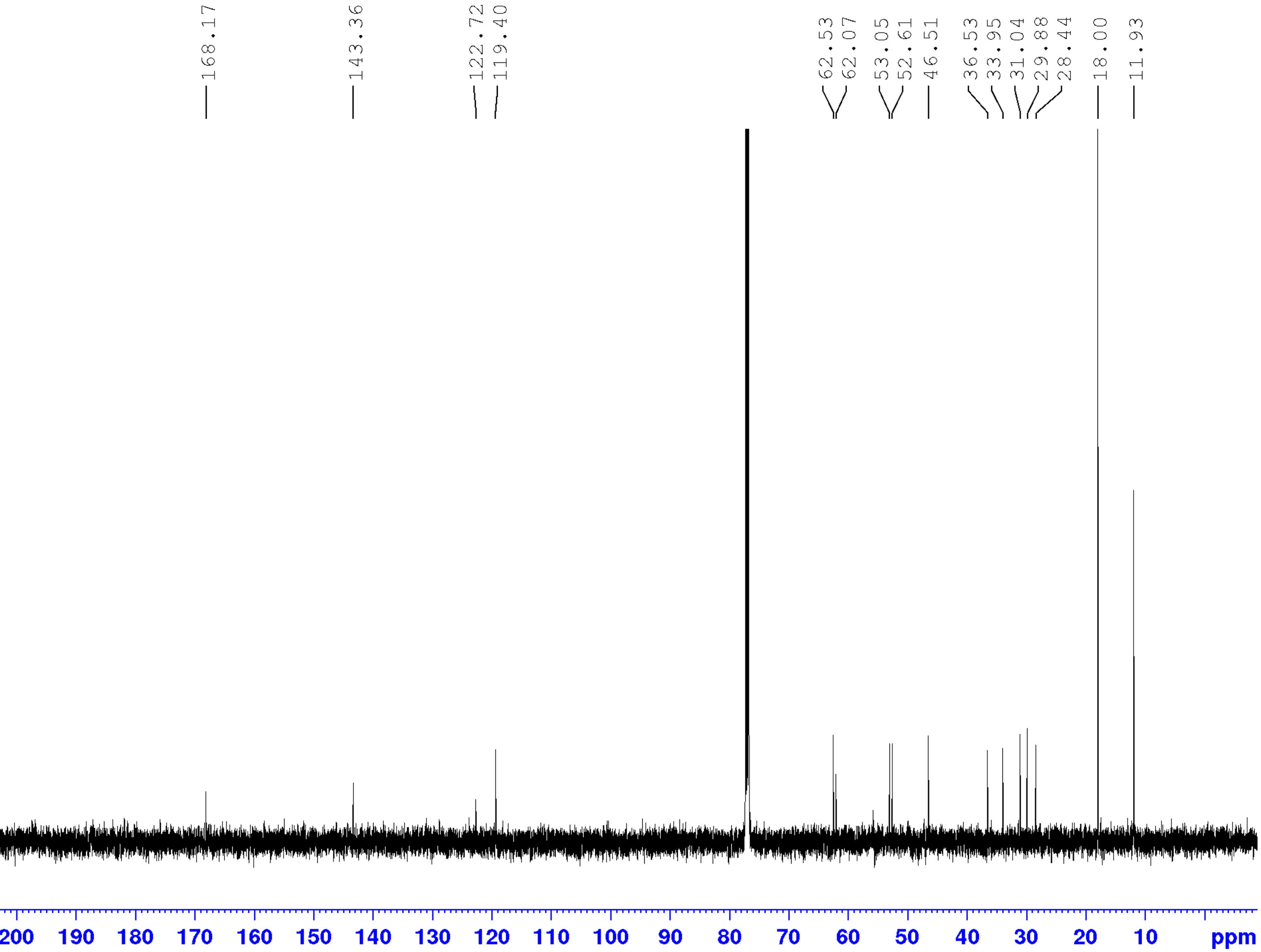

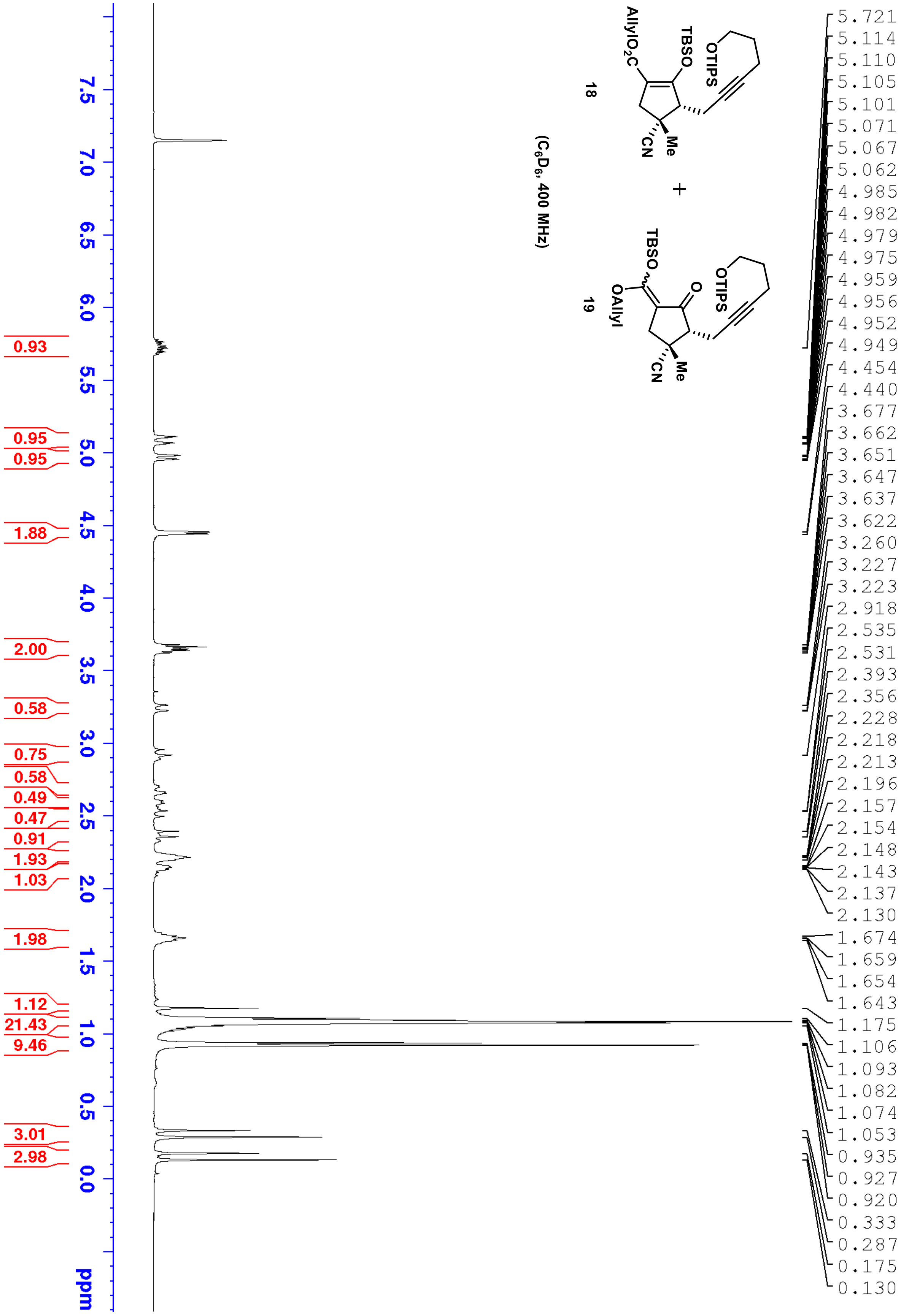

OTIPS
TBSO
AllylO$_2$C
Me
CN
18
+
OTIPS
O
TBSO
OAllyl
Me
CN
19
($C_6D_6$, 400 MHz)
5.721
5.114
5.110
5.105
5.101
5.071
5.067
5.062
4.985
4.982
4.979
4.975
4.959
4.956
4.952
4.949
4.454
4.440
3.677
3.662
3.651
3.647
3.637
3.622
3.260
3.227
3.223
2.918
2.535
2.531
2.393
2.356
2.228
2.218
2.213
2.196
2.157
2.154
2.148
2.143
2.137
2.130
1.674
1.659
1.654
1.643
1.175
1.106
1.093
1.082
1.074
1.053
0.935
0.927
0.920
0.333
0.287
0.175
0.130
0.93
0.95
0.95
1.88
2.00
0.58
0.75
0.58
0.49
0.47
0.91
1.93
1.03
1.98
1.12
21.43
9.46
3.01
2.98
7.5
7.0
6.5
6.0
5.5
5.0
4.5
4.0
3.5
3.0
2.5
2.0
1.5
1.0
0.5
0.0
ppm

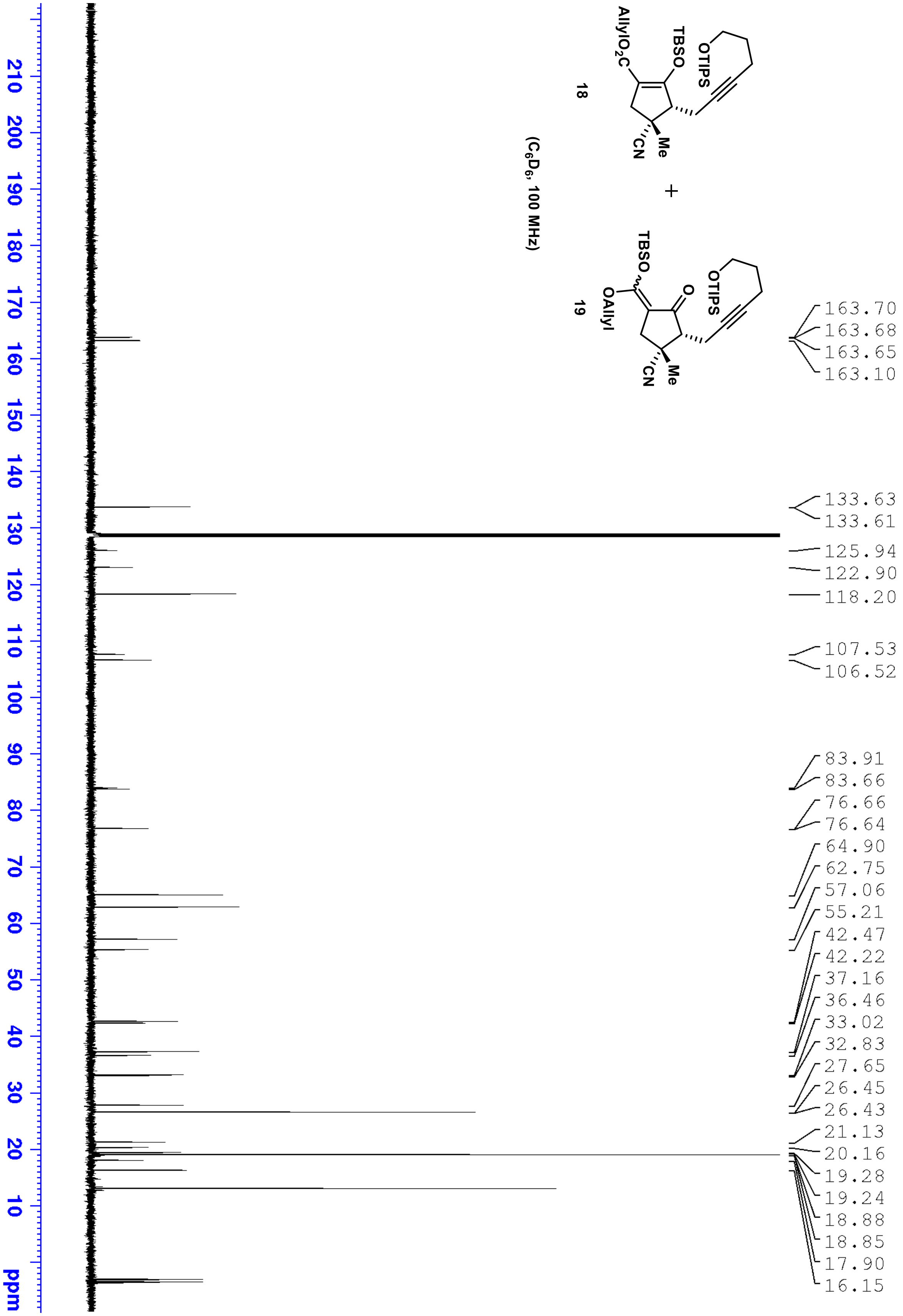

OTIPS
TBSO
AllylO2C
18
Me
CN
+
OTIPS
O
TBSO
OAllyl
19
Me
CN
(C6D6, 100 MHz)
163.70
163.68
163.65
163.10
133.63
133.61
125.94
122.90
118.20
107.53
106.52
83.91
83.66
76.66
76.64
64.90
62.75
57.06
55.21
42.47
42.22
37.16
36.46
33.02
32.83
27.65
26.45
26.43
21.13
20.16
19.28
19.24
18.88
18.85
17.90
16.15
210
200
190
180
170
160
150
140
130
120
110
100
90
80
70
60
50
40
30
20
10
ppm

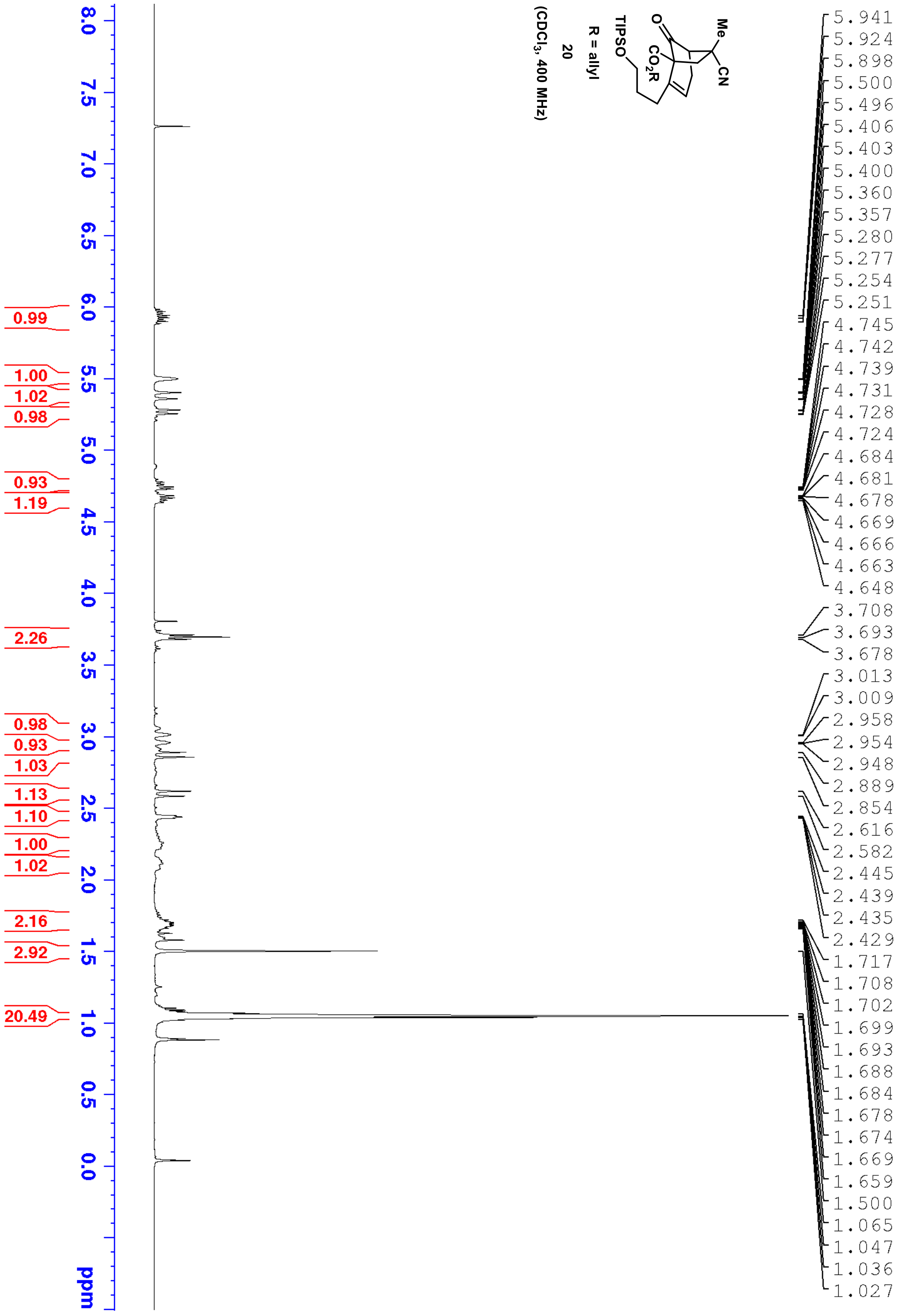
Me
CN
O
CO2R
TIPSO
R = allyl
20
(CDCl3, 400 MHz)
5.941
5.924
5.898
5.500
5.496
5.406
5.403
5.400
5.360
5.357
5.280
5.277
5.254
5.251
4.745
4.742
4.739
4.731
4.728
4.724
4.684
4.681
4.678
4.669
4.666
4.663
4.648
3.708
3.693
3.678
3.013
3.009
2.958
2.954
2.948
2.889
2.854
2.616
2.582
2.445
2.439
2.435
2.429
1.717
1.708
1.702
1.699
1.693
1.688
1.684
1.678
1.674
1.669
1.659
1.500
1.065
1.047
1.036
1.027
0.99
1.00
1.02
0.98
0.93
1.19
2.26
0.98
0.93
1.03
1.13
1.10
1.00
1.02
2.16
2.92
20.49
8.0
7.5
7.0
6.5
6.0
5.5
5.0
4.5
4.0
3.5
3.0
2.5
2.0
1.5
1.0
0.5
0.0
ppm

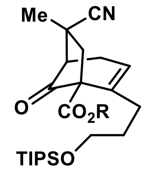


R = allyl

20

($CDCl_3$, 100 MHz)

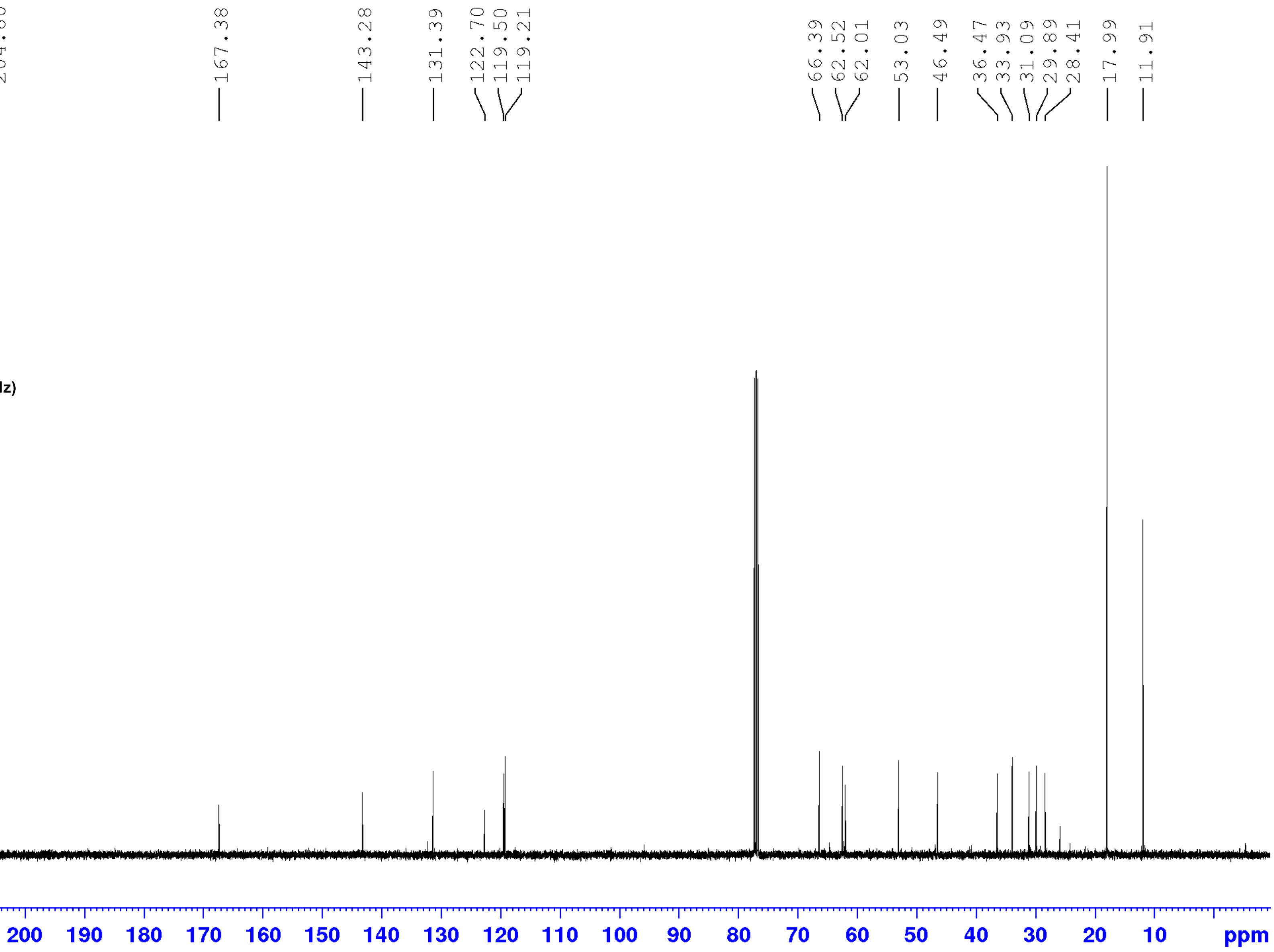

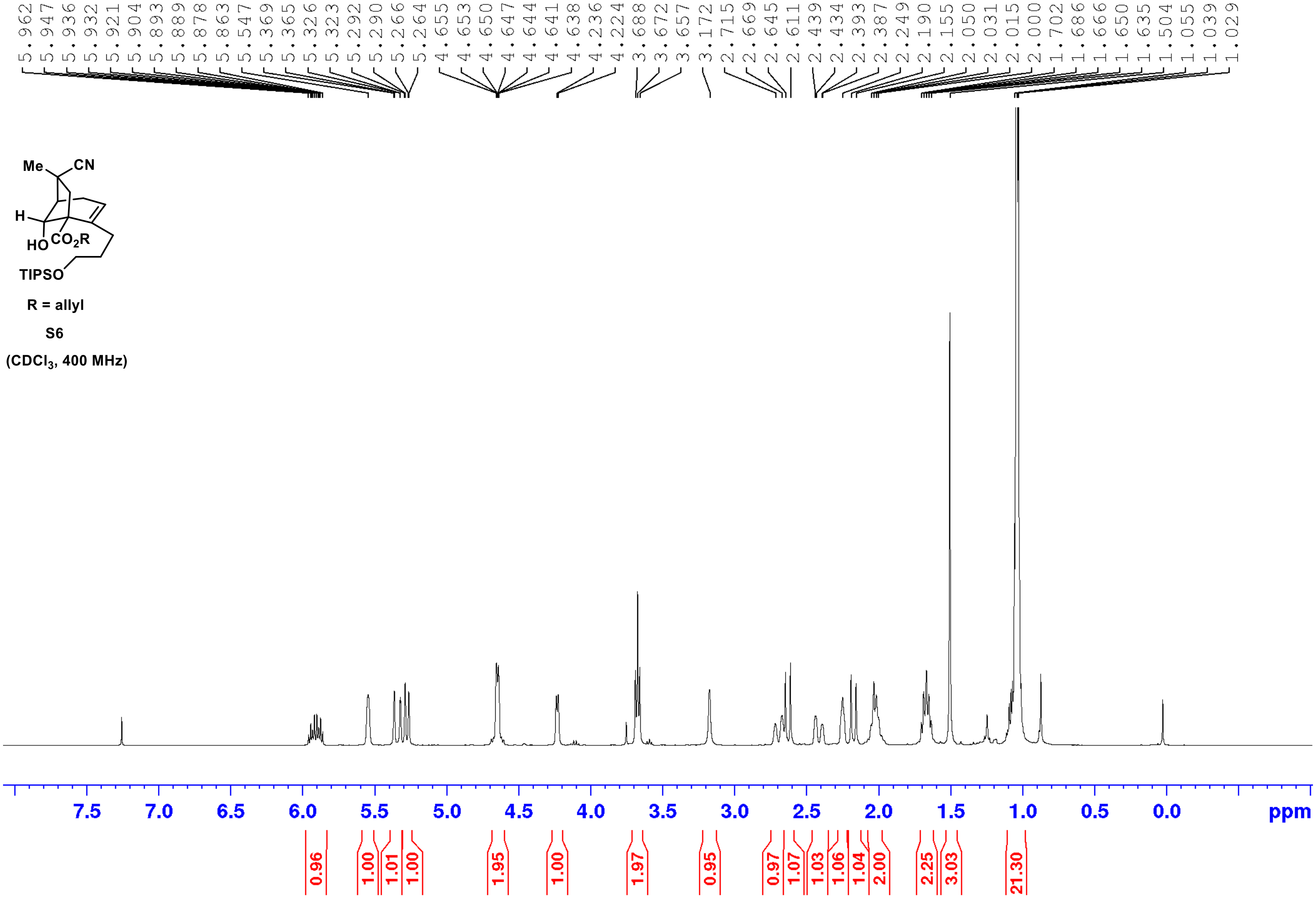

R = allyl

**S6**

($CDCl_3$, 400 MHz)

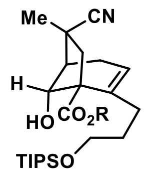


R = allyl

**S6**

**($CDCl_3$, 100 MHz)**

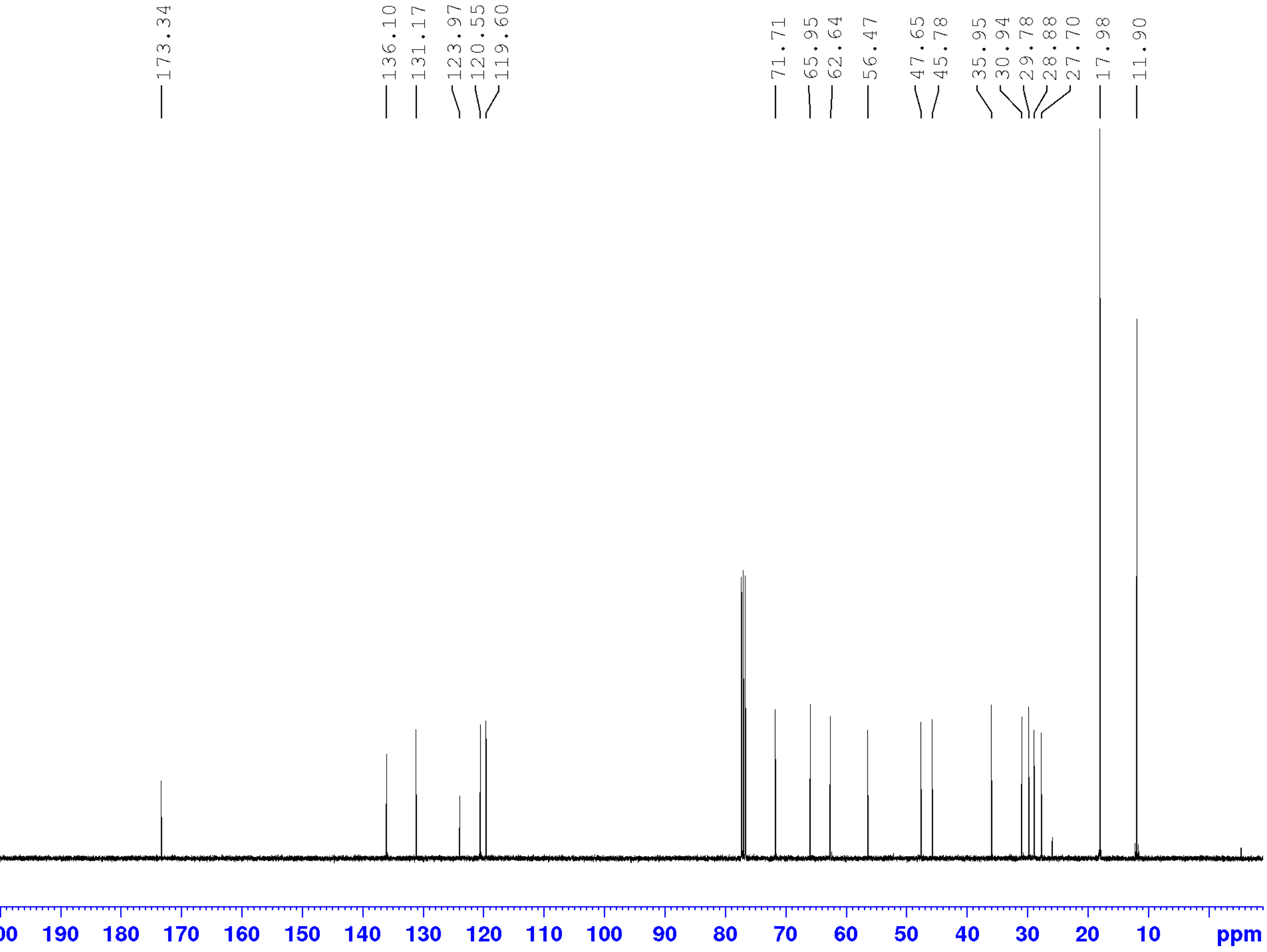

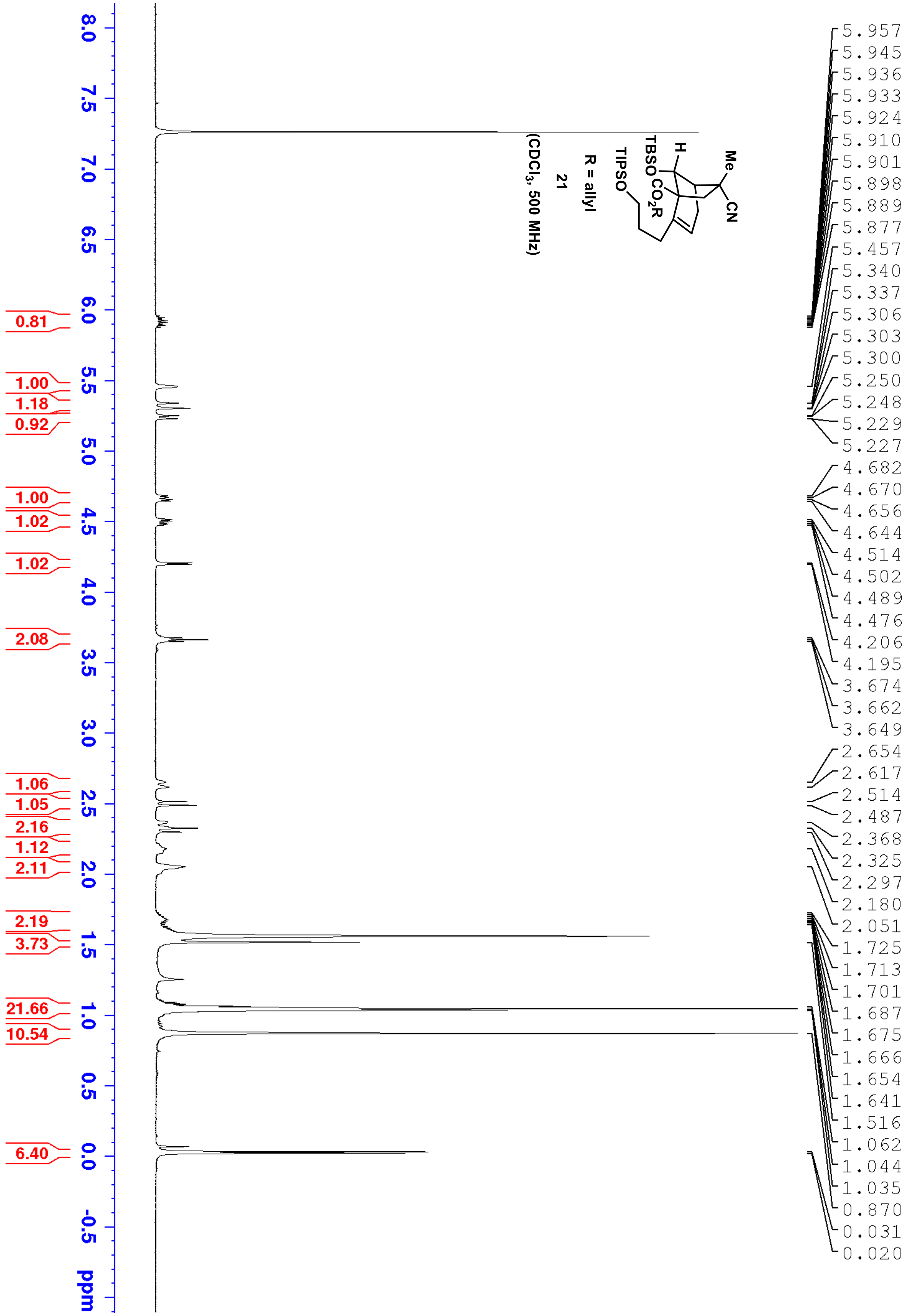
Me
CN
H
TBSO
CO2R
TIPSO
R = allyl
21
(CDCl3, 500 MHz)
5.957
5.945
5.936
5.933
5.924
5.910
5.901
5.898
5.889
5.877
5.457
5.340
5.337
5.306
5.303
5.300
5.250
5.248
5.229
5.227
4.682
4.670
4.656
4.644
4.514
4.502
4.489
4.476
4.206
4.195
3.674
3.662
3.649
2.654
2.617
2.514
2.487
2.368
2.325
2.297
2.180
2.051
1.725
1.713
1.701
1.687
1.675
1.666
1.654
1.641
1.516
1.062
1.044
1.035
0.870
0.031
0.020
0.81
1.00
1.18
0.92
1.00
1.02
1.02
2.08
1.06
1.05
2.16
1.12
2.11
2.19
3.73
21.66
10.54
6.40
8.0
7.5
7.0
6.5
6.0
5.5
5.0
4.5
4.0
3.5
3.0
2.5
2.0
1.5
1.0
0.5
0.0
-0.5
ppm

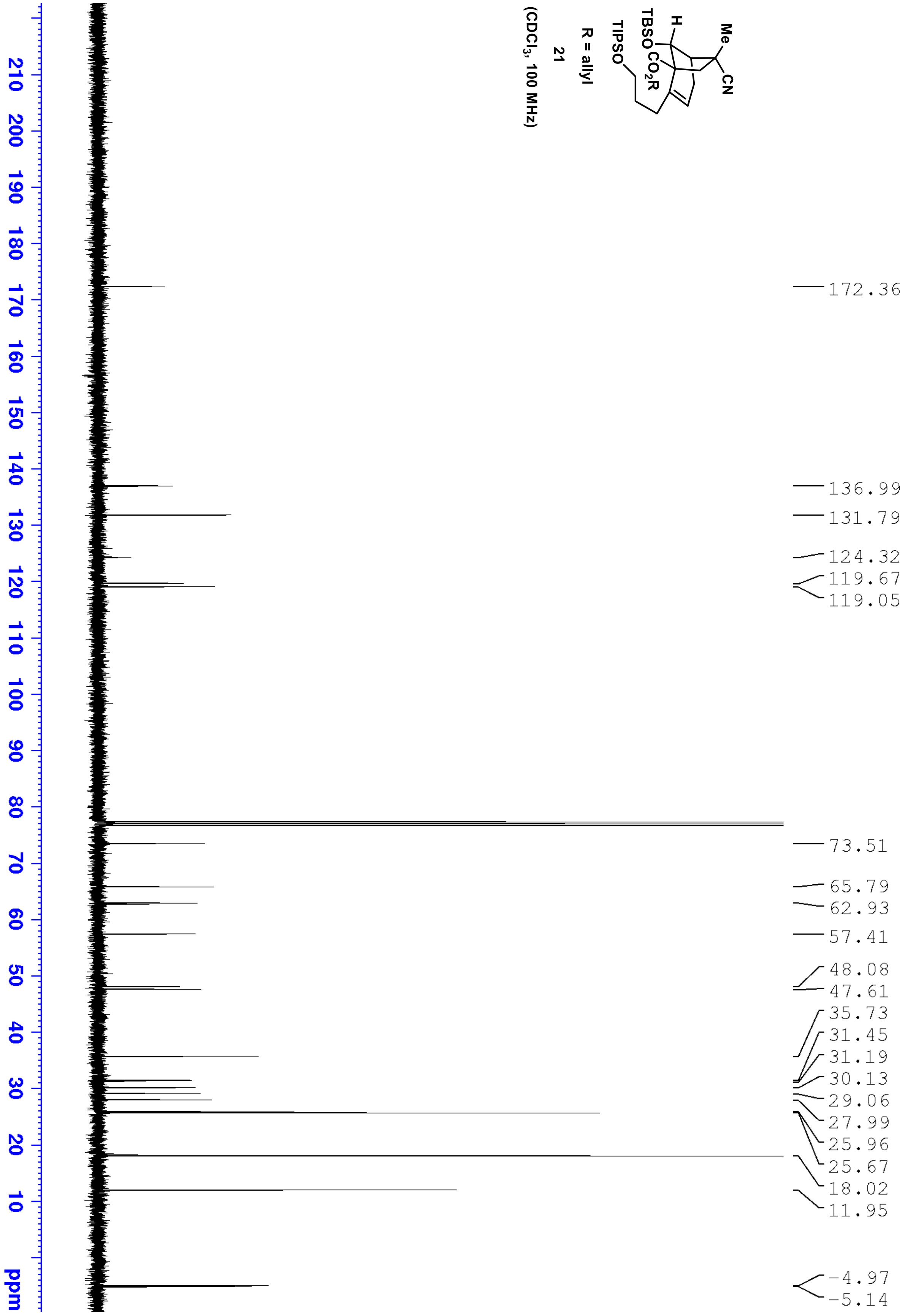
Me
CN
H
TBSO
CO2R
TIPSO
R = allyl
21
(CDCl3, 100 MHz)
172.36
136.99
131.79
124.32
119.67
119.05
73.51
65.79
62.93
57.41
48.08
47.61
35.73
31.45
31.19
30.13
29.06
27.99
25.96
25.67
18.02
11.95
-4.97
-5.14
210
200
190
180
170
160
150
140
130
120
110
100
90
80
70
60
50
40
30
20
10
ppm

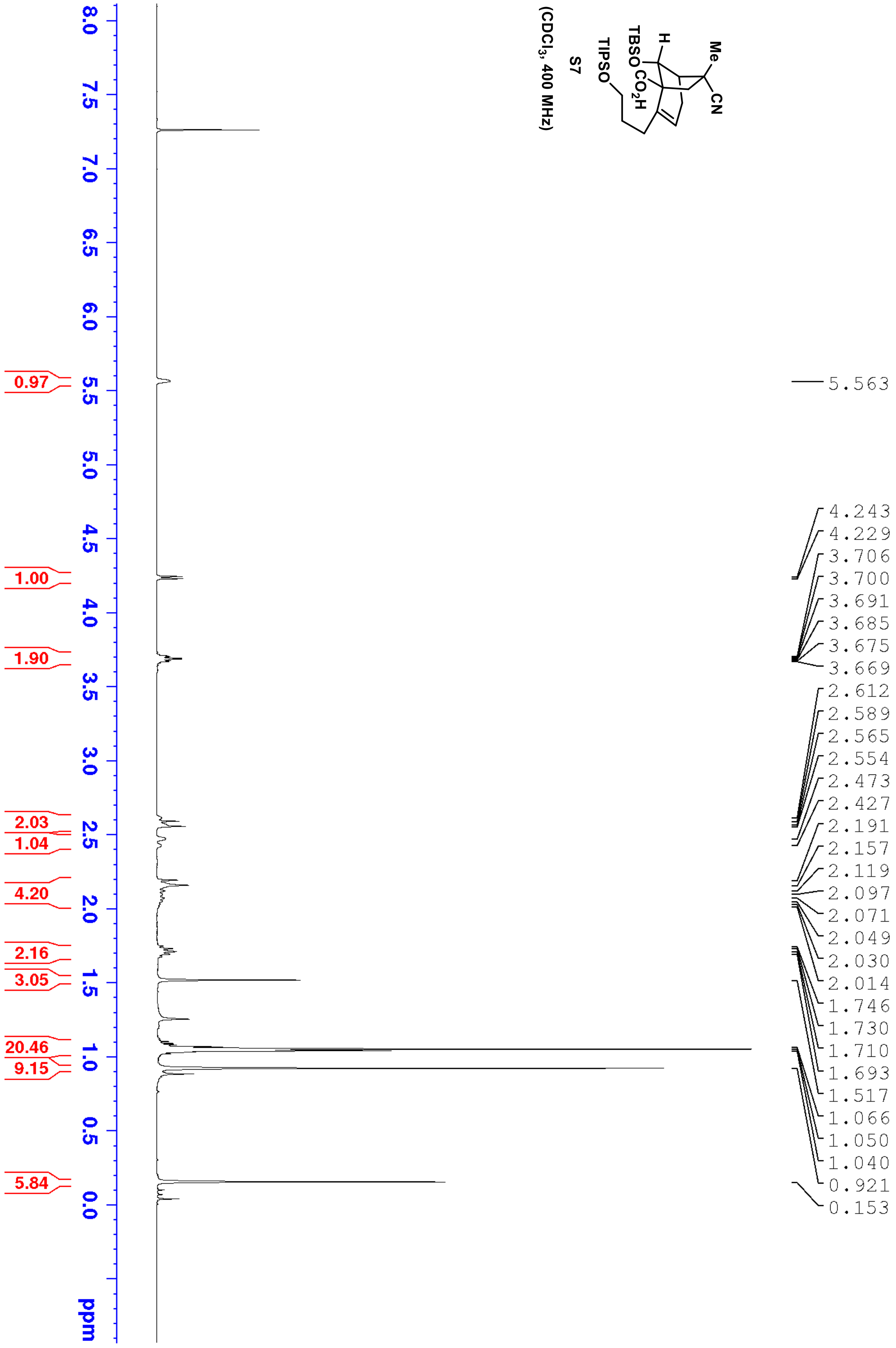
Me
CN
H
TBSO
CO2H
TIPSO
S7
(CDCl3, 400 MHz)
5.563
4.243
4.229
3.706
3.700
3.691
3.685
3.675
3.669
2.612
2.589
2.565
2.554
2.473
2.427
2.191
2.157
2.119
2.097
2.071
2.049
2.030
2.014
1.746
1.730
1.710
1.693
1.517
1.066
1.050
1.040
0.921
0.153
0.97
1.00
1.90
2.03
1.04
4.20
2.16
3.05
20.46
9.15
5.84
8.0
7.5
7.0
6.5
6.0
5.5
5.0
4.5
4.0
3.5
3.0
2.5
2.0
1.5
1.0
0.5
0.0
ppm

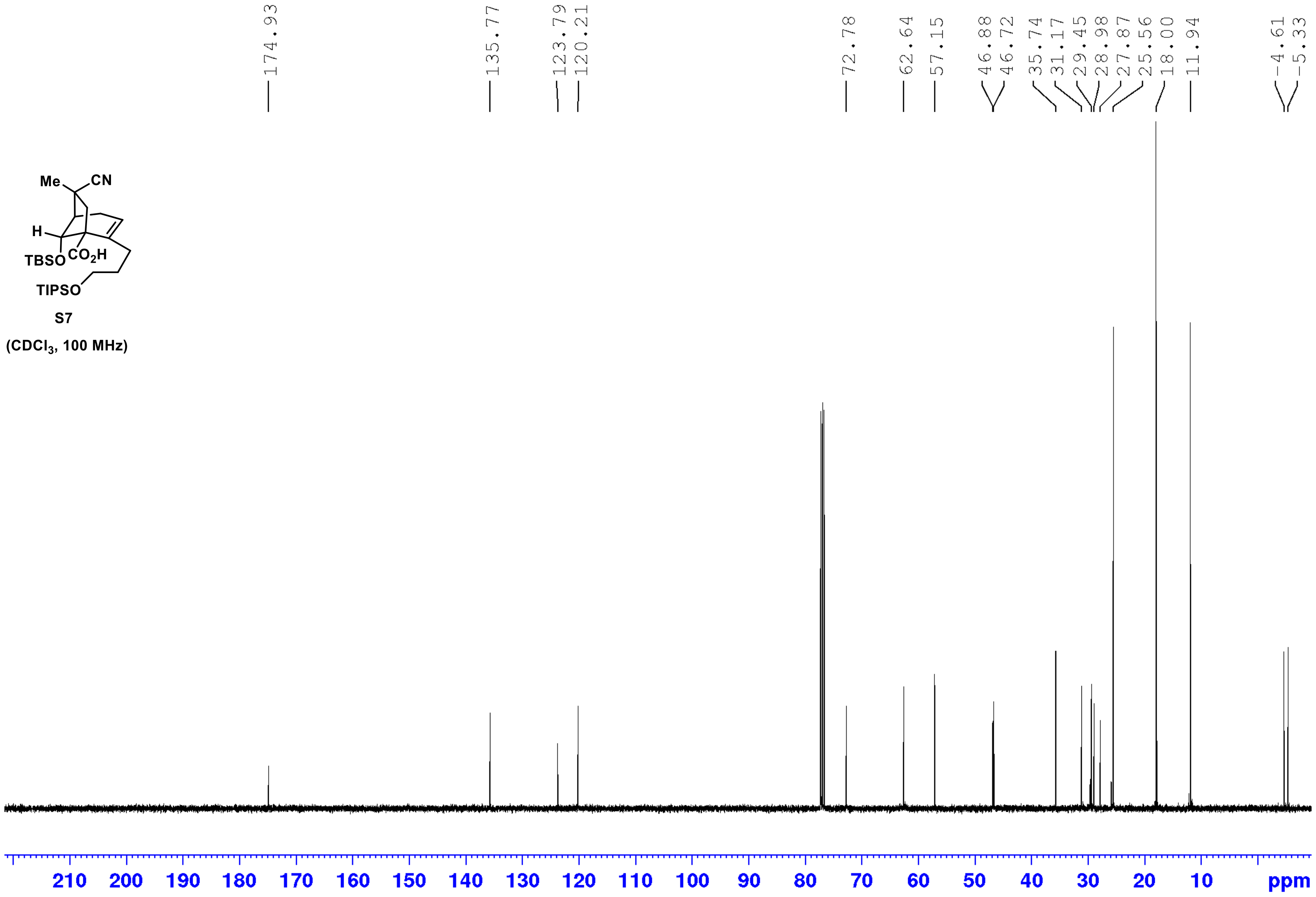


S7

($CDCl_3$, 100 MHz)

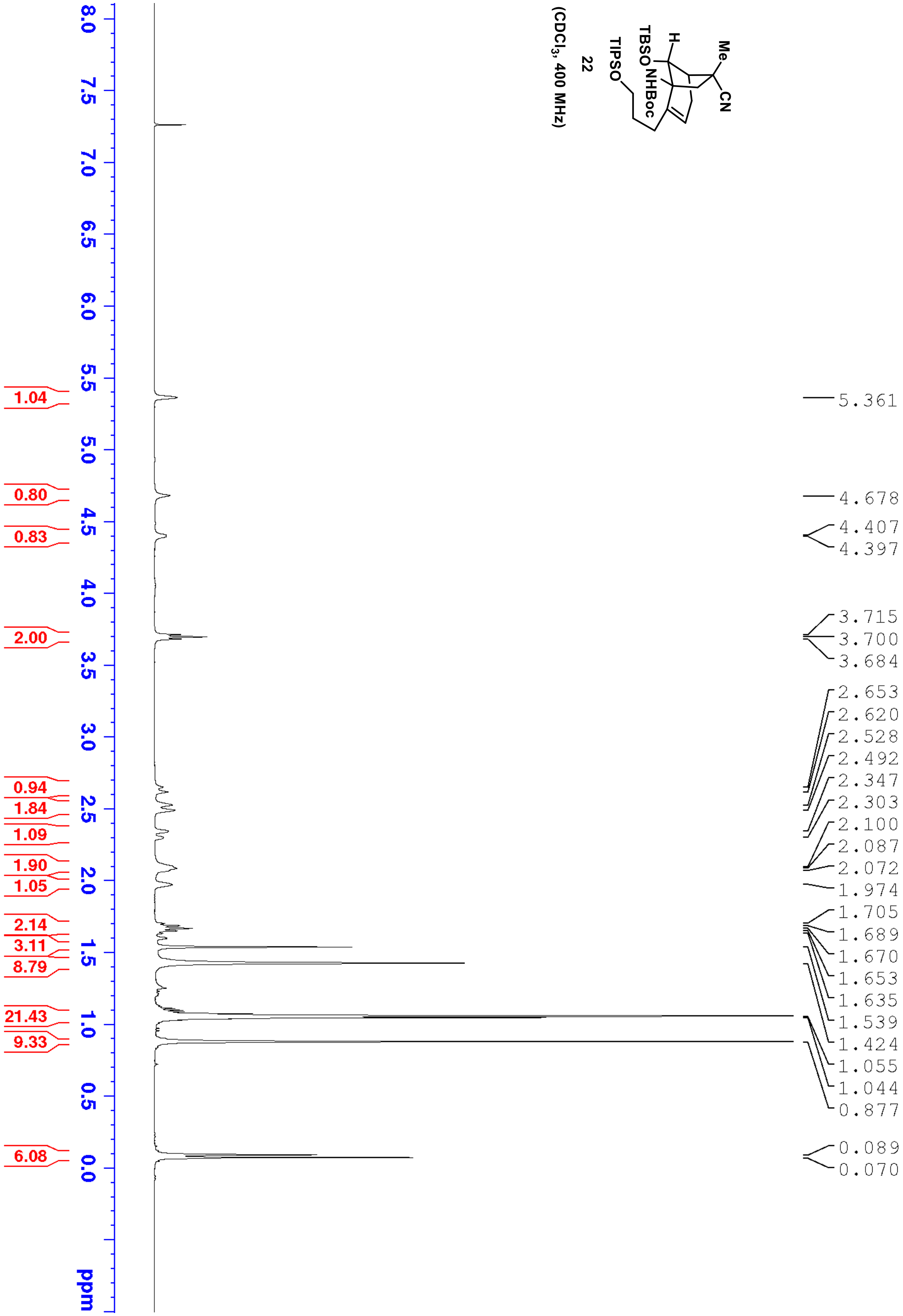
Me
CN
H
TBSO
NHBoc
TIPSO
22
(CDCl3, 400 MHz)
5.361
4.678
4.407
4.397
3.715
3.700
3.684
2.653
2.620
2.528
2.492
2.347
2.303
2.100
2.087
2.072
1.974
1.705
1.689
1.670
1.653
1.635
1.539
1.424
1.055
1.044
0.877
0.089
0.070
1.04
0.80
0.83
2.00
0.94
1.84
1.09
1.90
1.05
2.14
3.11
8.79
21.43
9.33
6.08
8.0
7.5
7.0
6.5
6.0
5.5
5.0
4.5
4.0
3.5
3.0
2.5
2.0
1.5
1.0
0.5
0.0
ppm

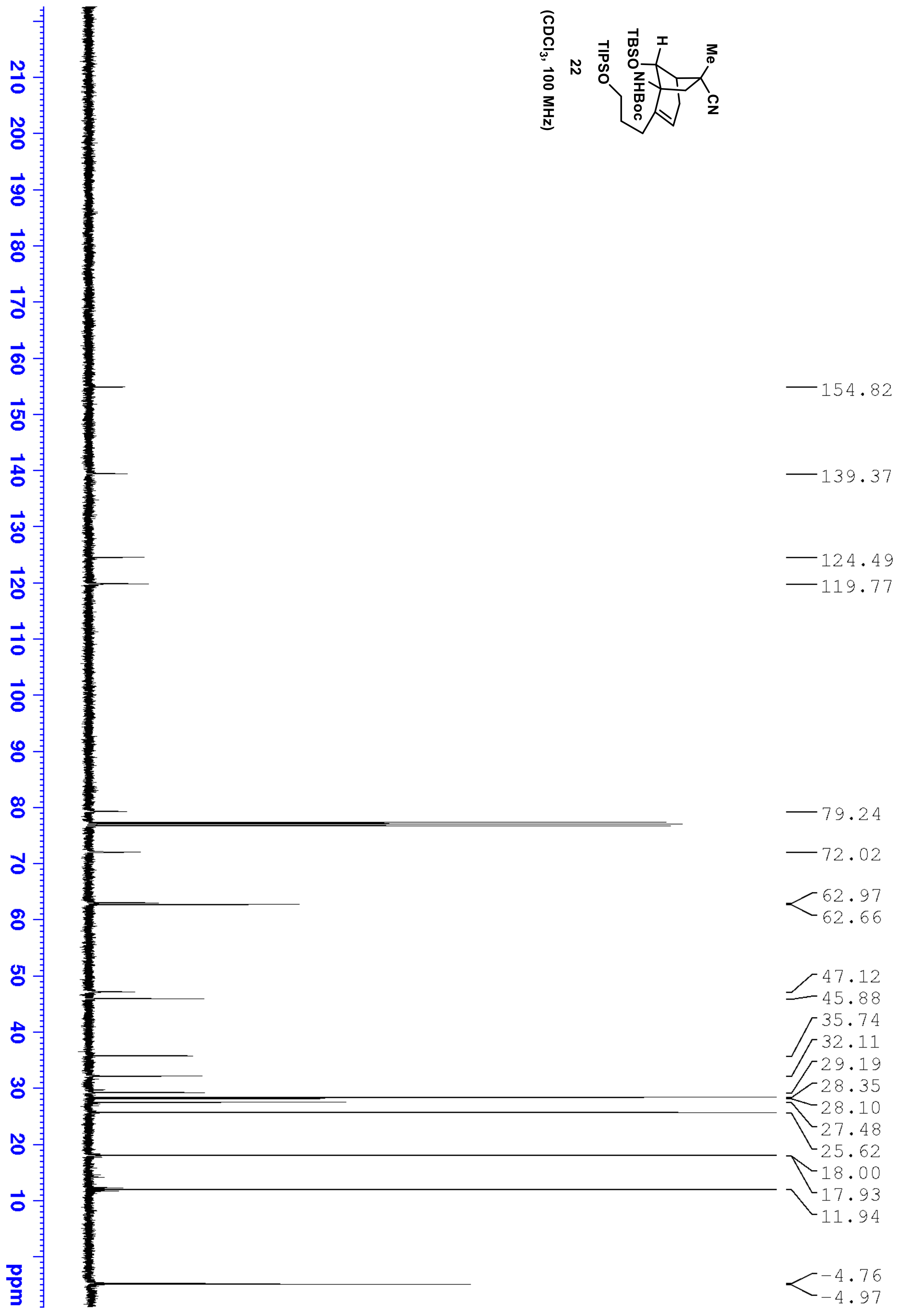
Me
CN
H
TBSO
NHBoc
TIPSO
22
(CDCl3, 100 MHz)
154.82
139.37
124.49
119.77
79.24
72.02
62.97
62.66
47.12
45.88
35.74
32.11
29.19
28.35
28.10
27.48
25.62
18.00
17.93
11.94
-4.76
-4.97
210
200
190
180
170
160
150
140
130
120
110
100
90
80
70
60
50
40
30
20
10
ppm

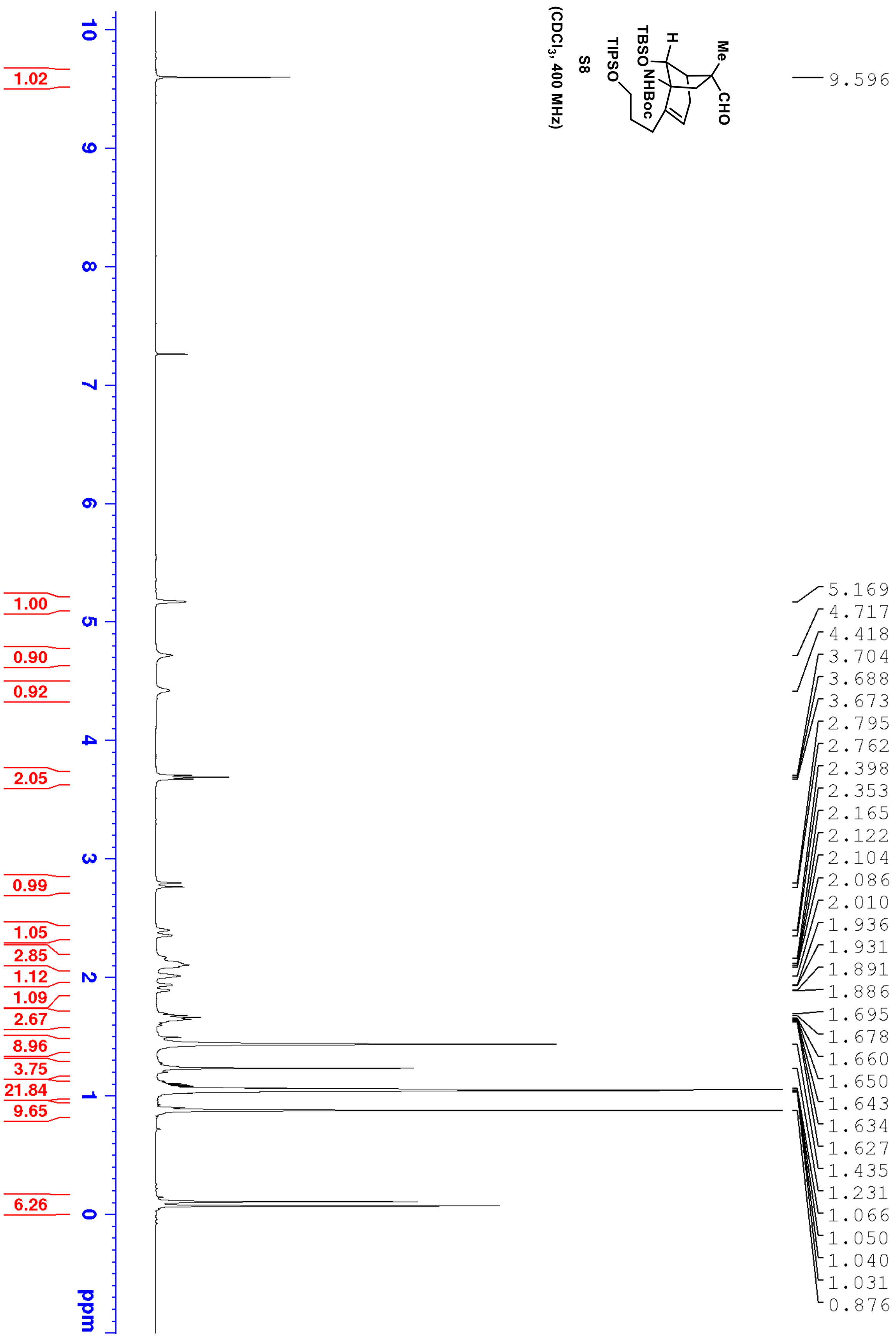

Me
CHO
H
TBSO
NHBoc
TIPSO
S8
(CDCl$_3$, 400 MHz)
9.596
5.169
4.717
4.418
3.704
3.688
3.673
2.795
2.762
2.398
2.353
2.165
2.122
2.104
2.086
2.010
1.936
1.931
1.891
1.886
1.695
1.678
1.660
1.650
1.643
1.634
1.627
1.435
1.231
1.066
1.050
1.040
1.031
0.876
1.02
1.00
0.90
0.92
2.05
0.99
1.05
2.85
1.12
1.09
2.67
8.96
3.75
21.84
9.65
6.26
10
9
8
7
6
5
4
3
2
1
0
ppm

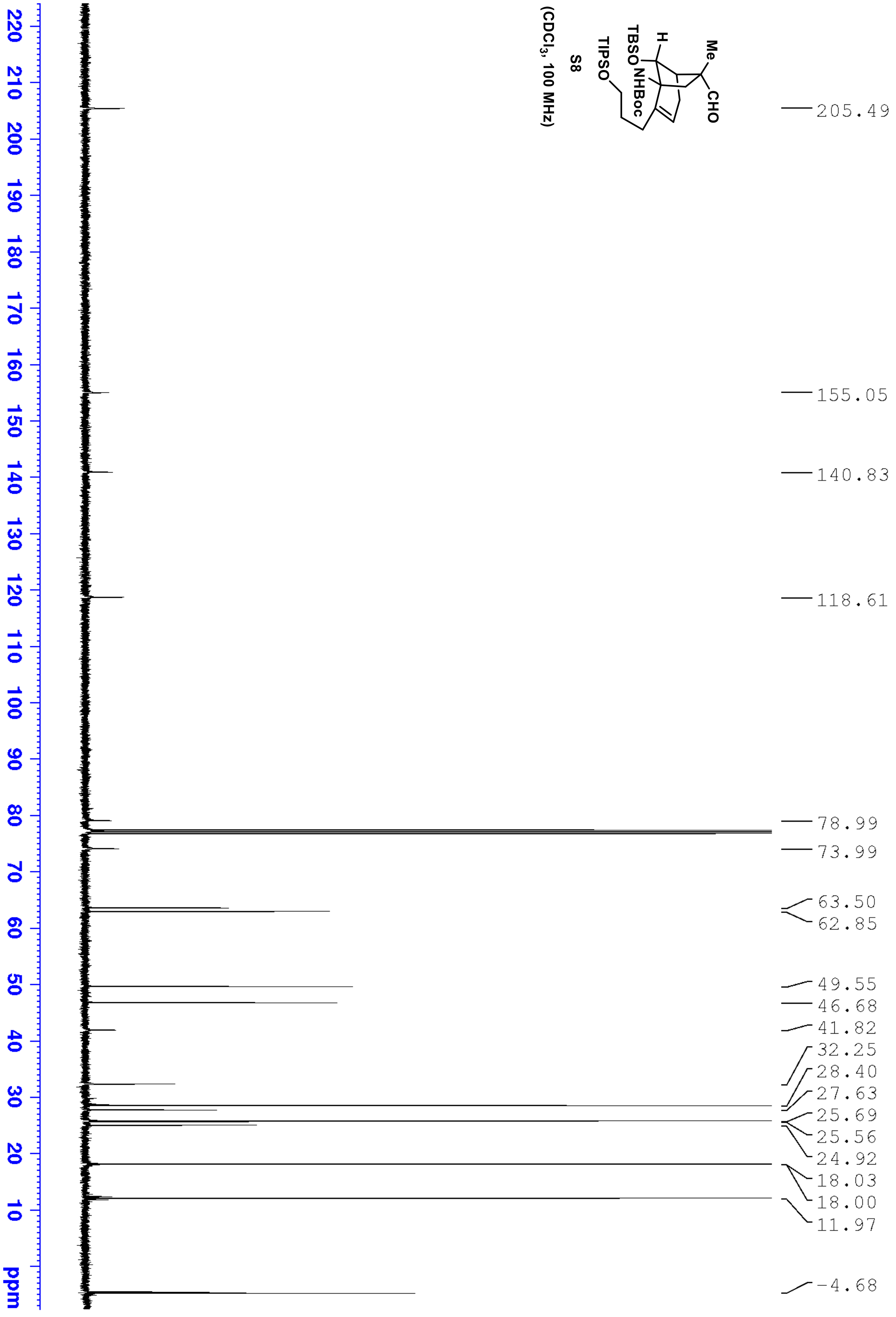
Me
CHO
H
TBSO
NHBoc
TIPSO
S8
(CDCl3, 100 MHz)
205.49
155.05
140.83
118.61
78.99
73.99
63.50
62.85
49.55
46.68
41.82
32.25
28.40
27.63
25.69
25.56
24.92
18.03
18.00
11.97
-4.68
220
210
200
190
180
170
160
150
140
130
120
110
100
90
80
70
60
50
40
30
20
10
ppm

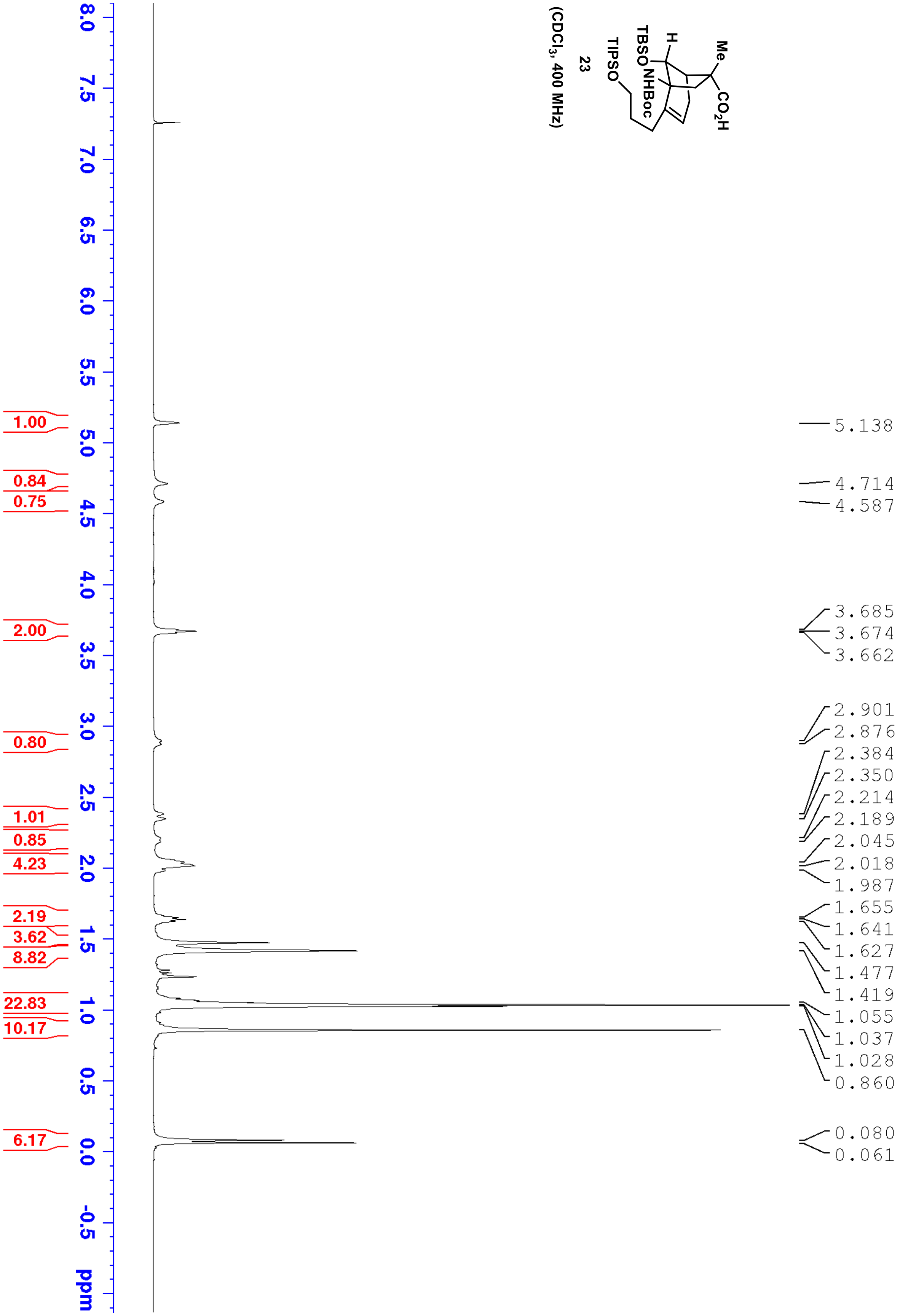
Me
CO2H
H
TBSO
NHBoc
TIPSO
23
(CDCl3, 400 MHz)
5.138
4.714
4.587
3.685
3.674
3.662
2.901
2.876
2.384
2.350
2.214
2.189
2.045
2.018
1.987
1.655
1.641
1.627
1.477
1.419
1.055
1.037
1.028
0.860
0.080
0.061
1.00
0.84
0.75
2.00
0.80
1.01
0.85
4.23
2.19
3.62
8.82
22.83
10.17
6.17
8.0
7.5
7.0
6.5
6.0
5.5
5.0
4.5
4.0
3.5
3.0
2.5
2.0
1.5
1.0
0.5
0.0
-0.5
ppm

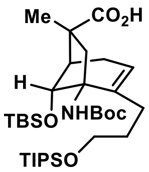


**23**

**($CDCl_3$, 100 MHz)**

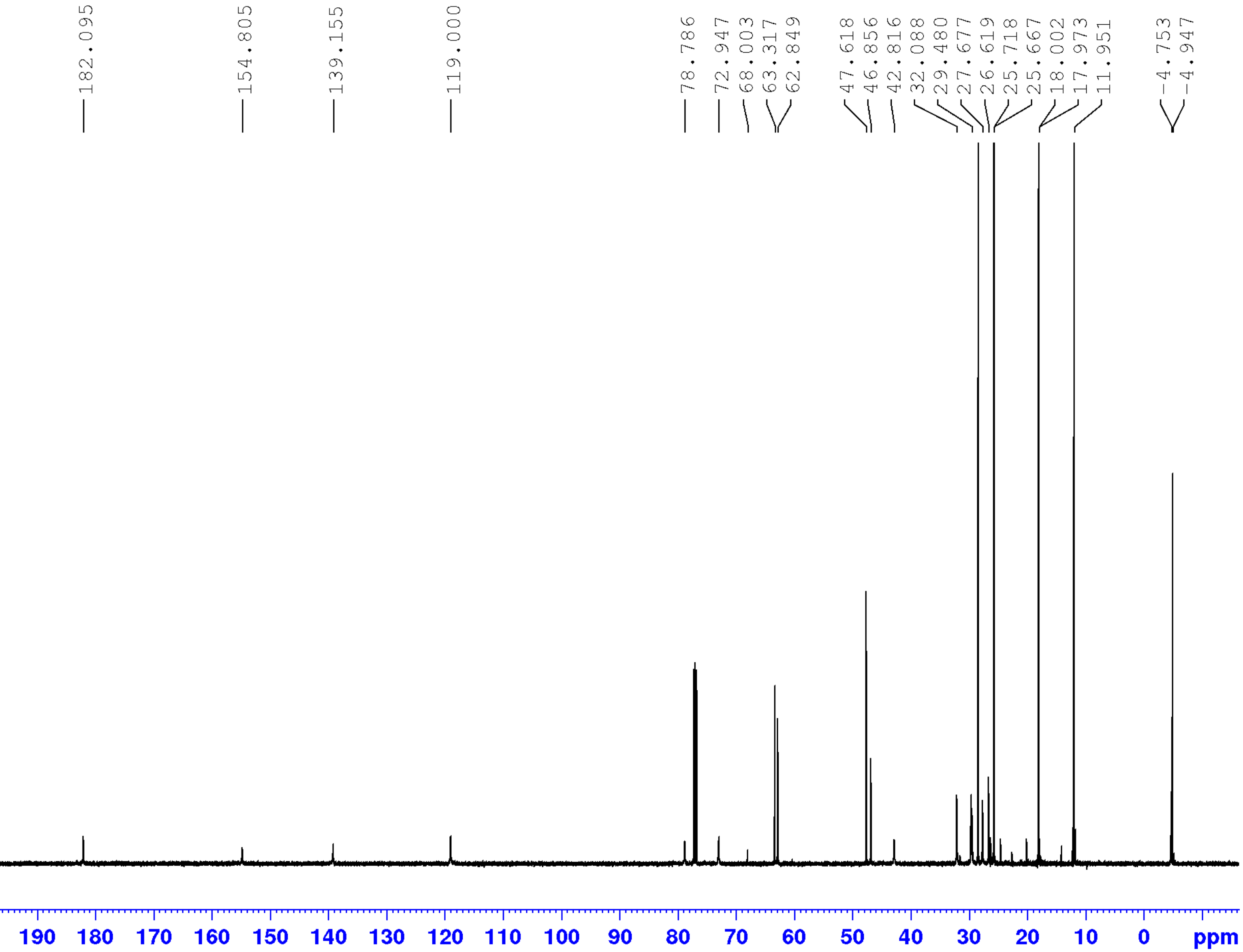

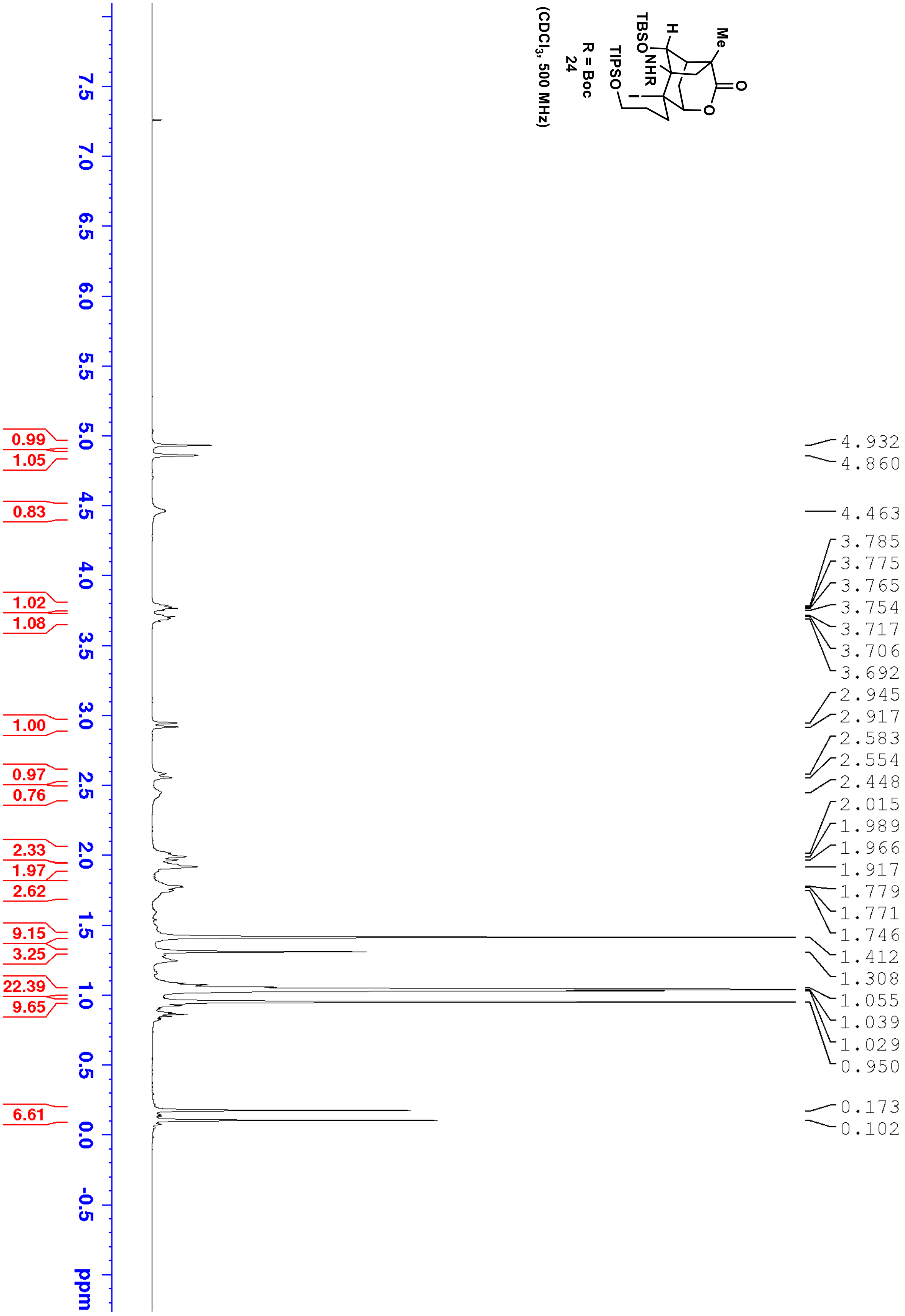
Me
O
H
TBSO
NHR
TIPSO
R = Boc
24
(CDCl3, 500 MHz)
7.5
7.0
6.5
6.0
5.5
5.0
4.5
4.0
3.5
3.0
2.5
2.0
1.5
1.0
0.5
0.0
-0.5
ppm
0.99
1.05
0.83
1.02
1.08
1.00
0.97
0.76
2.33
1.97
2.62
9.15
3.25
22.39
9.65
6.61
4.932
4.860
4.463
3.785
3.775
3.765
3.754
3.717
3.706
3.692
2.945
2.917
2.583
2.554
2.448
2.015
1.989
1.966
1.917
1.779
1.771
1.746
1.412
1.308
1.055
1.039
1.029
0.950
0.173
0.102

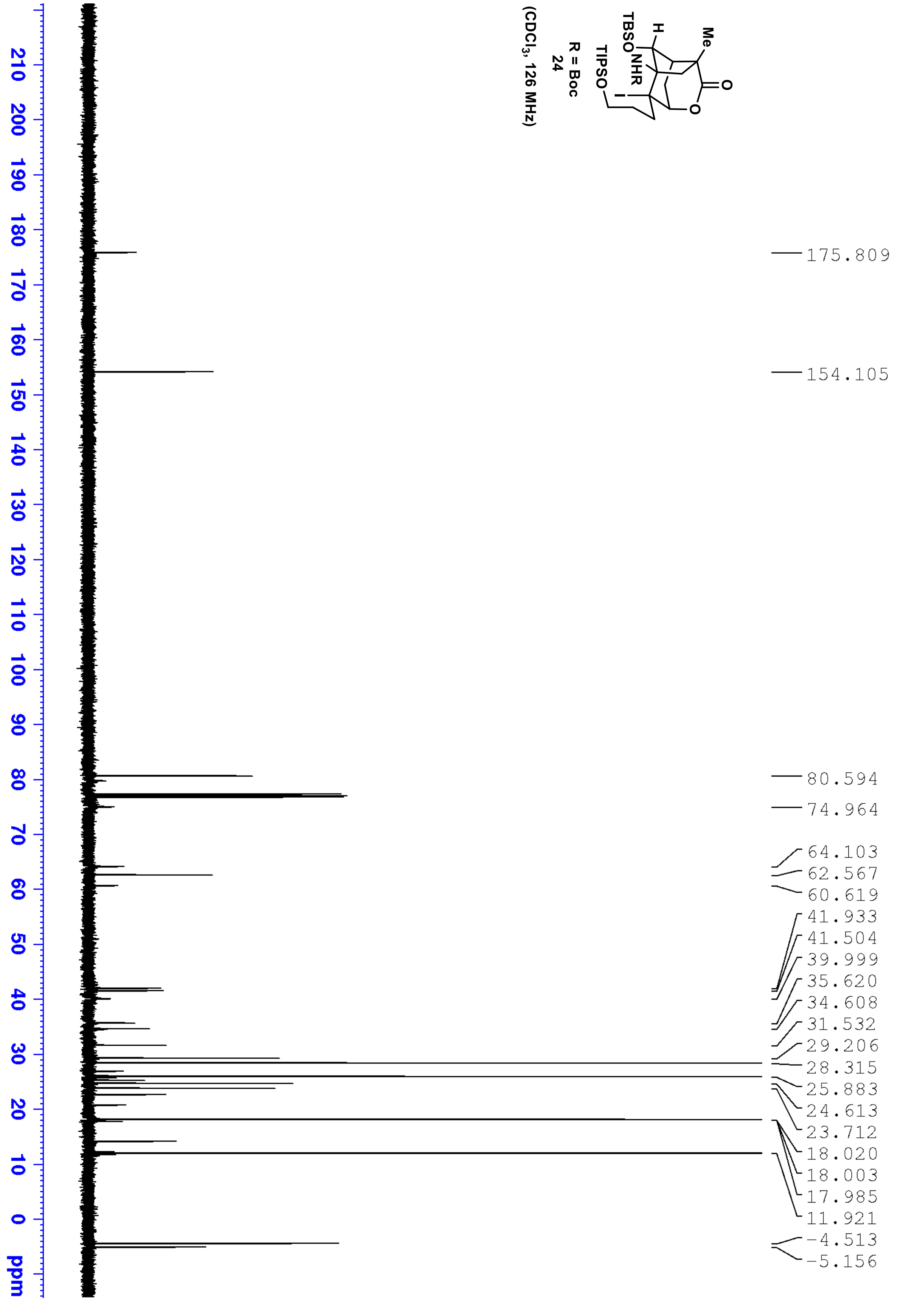

Me
O
O
H
TBSO
NHR
I
TIPSO
R = Boc
24
(CDCl3, 126 MHz)
175.809
154.105
80.594
74.964
64.103
62.567
60.619
41.933
41.504
39.999
35.620
34.608
31.532
29.206
28.315
25.883
24.613
23.712
18.020
18.003
17.985
11.921
-4.513
-5.156
210
200
190
180
170
160
150
140
130
120
110
100
90
80
70
60
50
40
30
20
10
0
ppm

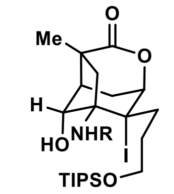

R = Boc
26
($CDCl_3$, 500 MHz)

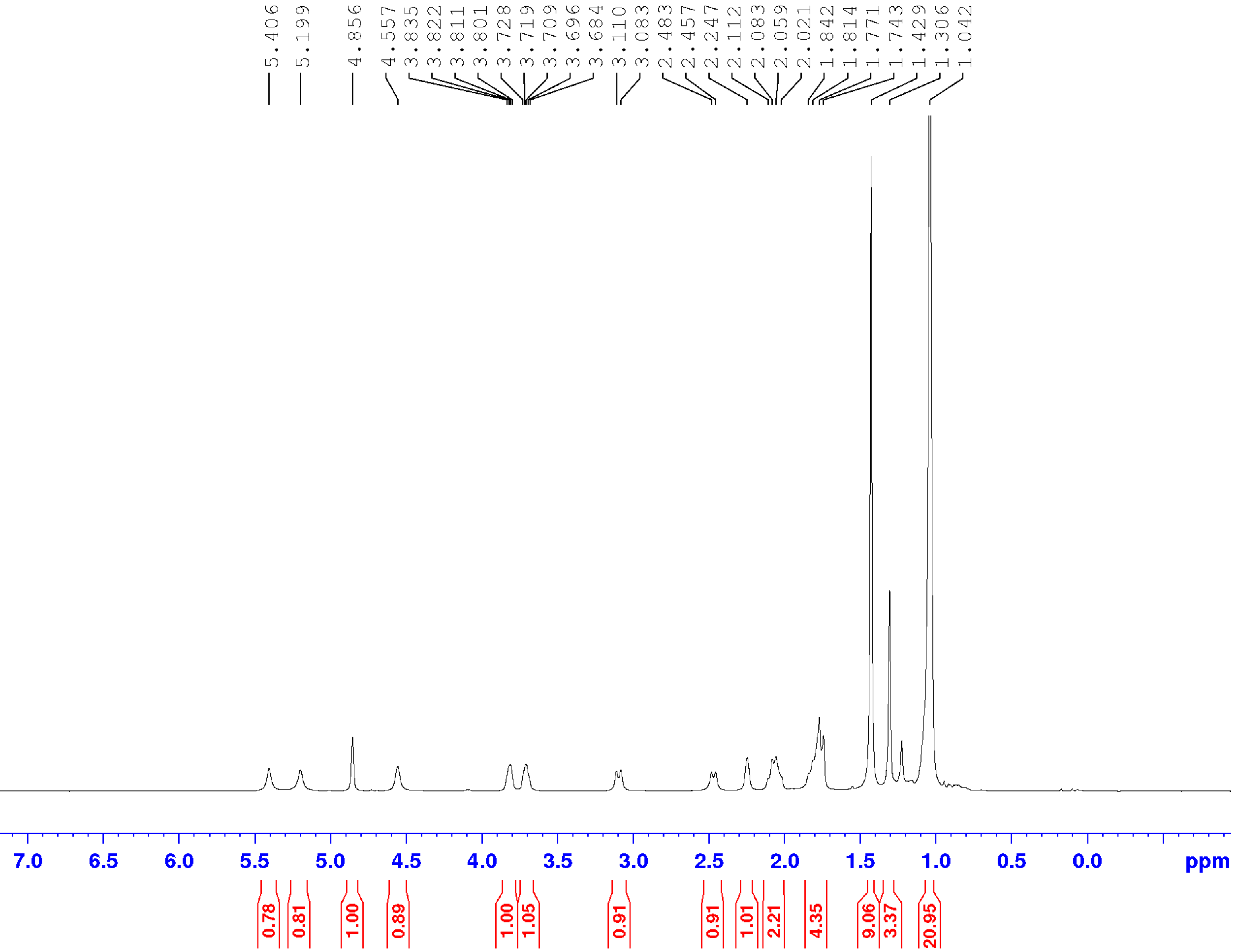

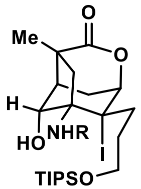


R = Boc
26
(CDCl$_3$, 126 MHz)

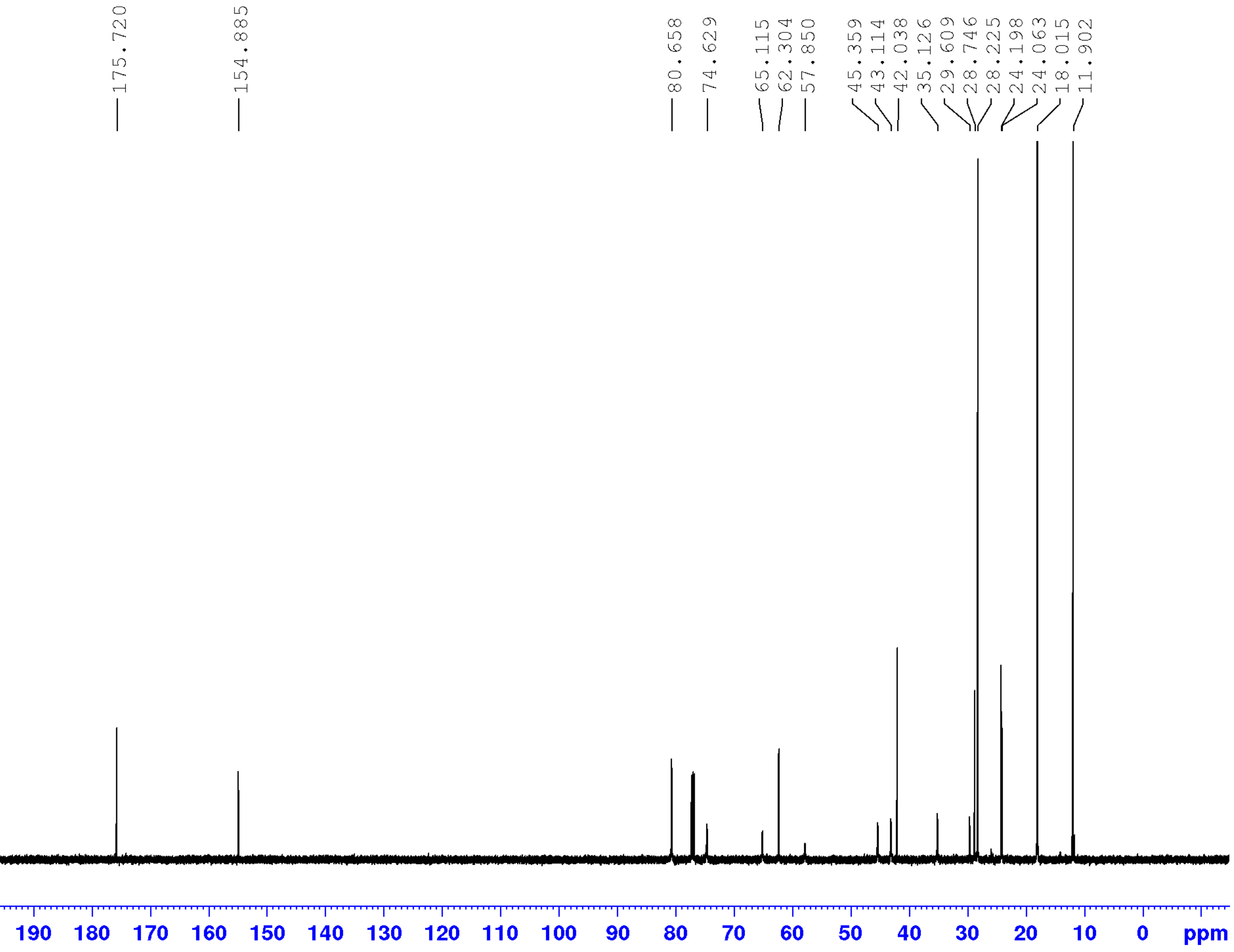

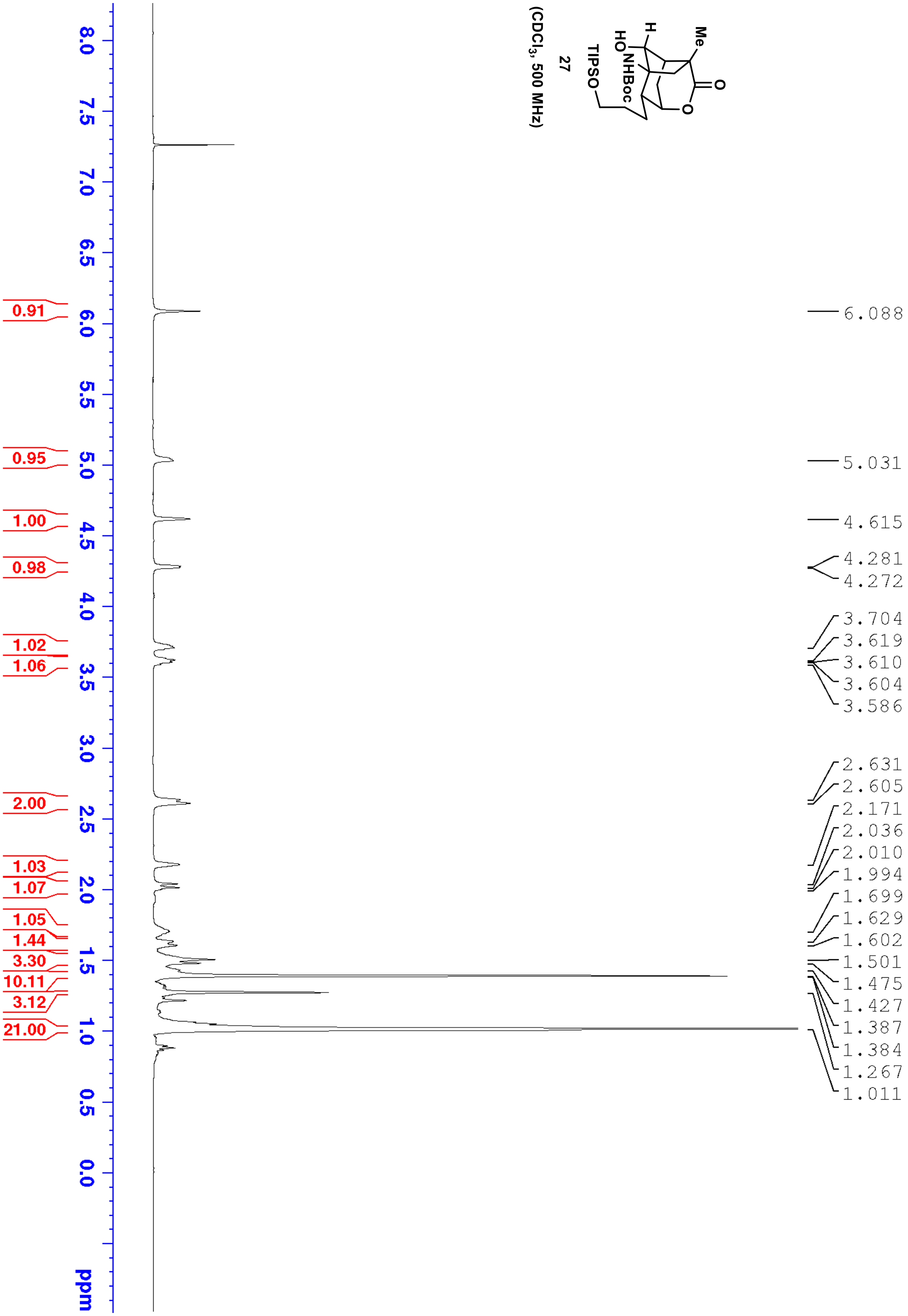
27
(CDCl3, 500 MHz)
Me
O
O
H
HO
NHBoc
TIPSO
6.088
5.031
4.615
4.281
4.272
3.704
3.619
3.610
3.604
3.586
2.631
2.605
2.171
2.036
2.010
1.994
1.699
1.629
1.602
1.501
1.475
1.427
1.387
1.384
1.267
1.011
0.91
0.95
1.00
0.98
1.02
1.06
2.00
1.03
1.07
1.05
1.44
3.30
10.11
3.12
21.00
8.0
7.5
7.0
6.5
6.0
5.5
5.0
4.5
4.0
3.5
3.0
2.5
2.0
1.5
1.0
0.5
0.0
ppm

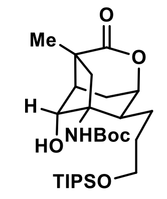


27

(CDCl$_3$, 126 MHz)

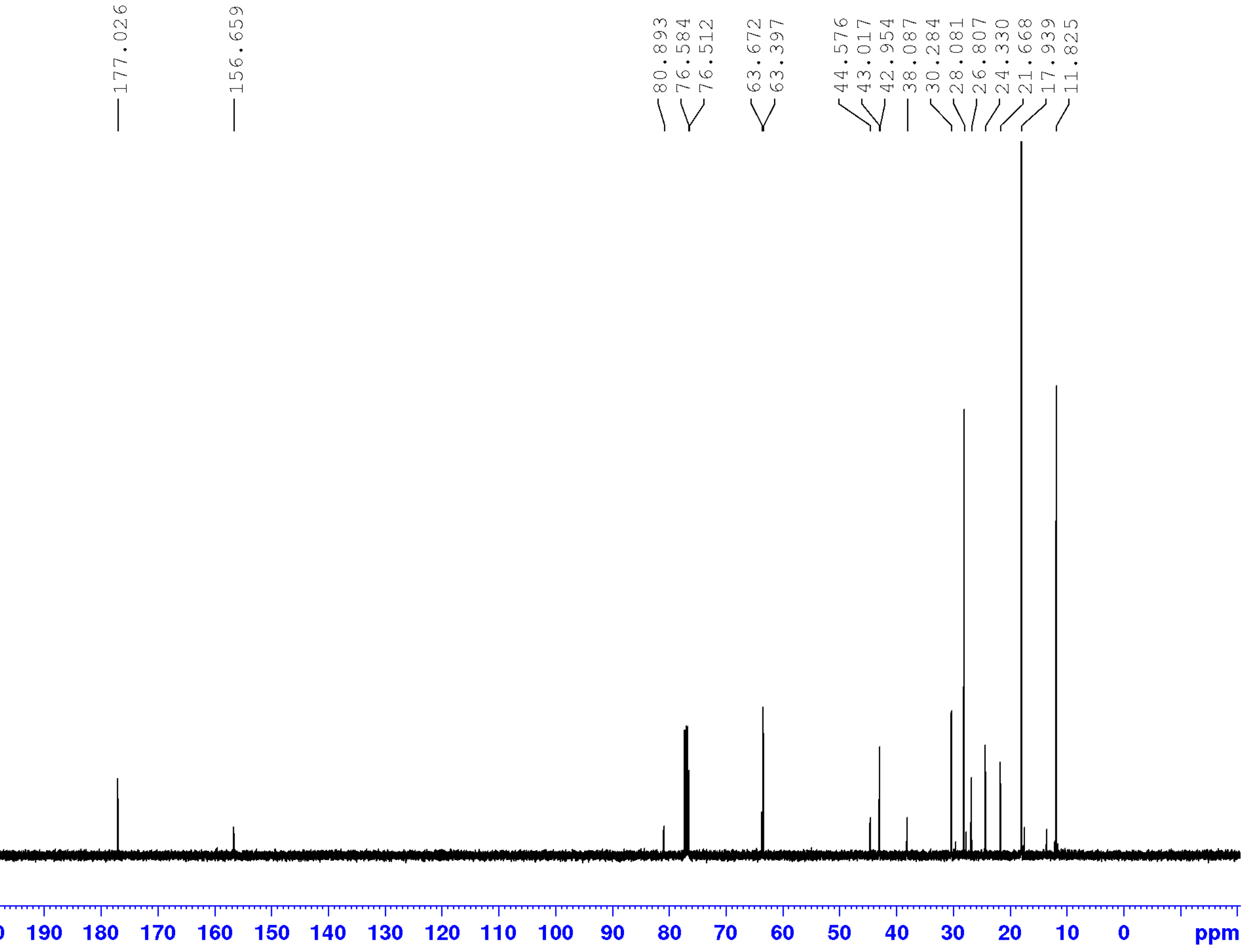

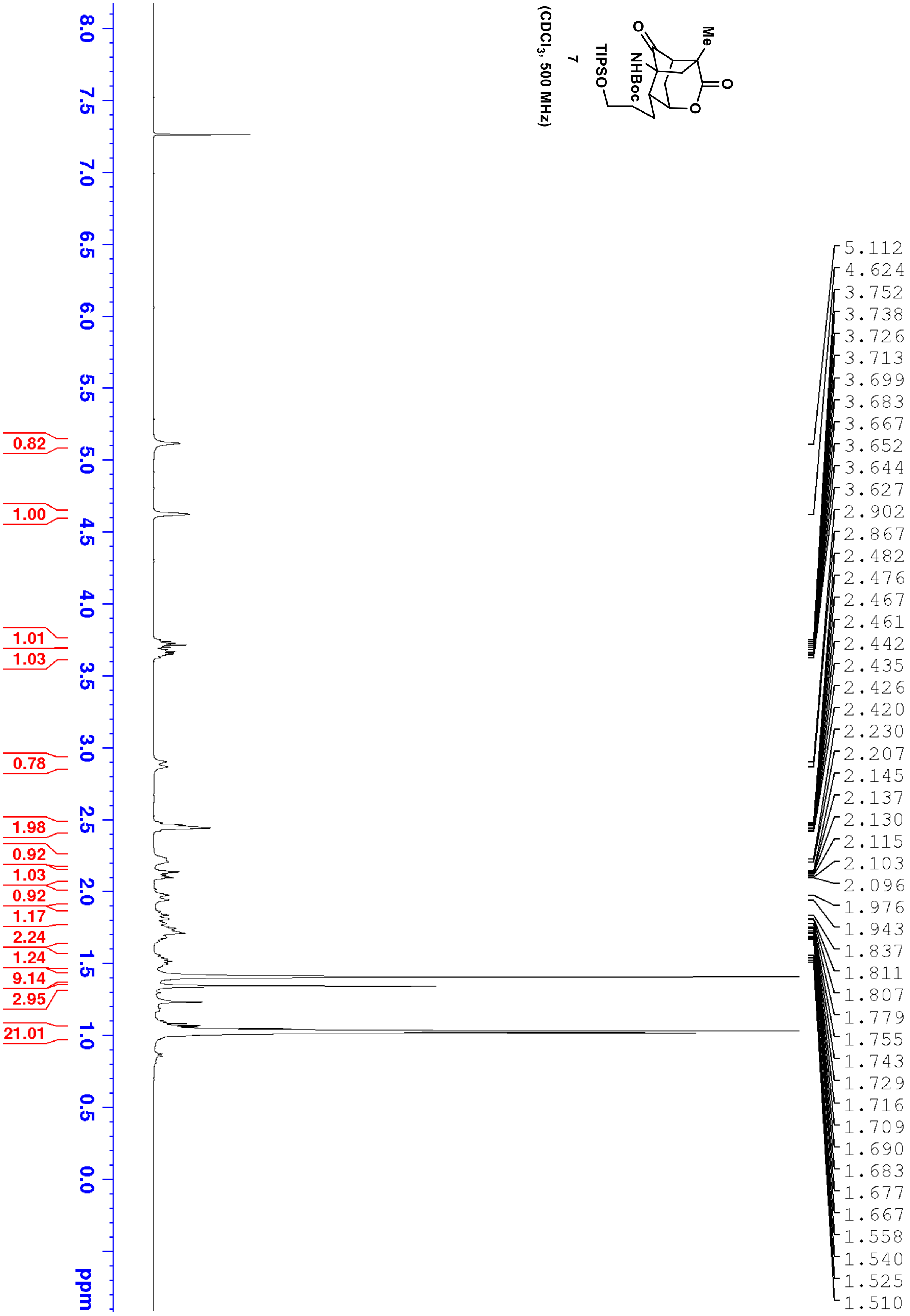
Me
O
O
O
NHBoc
TIPSO
7
(CDCl3, 500 MHz)
5.112
4.624
3.752
3.738
3.726
3.713
3.699
3.683
3.667
3.652
3.644
3.627
2.902
2.867
2.482
2.476
2.467
2.461
2.442
2.435
2.426
2.420
2.230
2.207
2.145
2.137
2.130
2.115
2.103
2.096
1.976
1.943
1.837
1.811
1.807
1.779
1.755
1.743
1.729
1.716
1.709
1.690
1.683
1.677
1.667
1.558
1.540
1.525
1.510
0.82
1.00
1.01
1.03
0.78
1.98
0.92
1.03
0.92
1.17
2.24
1.24
9.14
2.95
21.01
8.0
7.5
7.0
6.5
6.0
5.5
5.0
4.5
4.0
3.5
3.0
2.5
2.0
1.5
1.0
0.5
0.0
ppm

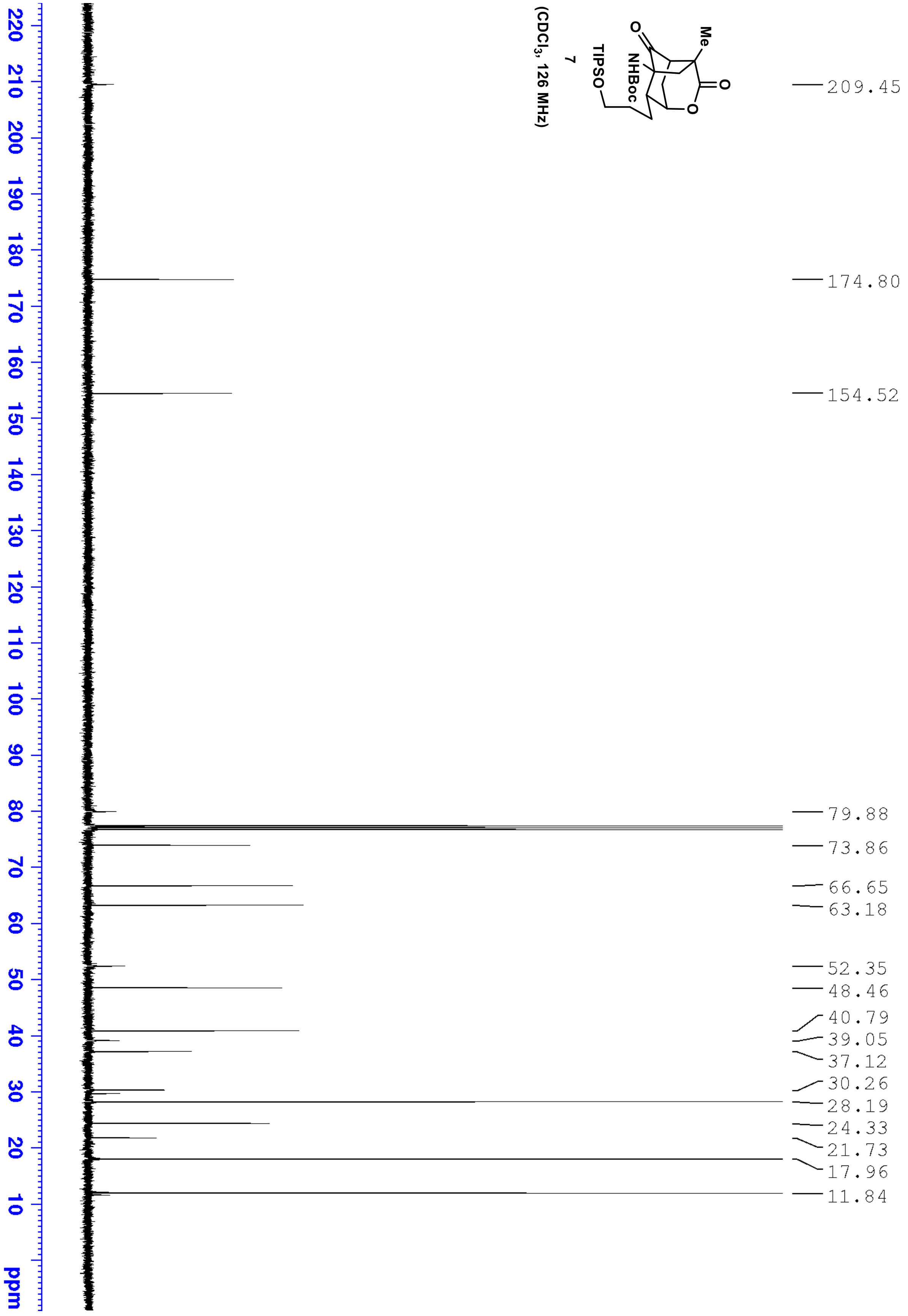

7
(CDCl$_3$, 126 MHz)
Me
O
NHBoc
TIPSO
209.45
174.80
154.52
79.88
73.86
66.65
63.18
52.35
48.46
40.79
39.05
37.12
30.26
28.19
24.33
21.73
17.96
11.84
220 210 200 190 180 170 160 150 140 130 120 110 100 90 80 70 60 50 40 30 20 10 ppm

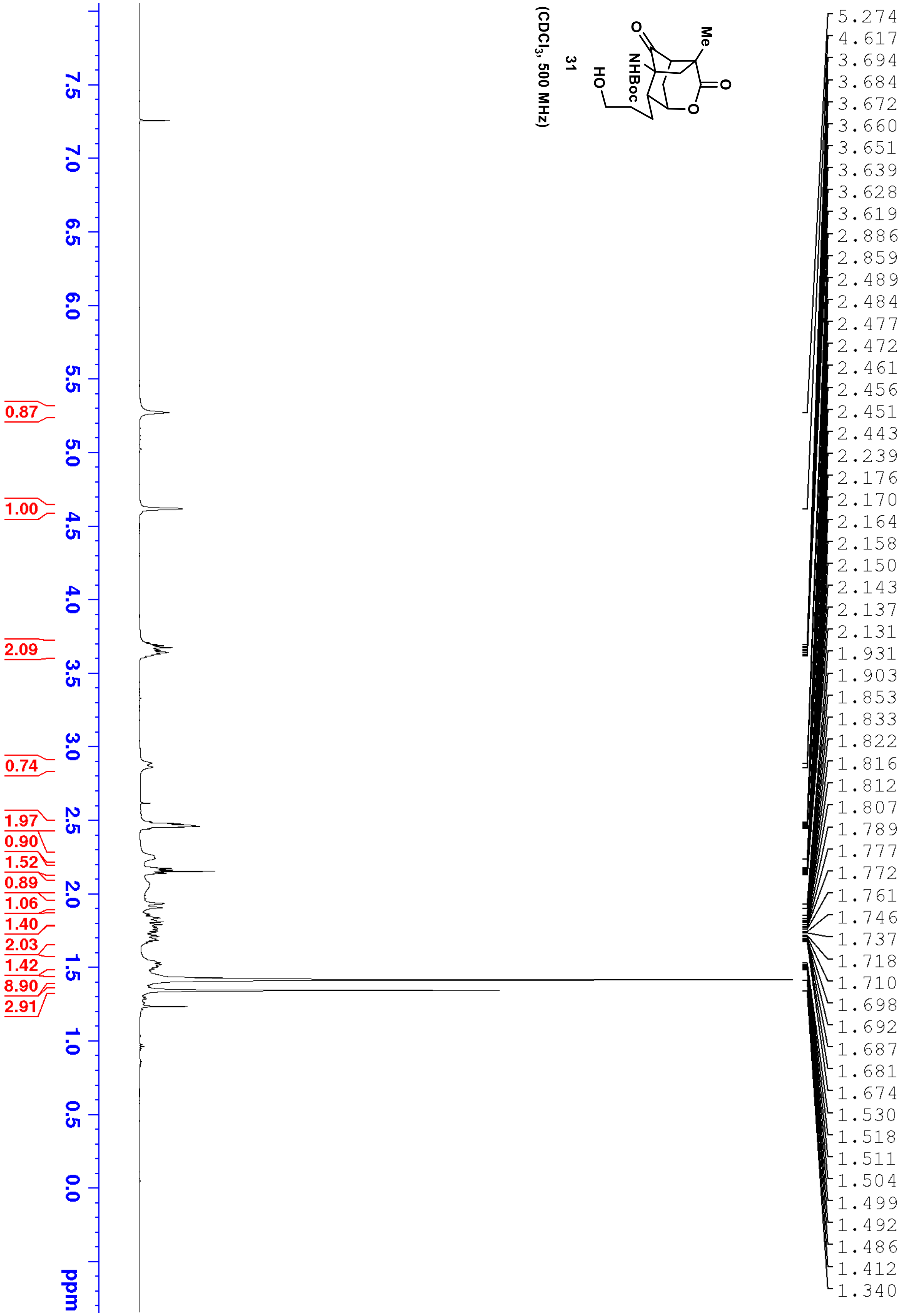
Me
O
O
O
NHBoc
HO
31
(CDCl3, 500 MHz)
5.274
4.617
3.694
3.684
3.672
3.660
3.651
3.639
3.628
3.619
2.886
2.859
2.489
2.484
2.477
2.472
2.461
2.456
2.451
2.443
2.239
2.176
2.170
2.164
2.158
2.150
2.143
2.137
2.131
1.931
1.903
1.853
1.833
1.822
1.816
1.812
1.807
1.789
1.777
1.772
1.761
1.746
1.737
1.718
1.710
1.698
1.692
1.687
1.681
1.674
1.530
1.518
1.511
1.504
1.499
1.492
1.486
1.412
1.340
0.87
1.00
2.09
0.74
1.97
0.90
1.52
0.89
1.06
1.40
2.03
1.42
8.90
2.91
7.5
7.0
6.5
6.0
5.5
5.0
4.5
4.0
3.5
3.0
2.5
2.0
1.5
1.0
0.5
0.0
ppm

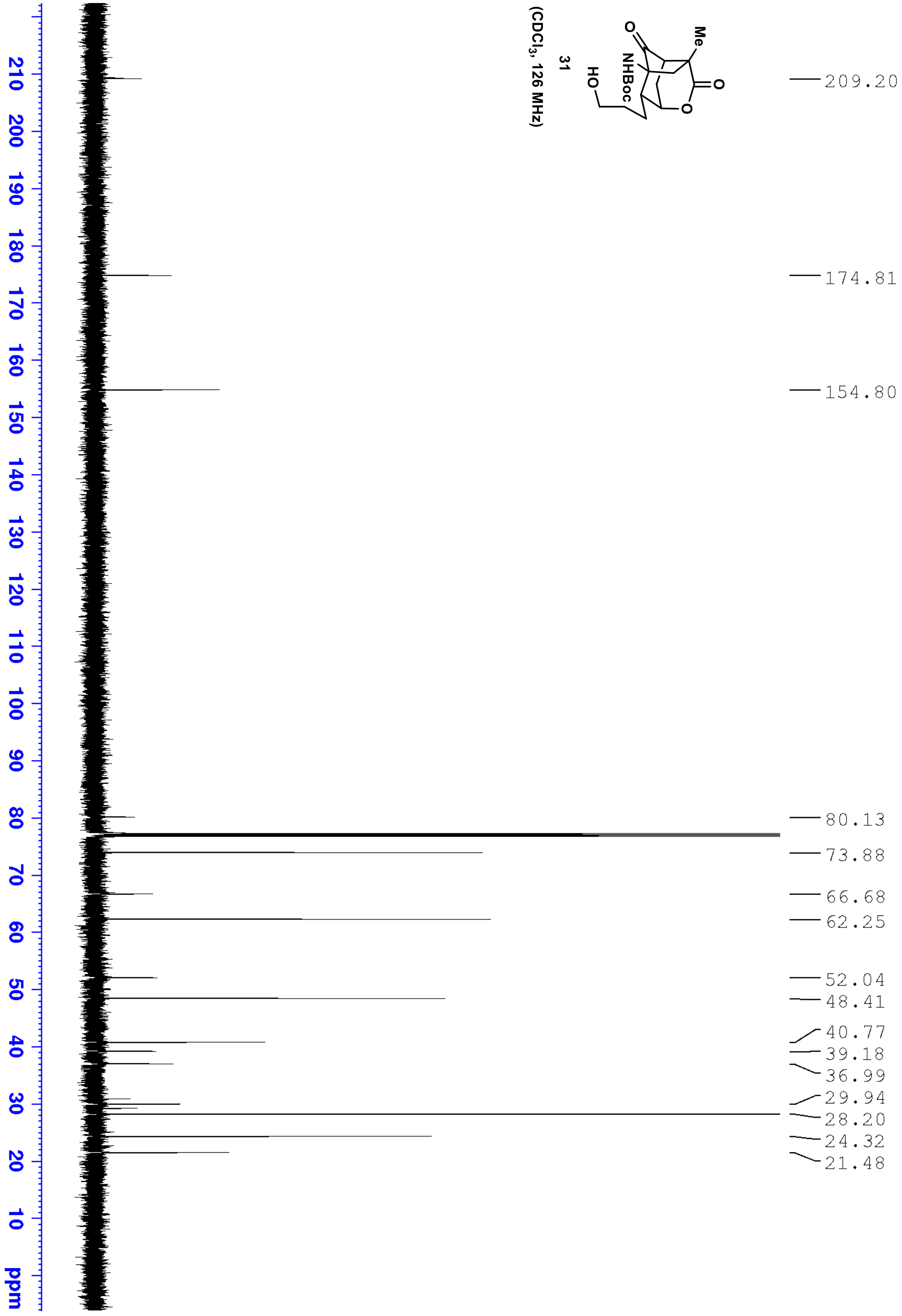
Me
O
O
O
NHBoc
HO
31
(CDCl3, 126 MHz)
209.20
174.81
154.80
80.13
73.88
66.68
62.25
52.04
48.41
40.77
39.18
36.99
29.94
28.20
24.32
21.48
210
200
190
180
170
160
150
140
130
120
110
100
90
80
70
60
50
40
30
20
10
ppm

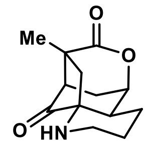


**32**

**(CDCl$_3$, 500 MHz)**

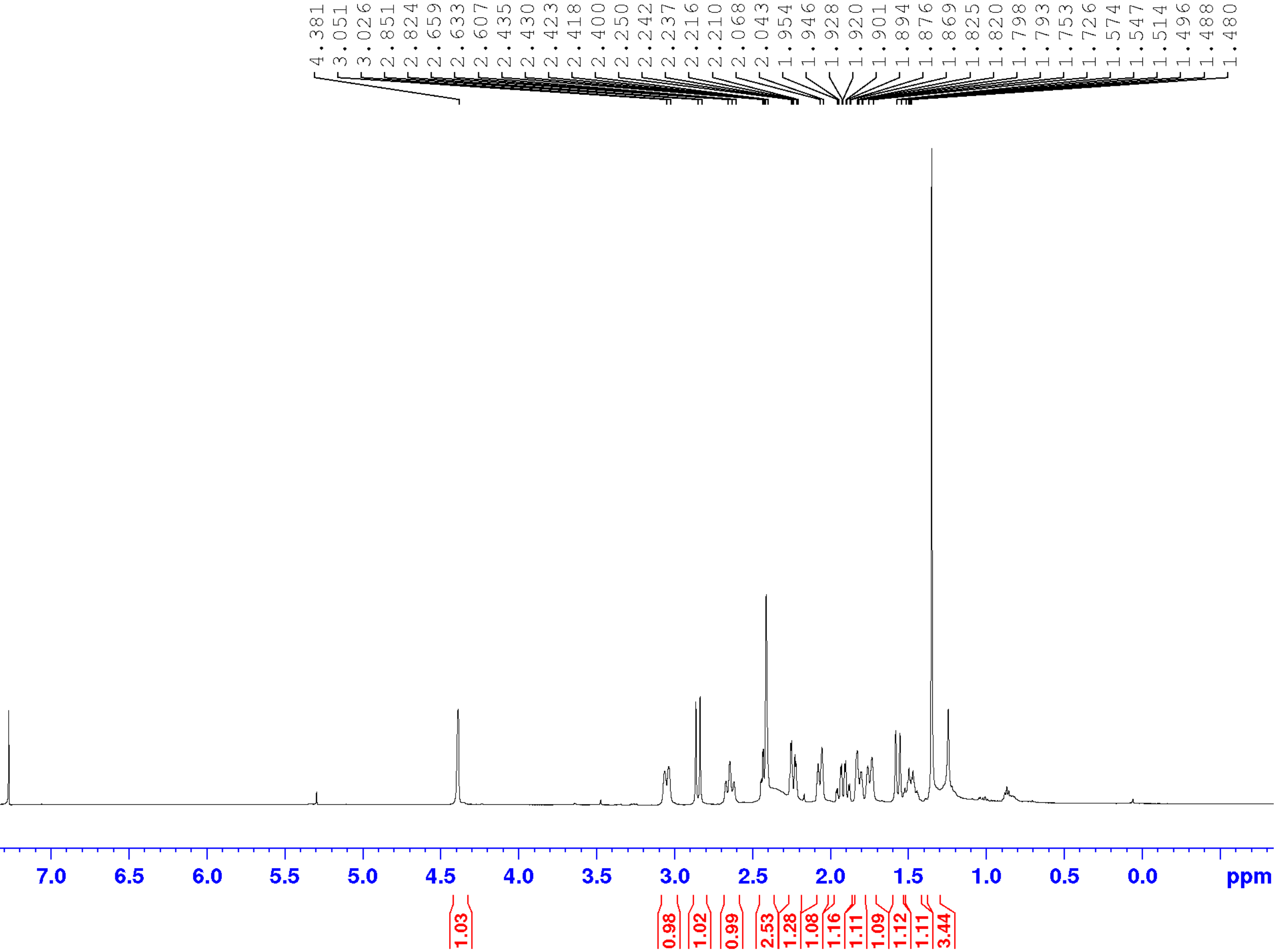

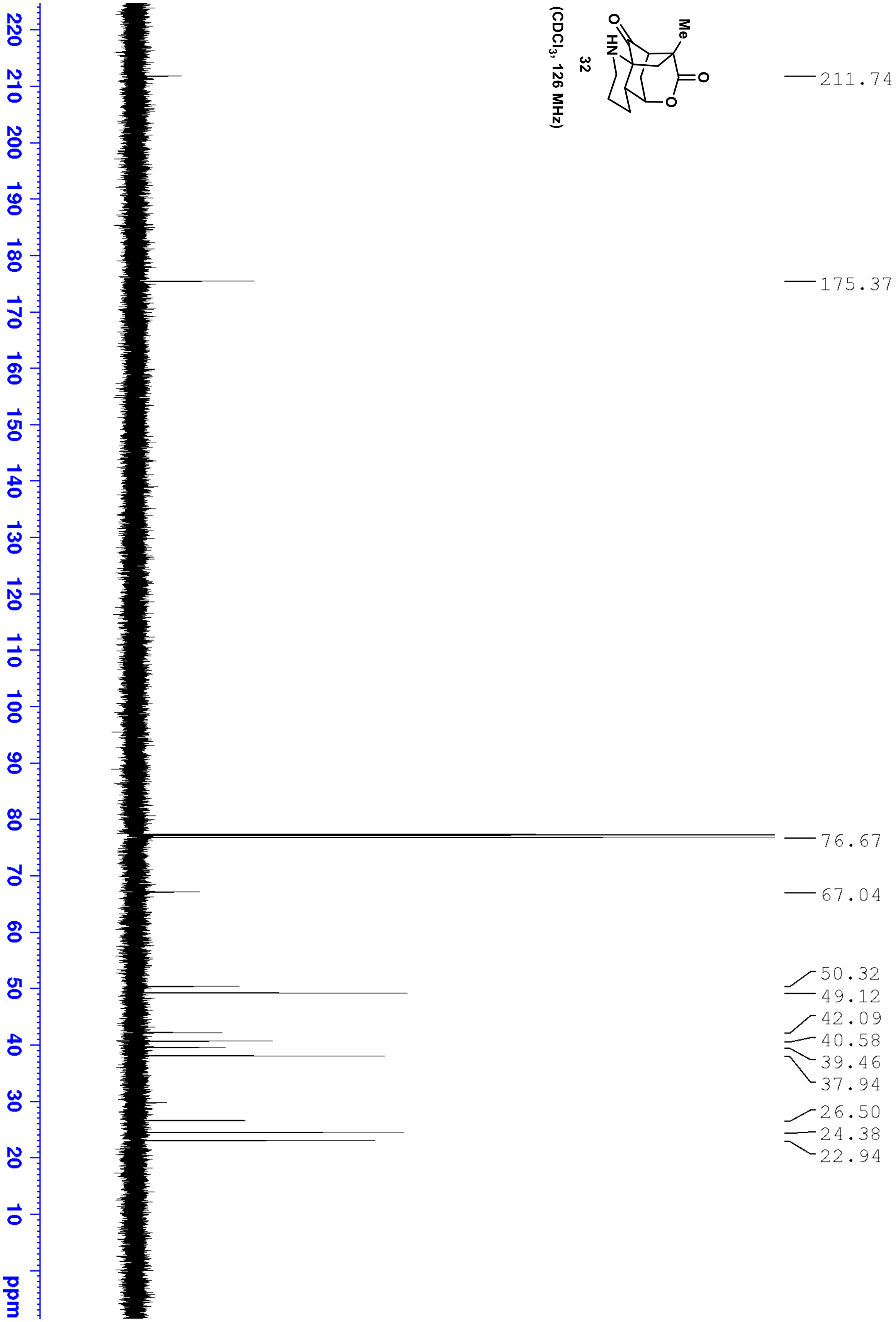
Me
O
O
HN
O
O
32
(CDCl3, 126 MHz)
211.74
175.37
76.67
67.04
50.32
49.12
42.09
40.58
39.46
37.94
26.50
24.38
22.94
220 210 200 190 180 170 160 150 140 130 120 110 100 90 80 70 60 50 40 30 20 10 ppm

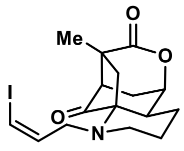

S9

($CDCl_3$, 500 MHz)

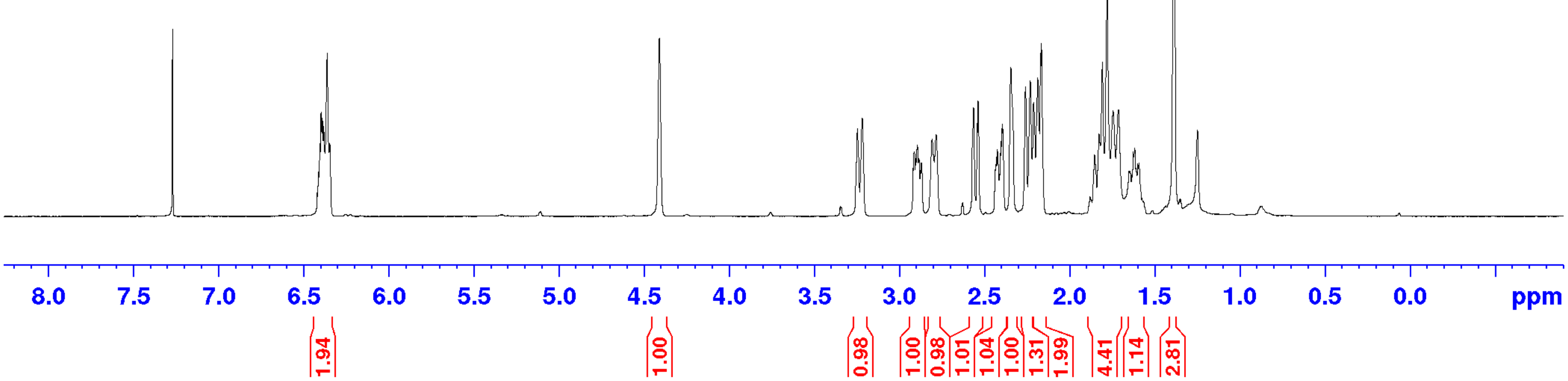

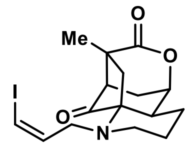


S9

($CDCl_3$, 126 MHz)

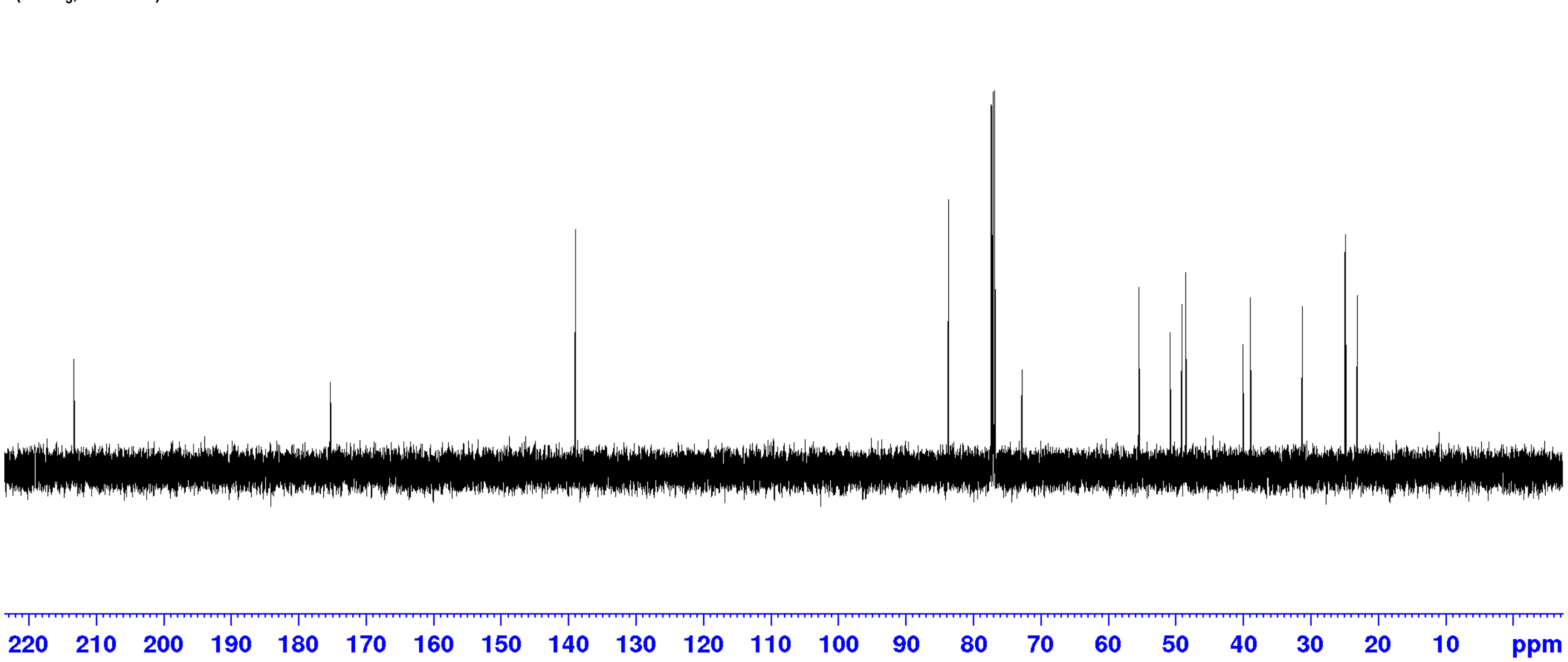

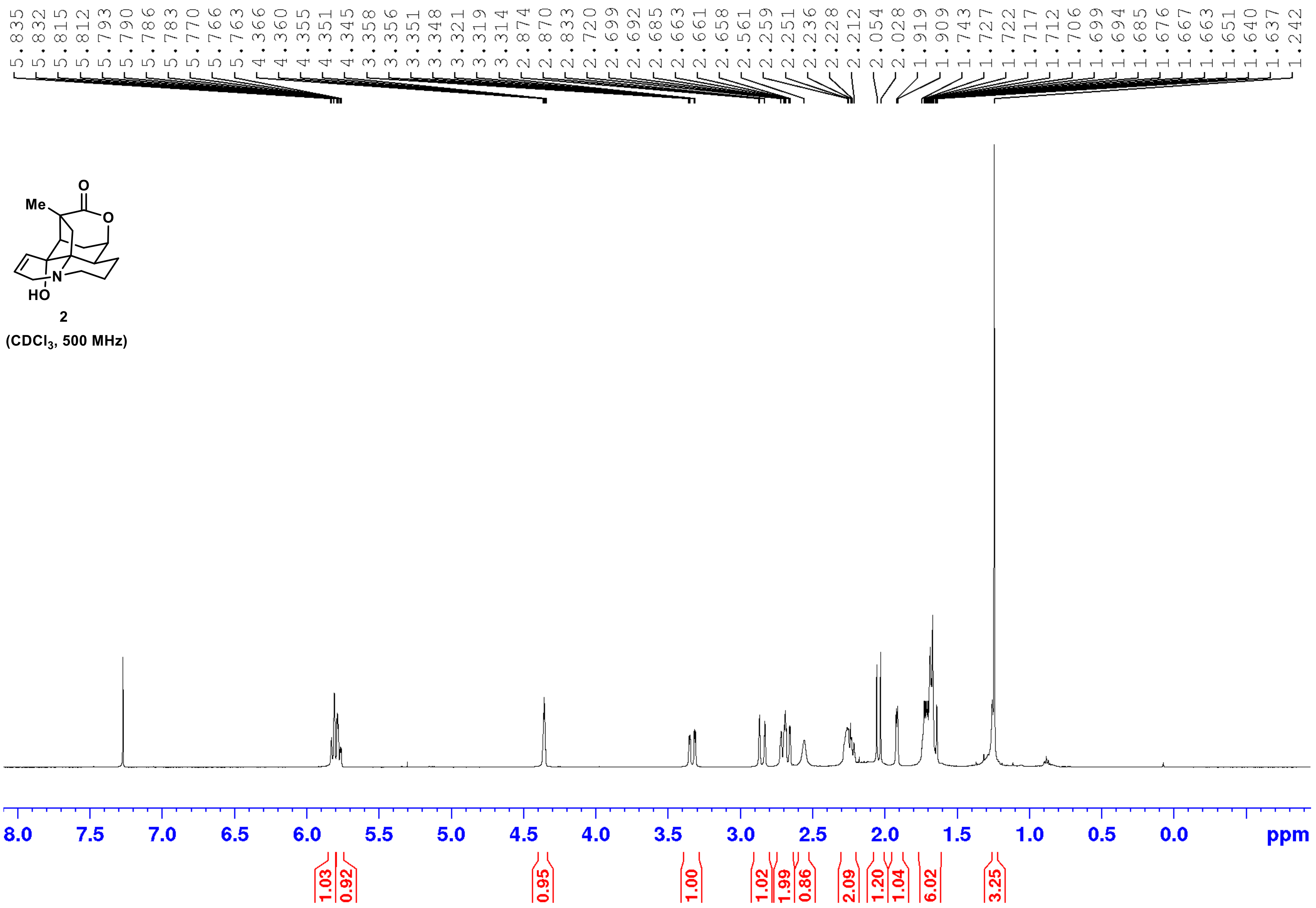
5.835
5.832
5.815
5.812
5.793
5.790
5.786
5.783
5.770
5.766
5.763
4.366
4.360
4.355
4.351
4.345
3.358
3.356
3.351
3.348
3.321
3.319
3.314
2.874
2.870
2.833
2.720
2.699
2.692
2.685
2.663
2.661
2.658
2.561
2.259
2.251
2.236
2.228
2.212
2.054
2.028
1.919
1.909
1.743
1.727
1.722
1.717
1.712
1.706
1.699
1.694
1.685
1.676
1.667
1.663
1.651
1.640
1.637
1.242
Me
O
O
N
HO
2
(CDCl3, 500 MHz)
8.0
7.5
7.0
6.5
6.0
5.5
5.0
4.5
4.0
3.5
3.0
2.5
2.0
1.5
1.0
0.5
0.0
ppm
1.03
0.92
0.95
1.00
1.02
1.99
0.86
2.09
1.20
1.04
6.02
3.25

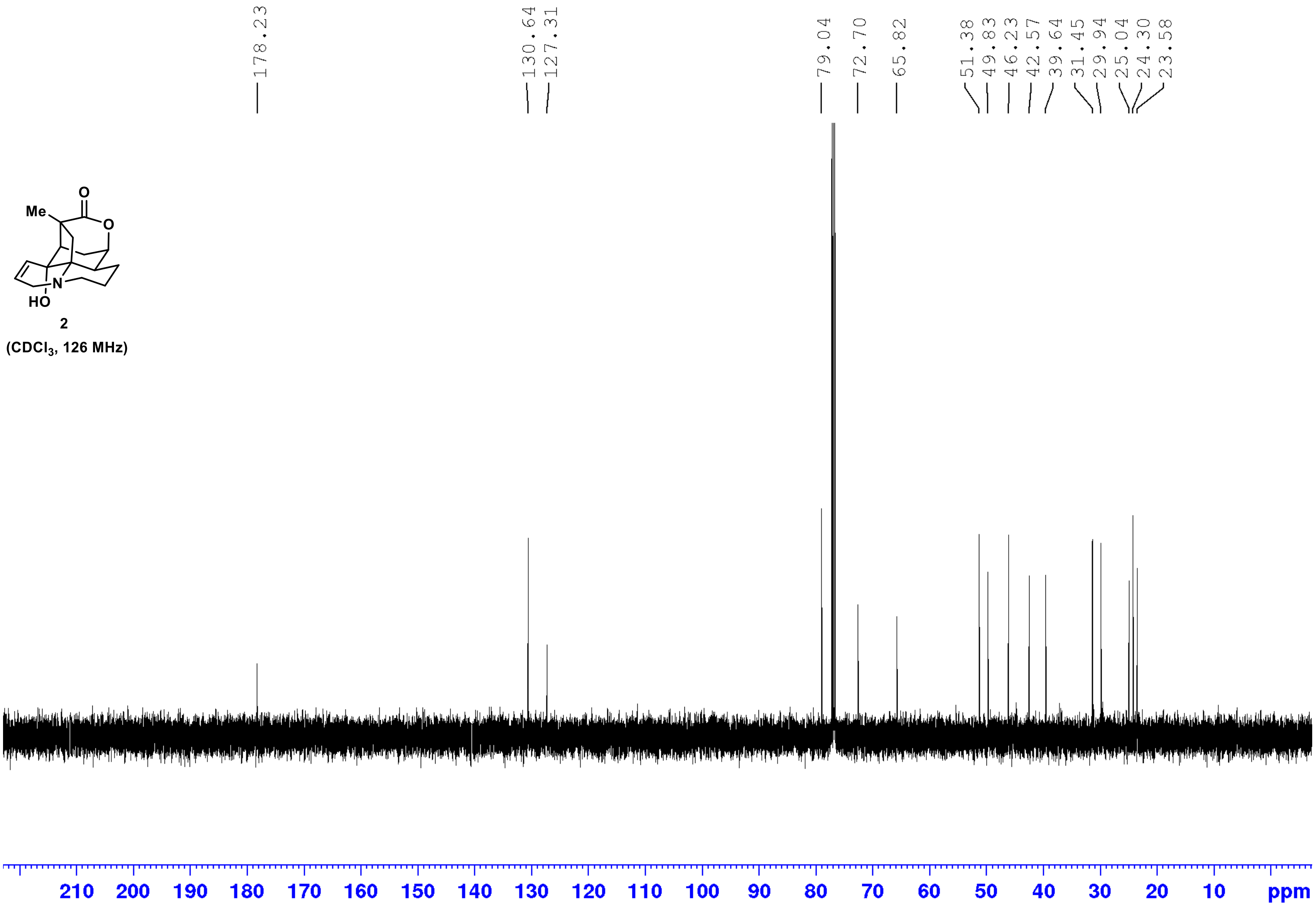

Me
O
O
N
HO
2
(CDCl$_3$, 126 MHz)
178.23
130.64
127.31
79.04
72.70
65.82
51.38
49.83
46.23
42.57
39.64
31.45
29.94
25.04
24.30
23.58
210 200 190 180 170 160 150 140 130 120 110 100 90 80 70 60 50 40 30 20 10 ppm

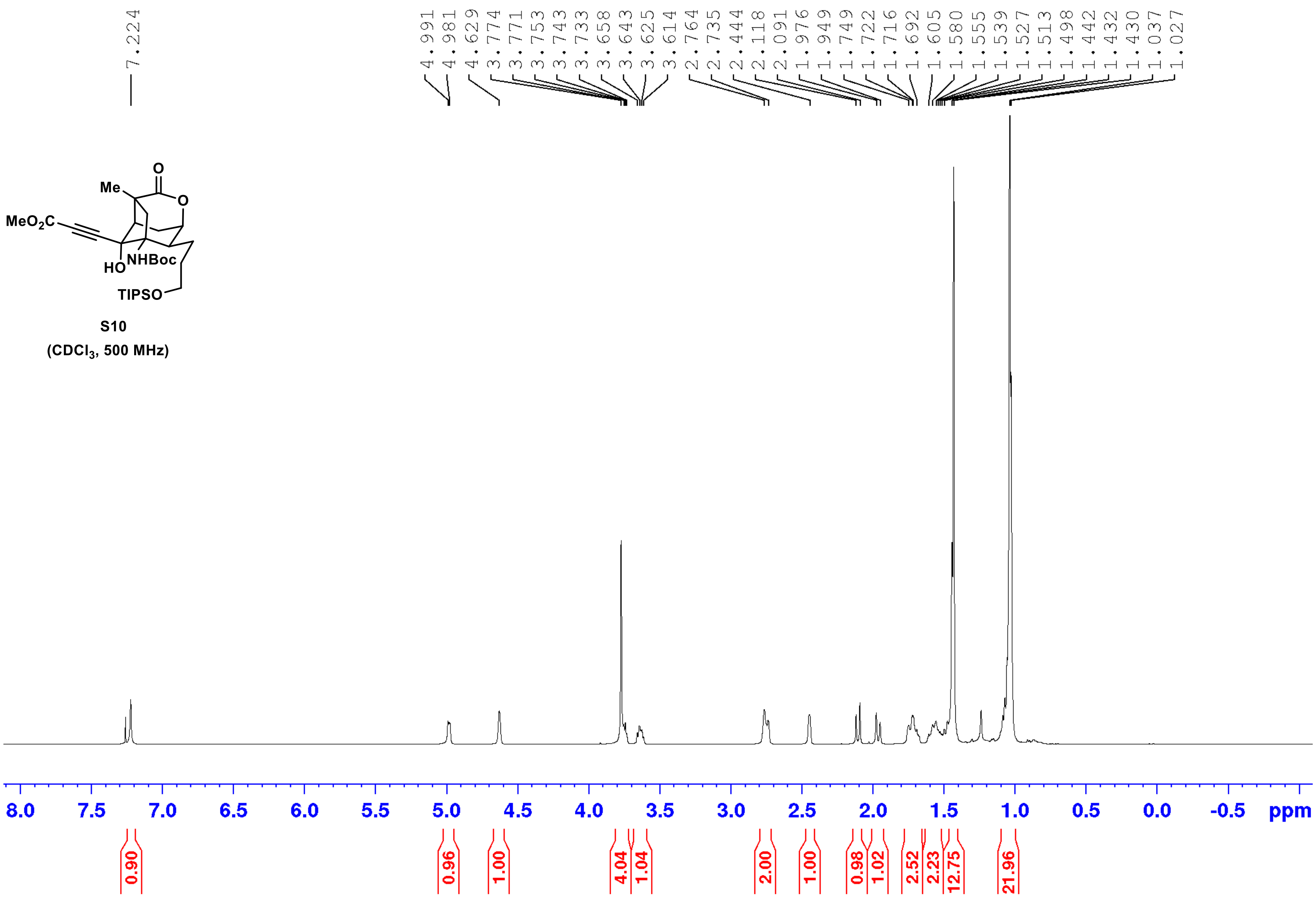


S10

($CDCl_3$, 500 MHz)

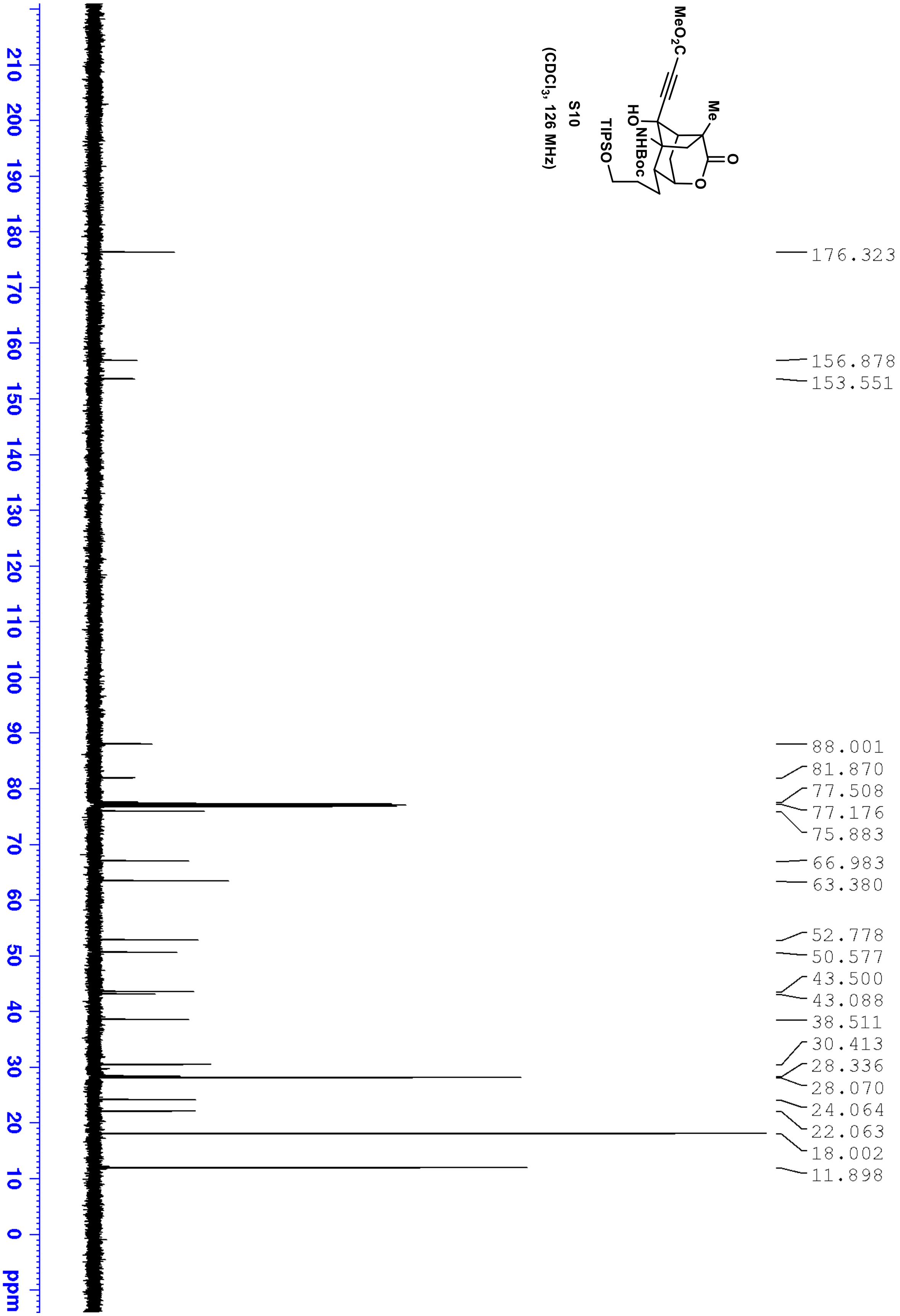
S10
(CDCl3, 126 MHz)
TIPSO
HO
NHBoc
Me
O
O
MeO2C
176.323
156.878
153.551
88.001
81.870
77.508
77.176
75.883
66.983
63.380
52.778
50.577
43.500
43.088
38.511
30.413
28.336
28.070
24.064
22.063
18.002
11.898
ppm

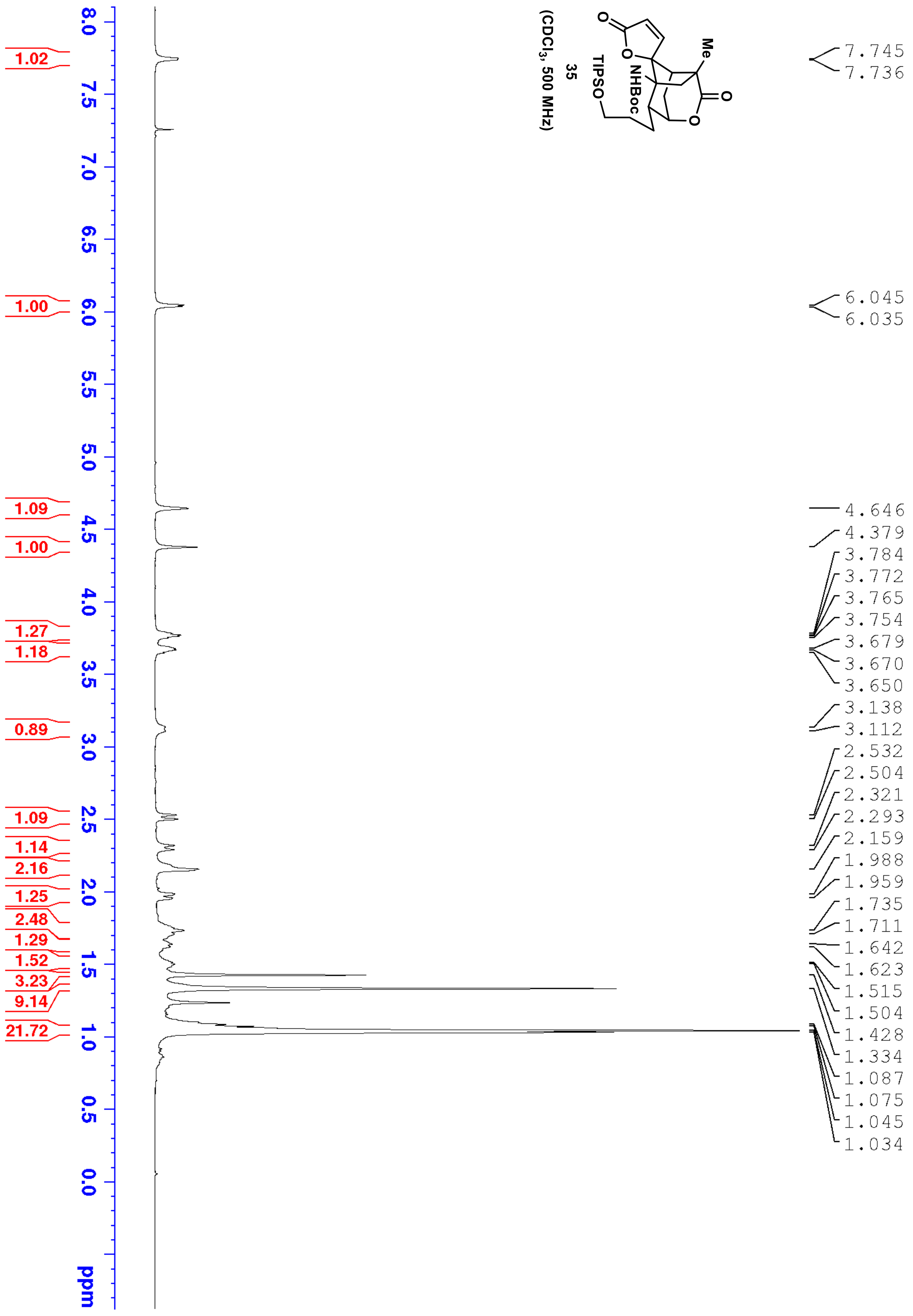
Me
O
NHBoc
TIPSO
35
(CDCl3, 500 MHz)
7.745
7.736
6.045
6.035
4.646
4.379
3.784
3.772
3.765
3.754
3.679
3.670
3.650
3.138
3.112
2.532
2.504
2.321
2.293
2.159
1.988
1.959
1.735
1.711
1.642
1.623
1.515
1.504
1.428
1.334
1.087
1.075
1.045
1.034
1.02
1.00
1.09
1.00
1.27
1.18
0.89
1.09
1.14
2.16
1.25
2.48
1.29
1.52
3.23
9.14
21.72
8.0
7.5
7.0
6.5
6.0
5.5
5.0
4.5
4.0
3.5
3.0
2.5
2.0
1.5
1.0
0.5
0.0
ppm

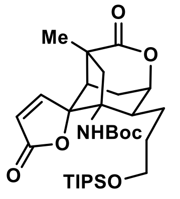


35

($CDCl_3$, 126 MHz)

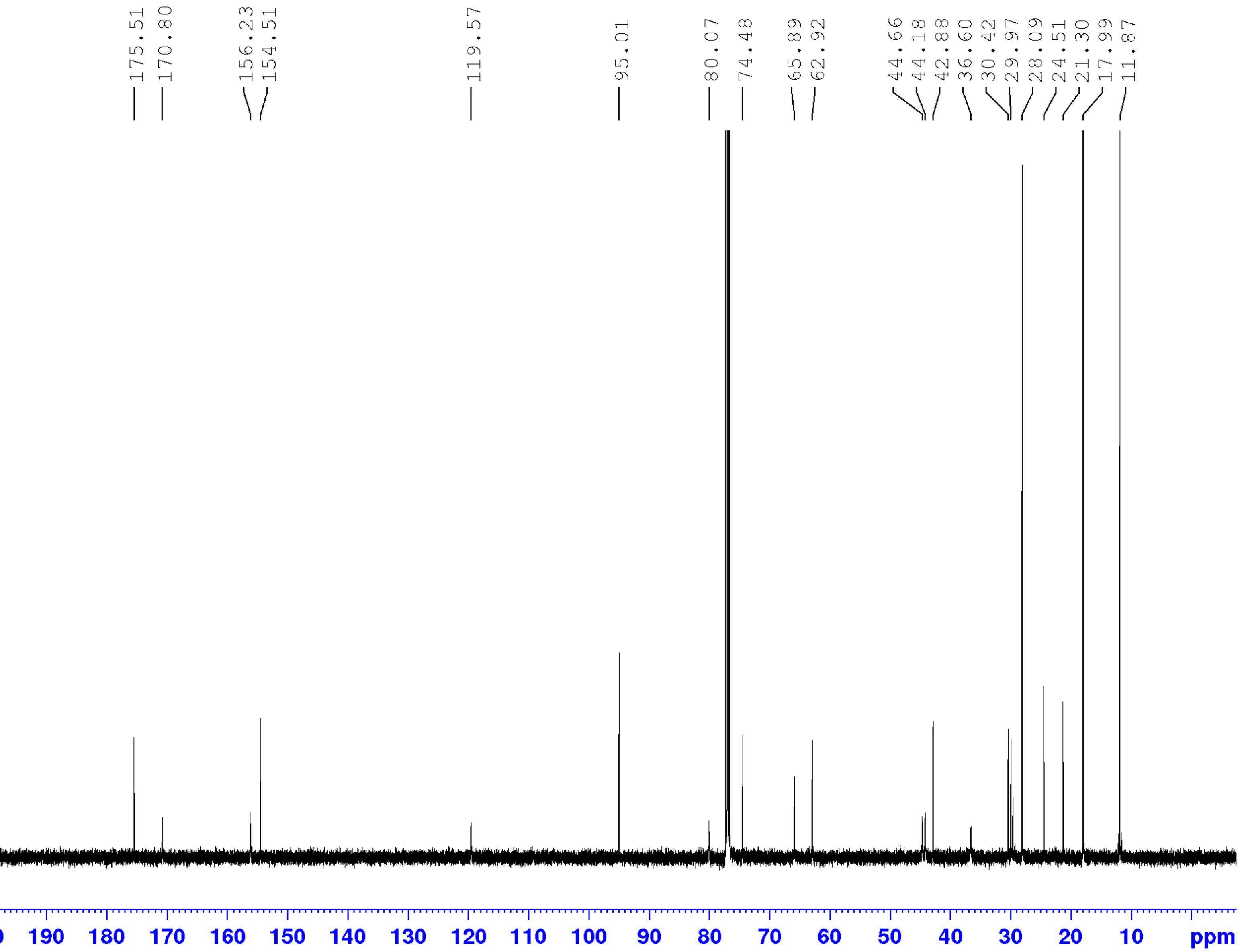

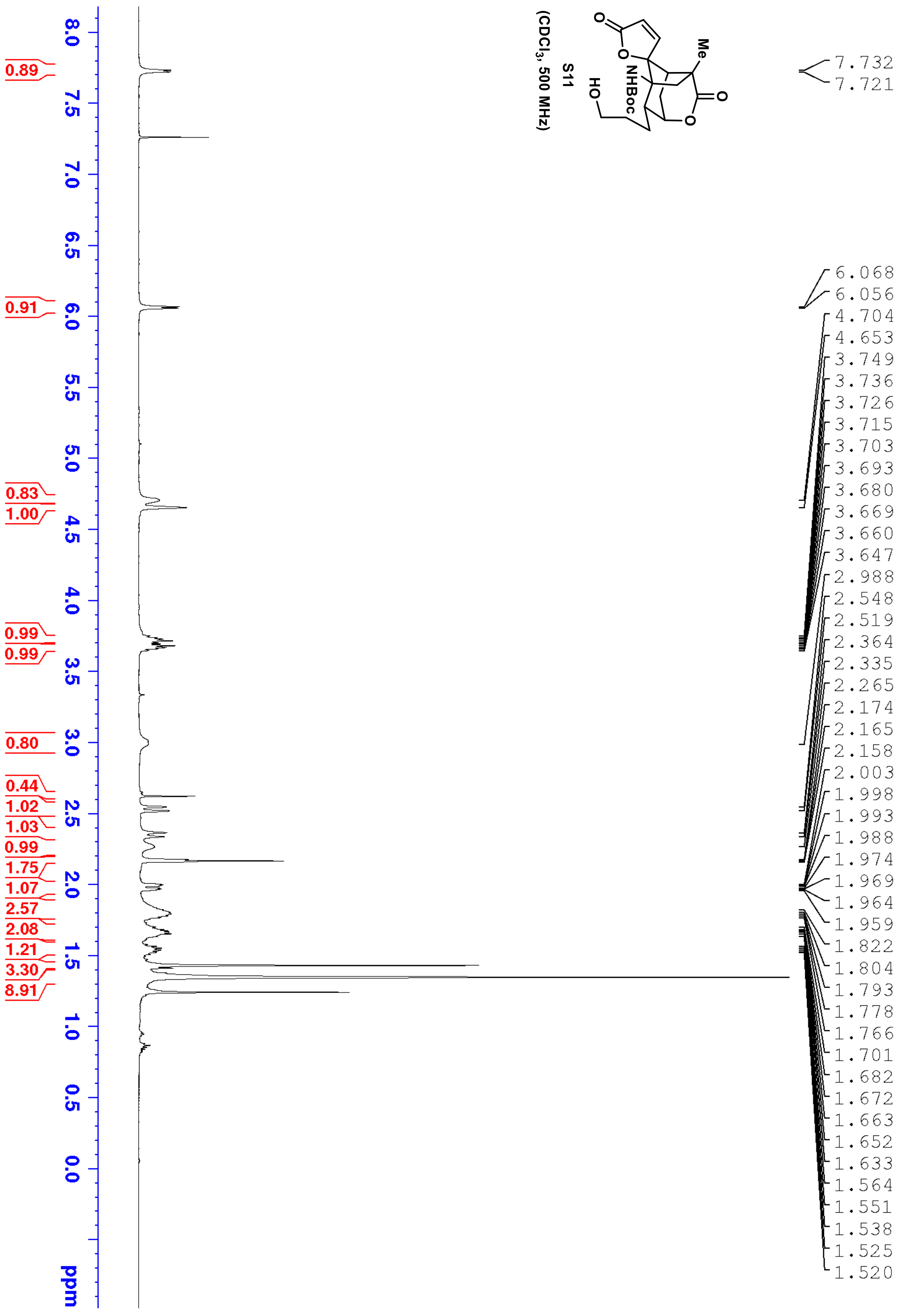
Me
O
O
O
O
NHBoc
HO
S11
(CDCl$_3$, 500 MHz)
7.732
7.721
6.068
6.056
4.704
4.653
3.749
3.736
3.726
3.715
3.703
3.693
3.680
3.669
3.660
3.647
2.988
2.548
2.519
2.364
2.335
2.265
2.174
2.165
2.158
2.003
1.998
1.993
1.988
1.974
1.969
1.964
1.959
1.822
1.804
1.793
1.778
1.766
1.701
1.682
1.672
1.663
1.652
1.633
1.564
1.551
1.538
1.525
1.520
0.89
0.91
0.83
1.00
0.99
0.99
0.80
0.44
1.02
1.03
0.99
1.75
1.07
2.57
2.08
1.21
3.30
8.91
8.0
7.5
7.0
6.5
6.0
5.5
5.0
4.5
4.0
3.5
3.0
2.5
2.0
1.5
1.0
0.5
0.0
ppm

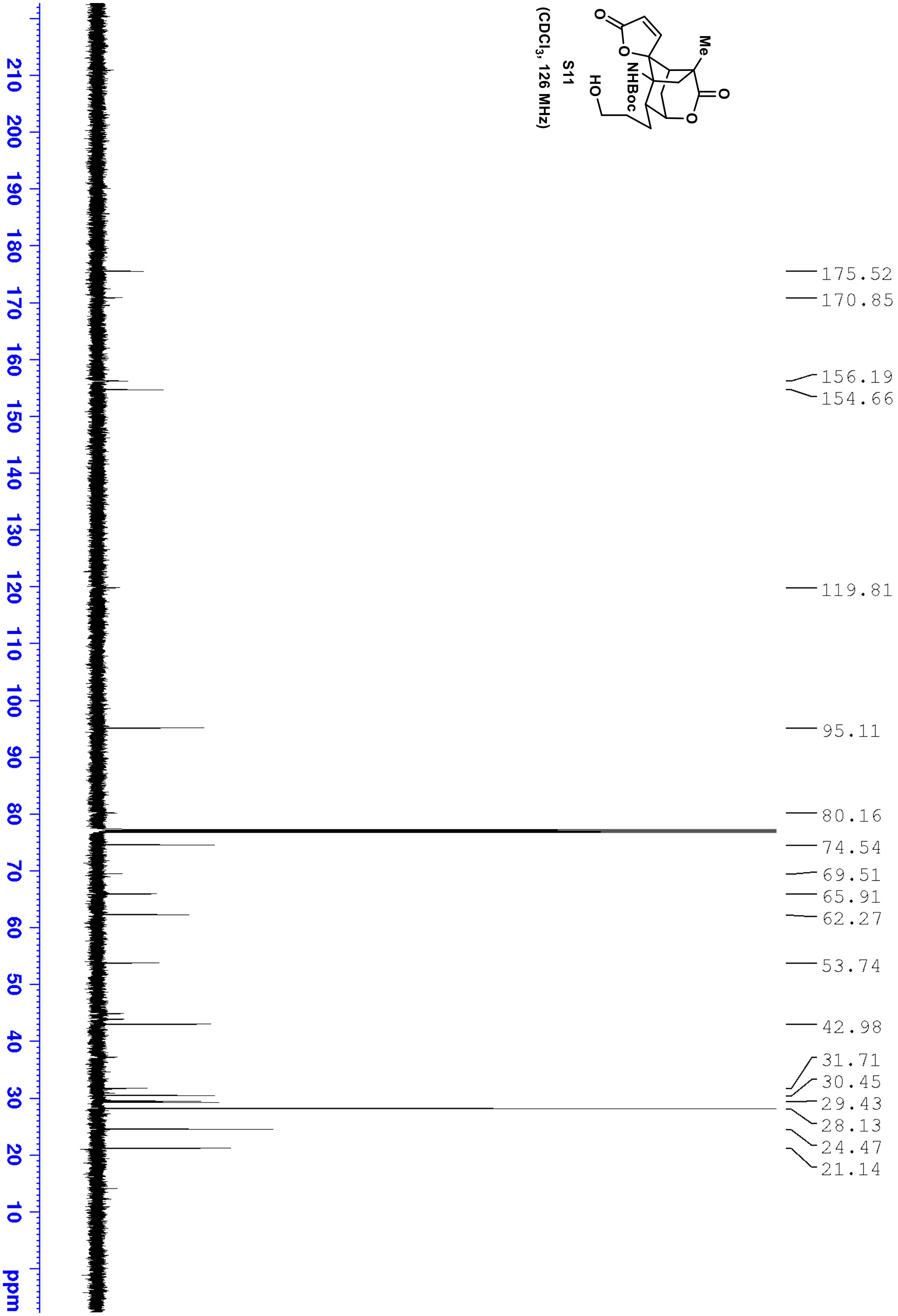
S11
(CDCl3, 126 MHz)
Me
NHBoc
HO
175.52
170.85
156.19
154.66
119.81
95.11
80.16
74.54
69.51
65.91
62.27
53.74
42.98
31.71
30.45
29.43
28.13
24.47
21.14
ppm

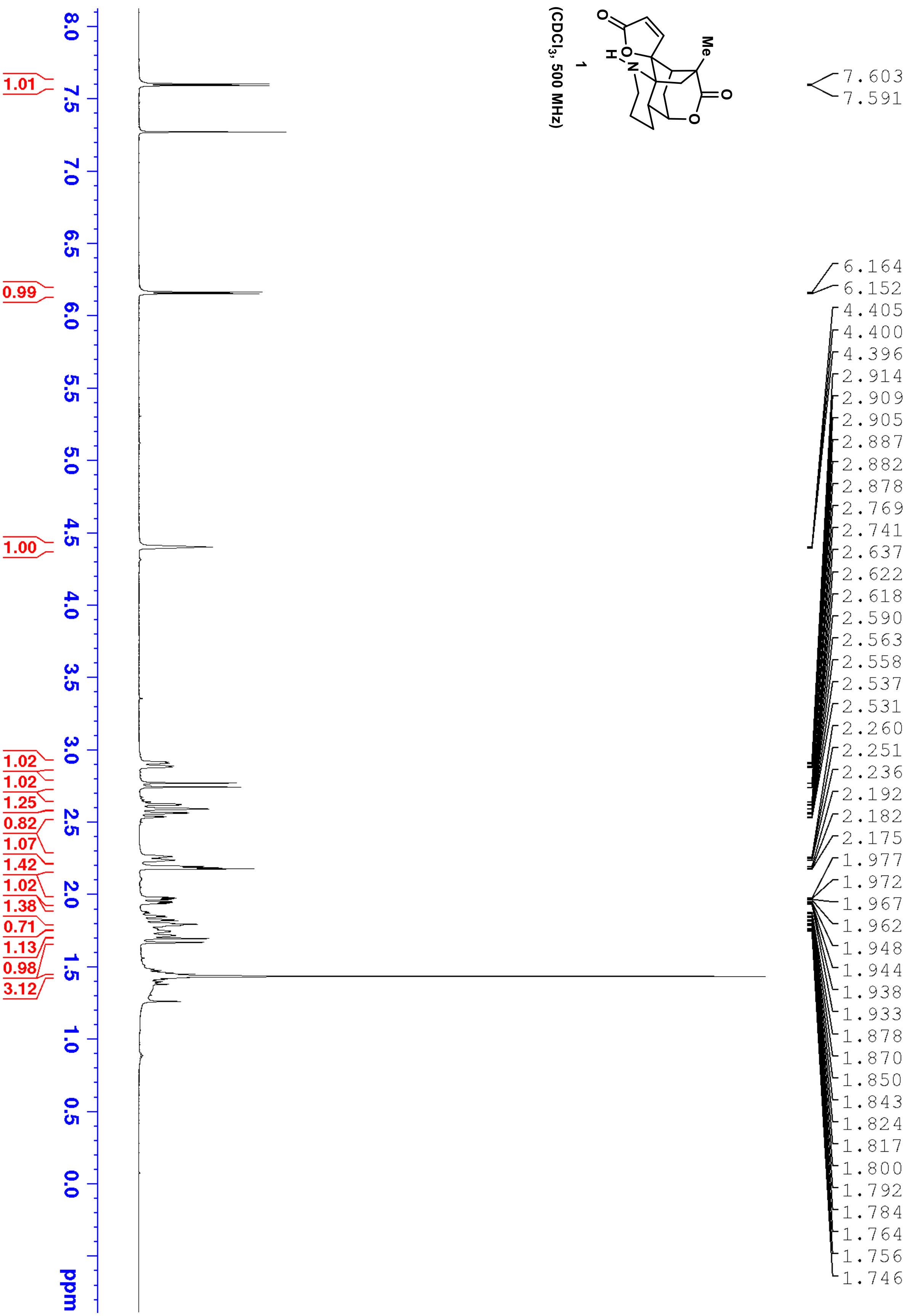
Me
O
O
H
N
O
O
1
(CDCl3, 500 MHz)
7.603
7.591
6.164
6.152
4.405
4.400
4.396
2.914
2.909
2.905
2.887
2.882
2.878
2.769
2.741
2.637
2.622
2.618
2.590
2.563
2.558
2.537
2.531
2.260
2.251
2.236
2.192
2.182
2.175
1.977
1.972
1.967
1.962
1.948
1.944
1.938
1.933
1.878
1.870
1.850
1.843
1.824
1.817
1.800
1.792
1.784
1.764
1.756
1.746
1.01
0.99
1.00
1.02
1.02
1.25
0.82
1.07
1.42
1.02
1.38
0.71
1.13
0.98
3.12
8.0
7.5
7.0
6.5
6.0
5.5
5.0
4.5
4.0
3.5
3.0
2.5
2.0
1.5
1.0
0.5
0.0
ppm

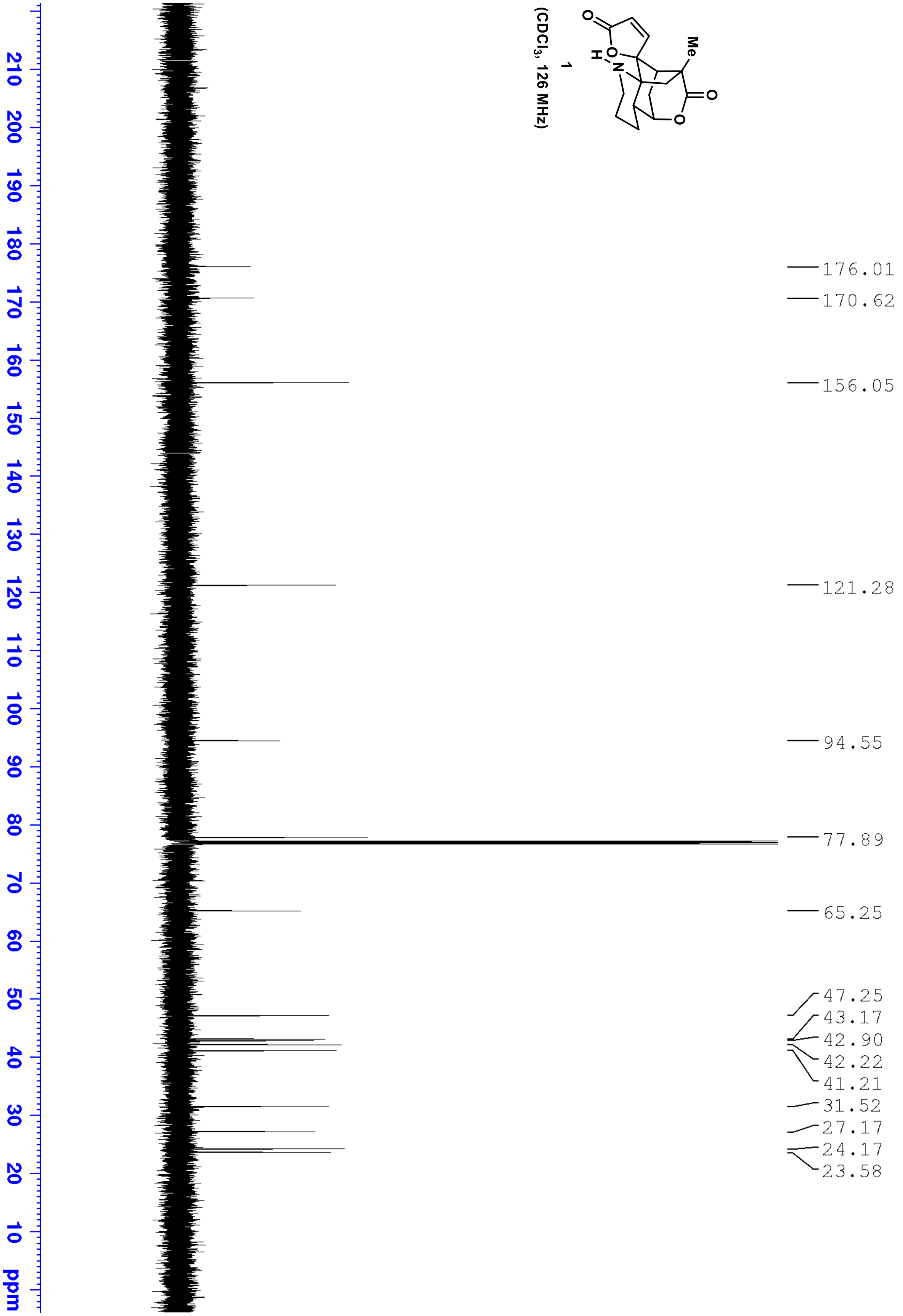
Me
O
O
O
O
H
N
1
(CDCl3, 126 MHz)
176.01
170.62
156.05
121.28
94.55
77.89
65.25
47.25
43.17
42.90
42.22
41.21
31.52
27.17
24.17
23.58
210
200
190
180
170
160
150
140
130
120
110
100
90
80
70
60
50
40
30
20
10
ppm

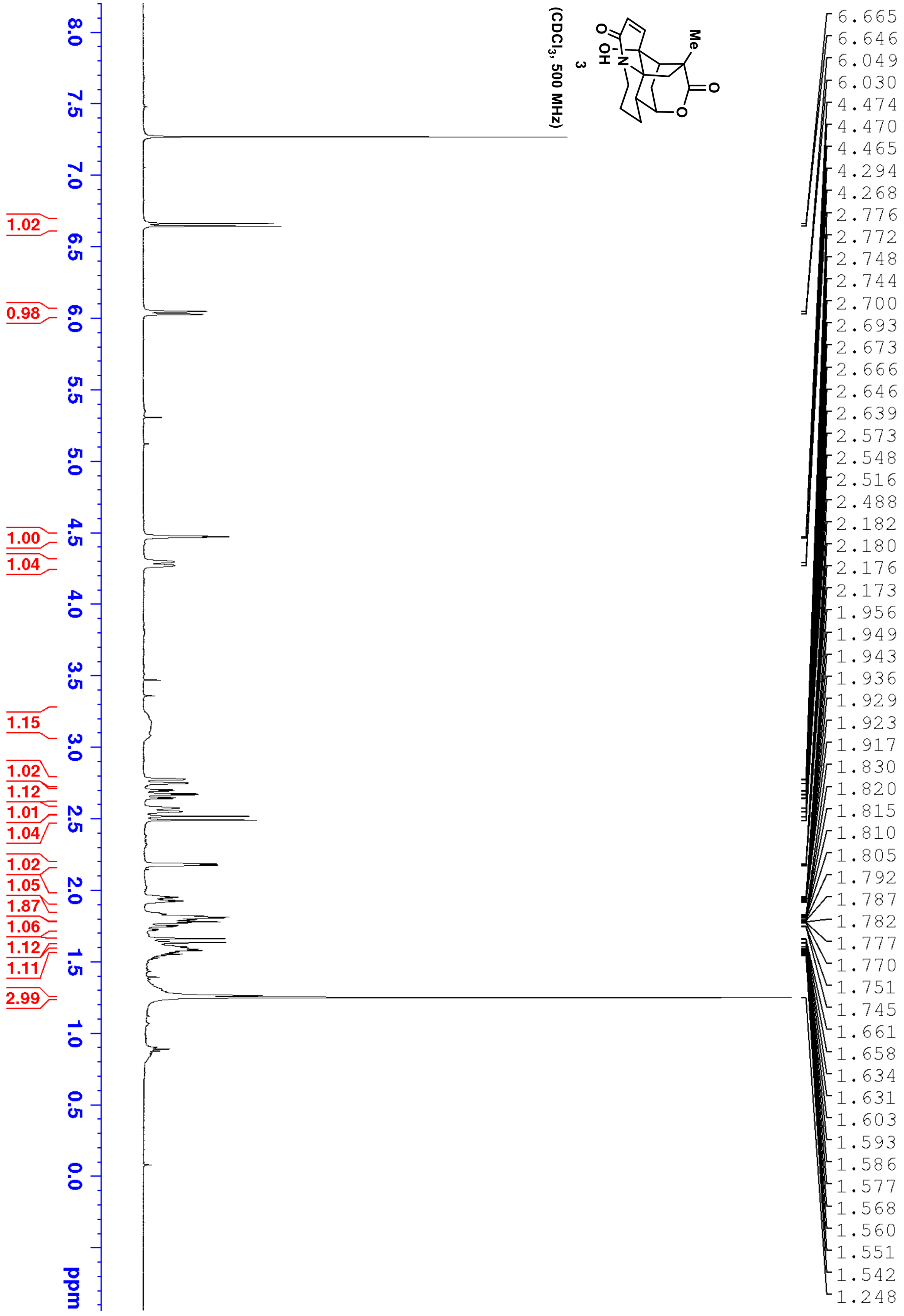
Me
O
O
N
O OH
3
(CDCl3, 500 MHz)
6.665
6.646
6.049
6.030
4.474
4.470
4.465
4.294
4.268
2.776
2.772
2.748
2.744
2.700
2.693
2.673
2.666
2.646
2.639
2.573
2.548
2.516
2.488
2.182
2.180
2.176
2.173
1.956
1.949
1.943
1.936
1.929
1.923
1.917
1.830
1.820
1.815
1.810
1.805
1.792
1.787
1.782
1.777
1.770
1.751
1.745
1.661
1.658
1.634
1.631
1.603
1.593
1.586
1.577
1.568
1.560
1.551
1.542
1.248
1.02
0.98
1.00
1.04
1.15
1.02
1.12
1.01
1.04
1.02
1.05
1.87
1.06
1.12
1.11
2.99
8.0
7.5
7.0
6.5
6.0
5.5
5.0
4.5
4.0
3.5
3.0
2.5
2.0
1.5
1.0
0.5
0.0
ppm

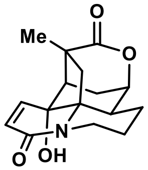


3

(CDCl$_3$, 126 MHz)

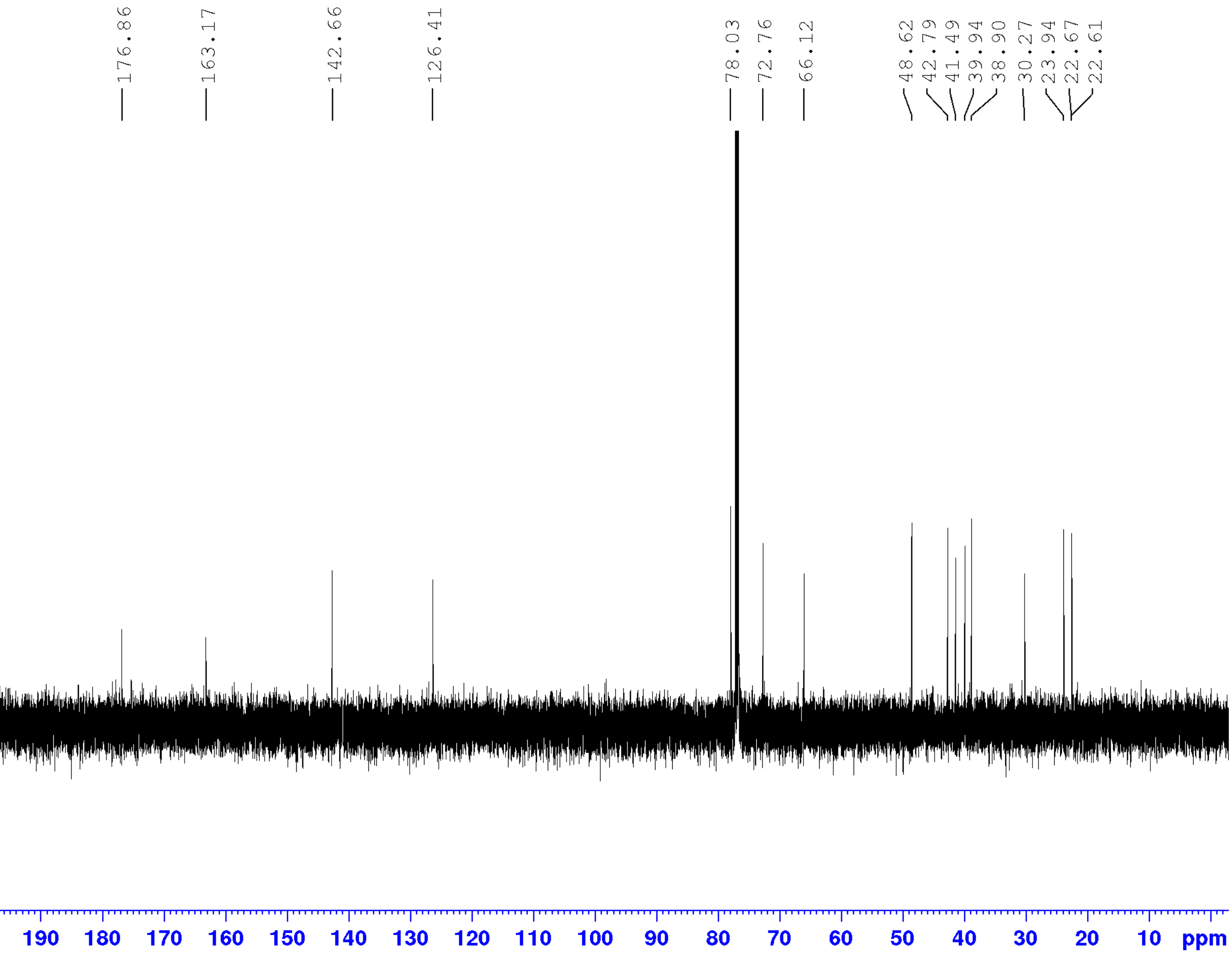

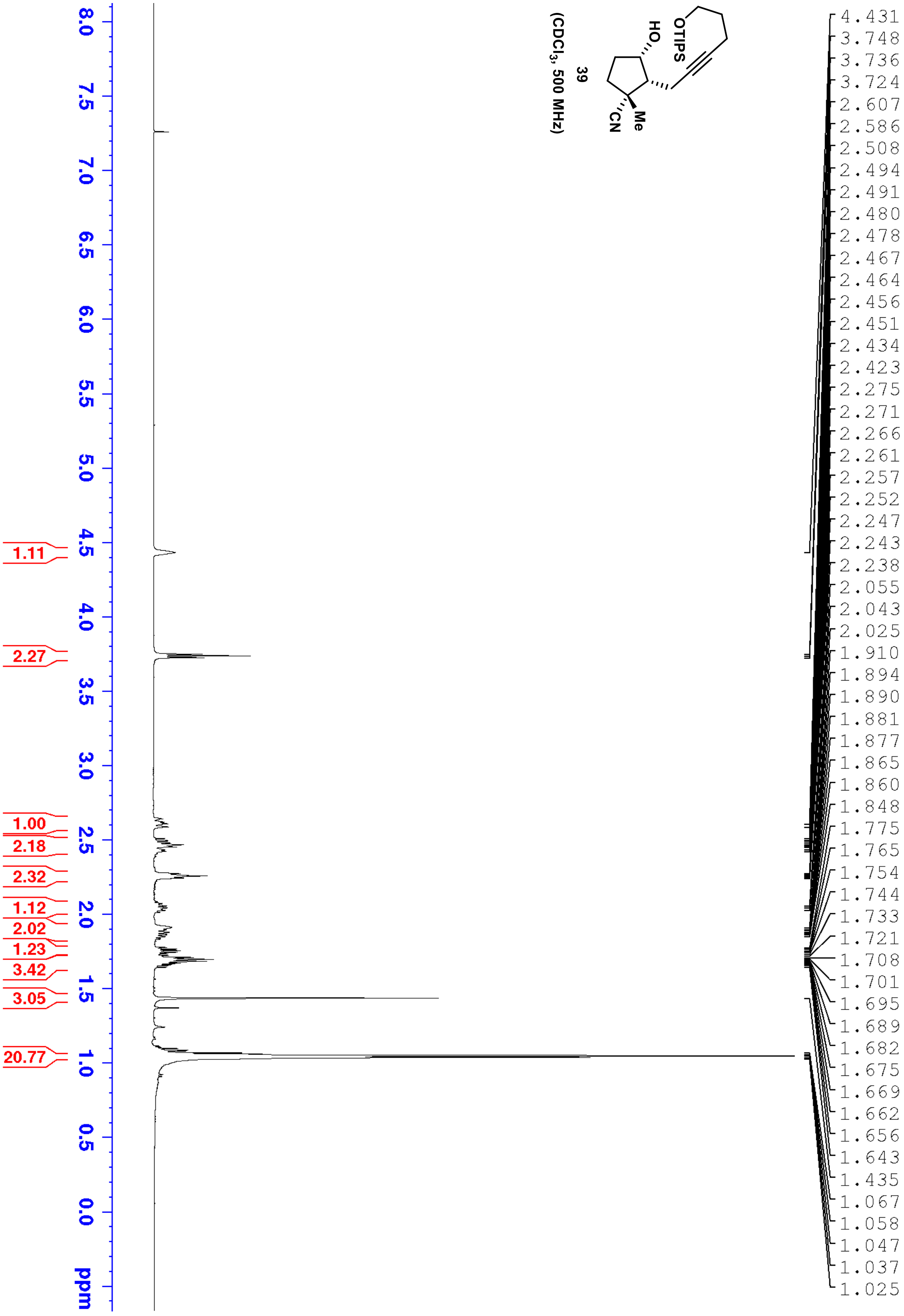
OTIPS
HO
Me
CN
39
(CDCl3, 500 MHz)
4.431
3.748
3.736
3.724
2.607
2.586
2.508
2.494
2.491
2.480
2.478
2.467
2.464
2.456
2.451
2.434
2.423
2.275
2.271
2.266
2.261
2.257
2.252
2.247
2.243
2.238
2.055
2.043
2.025
1.910
1.894
1.890
1.881
1.877
1.865
1.860
1.848
1.775
1.765
1.754
1.744
1.733
1.721
1.708
1.701
1.695
1.689
1.682
1.675
1.669
1.662
1.656
1.643
1.435
1.067
1.058
1.047
1.037
1.025
1.11
2.27
1.00
2.18
2.32
1.12
2.02
1.23
3.42
3.05
20.77
8.0
7.5
7.0
6.5
6.0
5.5
5.0
4.5
4.0
3.5
3.0
2.5
2.0
1.5
1.0
0.5
0.0
ppm

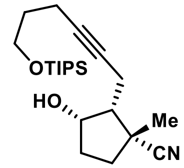


**39**
($CDCl_3$, 126 MHz)

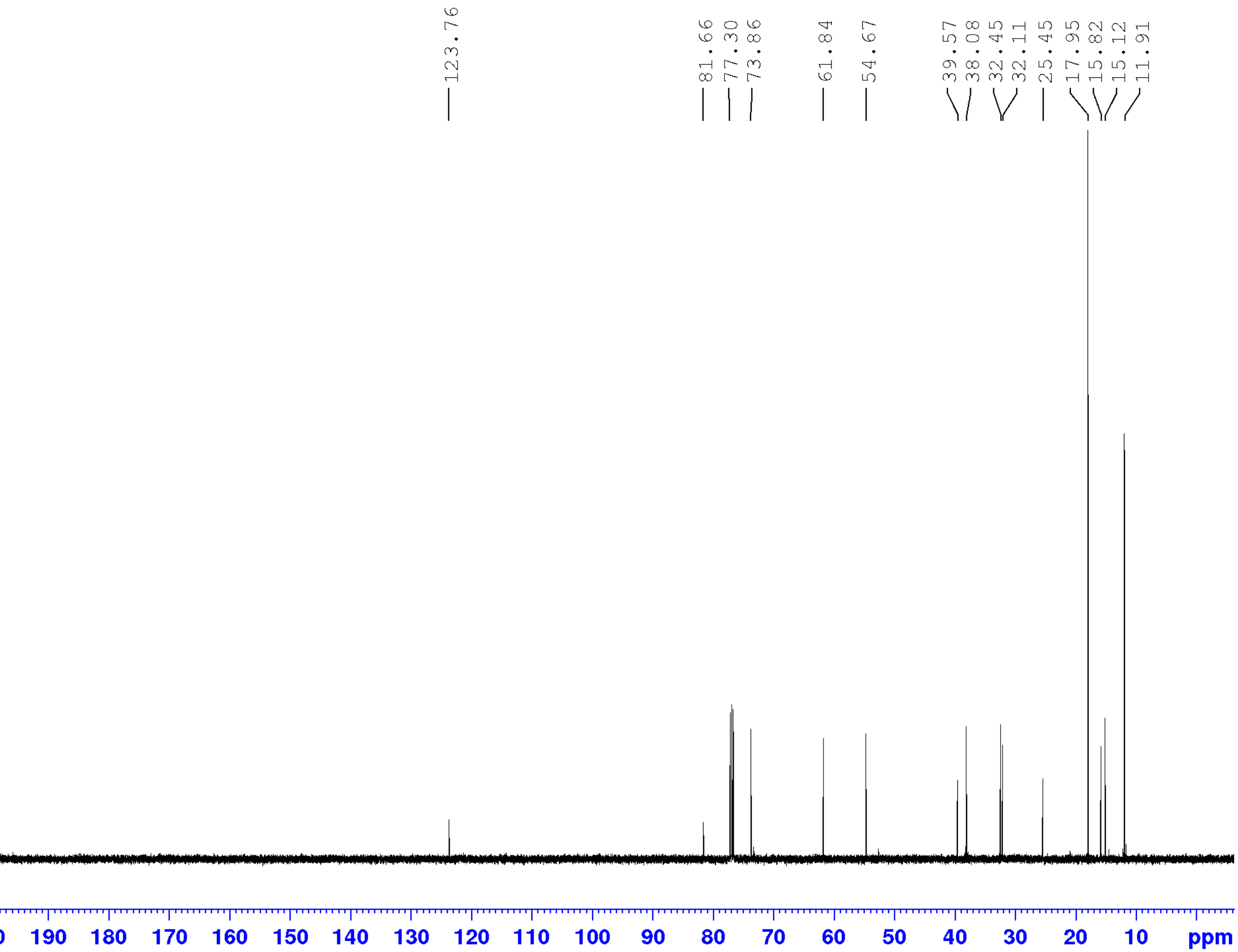

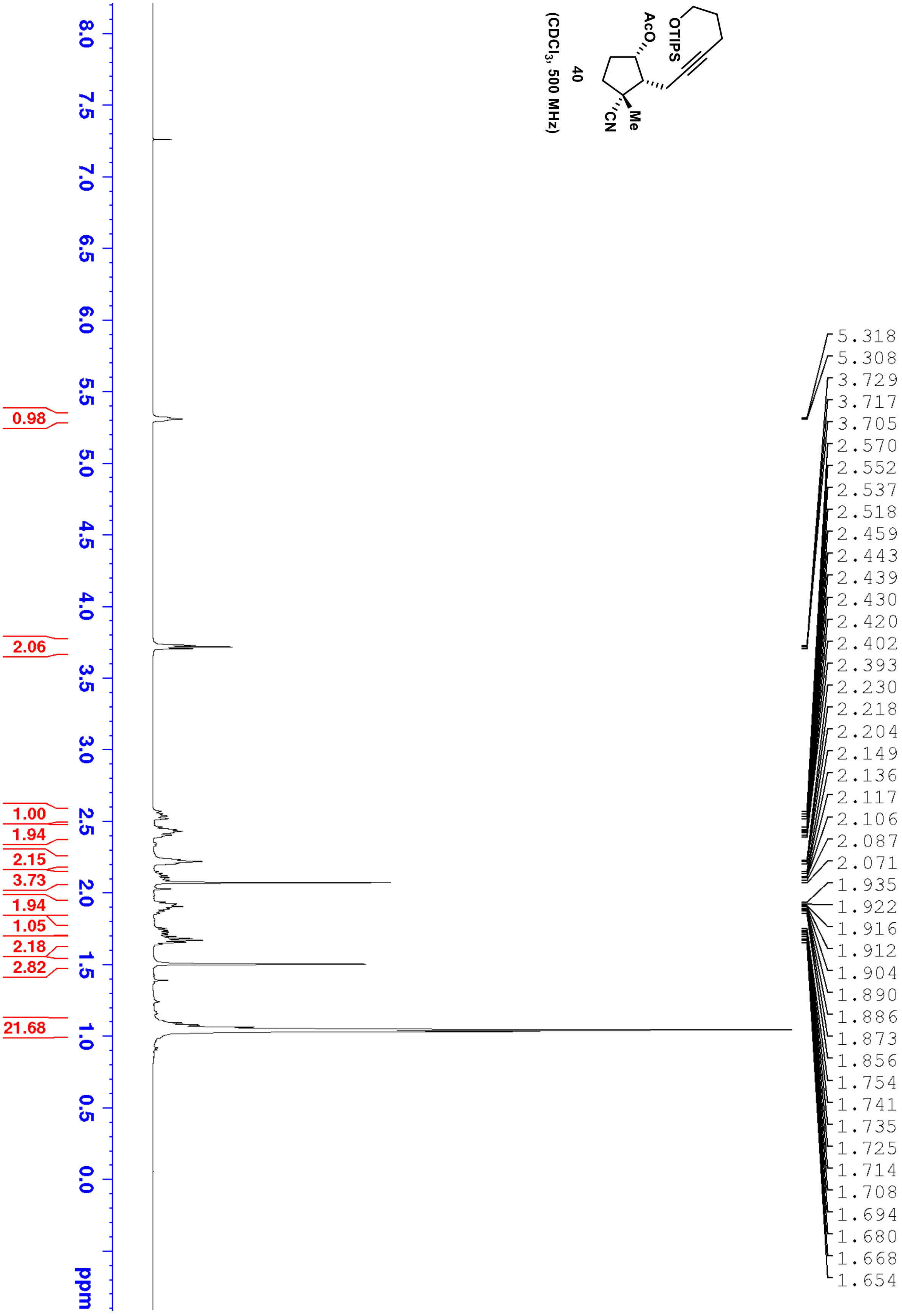
OTIPS
AcO
Me
CN
40
(CDCl3, 500 MHz)
5.318
5.308
3.729
3.717
3.705
2.570
2.552
2.537
2.518
2.459
2.443
2.439
2.430
2.420
2.402
2.393
2.230
2.218
2.204
2.149
2.136
2.117
2.106
2.087
2.071
1.935
1.922
1.916
1.912
1.904
1.890
1.886
1.873
1.856
1.754
1.741
1.735
1.725
1.714
1.708
1.694
1.680
1.668
1.654
0.98
2.06
1.00
1.94
2.15
3.73
1.94
1.05
2.18
2.82
21.68
8.0
7.5
7.0
6.5
6.0
5.5
5.0
4.5
4.0
3.5
3.0
2.5
2.0
1.5
1.0
0.5
0.0
ppm

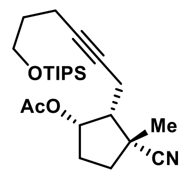


40

($CDCl_3$, 126 MHz)

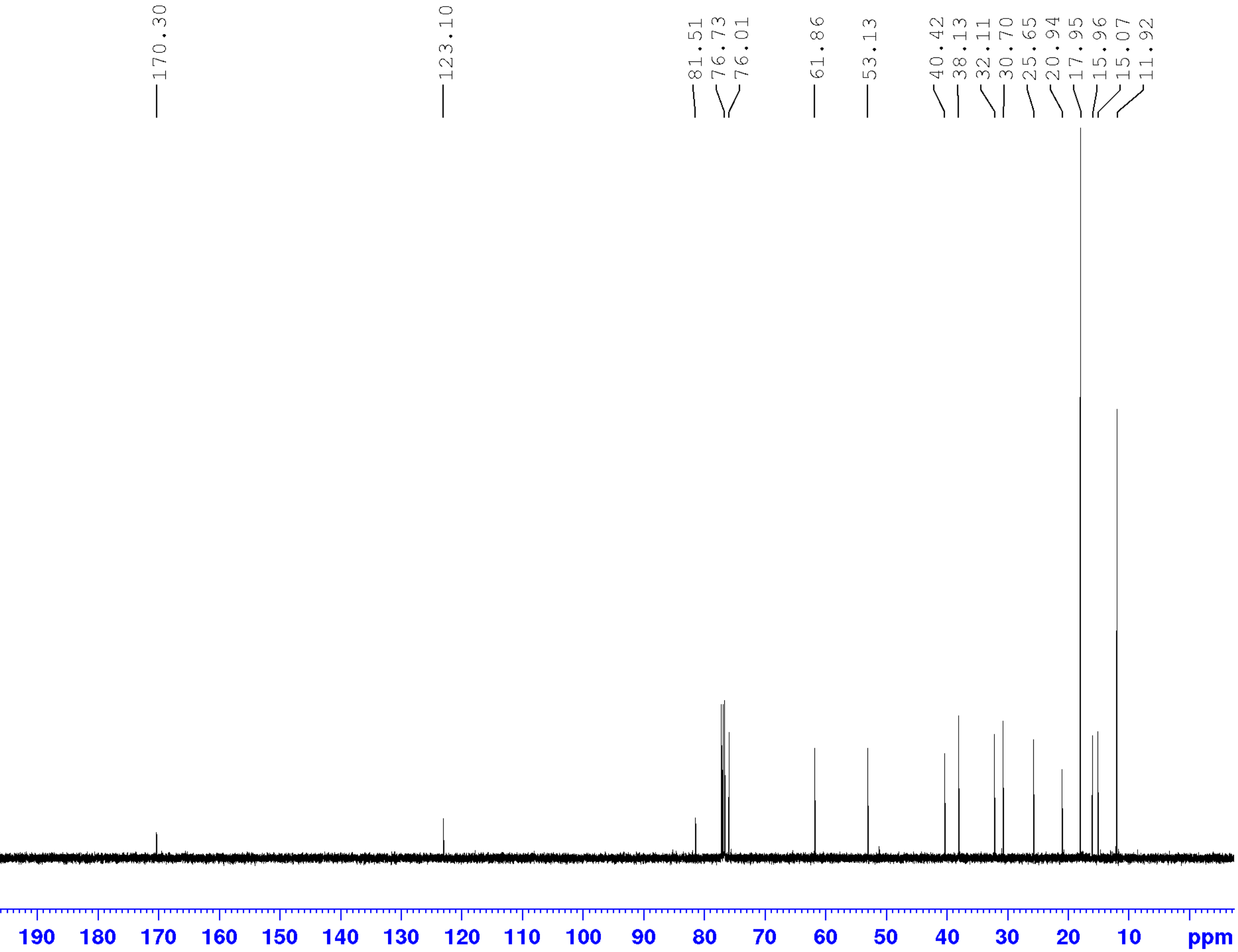

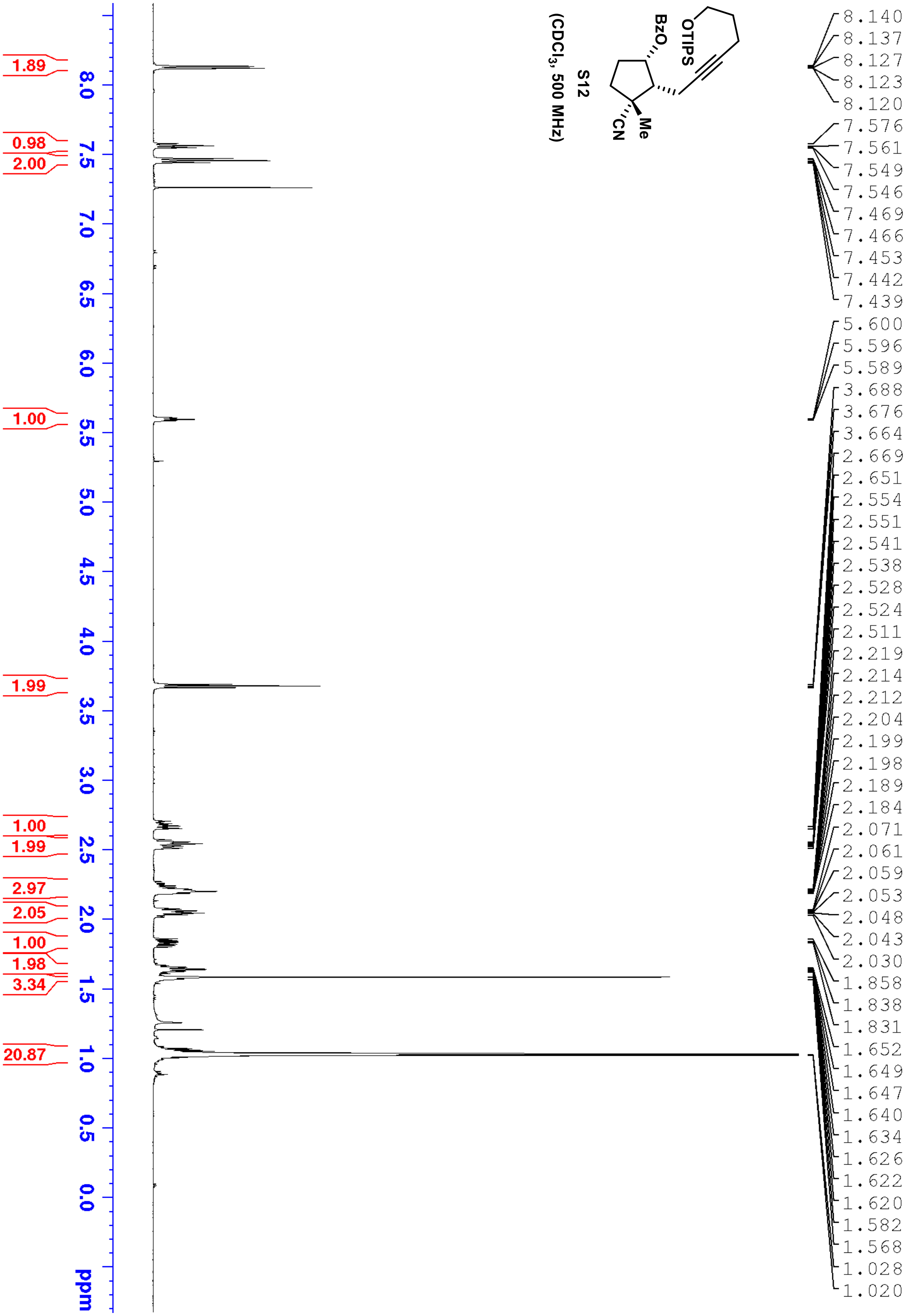
8.140
8.137
8.127
8.123
8.120
7.576
7.561
7.549
7.546
7.469
7.466
7.453
7.442
7.439
5.600
5.596
5.589
3.688
3.676
3.664
2.669
2.651
2.554
2.551
2.541
2.538
2.528
2.524
2.511
2.219
2.214
2.212
2.204
2.199
2.198
2.189
2.184
2.071
2.061
2.059
2.053
2.048
2.043
2.030
1.858
1.838
1.831
1.652
1.649
1.647
1.640
1.634
1.626
1.622
1.620
1.582
1.568
1.028
1.020
OTIPS
BzO
Me
CN
S12
(CDCl3, 500 MHz)
1.89
0.98
2.00
1.00
1.99
1.00
1.99
2.97
2.05
1.00
1.98
3.34
20.87
8.0
7.5
7.0
6.5
6.0
5.5
5.0
4.5
4.0
3.5
3.0
2.5
2.0
1.5
1.0
0.5
0.0
ppm

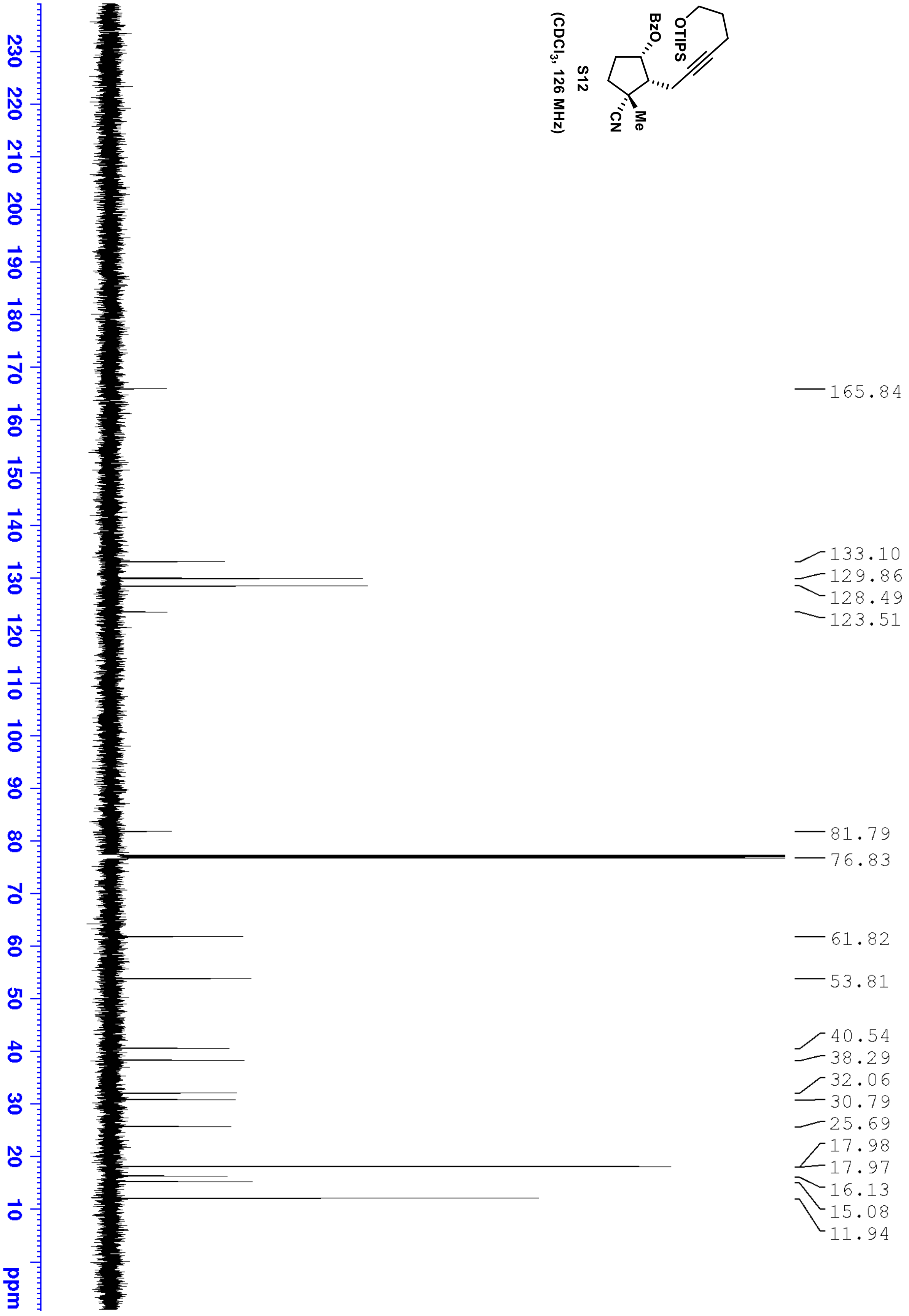
OTIPS
BzO
Me
CN
S12
(CDCl3, 126 MHz)
165.84
133.10
129.86
128.49
123.51
81.79
76.83
61.82
53.81
40.54
38.29
32.06
30.79
25.69
17.98
17.97
16.13
15.08
11.94
230 220 210 200 190 180 170 160 150 140 130 120 110 100 90 80 70 60 50 40 30 20 10 ppm

## Crystal Experiment Section

**Crystal growth of $C_{29}H_{53}NO_4Si_2$**: Pei Qu (prof. Scott Snyder's group).

**Data collected/reported**: Andrew McNeece/Alexander S. Filatov, September/2019 (X-ray Laboratory, Searle B013, Department of Chemistry, the University of Chicago, Chicago, Il).

**General information**: A colorless block was selected and mounted on a Dual-Thickness MicroMount$^{tm}$ (MiTeGen) with 30 μm sample aperture with Fluorolube™ oil. The diffraction data were measured at 100 K on a Bruker D8 VENTURE diffractometer equipped with a microfocus Mo-target X-ray tube ($\lambda$ = 0.71073 Å) and PHOTON 100 CMOS detector. Data were collected using ϕ and ω scans to survey a hemisphere of reciprocal space. Data reduction and integration were performed with the Bruker APEX3 software package (Bruker AXS, version 2017.3-0, 2018). Data were scaled and corrected for absorption effects using the multi-scan procedure as implemented in SADABS (Bruker AXS, version 2014/5, Krause, Herbst-Irmer, Sheldrick & Stalke, *J. Appl. Cryst.* **2015**, *48*, 3-10). The structure was solved by SHELXT (Version 2018/2: Sheldrick, G. M. *Acta Crystallogr*. **2015**, *A71*, 3-8) and refined by a full-matrix least-squares procedure using OLEX2 (O. V. Dolomanov, L. J. Bourhis, R. J. Gildea, J. A. K. Howard and H. Puschmann. J. Appl. Crystallogr. **2009**, *42*, 339-341) (XL refinement program version 2018/3, *Sheldrick, G. M. Acta Crystallogr.* **2015**, *C71*, 3-8). Crystallographic data and details of the data collection and structure refinement are listed in Table 1.

**Specific details for structure refinement**: All atoms were refined with anisotropic thermal parameters. Hydrogen atoms were included in idealized positions for structure factor calculations except those bound to oxygen atom of a carboxylic group which was located in the difference Fourier map. This hydrogen atom was fully independently refined. All structures are drawn with thermal ellipsoids at 50% probability.

**Table 1 Crystal data and structure refinement for mo_0806_Snyder_0m.**

| | |
|---|---|
| Identification code | mo_0806_Snyder_0m |
| Empirical formula | $C_{29}H_{53}NO_4Si_2$ |
| Formula weight | 535.90 |
| Temperature/K | 100(2) |
| Crystal system | monoclinic |
| Space group | $P2_1/c$ |
| a/Å | 16.2099(11) |
| b/Å | 11.5462(8) |
| c/Å | 17.8961(12) |
| α/° | 90 |
| β/° | 106.578(2) |
| γ/° | 90 |
| Volume/Å$^3$ | 3210.3(4) |
| Z | 4 |
| $\rho_{calc}$g/cm$^3$ | 1.109 |
| μ/mm$^{-1}$ | 0.142 |
| F(000) | 1176.0 |
| Crystal size/mm$^3$ | 0.44 × 0.31 × 0.28 |
| Radiation | MoKα (λ = 0.71073) |
| 2Θ range for data collection/° | 4.252 to 56.87 |
| Index ranges | -21 ≤ h ≤ 21, -15 ≤ k ≤ 15, -23 ≤ l ≤ 23 |
| Reflections collected | 110805 |
| Independent reflections | 8064 [$R_{int}$ = 0.0695, $R_{sigma}$ = 0.0329] |
| Data/restraints/parameters | 8064/0/340 |
| Goodness-of-fit on F$^2$ | 1.071 |
| Final R indexes [I>=2σ (I)] | $R_1$ = 0.0482, w$R_2$ = 0.1049 |
| Final R indexes [all data] | $R_1$ = 0.0686, w$R_2$ = 0.1140 |
| Largest diff. peak/hole / e Å$^{-3}$ | 0.58/-0.27 |

$R_{int} = \Sigma \mid F_o^2 - \langle F_o^2 \rangle \mid / \Sigma \mid F_o^2 \mid$

$R1 = \Sigma \mid \mid F_o \mid - \mid F_c \mid \mid / \Sigma \mid F_o \mid$

$wR2 = [\Sigma [w (F_o^2 - F_c^2)^2] / \Sigma [w (F_o^2)^2]]^{1/2}$

Goodness-of-fit = $[\Sigma [w (F_o^2 - F_c^2)^2] / (n-p)]^{1/2}$

n: number of independent reflections; p: number of refined parameters

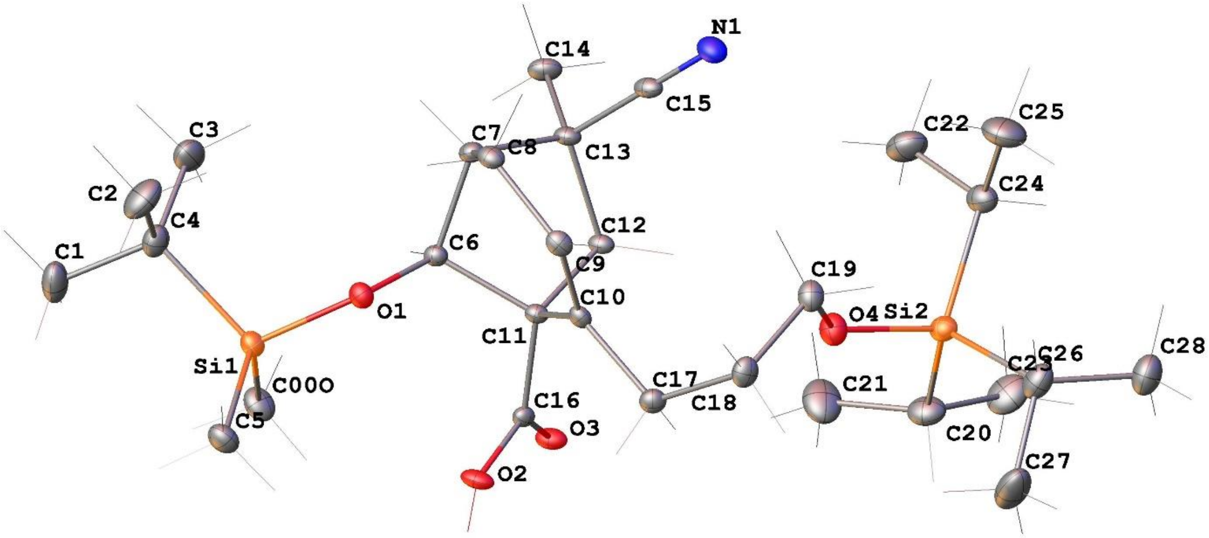
N1
C14
C15
C3
C7
C8
C13
C22
C25
C2
C4
C24
C1
C6
C12
C9
C19
C10
O4
Si2
O1
C11
C28
Si1
C26
C23
C00O
C16
C21
C17
C18
C5
O3
C20
O2
C27

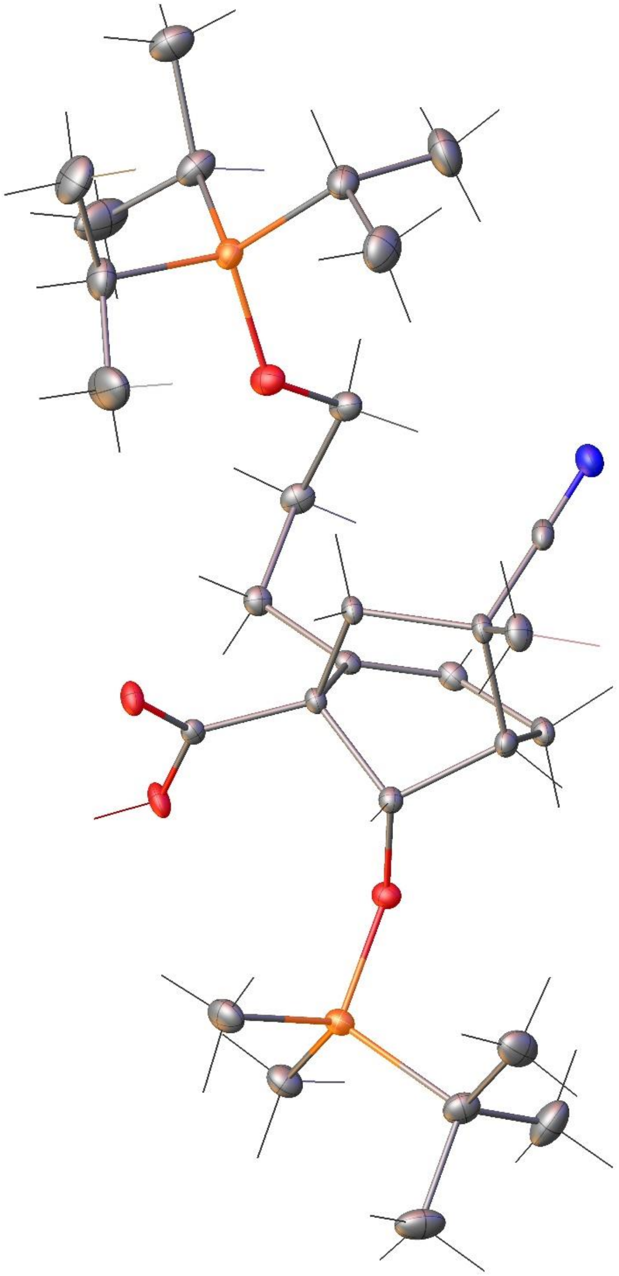

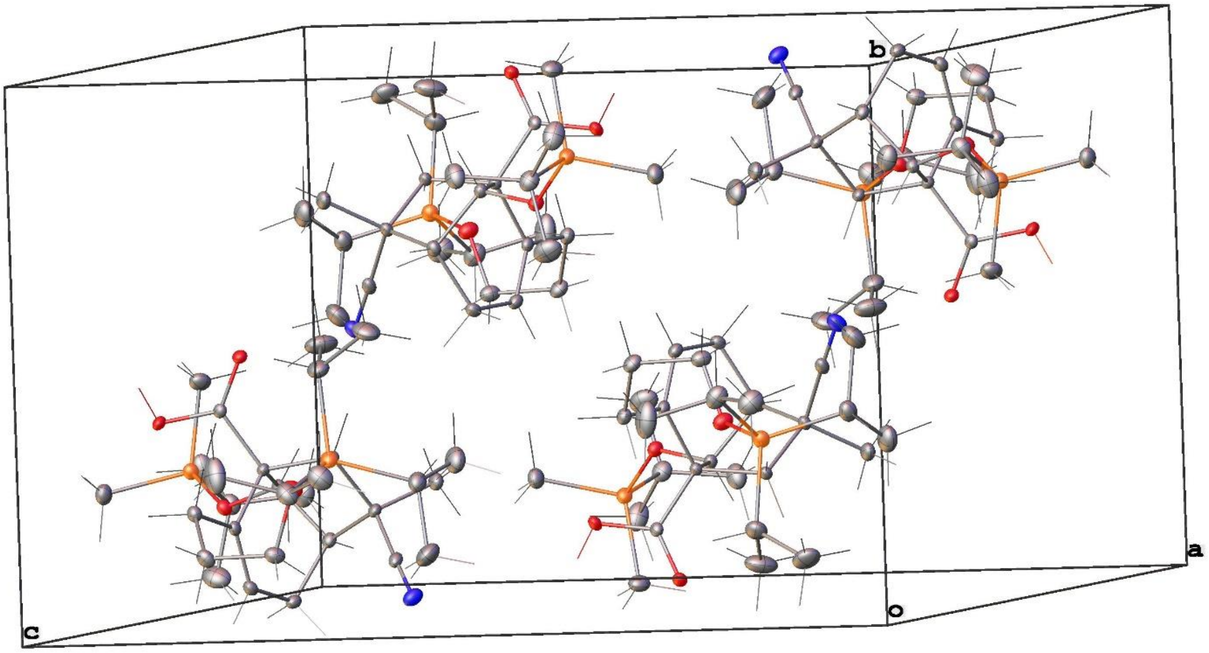
b
a
o
c

**Table 2 Fractional Atomic Coordinates (×10$^4$) and Equivalent Isotropic Displacement Parameters (Å$^2$×10$^3$) for mo_0806_Snyder_0m. $U_{eq}$ is defined as 1/3 of of the trace of the orthogonalised $U_{IJ}$ tensor.**

| Atom | *x* | *y* | *z* | U(eq) |
|---|---|---|---|---|
| Si1 | 2605.0(3) | 2186.7(4) | 3896.5(2) | 15.18(10) |
| Si2 | 8423.7(3) | 2585.0(4) | 4323.4(2) | 17.51(10) |
| O1 | 3450.3(7) | 2925.8(9) | 3816.9(6) | 15.0(2) |
| O2 | 4719.0(7) | 1508.5(9) | 4982.5(6) | 17.2(2) |
| O3 | 5277.7(7) | 412.1(9) | 4219.5(6) | 16.3(2) |
| O4 | 7494.0(7) | 3005.2(10) | 4432.8(7) | 20.5(2) |
| N1 | 6032.0(9) | 4852.7(12) | 2572.3(8) | 21.9(3) |
| C00O | 2755.8(11) | 600.9(14) | 3810.9(10) | 24.4(4) |
| C1 | 838.4(11) | 1975.6(18) | 3137.1(11) | 32.3(4) |
| C2 | 1451.3(12) | 3975.3(17) | 3237.3(12) | 35.0(4) |
| C3 | 1773.8(12) | 2562.3(17) | 2305.5(10) | 28.9(4) |
| C4 | 1625.8(10) | 2696.7(15) | 3112.3(9) | 22.2(3) |
| C5 | 2510.2(11) | 2546.2(15) | 4878.5(9) | 22.8(3) |
| C6 | 4017.1(9) | 2624.7(12) | 3380.8(8) | 12.5(3) |
| C7 | 4157.5(9) | 3621.6(13) | 2865.7(8) | 14.0(3) |
| C8 | 4334.0(10) | 4717.9(12) | 3360.6(8) | 15.0(3) |
| C9 | 5043.9(10) | 4523.1(13) | 4100.9(8) | 14.9(3) |
| C10 | 5334.6(9) | 3478.7(12) | 4359.7(8) | 12.1(3) |
| C11 | 4959.9(9) | 2409.6(12) | 3878.7(8) | 11.3(3) |
| C12 | 5396.3(9) | 2261.9(12) | 3227.6(8) | 12.6(3) |
| C13 | 4943.6(9) | 3186.0(12) | 2601.2(8) | 13.6(3) |
| C14 | 4646.0(11) | 2667.4(14) | 1780.0(9) | 19.9(3) |
| C15 | 5552.2(10) | 4132.1(13) | 2590.6(8) | 15.8(3) |
| C16 | 5010.8(9) | 1342.4(12) | 4375.6(8) | 11.5(3) |
| C17 | 6081.0(9) | 3274.3(13) | 5081.1(8) | 15.6(3) |
| C18 | 6816.4(10) | 4146.6(14) | 5223.1(9) | 18.5(3) |
| C19 | 7253.8(10) | 4158.1(14) | 4577.9(9) | 19.5(3) |
| C20 | 8407.6(12) | 964.1(15) | 4408.9(11) | 27.6(4) |
| C21 | 7508.3(14) | 442.0(17) | 4052.0(14) | 41.8(5) |
| C22 | 7825.9(13) | 2592.4(18) | 2656.5(11) | 34.9(4) |
| C23 | 9066.6(13) | 371.9(17) | 4070.0(12) | 35.7(5) |
| C24 | 8546.0(11) | 3035.8(15) | 3352.1(10) | 23.3(3) |
| C25 | 8628.9(15) | 4350.0(17) | 3288.7(12) | 38.5(5) |
| C26 | 9305.4(11) | 3267.1(16) | 5127.8(10) | 26.6(4) |
| C27 | 9261.7(13) | 2904(2) | 5937.2(11) | 45.3(6) |
| C28 | 10215.5(11) | 3075.5(18) | 5055.2(11) | 31.8(4) |

**Table 3 Anisotropic Displacement Parameters (Å$^2$×10$^3$) for mo_0806_Snyder_0m. The Anisotropic displacement factor exponent takes the form: $-2\pi^2[h^2a^{*2}U_{11}+2hka^*b^*U_{12}+\ldots]$.**

| Atom | $U_{11}$ | $U_{22}$ | $U_{33}$ | $U_{23}$ | $U_{13}$ | $U_{12}$ |
|---|---|---|---|---|---|---|
| Si1 | 15.2(2) | 15.3(2) | 16.1(2) | -1.17(16) | 6.01(15) | -2.10(16) |
| Si2 | 18.2(2) | 16.9(2) | 17.5(2) | 1.62(16) | 5.34(16) | 3.59(17) |
| O1 | 15.6(5) | 14.5(5) | 16.2(5) | -1.5(4) | 6.8(4) | 0.1(4) |
| O2 | 28.5(6) | 11.5(5) | 15.0(5) | 3.9(4) | 11.6(5) | 2.2(4) |
| O3 | 25.0(6) | 11.1(5) | 14.4(5) | 2.1(4) | 8.1(4) | 2.8(4) |
| O4 | 19.6(6) | 17.7(6) | 25.5(6) | -3.4(5) | 8.4(5) | 0.5(4) |
| N1 | 23.6(7) | 18.5(7) | 25.6(7) | 6.4(6) | 10.2(6) | 1.8(6) |
| C00O | 26.9(9) | 17.0(8) | 31.8(9) | -1.8(7) | 12.6(7) | -4.8(7) |
| C1 | 17.5(8) | 49.9(12) | 28.9(9) | -9.0(9) | 5.5(7) | -6.3(8) |
| C2 | 29.2(10) | 30.5(10) | 37.8(11) | -1.0(8) | -2.5(8) | 10.3(8) |
| C3 | 23.7(9) | 39.7(11) | 20.2(8) | 0.5(7) | 1.2(7) | -6.4(8) |
| C4 | 17.1(7) | 26.2(9) | 21.2(8) | -3.7(7) | 2.1(6) | -1.4(6) |
| C5 | 25.6(8) | 25.5(9) | 20.2(8) | -0.1(7) | 11.2(7) | -2.8(7) |
| C6 | 14.5(7) | 12.2(7) | 11.3(6) | 0.1(5) | 4.6(5) | 1.0(5) |
| C7 | 15.1(7) | 14.8(7) | 11.8(7) | 2.4(5) | 3.5(5) | 2.2(6) |
| C8 | 18.7(7) | 11.1(7) | 16.8(7) | 2.4(5) | 7.7(6) | 1.8(6) |
| C9 | 18.8(7) | 12.9(7) | 14.6(7) | -3.3(5) | 7.1(6) | -1.7(6) |
| C10 | 13.7(7) | 13.1(7) | 11.2(6) | -1.2(5) | 6.2(5) | -1.0(5) |
| C11 | 14.3(6) | 9.6(6) | 10.6(6) | 0.7(5) | 4.6(5) | 0.2(5) |
| C12 | 17.3(7) | 10.2(7) | 11.4(6) | 1.6(5) | 5.9(5) | 2.0(5) |
| C13 | 18.8(7) | 11.3(7) | 11.5(7) | 2.8(5) | 5.8(5) | 1.7(6) |
| C14 | 28.3(8) | 19.0(8) | 12.3(7) | 1.2(6) | 5.7(6) | 2.5(6) |
| C15 | 20.5(7) | 14.8(7) | 13.9(7) | 4.2(6) | 7.7(6) | 6.0(6) |
| C16 | 12.0(6) | 12.4(7) | 9.3(6) | -0.4(5) | 1.5(5) | -1.4(5) |
| C17 | 17.7(7) | 16.1(7) | 12.9(7) | -1.2(6) | 4.3(6) | 0.0(6) |
| C18 | 16.8(7) | 19.1(8) | 18.1(7) | -5.7(6) | 2.6(6) | -1.6(6) |
| C19 | 17.4(7) | 16.1(7) | 25.4(8) | -2.1(6) | 6.6(6) | -0.4(6) |
| C20 | 35.2(10) | 19.6(8) | 27.9(9) | 5.2(7) | 8.9(7) | 6.4(7) |
| C21 | 39.9(11) | 20.2(9) | 66.6(15) | 5.8(9) | 17.3(11) | -2.3(8) |
| C22 | 40.1(11) | 41.9(11) | 20.9(9) | 3.5(8) | 5.7(8) | 6.9(9) |
| C23 | 39.6(11) | 23.0(9) | 41.5(11) | -1.8(8) | 6.8(9) | 12.6(8) |
| C24 | 24.6(8) | 25.2(9) | 22.2(8) | 3.4(7) | 10.0(7) | 5.6(7) |
| C25 | 51.6(13) | 30.8(11) | 36.3(11) | 12.4(9) | 17.8(10) | 0.4(9) |
| C26 | 21.2(8) | 30.6(9) | 25.6(9) | -3.3(7) | 2.7(7) | 5.4(7) |
| C27 | 28.4(10) | 81.5(17) | 22.2(9) | -1.5(10) | 1.0(8) | 7.8(11) |
| C28 | 21.1(9) | 36.3(10) | 35.2(10) | -5.3(8) | 3.3(7) | 0.9(8) |

**Table 4 Bond Lengths for mo_0806_Snyder_0m.**

| Atom | Atom | Length/Å | Atom | Atom | Length/Å |
|---|---|---|---|---|---|
| Si1 | O1 | 1.6545(11) | C7 | C13 | 1.564(2) |
| Si1 | C00O | 1.8594(17) | C8 | C9 | 1.503(2) |
| Si1 | C4 | 1.8879(17) | C9 | C10 | 1.329(2) |
| Si1 | C5 | 1.8543(16) | C10 | C11 | 1.5282(19) |
| Si2 | O4 | 1.6473(12) | C10 | C17 | 1.515(2) |
| Si2 | C20 | 1.8787(18) | C11 | C12 | 1.5354(19) |
| Si2 | C24 | 1.8784(17) | C11 | C16 | 1.5081(19) |
| Si2 | C26 | 1.8871(18) | C12 | C13 | 1.5697(19) |
| O1 | C6 | 1.4082(17) | C13 | C14 | 1.532(2) |
| O2 | C16 | 1.3164(17) | C13 | C15 | 1.476(2) |
| O3 | C16 | 1.2199(17) | C17 | C18 | 1.525(2) |
| O4 | C19 | 1.4313(19) | C18 | C19 | 1.518(2) |
| N1 | C15 | 1.146(2) | C20 | C21 | 1.538(3) |
| C1 | C4 | 1.535(2) | C20 | C23 | 1.532(3) |
| C2 | C4 | 1.532(3) | C22 | C24 | 1.531(3) |
| C3 | C4 | 1.538(2) | C24 | C25 | 1.530(3) |
| C6 | C7 | 1.532(2) | C26 | C27 | 1.529(3) |
| C6 | C11 | 1.5538(19) | C26 | C28 | 1.534(2) |
| C7 | C8 | 1.524(2) | | | |

**Table 5 Bond Angles for mo_0806_Snyder_0m.**

| Atom | Atom | Atom | Angle/˚ | Atom | Atom | Atom | Angle/˚ |
|---|---|---|---|---|---|---|---|
| O1 | Si1 | C00O | 111.67(7) | C17 | C10 | C11 | 116.53(12) |
| O1 | Si1 | C4 | 108.45(7) | C10 | C11 | C6 | 111.21(11) |
| O1 | Si1 | C5 | 105.12(7) | C10 | C11 | C12 | 108.79(11) |
| C00O | Si1 | C4 | 110.38(8) | C12 | C11 | C6 | 99.98(11) |
| C5 | Si1 | C00O | 110.29(8) | C16 | C11 | C6 | 110.37(11) |
| C5 | Si1 | C4 | 110.80(8) | C16 | C11 | C10 | 112.44(11) |
| O4 | Si2 | C20 | 104.43(7) | C16 | C11 | C12 | 113.41(11) |
| O4 | Si2 | C24 | 111.73(7) | C11 | C12 | C13 | 104.11(11) |
| O4 | Si2 | C26 | 107.81(7) | C7 | C13 | C12 | 104.94(11) |
| C20 | Si2 | C26 | 112.25(8) | C14 | C13 | C7 | 110.82(12) |
| C24 | Si2 | C20 | 111.04(8) | C14 | C13 | C12 | 112.00(12) |
| C24 | Si2 | C26 | 109.47(8) | C15 | C13 | C7 | 111.80(12) |
| C6 | O1 | Si1 | 127.42(9) | C15 | C13 | C12 | 109.53(12) |
| C19 | O4 | Si2 | 126.93(10) | C15 | C13 | C14 | 107.80(12) |
| C1 | C4 | Si1 | 109.82(12) | N1 | C15 | C13 | 178.55(16) |
| C1 | C4 | C3 | 109.04(14) | O2 | C16 | C11 | 113.29(12) |

| | | | | | | | |
|---|---|---|---|---|---|---|---|
| C2 | C4 | Si1 | 110.07(11) | O3 | C16 | O2 | 123.49(13) |
| C2 | C4 | C1 | 109.20(15) | O3 | C16 | C11 | 123.21(12) |
| C2 | C4 | C3 | 108.62(15) | C10 | C17 | C18 | 115.81(13) |
| C3 | C4 | Si1 | 110.07(11) | C19 | C18 | C17 | 113.04(12) |
| O1 | C6 | C7 | 112.37(11) | O4 | C19 | C18 | 109.88(13) |
| O1 | C6 | C11 | 114.34(11) | C21 | C20 | Si2 | 112.97(13) |
| C7 | C6 | C11 | 100.05(11) | C23 | C20 | Si2 | 112.64(13) |
| C6 | C7 | C13 | 102.38(11) | C23 | C20 | C21 | 110.25(16) |
| C8 | C7 | C6 | 108.05(11) | C22 | C24 | Si2 | 113.73(12) |
| C8 | C7 | C13 | 114.14(12) | C25 | C24 | Si2 | 112.21(12) |
| C9 | C8 | C7 | 110.93(12) | C25 | C24 | C22 | 109.50(15) |
| C10 | C9 | C8 | 123.30(13) | C27 | C26 | Si2 | 112.26(14) |
| C9 | C10 | C11 | 119.48(13) | C27 | C26 | C28 | 110.28(15) |
| C9 | C10 | C17 | 123.78(13) | C28 | C26 | Si2 | 114.38(12) |

**Crystal growth of $C_{16}H_{19}NO_4$**: Pei Qu (prof. Scott Snyder's group).

**Data collected/reported**: Kate Jesse /Alexander S. Filatov, November / 2020 (X-ray Laboratory, Searle B013, Department of Chemistry, the University of Chicago, Chicago, Il).

**General information**: The diffraction data were measured at 100 K on a Bruker D8 VENTURE diffractometer equipped with a microfocus Mo-target X-ray tube ($\lambda$ = 0.71073 Å) and PHOTON 100 CMOS detector. Data were collected using ω scans to survey a sphere of reciprocal space. Data reduction and integration were performed with the Bruker APEX3 software package (Bruker AXS, version 2017.3-0, 2018). Data were scaled and corrected for absorption effects using the multi-scan procedure as implemented in SADABS (Bruker AXS, version 2014/5, Krause, Herbst-Irmer, Sheldrick & Stalke, *J. Appl. Cryst.* **2015**, *48*, 3-10). The structure was solved by SHELXT (Version 2018/2: Sheldrick, G. M. *Acta Crystallogr*. **2015**, *A71*, 3-8) and refined by a full-matrix least-squares procedure using OLEX2 (O. V. Dolomanov, L. J. Bourhis, R. J. Gildea, J. A. K. Howard and H. Puschmann. J. Appl. Crystallogr. **2009**, *42*, 339-341) (XL refinement program version 2018/3, Sheldrick, G. M. Acta Crystallogr. **2015**, *C71*, 3-8). Crystallographic data and details of the data collection and structure refinement are listed in Table 1.

**Specific details for structure refinement**: All atoms were refined with anisotropic thermal parameters. All hydrogen atoms were included in idealized positions for structure factor calculations except the hydrogen atom of the NH group. This atom was found in the difference Fourier map and refined without geometric restraints. All structures are drawn with thermal ellipsoids at 50% probability.

Table 1 Crystal data and structure refinement for 0957_PQ_Snyder_1.

| | |
|---|---|
| Identification code | 0957_PQ_Snyder_1 |
| Empirical formula | $C_{16}H_{19}NO_4$ |
| Formula weight | 289.32 |
| Temperature/K | 100(2) |
| Crystal system | triclinic |
| Space group | P-1 |
| a/Å | 8.2800(6) |
| b/Å | 8.7370(6) |
| c/Å | 10.9196(8) |
| α/° | 66.854(2) |
| β/° | 72.523(2) |
| γ/° | 70.057(2) |
| Volume/Å$^3$ | 670.06(8) |
| Z | 2 |
| $\rho_{calc}$g/cm$^3$ | 1.434 |
| μ/mm$^{-1}$ | 0.103 |
| F(000) | 308.0 |
| Crystal size/mm$^3$ | 0.245 × 0.124 × 0.111 |
| Radiation | MoKα (λ = 0.71073) |
| 2Θ range for data collection/° | 4.134 to 60.026 |
| Index ranges | $-10 \le h \le 11$, $-11 \le k \le 12$, $-15 \le l \le 15$ |
| Reflections collected | 13695 |
| Independent reflections | 3372 [$R_{int}$ = 0.0367, $R_{sigma}$ = 0.0475] |
| Data/restraints/parameters | 3372/0/195 |
| Goodness-of-fit on $F^2$ | 1.025 |
| Final R indexes [I>=2σ (I)] | $R_1$ = 0.0463, $wR_2$ = 0.0972 |
| Final R indexes [all data] | $R_1$ = 0.0741, $wR_2$ = 0.1072 |
| Largest diff. peak/hole / e Å$^{-3}$ | 0.53/-0.26 |

$R_{int} = \Sigma | F_o^2 - \langle F_o^2 \rangle | / \Sigma | F_o^2|$

$R1 = \Sigma | | F_o| - | F_c|| / \Sigma| F_o|$

$wR2 = [\Sigma [w (F_o^2 - F_c^2)^2] / \Sigma [w (F_o^2)^2]]^{1/2}$

Goodness-of-fit = $[\Sigma [w (F_o^2 - F_c^2)^2] / (n-p)^{1/2}$

n: number of independent reflections; p: number of refined parameters

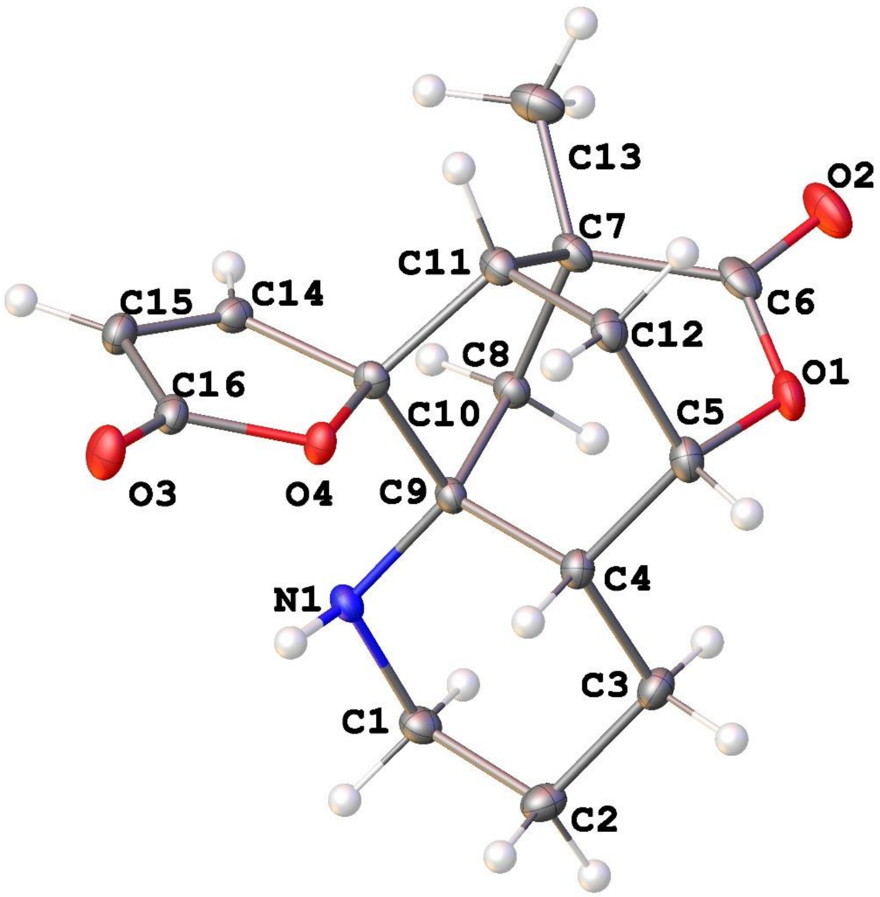

C13
O2
C7
C11
C14
C15
C6
C12
C8
O1
C16
C10
C5
O3
O4
C9
C4
N1
C3
C1
C2

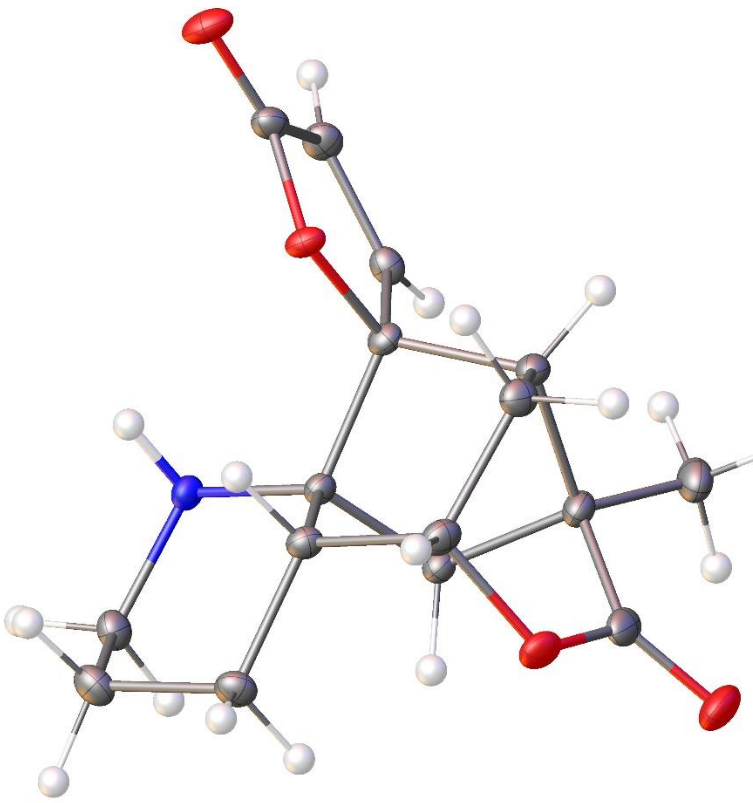

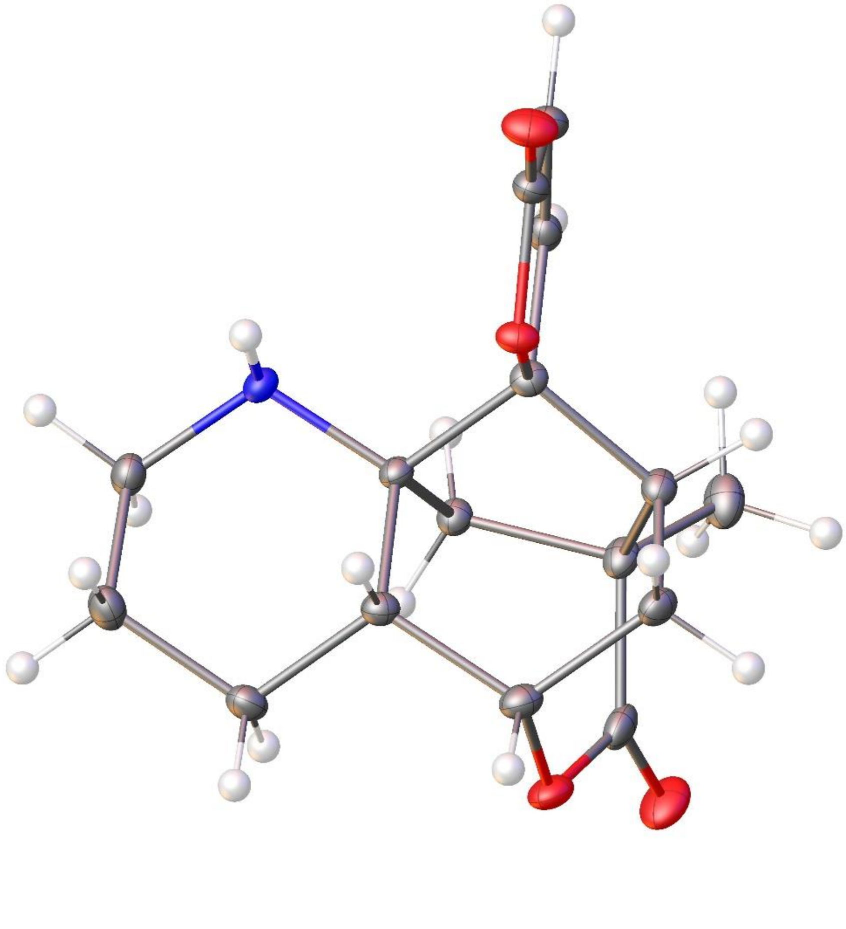

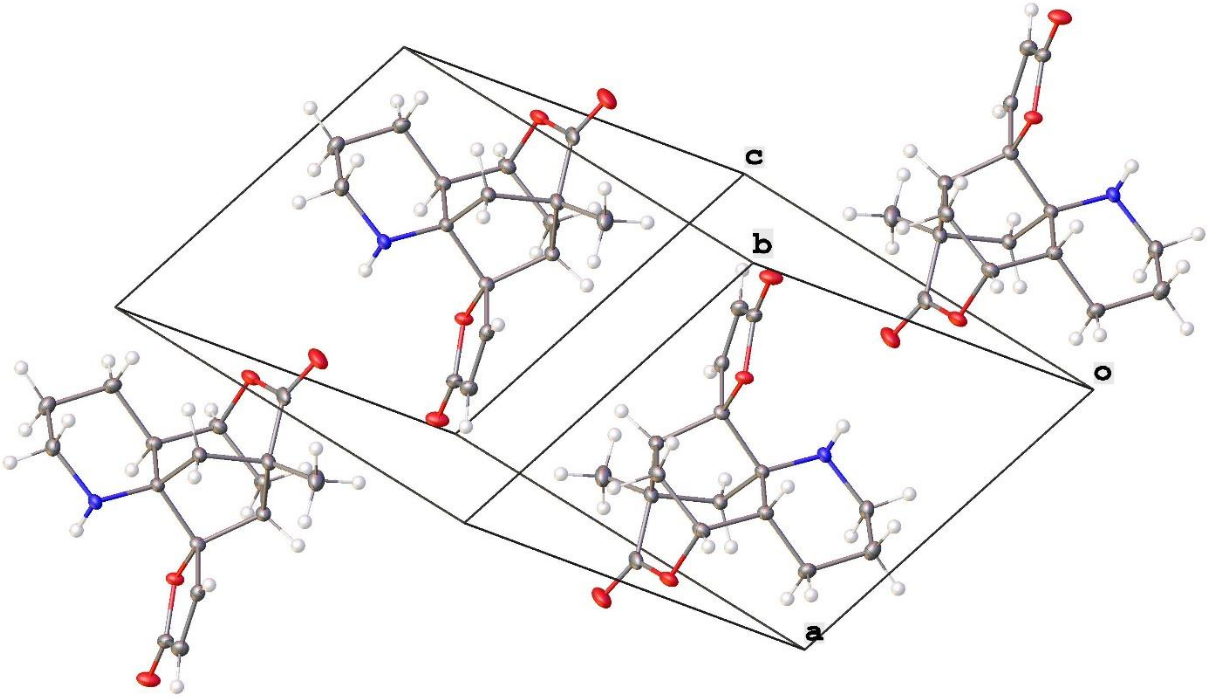
c
b
o
a

Table 2 Fractional Atomic Coordinates (×10[4]) and Equivalent Isotropic Displacement Parameters (Å[2]×10[3]) for 0957_PQ_Snyder_1. $U_{eq}$ is defined as 1/3 of of the trace of the orthogonalised $U_{IJ}$ tensor.

| Atom | *x* | *y* | *z* | U(eq) |
|---|---|---|---|---|
| O1 | 297.5(13) | 6835.6(14) | 8952.0(11) | 17.4(3) |
| O2 | -1260.9(14) | 7811.5(15) | 7362.8(13) | 24.9(3) |
| O3 | 9122.6(13) | 4210.9(14) | 7139.5(11) | 18.6(3) |
| O4 | 6233.3(12) | 5383.5(13) | 7623.0(10) | 12.2(2) |
| N1 | 4863.2(16) | 9240.9(16) | 7284.6(12) | 11.9(3) |
| C1 | 3970.2(19) | 10508.8(19) | 8000.6(15) | 14.7(3) |
| C2 | 3261(2) | 9736(2) | 9505.0(15) | 16.2(3) |
| C3 | 2095.6(19) | 8602.4(19) | 9703.3(15) | 14.8(3) |
| C4 | 3095.3(18) | 7229.5(18) | 9023.9(14) | 11.9(3) |
| C5 | 2038.5(18) | 5959.6(19) | 9297.1(16) | 14.6(3) |
| C6 | 171.8(19) | 7317.1(19) | 7646.2(17) | 16.5(3) |
| C7 | 1863.2(18) | 7402.8(19) | 6596.2(15) | 13.9(3) |
| C8 | 2382.9(18) | 8929.1(18) | 6655.1(15) | 11.9(3) |
| C9 | 3829.8(18) | 8063.0(18) | 7504.4(14) | 10.8(3) |
| C10 | 4884.4(18) | 6622.8(18) | 6881.0(14) | 11.2(3) |
| C11 | 3424.6(18) | 5797.1(19) | 7020.0(15) | 13.0(3) |
| C12 | 2935.5(19) | 4719.3(19) | 8494.0(15) | 14.4(3) |
| C13 | 1558(2) | 7680(2) | 5209.0(17) | 20.0(4) |
| C14 | 5875.8(19) | 7233.6(19) | 5464.0(15) | 12.6(3) |
| C15 | 7554.5(19) | 6404.2(19) | 5396.1(15) | 14.5(3) |
| C16 | 7820.0(19) | 5211.8(19) | 6753.6(15) | 13.4(3) |

Table 3 Anisotropic Displacement Parameters (Å[2]×10[3]) for 0957_PQ_Snyder_1. The Anisotropic displacement factor exponent takes the form: $-2\pi^2[h^2a^{*2}U_{11}+2hka^*b^*U_{12}+\ldots]$.

| Atom | $U_{11}$ | $U_{22}$ | $U_{33}$ | $U_{23}$ | $U_{13}$ | $U_{12}$ |
|---|---|---|---|---|---|---|
| O1 | 9.2(5) | 19.1(6) | 23.8(6) | -8.3(5) | 1.0(4) | -5.4(4) |
| O2 | 11.8(5) | 25.6(6) | 41.4(8) | -14.0(6) | -8.3(5) | -3.3(5) |
| O3 | 10.8(5) | 20.3(6) | 18.2(6) | -4.7(5) | -2.1(4) | 1.4(4) |
| O4 | 8.6(5) | 12.9(5) | 11.6(5) | -3.0(4) | -1.1(4) | -0.6(4) |
| N1 | 9.6(6) | 12.2(6) | 15.2(7) | -4.8(5) | -2.0(5) | -4.1(5) |
| C1 | 15.9(7) | 13.7(7) | 16.8(8) | -6.0(6) | -3.4(6) | -5.3(6) |
| C2 | 20.3(8) | 15.6(8) | 14.4(8) | -6.9(6) | -2.5(6) | -4.7(6) |
| C3 | 14.7(7) | 15.8(8) | 13.0(8) | -6.2(6) | 1.0(6) | -4.0(6) |

Table 3 Anisotropic Displacement Parameters ($Å^2×10^3$) for 0957_PQ_Snyder_1. The Anisotropic displacement factor exponent takes the form: $-2\pi^2[h^2a^{*2}U_{11}+2hka^*b^*U_{12}+…]$.

| Atom | $U_{11}$ | $U_{22}$ | $U_{33}$ | $U_{23}$ | $U_{13}$ | $U_{12}$ |
|---|---|---|---|---|---|---|
| C4 | 10.3(7) | 11.9(7) | 12.3(7) | -3.7(6) | -1.2(5) | -2.5(5) |
| C5 | 10.7(7) | 14.9(8) | 16.7(8) | -4.3(6) | -0.6(6) | -4.1(6) |
| C6 | 13.5(7) | 11.7(7) | 27.8(9) | -8.4(7) | -4.7(6) | -4.3(6) |
| C7 | 10.9(7) | 13.4(7) | 19.4(8) | -6.2(6) | -5.1(6) | -2.3(6) |
| C8 | 10.5(7) | 11.3(7) | 14.3(7) | -4.4(6) | -3.3(6) | -2.2(6) |
| C9 | 8.6(6) | 11.2(7) | 13.0(7) | -4.4(6) | -1.3(5) | -2.9(5) |
| C10 | 9.7(7) | 10.9(7) | 12.0(7) | -2.6(6) | -3.9(5) | -1.0(5) |
| C11 | 10.5(7) | 11.9(7) | 17.4(8) | -6.4(6) | -3.1(6) | -1.6(6) |
| C12 | 13.3(7) | 11.9(7) | 17.9(8) | -4.2(6) | -1.2(6) | -5.3(6) |
| C13 | 19.1(8) | 19.2(8) | 26.7(9) | -10.1(7) | -11.2(7) | -1.7(6) |
| C14 | 15.2(7) | 12.0(7) | 11.5(7) | -3.9(6) | -1.9(6) | -5.1(6) |
| C15 | 13.9(7) | 16.2(8) | 12.7(8) | -5.2(6) | 0.9(6) | -5.4(6) |
| C16 | 11.5(7) | 13.7(7) | 15.3(8) | -7.0(6) | 0.7(6) | -3.6(6) |

Table 4 Bond Lengths for 0957_PQ_Snyder_1.

| Atom | Atom | Length/Å | Atom | Atom | Length/Å |
|---|---|---|---|---|---|
| O1 | C5 | 1.4673(17) | C5 | C12 | 1.517(2) |
| O1 | C6 | 1.3448(19) | C6 | C7 | 1.524(2) |
| O2 | C6 | 1.2064(18) | C7 | C8 | 1.563(2) |
| O3 | C16 | 1.2022(17) | C7 | C11 | 1.570(2) |
| O4 | C10 | 1.4510(16) | C7 | C13 | 1.522(2) |
| O4 | C16 | 1.3705(17) | C8 | C9 | 1.5379(19) |
| N1 | C1 | 1.4697(19) | C9 | C10 | 1.541(2) |
| N1 | C9 | 1.4602(18) | C10 | C11 | 1.550(2) |
| C1 | C2 | 1.523(2) | C10 | C14 | 1.496(2) |
| C2 | C3 | 1.522(2) | C11 | C12 | 1.523(2) |
| C3 | C4 | 1.526(2) | C14 | C15 | 1.325(2) |
| C4 | C5 | 1.527(2) | C15 | C16 | 1.469(2) |
| C4 | C9 | 1.549(2) | | | |

Table 5 Bond Angles for 0957_PQ_Snyder_1.

| Atom | Atom | Atom | Angle/° | Atom | Atom | Atom | Angle/° |
|---|---|---|---|---|---|---|---|
| C6 | O1 | C5 | 117.57(11) | N1 | C9 | C4 | 113.01(11) |
| C16 | O4 | C10 | 109.87(11) | N1 | C9 | C8 | 111.47(12) |
| C9 | N1 | C1 | 113.32(11) | N1 | C9 | C10 | 111.90(11) |
| N1 | C1 | C2 | 114.40(12) | C8 | C9 | C4 | 111.92(11) |
| C3 | C2 | C1 | 110.21(12) | C8 | C9 | C10 | 98.97(11) |
| C2 | C3 | C4 | 110.14(12) | C10 | C9 | C4 | 108.73(11) |
| C3 | C4 | C5 | 112.25(12) | O4 | C10 | C9 | 112.54(11) |
| C3 | C4 | C9 | 110.81(12) | O4 | C10 | C11 | 111.16(11) |
| C5 | C4 | C9 | 114.56(12) | O4 | C10 | C14 | 103.46(11) |
| O1 | C5 | C4 | 112.05(11) | C9 | C10 | C11 | 101.04(11) |
| O1 | C5 | C12 | 106.99(11) | C14 | C10 | C9 | 114.44(12) |
| C12 | C5 | C4 | 112.48(12) | C14 | C10 | C11 | 114.56(12) |
| O1 | C6 | C7 | 116.79(12) | C10 | C11 | C7 | 102.92(11) |
| O2 | C6 | O1 | 118.95(14) | C12 | C11 | C7 | 110.33(12) |
| O2 | C6 | C7 | 123.84(15) | C12 | C11 | C10 | 109.44(12) |
| C6 | C7 | C8 | 103.51(12) | C5 | C12 | C11 | 107.02(12) |
| C6 | C7 | C11 | 112.96(12) | C15 | C14 | C10 | 110.06(13) |
| C8 | C7 | C11 | 104.41(11) | C14 | C15 | C16 | 108.71(13) |
| C13 | C7 | C6 | 110.36(12) | O3 | C16 | O4 | 121.52(13) |
| C13 | C7 | C8 | 113.56(12) | O3 | C16 | C15 | 130.64(14) |
| C13 | C7 | C11 | 111.69(12) | O4 | C16 | C15 | 107.84(12) |
| C9 | C8 | C7 | 104.70(11) | | | | |

**Data collected/reported**: Kate Jesse /Alexander S. Filatov, December / 2020 (X-ray Laboratory, Searle B013, Department of Chemistry, the University of Chicago, Chicago, Il).

**General information**: The diffraction data were measured at 100 K on a Bruker D8 VENTURE diffractometer equipped with a microfocus Mo-target X-ray tube ($\lambda$ = 0.71073 Å) and PHOTON 100 CMOS detector. Data were collected using ω scans to survey a sphere of reciprocal space. Data reduction and integration were performed with the Bruker APEX3 software package (Bruker AXS, version 2017.3-0, 2018). Data were scaled and corrected for absorption effects using the multi-scan procedure as implemented in SADABS (Bruker AXS, version 2014/5, Krause, Herbst-Irmer, Sheldrick & Stalke, *J. Appl. Cryst.* **2015**, *48*, 3-10). The structure was solved by SHELXT (Version 2018/2: Sheldrick, G. M. *Acta Crystallogr*. **2015**, *A**71***, 3-8) and refined by a full-matrix least-squares procedure using OLEX2 (O. V. Dolomanov, L. J. Bourhis, R. J. Gildea, J. A. K. Howard and H. Puschmann. J. Appl. Crystallogr. **2009**, *42*, 339-341) (XL refinement program version 2018/3, Sheldrick, G. M. Acta Crystallogr. **2015**, *C71*, 3-8). Crystallographic data and details of the data collection and structure refinement are listed in Table 1.

**Specific details for structure refinement**: All atoms were refined with anisotropic thermal parameters. All hydrogen atoms were included in idealized positions for structure factor calculations except the hydrogen atom of the OH group. This atom was found in the difference Fourier map and refined without any restraints. All structures are drawn with thermal ellipsoids at 50% probability.

Table 1 Crystal data and structure refinement for 0961_PQ_Snyder_2.

| | |
|---|---|
| Identification code | 0961_PQ_Snyder_2 |
| Empirical formula | $C_{16}H_{19}NO_4$ |
| Formula weight | 289.32 |
| Temperature/K | 100(2) |
| Crystal system | monoclinic |
| Space group | $P2_1/n$ |
| a/Å | 6.9925(5) |
| b/Å | 22.6400(15) |
| c/Å | 8.7190(6) |
| α/° | 90 |
| β/° | 107.547(2) |
| γ/° | 90 |
| Volume/Å$^3$ | 1316.08(16) |
| Z | 4 |
| $\rho_{calc}$g/cm$^3$ | 1.460 |
| μ/mm$^{-1}$ | 0.105 |
| F(000) | 616.0 |
| Crystal size/mm$^3$ | 0.256 × 0.161 × 0.096 |
| Radiation | MoKα (λ = 0.71073) |
| 2Θ range for data collection/° | 5.22 to 61.02 |
| Index ranges | $-9 \le h \le 9$, $-30 \le k \le 31$, $-12 \le l \le 12$ |
| Reflections collected | 20251 |
| Independent reflections | 3851 [$R_{int}$ = 0.0478, $R_{sigma}$ = 0.0464] |
| Data/restraints/parameters | 3851/0/195 |
| Goodness-of-fit on $F^2$ | 1.044 |
| Final R indexes [I>=2σ (I)] | $R_1$ = 0.0528, $wR_2$ = 0.1147 |
| Final R indexes [all data] | $R_1$ = 0.0846, $wR_2$ = 0.1270 |
| Largest diff. peak/hole / e Å$^{-3}$ | 0.49/-0.24 |

$R_{int} = \Sigma | F_o^2 - \langle F_o^2 \rangle | / \Sigma | F_o^2|$

$R1 = \Sigma || F_o| - | F_c|| / \Sigma| F_o|$

$wR2 = [\Sigma [w (F_o^2 - F_c^2)^2] / \Sigma [w (F_o^2)^2]]^{1/2}$

$\text{Goodness-of-fit} = [\Sigma [w (F_o^2 - F_c^2)^2] / (n-p)]^{1/2}$

n: number of independent reflections; p: number of refined parameters

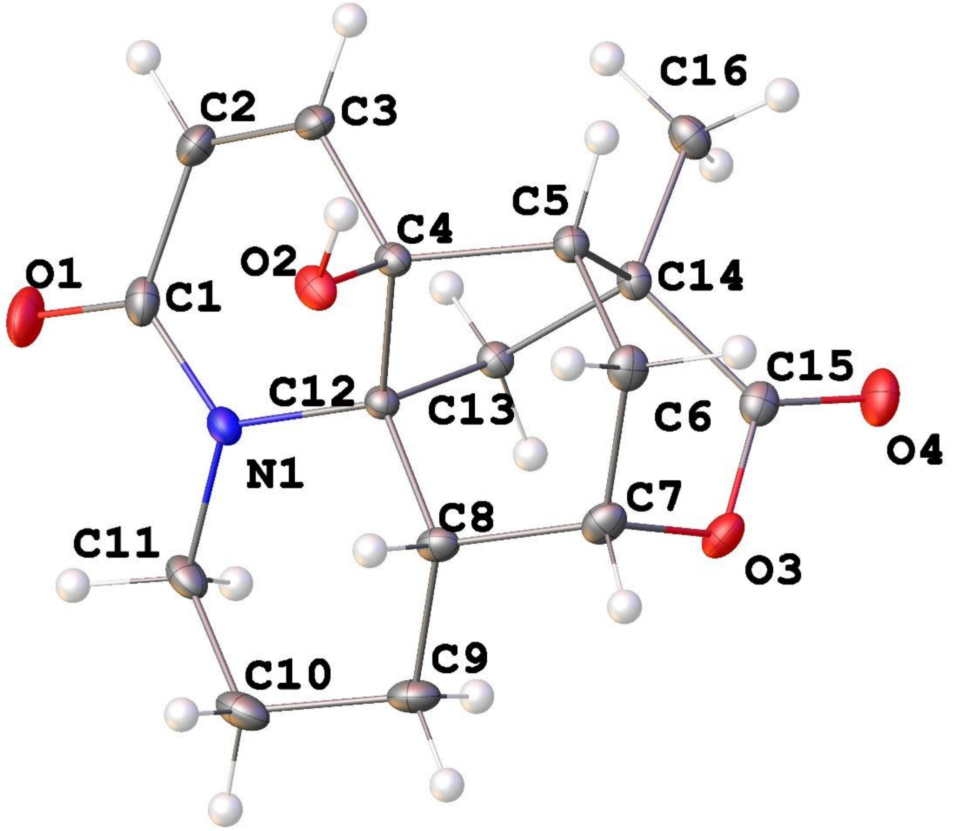
C16
C2
C3
C5
C4
O2
C14
O1
C1
C15
O4
C12
C13
C6
N1
C7
C8
C11
O3
C9
C10

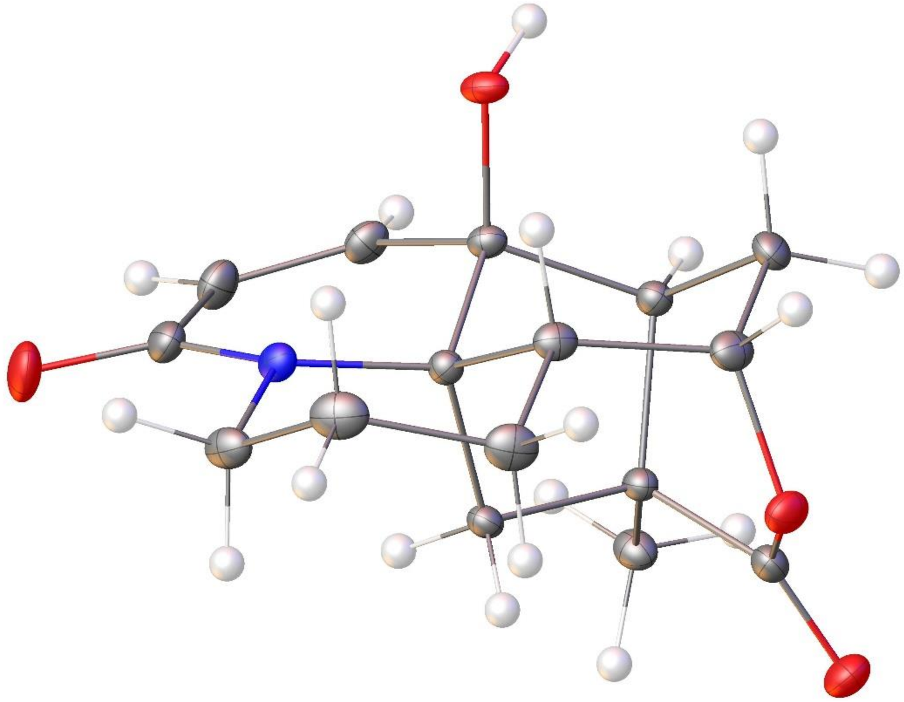

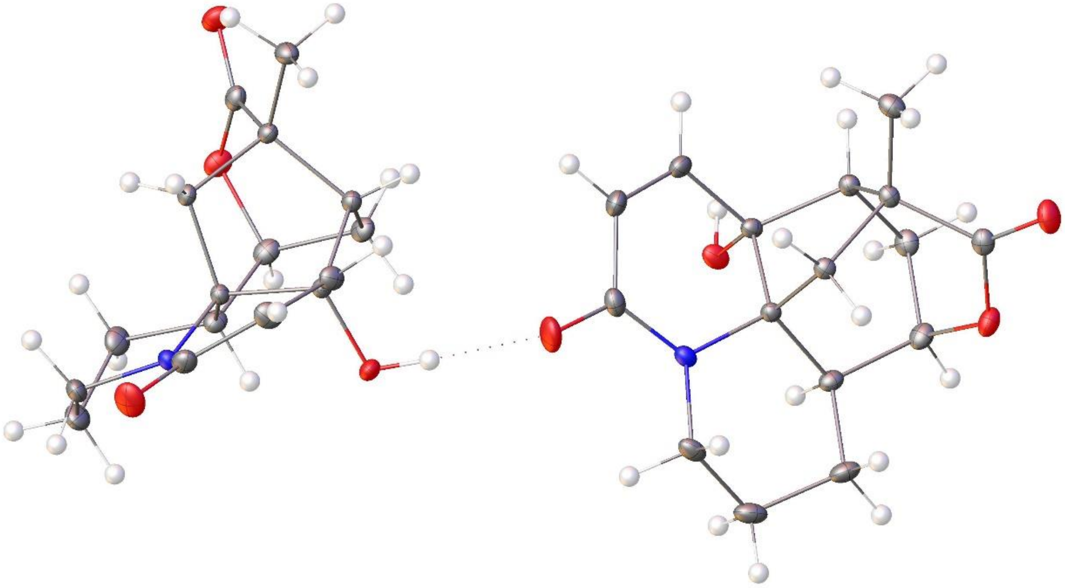

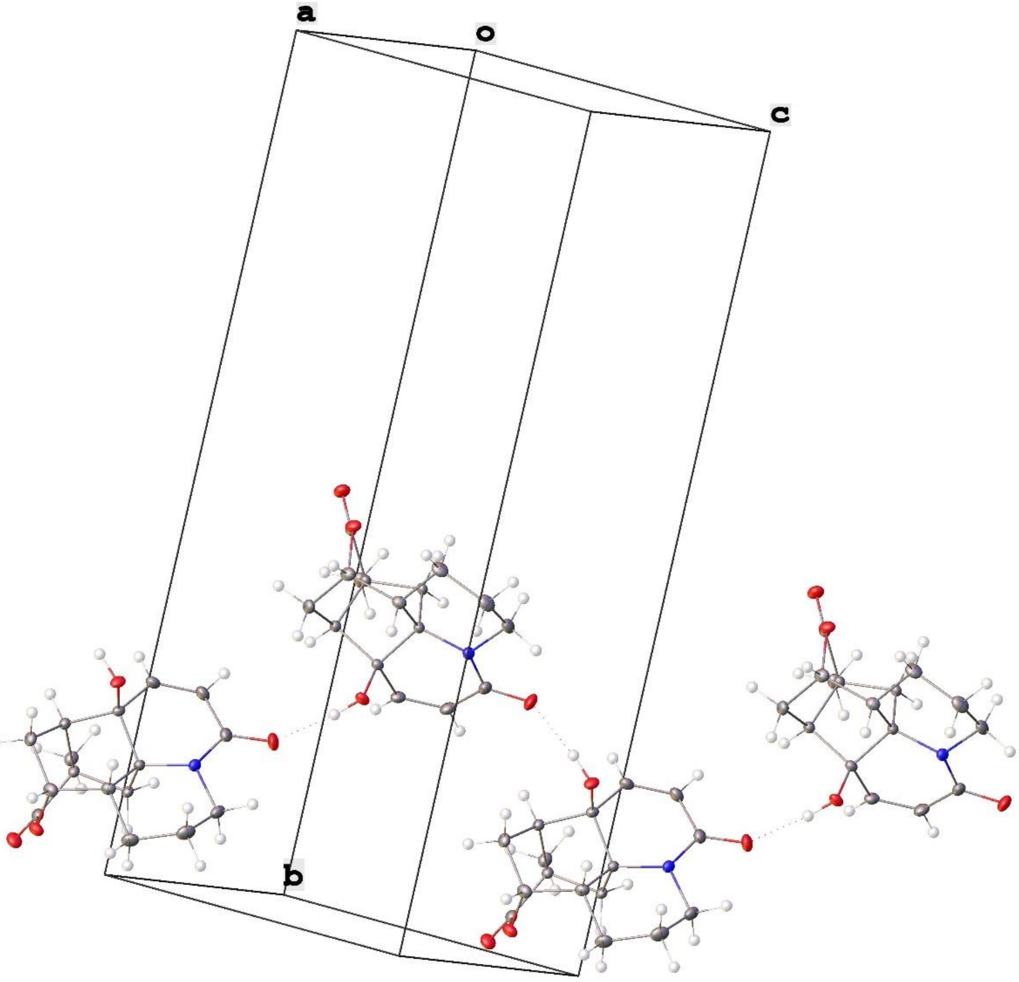
a
o
c
b

Table 2 Fractional Atomic Coordinates (×10⁴) and Equivalent Isotropic Displacement Parameters (Å²×10³) for 0961_PQ_Snyder_2. $U_{eq}$ is defined as 1/3 of of the trace of the orthogonalised $U_{IJ}$ tensor.

| **Atom** | ***x*** | ***y*** | ***z*** | **U(eq)** |
|---|---|---|---|---|
| O1 | 3938(2) | 6965.3(6) | 8801.5(15) | 29.5(3) |
| O2 | 8423.6(17) | 7299.0(5) | 6039.3(13) | 17.7(2) |
| O3 | 8312.5(17) | 5405.2(5) | 4428.6(14) | 19.9(3) |
| O4 | 5812.3(19) | 5132.9(5) | 2344.0(14) | 24.6(3) |
| N1 | 6245(2) | 6537.0(6) | 7822.2(14) | 14.9(3) |
| C1 | 4733(3) | 6922.3(7) | 7713.0(19) | 19.1(3) |
| C2 | 4028(3) | 7289.8(7) | 6252.3(19) | 20.2(3) |
| C3 | 4901(2) | 7286.8(7) | 5096.1(19) | 17.2(3) |
| C4 | 6768(2) | 6926.4(6) | 5302.6(17) | 13.0(3) |
| C5 | 6894(2) | 6622.2(6) | 3749.9(17) | 13.4(3) |
| C6 | 9049(2) | 6416.3(7) | 4028.0(19) | 16.8(3) |
| C7 | 9409(2) | 5922.3(7) | 5255.3(19) | 17.8(3) |
| C8 | 8866(2) | 6097.0(7) | 6772.7(18) | 15.4(3) |
| C9 | 9118(2) | 5597.8(7) | 7983.9(19) | 21.1(3) |
| C10 | 8735(3) | 5841.0(8) | 9487.1(19) | 23.7(4) |
| C11 | 6657(3) | 6098.0(7) | 9133.2(18) | 19.2(3) |
| C12 | 6772(2) | 6385.3(6) | 6360.8(17) | 12.2(3) |
| C13 | 5153(2) | 5998.0(6) | 5231.0(17) | 12.7(3) |
| C14 | 5480(2) | 6070.8(6) | 3555.6(17) | 13.1(3) |
| C15 | 6527(2) | 5508.4(7) | 3327.1(18) | 16.6(3) |
| C16 | 3511(2) | 6143.3(7) | 2227.1(18) | 17.8(3) |

Table 3 Anisotropic Displacement Parameters (Å²×10³) for 0961_PQ_Snyder_2. The Anisotropic displacement factor exponent takes the form: $-2\pi^2[h^2a^{*2}U_{11}+2hka^*b^*U_{12}+\ldots]$.

| **Atom** | **$U_{11}$** | **$U_{22}$** | **$U_{33}$** | **$U_{23}$** | **$U_{13}$** | **$U_{12}$** |
|---|---|---|---|---|---|---|
| O1 | 43.9(8) | 27.0(7) | 26.5(6) | -3.8(5) | 24.0(6) | 4.4(6) |
| O2 | 22.3(6) | 13.9(5) | 15.7(5) | -0.6(4) | 4.1(5) | -7.1(4) |
| O3 | 19.4(6) | 14.4(5) | 26.6(6) | -3.3(4) | 8.0(5) | 2.1(4) |
| O4 | 31.2(7) | 19.0(6) | 25.6(6) | -8.6(5) | 11.3(5) | -5.1(5) |
| N1 | 19.5(7) | 14.4(6) | 10.6(6) | 0.1(5) | 4.5(5) | -0.8(5) |
| C1 | 24.5(8) | 17.1(8) | 17.7(7) | -4.7(6) | 9.6(6) | -2.4(6) |
| C2 | 23.3(8) | 16.8(8) | 20.8(8) | -2.0(6) | 7.1(7) | 5.8(6) |
| C3 | 20.3(8) | 12.5(7) | 17.0(7) | 0.5(6) | 3.1(6) | 3.6(6) |

Table 3 Anisotropic Displacement Parameters ($Å^2×10^3$) for 0961_PQ_Snyder_2. The Anisotropic displacement factor exponent takes the form: $-2\pi^2[h^2a^{*2}U_{11}+2hka^*b^*U_{12}+…]$.

| Atom | $U_{11}$ | $U_{22}$ | $U_{33}$ | $U_{23}$ | $U_{13}$ | $U_{12}$ |
|---|---|---|---|---|---|---|
| C4 | 14.7(7) | 11.1(7) | 12.3(6) | 0.7(5) | 2.7(5) | -1.3(5) |
| C5 | 14.5(7) | 12.8(7) | 13.4(6) | 0.3(5) | 4.9(5) | -1.0(5) |
| C6 | 13.7(7) | 19.2(8) | 19.6(7) | -1.2(6) | 8.0(6) | -2.7(6) |
| C7 | 12.1(7) | 17.1(8) | 23.9(8) | -1.1(6) | 4.9(6) | 0.6(6) |
| C8 | 12.6(7) | 14.7(7) | 17.1(7) | 1.4(6) | 1.4(6) | 0.7(6) |
| C9 | 19.5(8) | 18.0(8) | 22.8(8) | 6.7(6) | 1.6(7) | 3.8(6) |
| C10 | 26.1(9) | 23.0(8) | 16.9(8) | 6.3(6) | -1.1(7) | -1.5(7) |
| C11 | 26.8(8) | 18.5(8) | 11.7(7) | 2.0(6) | 4.9(6) | -4.4(6) |
| C12 | 13.4(7) | 11.7(7) | 11.5(6) | 0.9(5) | 3.9(5) | -0.1(5) |
| C13 | 12.7(7) | 12.5(7) | 13.5(6) | 0.7(5) | 4.8(5) | -1.7(5) |
| C14 | 13.8(7) | 13.2(7) | 12.9(6) | -1.2(5) | 4.8(5) | -2.5(5) |
| C15 | 18.4(8) | 15.5(7) | 18.6(7) | -0.3(6) | 9.7(6) | -2.5(6) |
| C16 | 16.1(7) | 21.8(8) | 14.6(7) | -0.5(6) | 3.3(6) | -3.8(6) |

Table 4 Bond Lengths for 0961_PQ_Snyder_2.

| Atom | Atom | Length/Å | Atom | Atom | Length/Å |
|---|---|---|---|---|---|
| O1 | C1 | 1.2402(19) | C5 | C6 | 1.525(2) |
| O2 | C4 | 1.4199(18) | C5 | C14 | 1.570(2) |
| O3 | C7 | 1.4644(19) | C6 | C7 | 1.515(2) |
| O3 | C15 | 1.3464(19) | C7 | C8 | 1.534(2) |
| O4 | C15 | 1.2037(19) | C8 | C9 | 1.520(2) |
| N1 | C1 | 1.352(2) | C8 | C12 | 1.543(2) |
| N1 | C11 | 1.4761(19) | C9 | C10 | 1.518(2) |
| N1 | C12 | 1.4699(18) | C10 | C11 | 1.508(2) |
| C1 | C2 | 1.476(2) | C12 | C13 | 1.532(2) |
| C2 | C3 | 1.327(2) | C13 | C14 | 1.554(2) |
| C3 | C4 | 1.504(2) | C14 | C15 | 1.512(2) |
| C4 | C5 | 1.545(2) | C14 | C16 | 1.517(2) |
| C4 | C12 | 1.533(2) | | | |

Table 5 Bond Angles for 0961_PQ_Snyder_2.

| Atom | Atom | Atom | Angle/° | Atom | Atom | Atom | Angle/° |
|---|---|---|---|---|---|---|---|
| C15 | O3 | C7 | 116.61(12) | C7 | C8 | C12 | 111.87(12) |
| C1 | N1 | C11 | 117.06(13) | C9 | C8 | C7 | 113.61(13) |
| C1 | N1 | C12 | 119.00(12) | C9 | C8 | C12 | 112.27(13) |
| C12 | N1 | C11 | 118.21(12) | C10 | C9 | C8 | 108.45(13) |
| O1 | C1 | N1 | 121.23(15) | C11 | C10 | C9 | 111.91(13) |
| O1 | C1 | C2 | 120.37(15) | N1 | C11 | C10 | 111.66(13) |
| N1 | C1 | C2 | 118.40(13) | N1 | C12 | C4 | 112.30(12) |
| C3 | C2 | C1 | 122.82(15) | N1 | C12 | C8 | 111.42(12) |
| C2 | C3 | C4 | 119.58(14) | N1 | C12 | C13 | 111.18(12) |
| O2 | C4 | C3 | 106.97(12) | C4 | C12 | C8 | 107.91(12) |
| O2 | C4 | C5 | 114.03(12) | C13 | C12 | C4 | 101.74(11) |
| O2 | C4 | C12 | 110.44(12) | C13 | C12 | C8 | 111.88(12) |
| C3 | C4 | C5 | 114.44(12) | C12 | C13 | C14 | 104.73(11) |
| C3 | C4 | C12 | 110.47(12) | C13 | C14 | C5 | 104.43(11) |
| C12 | C4 | C5 | 100.42(11) | C15 | C14 | C5 | 111.72(12) |
| C4 | C5 | C14 | 104.41(11) | C15 | C14 | C13 | 104.42(12) |
| C6 | C5 | C4 | 108.15(12) | C15 | C14 | C16 | 110.77(12) |
| C6 | C5 | C14 | 109.50(12) | C16 | C14 | C5 | 113.16(12) |
| C7 | C6 | C5 | 106.49(12) | C16 | C14 | C13 | 111.86(12) |
| O3 | C7 | C6 | 106.94(12) | O3 | C15 | C14 | 115.71(13) |
| O3 | C7 | C8 | 113.67(13) | O4 | C15 | O3 | 118.82(15) |
| C6 | C7 | C8 | 112.76(13) | O4 | C15 | C14 | 125.12(15) |

Table 6 Torsion Angles for 0961_PQ_Snyder_2.

| A | B | C | D | Angle/° | A | B | C | D | Angle/° |
|---|---|---|---|---|---|---|---|---|---|
| O1 | C1 | C2 | C3 | 175.92(16) | C6 | C5 | C14 | C16 | -138.46(13) |
| O2 | C4 | C5 | C6 | 41.62(16) | C6 | C7 | C8 | C9 | 177.11(13) |
| O2 | C4 | C5 | C14 | 158.16(12) | C6 | C7 | C8 | C12 | 48.71(17) |
| O2 | C4 | C12 | N1 | 71.14(15) | C7 | O3 | C15 | O4 | 161.41(14) |
| O2 | C4 | C12 | C8 | -52.05(15) | C7 | O3 | C15 | C14 | -25.02(18) |
| O2 | C4 | C12 | C13 | -169.90(11) | C7 | C8 | C9 | C10 | 174.53(13) |
| O3 | C7 | C8 | C9 | 55.17(17) | C7 | C8 | C12 | N1 | 178.54(12) |
| O3 | C7 | C8 | C12 | -73.23(16) | C7 | C8 | C12 | C4 | -57.74(16) |
| N1 | C1 | C2 | C3 | -4.7(2) | C7 | C8 | C12 | C13 | 53.37(16) |

Table 6 Torsion Angles for 0961_PQ_Snyder_2.

| A | B | C | D | Angle/˚ | A | B | C | D | Angle/˚ |
|---|---|---|---|---|---|---|---|---|---|
| N1 | C12 | C13 | C14 | 159.50(11) | C8 | C9 | C10 | C11 | 59.28(18) |
| C1 | N1 | C11 | C10 | -160.51(14) | C8 | C12 | C13 | C14 | -75.20(14) |
| C1 | N1 | C12 | C4 | 41.96(18) | C9 | C8 | C12 | N1 | 49.44(17) |
| C1 | N1 | C12 | C8 | 163.15(13) | C9 | C8 | C12 | C4 | 173.16(12) |
| C1 | N1 | C12 | C13 | -71.29(17) | C9 | C8 | C12 | C13 | -75.72(15) |
| C1 | C2 | C3 | C4 | -4.0(2) | C9 | C10 | C11 | N1 | -53.07(18) |
| C2 | C3 | C4 | O2 | -90.57(17) | C11 | N1 | C1 | O1 | 10.8(2) |
| C2 | C3 | C4 | C5 | 142.10(15) | C11 | N1 | C1 | C2 | -168.53(14) |
| C2 | C3 | C4 | C12 | 29.67(19) | C11 | N1 | C12 | C4 | -165.46(13) |
| C3 | C4 | C5 | C6 | 165.24(12) | C11 | N1 | C12 | C8 | -44.26(17) |
| C3 | C4 | C5 | C14 | -78.21(15) | C11 | N1 | C12 | C13 | 81.29(16) |
| C3 | C4 | C12 | N1 | -47.00(16) | C12 | N1 | C1 | O1 | 163.72(15) |
| C3 | C4 | C12 | C8 | -170.19(12) | C12 | N1 | C1 | C2 | -15.6(2) |
| C3 | C4 | C12 | C13 | 71.96(14) | C12 | N1 | C11 | C10 | 46.38(18) |
| C4 | C5 | C6 | C7 | 68.30(15) | C12 | C4 | C5 | C6 | -76.46(14) |
| C4 | C5 | C14 | C13 | -15.94(14) | C12 | C4 | C5 | C14 | 40.08(14) |
| C4 | C5 | C14 | C15 | -128.23(12) | C12 | C8 | C9 | C10 | -57.29(17) |
| C4 | C5 | C14 | C16 | 105.94(14) | C12 | C13 | C14 | C5 | -14.46(14) |
| C4 | C12 | C13 | C14 | 39.76(14) | C12 | C13 | C14 | C15 | 102.97(13) |
| C5 | C4 | C12 | N1 | -168.17(11) | C12 | C13 | C14 | C16 | -137.19(13) |
| C5 | C4 | C12 | C8 | 68.64(13) | C13 | C14 | C15 | O3 | -60.53(16) |
| C5 | C4 | C12 | C13 | -49.21(13) | C13 | C14 | C15 | O4 | 112.59(16) |
| C5 | C6 | C7 | O3 | 73.38(14) | C14 | C5 | C6 | C7 | -44.89(16) |
| C5 | C6 | C7 | C8 | -52.27(16) | C15 | O3 | C7 | C6 | -36.99(17) |
| C5 | C14 | C15 | O3 | 51.77(17) | C15 | O3 | C7 | C8 | 88.12(16) |
| C5 | C14 | C15 | O4 | -135.12(16) | C16 | C14 | C15 | O3 | 178.91(13) |
| C6 | C5 | C14 | C13 | 99.66(13) | C16 | C14 | C15 | O4 | -8.0(2) |
| C6 | C5 | C14 | C15 | -12.62(16) | | | | | |
